# Time-Series Niche Modelling Reveals Declining Tendencies of Habitat Suitability and Ecological Functions in a Mountainous Protected Area

**DOI:** 10.1007/s00267-026-02393-5

**Published:** 2026-02-18

**Authors:** Inês Freitas, João Alírio, Nuno Garcia, João C. Campos, Salvador Arenas-Castro, Isabel Pôças, Lia Duarte, Ana C. Teodoro, Neftalí Sillero

**Affiliations:** 1 Faculty of Sciences, CICGE—Centro de Investigação em Ciências GeoEspaciais, University of Porto, Vila Nova de Gaia, Portugal; 2 Department of Geosciences, Environment and Land Planning, Faculty of Sciences, University of Porto, Porto, Portugal; 3 Earth Sciences Institute (ICT), University of Porto, Porto, Portugal; 4 Centro de Investigação em Biodiversidade e Recursos Genéticos (CIBIO), InBIO Laboratório Associado, Universidade do Porto, Vairão, Portugal; 5 BIOPOLIS Program in Genomics, Biodiversity and Land Planning, CIBIO, Vairão, Portugal; 6 Área de Ecología, Departamento de Botánica, Ecología y Fisiología Vegetal, Facultad de Ciencias, Centro Andaluz de Investigación de Zoonosis y Enfermedades Emergentes CAIZEM, Universidad de Córdoba, Córdoba, Campus de Rabanales Spain; 7 CoLAB ForestWISE—Collaborative Laboratory for Integrated Forest & Fire Management, Quinta de Prados, Vila Real, Portugal

**Keywords:** Biodiversity monitoring, Ecological niche models, Habitat suitability index, Satellite remote sensing, Time series

## Abstract

Monitoring biodiversity in protected areas is essential to mitigate biodiversity loss and evaluate the effectiveness of conservation policies. Integrating satellite remote sensing technologies, ecological niche models, and time-series analyses of biodiversity trends offers a fast and robust approach for assessing habitat suitability changes and species vulnerability over time. In this study, we implemented a framework combining these tools to monitor biodiversity in the Montesinho/Nogueira Special Conservation Area (Northeast Portugal). Using the MaxEnt algorithm, we generated ecological niche models for 342 species based on a time series (2001–2023) of remote sensing data from the Moderate-Resolution Imaging Spectroradiometer (MODIS) sensor. We analysed habitat suitability trends with the Mann-Kendall test to detect changes in habitat quality, as a metric of species vulnerability for individual species, five major taxonomic groups (vascular flora, amphibians, reptiles, birds, and mammals), functional groups (e.g. climate affinity, habitat type, diet, activity, reproduction), and conservation status (regional and European levels). Our study revealed a significant decline in habitat suitability over the past two decades, impacting all taxonomic groups and ecological functions. We observed a high variability in habitat suitability trends among species and taxonomic/functional groups, highlighting the complexity of biodiversity responses to environmental changes. Functional traits such as climatic affinity, trophic level or habitat specialisation were associated with variable rates of habitat decline, with species of Atlantic affinity, species associated with croplands and wetlands, and species specialised in insectivorous diets being at higher risk. Overall, these findings emphasise the need for comprehensive biodiversity monitoring programmes and demonstrate the utility of our approach to inform evidence-based conservation strategies in protected areas globally.

## Introduction

The rapid decline in global biodiversity, driven by human factors such as land-use change, climate change, pollution, and the proliferation of invasive species, poses a significant conservation challenge (Waldron et al. 2017). These pressures disrupt entire ecosystems by affecting habitat quality and destabilising ecological processes on which species rely. Addressing this biodiversity crisis requires urgent and effective conservation strategies to mitigate these impacts and restore ecosystems (CBD 2022).

Protected areas (PAs) are central to biodiversity conservation strategies, playing a key role by limiting human activities and serving as refuge for species (Watson et al. 2014), while hosting important ecosystem services (Pu et al. 2023). Covering ~15% of Earth’s land, PAs act as primary defences against biodiversity loss (Jones et al. 2018). Efforts to enhance their coverage, management, and effectiveness have been undertaken globally. Conservation policies and actions implemented in these areas are often tailored to local ecological and social contexts, but common initiatives include large-scale habitat restoration projects (e.g. through reforestation with native species), promoting sustainable agriculture and forestry practices, regulating hunting/fishing, establishing ecological corridors to connect fragmented habitats and support species movement, and fostering sustainable resource use while reducing conflicts with local communities (Mokany et al. 2020, Maxwell et al. 2020). Despite these efforts, PAs face growing challenges due to ongoing human pressure and the impacts of climate change (Jones et al. 2018). Continuous monitoring is critical to assess their effectiveness and ensure they achieve conservation goals in the face of these evolving threats (CBD 2022).

The integration of satellite remote sensing (SRS) data/technologies and ecological niche models (ENMs) has opened new pathways for monitoring biodiversity, offering high-resolution assessments of species and their habitats over time (see Pasetto et al. 2018, Leitão and Santos 2019, Arenas-Castro and Sillero 2021, Regos et al. 2022, Campos et al. 2023, Garcia et al. 2024b). SRS provides extensive data with high resolution across spatial, temporal, spectral, and radiometric dimensions, capturing real-time ecological conditions. Key integrative ecosystem indicators driving species’ distribution, such as the enhanced vegetation index (EVI), normalised difference vegetation index and land surface temperature (LST) retrieved from optical sensors, are accessible for broad conservation applications (Gorelick et al. 2017). Correlative ENMs (Sillero 2011, Sillero et al. 2021) use species occurrence data and environmental variables to predict habitat suitability and have become instrumental in describing and forecasting global changes in biodiversity in the Anthropocene (see Guisan et al. 2013). While ENMs are widely applied in conservation, integrating SRS data can provide greater accuracy in spatial and temporal predictions (José-Silva et al. 2018, Arenas-Casto et al. 2018, 2019, Radin et al. 2020, Regos et al. 2022).

In addition to these tools, time-series analyses of biodiversity trends have emerged as a paramount tool for biodiversity monitoring. Arenas-Castro and Sillero (2021) introduced a method that uses successive ENMs to assess species vulnerability by analysing habitat suitability trends over time. This method combines Maximum Entropy (MaxEnt) models (Phillips et al. 2006, 2017) and the Mann-Kendall test (Kendall 1990) to determine whether habitat suitability remains stable, increases, or decreases over time. Applying these methods (i.e. RS and biodiversity trend analyses) within PAs can be highly effective in monitoring habitat quality, assessing species vulnerability over time, and informing targeted conservation strategies for protecting the most at-risk species and habitats (Luque et al. 2018).

In this study, we applied the workflow published by Arenas-Castro and Sillero (2021) to monitor the biodiversity in the Montesinho/Nogueira Special Conservation Area (MNPN), a European Union’s Natura 2000 site in north-east Portugal. We estimated species vulnerability within the MNPN by analysing habitat suitability trends (using the Mann-Kendall test) from 2001 to 2023, derived from ENMs (using the MaxEnt algorithm) calculated with a time series of remote sensing variables (MODIS sensor). The MNPN (~107347 hectares), located in a transition zone between the Mediterranean and Atlantic biogeographic regions, host a high number of species, including Iberian endemisms and key species of high conservation value (Castro et al. 2010, Garcia et al. 2024a, b). The park encompasses a mountainous agricultural landscape impacted by recent land-use changes due to the expansion of agroforestry systems and shrublands at the expense of agricultural areas (Castro 2010, Campos et al. 2024), representing typical rural mountain areas (and associated ecological trends and characteristics) found across European regions. These changes pose a significant challenge to local biodiversity, highlighting the need to comprehensively evaluate species extinction risk within the MNPN. Through this study, we aim to analyse habitat suitability trends and estimate species-based and taxonomic/functional vulnerability in the MNPN, providing essential insights for conservation policy and biodiversity management.

## Material and Methods

### Study Area

**Fig. 1:**
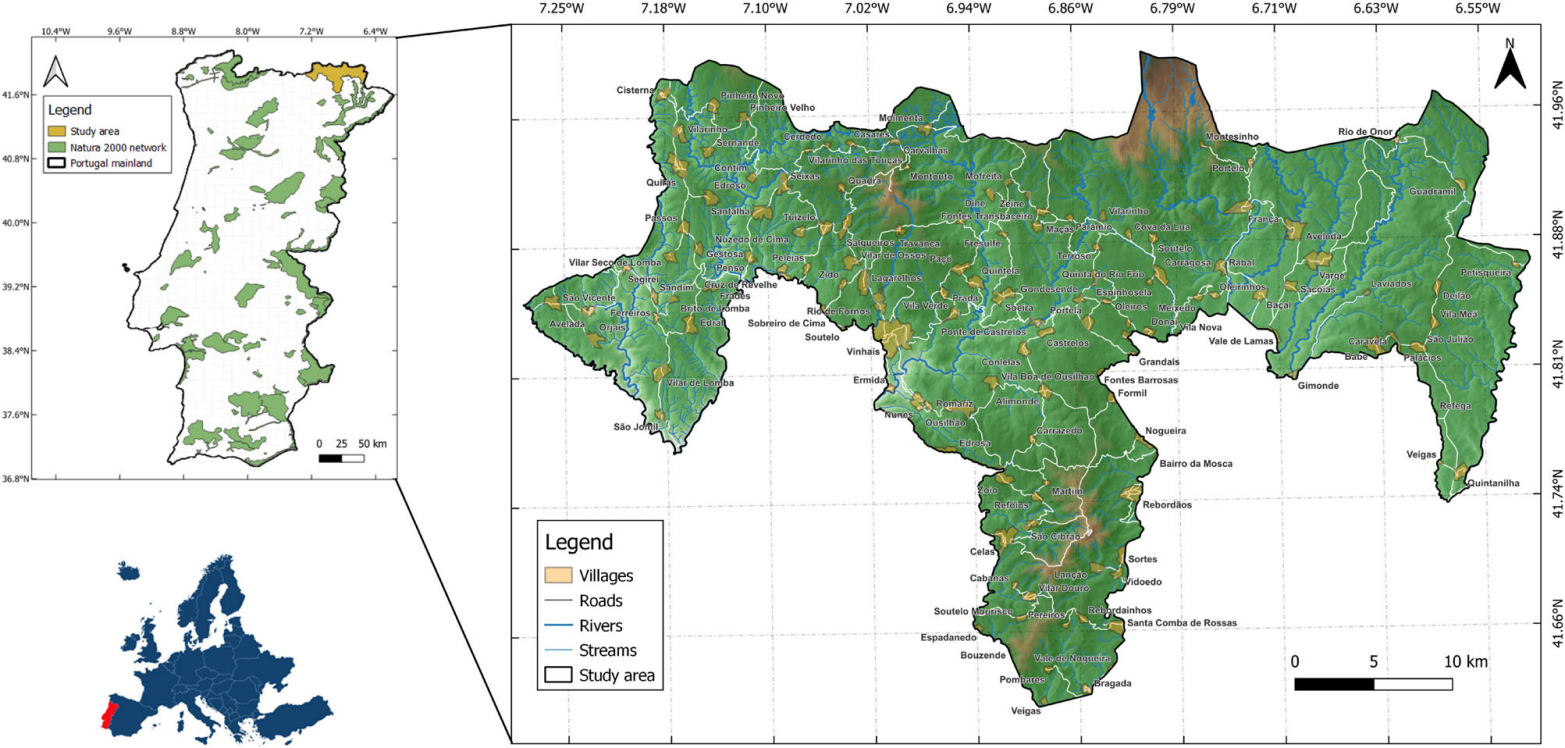
View of the Montesinho/Nogueira Special Conservation Area (MNPN). The background is a digital model terrain from the Portuguese Directorate General of the Territory (DGT; https://www.dgterritorio.gov.pt/). This figure was created using QGIS software Version 3.28.1 (https://www.qgis.org/)

The MNPN is an EU’s Natura 2000 site (Fig. 1). Located in north-east Portugal, the MNPN encompasses a large area of 107347 hectares that includes the Montesinho Natural Park with 74225 hectares (MNP; Castro et al. 2010) and the Nogueira Mountains (NM). The MNPN is located in the transition zone between the Mediterranean and Atlantic biogeographic regions and is influenced by the supra and oro-Mediterranean and Atlantic bioclimates. The altitudinal range varies between 380 and 1472 m. The region is characterised by dry-warm summers (July–September) and cool-wet winters (January–March), registering seasonal temperature fluctuations ranging from −12 °C to 40 °C (Deitch et al. 2017). The MNPN predominantly features an extensive mountainous agricultural landscape with wide-ranging areas of dry heathland, sweet chestnut (*Castanea sativa* Mill. (1768)) groves, shrublands, fragmented forests of oaks and pines, and rocky areas with sparse vegetation (Castro 2010, Campos et al. 2024). Over the last three decades, this region has been significantly affected by land abandonment and the expansion of agroforestry systems (Castro 2010, Campos et al. 2024). Home to many rare and endemic Iberian species, such as the Iberian wolf (*Canis lupus signatus)*, the roe deer *(Capreolus capreolus)*, the pine marten *(Martes martes)* and the wildcat *(Felis silvestris*), as well as priority habitats like oak forests, meadows, grasslands, and bushlands, the MNPN has the highest biodiversity in northern Portugal, making it a valuable site for conservation (Castro et al. 2010, Garcia et al. 2024a, Campos et al. 2024).

### Occurrence Data

The occurrence data used in this study consists of 16238 presence records of 342 species from five major taxonomic groups—vascular plants, amphibians, reptiles, birds, and mammals—chosen based on their well-documented occurrence data and ecological significance within the park (Online Resource 1). Among the 342 species analysed, 15 have been classified as key species of high conservation value at the regional level (ICNF, https://icnf.pt/). We identified these species based on their critical ecological roles, such as maintaining ecosystem stability and functioning, their ability to act as umbrella species by providing conservation benefits to a wide range of co-occurring species and habitats, and their cultural and symbolic significance.

Our dataset represents a subset of a larger database containing 32.587 species occurrence records from the study area, documenting 1.311 species (see Garcia et al. 2024a). We collected these data from various sources, such as online databases (e.g., Global Biodiversity Information Facility—GBIF, iNaturalist) and distribution atlases covering the period from 2001 to 2022, and fieldwork expeditions conducted between 2022-2023 under the MontObEO project—Montesinho Biodiversity Observatory (Garcia et al. 2024a). Given the heterogeneous and aggregated nature of citizen science and biodiversity data, we applied a series of quality-control filters prior to analysis. We manually removed records with nomenclature errors, missing or erroneous coordinates, or insufficient spatial accuracy, as well as doubtful observations falling outside the known species distributions or beyond the limits of the study area. We aggregated the remaining occurrence records to a 1 km grid, and removed duplicated records. We did not consider species with fewer than 15 occurrence records to ensure robust and reliable model predictions. A detailed description of dataset preparation is provided in Garcia et al. (2024a).

We collected information regarding relevant functional traits and the IUCN (International Union for Conservation of Nature) conservation status from regional and European assessments for each species analysed (see Online Resource 1). Traits and conservation data were obtained from publicly available databases (Animal Diversity Web; https://animaldiversity.org/; International Union for Conservation of Nature Red List; https://www.iucnredlist.org/; Bencatel et al. 2019; Flora-On: Flora de Portugal Interactiva, 2024; https://www.flora-on.pt/). The functional traits considered in this study (see Online Resource 1) included: (i) diet (carnivore, granivore, insectivore, omnivore and herbivore); (ii) habitat preference (croplands, forests, generalist, grasslands, open-woodlands, rocky outcrops, shrublands, water and wetlands); (iii) feeding type (herbivore and predator); (iv) climate affinity (Atlantic, Mediterranean and generalist); (v) activity (diurnal and nocturnal); (vi) photosynthesis type (C3 (three-carbon compound) plants adapted to moderate sunlight, temperature, and water availability, and vii) CAM (crassulacean acid metabolism) plants adapted to arid regions and tropical epiphytes) and plants reproduction type (anemophily and zoophily). We selected these traits for their capacity to capture key ecological roles and adaptive strategies of species, including trophic interactions and resource use, climatic and physiological adaptations and pollination strategies. Together, they provide a comprehensive framework for assessing biodiversity patterns, ecosystem functioning, and species’ vulnerability to environmental threats.

### Environmental Data

We initially considered nineteen MODIS satellite-derived products (Online Resource 2) available on Google Earth Engine (GEE; https://earthengine.google.com/). These products reflected both natural ecosystem functions (such as vegetation productivity, heat exchange and albedo), climatic effects (such as land surface temperature) and anthropogenic influences (such as fire regimes) (Arenas-Castro and Sillero 2021). We calculated annual averages for these products from 2001 to 2023 and aggregated them into 1 km grid cells using mean values within each grid cell, to match the spatial resolution of occurrence data. We assessed multicollinearity using Variance Inflation Factors (VIF) and the Pearson correlation coefficient and selected a final set of six predictors with VIF scores below 4 and correlations below 0.75. Multicollinearity and correlation analyses were performed in R using the packages “raster”, “usdm”, “corrplot” and “GGally” (Hijmans et al. 2022, Naimi 2017, Schloerke et al. 2021, Wei et al. 2021).

The chosen predictors included EV, surface reflectance (SR), day land surface temperature (LSTd), and night land surface temperature (LSTn), obtained directly from MODIS products. We processed these variables by applying product-specific scale factors and quality assurance masks to exclude pixels affected by clouds or other known artefacts, following standard MODIS data preprocessing procedures implemented in GEE. Additionally, we derived fire-related variables, namely area annually burned (AAB) and time since fire (TSF), from the MODIS Burned Area Monthly Global product, which required no additional scale corrections or quality masks (see Online Resource 2 for predictors details). AAB identifies cells where fire occurred each year (coded as 1 for fire presence, 0 for absence), while TSF represents the time since the last fire event. All data processing was conducted within the GEE platform (scripts available at https://github.com/SpatialBioLab).

### Modelling Procedure

#### MaxEnt Modelling

We calculated species’ realised niche models (sensu Sillero 2011) for each species using the Maximum Entropy (MaxEnt) algorithm, implemented in GEE (Campos et al. 2023). MaxEnt is a widely used correlative approach for modelling habitat suitability with presences and background data that contrasts environmental conditions at known presence locations with a large sample of locations across the study area (background records; Phillips et al. 2006, 2017). We applied standard procedures (Sillero et al. 2021, Sillero and Barbosa 2021), namely, ten replicate models for each year of the study period 2001–2023 to control for sampling variance; a random selection of 70% of records for training and 30% for testing in each replicate; and default parameters across models to ensure consistency and enable cross-species comparisons. We created a total of 870 background points within the MNP, corresponding to the centroids of all 1 km grid cells covering the study area. Annual habitat suitability was calculated as the mean of the ten replicate models.

We assessed model performance for each replicate using the area under the curve (AUC), which provides a robust, threshold-independent measure of presence/absence discrimination power (Lawson et al. 2014). Each model was trained on occurrence data from the MNP and projected to the whole MNPN, including the adjacent NM which has fewer occurrence data but holds significant conservation importance. In addition, we calculated null models following Raes and Steege (2007) methodology to further evaluate the performance of the MaxEnt models. To do this, we generated 100 different datasets with the same number of random points as each dataset following a Poisson distribution. MaxEnt models were generated in GEE for each random dataset in the same way as the empirical models. Then, we compared the training AUC values of the species models with the ones calculated for the null models using the non-parametric Wilcoxon test for repeated measures.

### Mann-Kendall Test and Habitat Suitability Trends Computation

To assess trends over time in habitat suitability across the study period, we applied the non-parametric Mann-Kendall test (Arenas-Castro and Sillero 2021, Kendall 1990) to detect monotonic trends in time series data, using the sensSlope function available within GEE. This method uses the annual Habitat Suitability Index (HSI) at each grid cell generated by MaxEnt to compute slope values representing changes in Habitat Suitability (HS) over the study period and an associated *p* value. Specifically, this method compares every HS value to every preceding value in the time series. If a trend is present, the significant values will tend to increase or decrease constantly (see Arenas-Castro and Sillero 2021).

### Habitat Suitability Trends Analyses for Individual Species, Taxonomic and Functional Groups and Conservation Levels

We first applied a two-step masking process to the habitat suitability trend maps: each map was masked by the Mann-Kendall test *p* values, preserving the grid cells with statistically significant trends (*p* < 0.05); and by the distribution range of the species to ensure that only significant habitat suitability trends within the species’ distribution range were retained (Arenas-Castro and Sillero 2021).

We then calculated the mean trend slopes across all species to assess overall biodiversity trends within the MNPN over the past two decades. To provide greater detail on temporal biodiversity patterns, we also examined trends across taxonomic groups, functional groups, and conservation statuses based on IUCN regional and European categories (https://www.iucn.org/). Additionally, we estimated HS trends for plant species occurring at traditional mountain semi-natural pastures (lameiros, in Portuguese), which are biodiversity-rich habitats with strong cultural significance. These pastures support endemic and threatened species but are increasingly at risk due to land abandonment and changing agricultural practices (Campos et al. 2024).

For each group, we aggregated the suitability trend maps of individual species and calculated the average trend and standard deviation. Finally, we also quantified the proportion of significant pixels with negative and positive trend values for each species and group. Negative trend slopes indicate a significant decline in habitat suitability and serve as a metric of extinction risk (Arenas-Castro and Sillero 2021, Sillero et al. 2024). To compare the proportion of negative habitat suitability trends among taxonomic groups, functional groups, and conservation status categories, we performed an analysis of variance (ANOVA), followed by post hoc Tukey’s Honest Significant Difference (HSD) tests to identify significant pairwise differences between groups.

All analyses were conducted using the R programming language (R version 4.0.3) and the “terra” package (scripts available at https://github.com/SpatialBioLab).

## Results

### Habitat Suitability Trends: Individual Species, Global, and Groups (Taxonomic, Functional and Conservation Statuses)

**Fig. 2:**
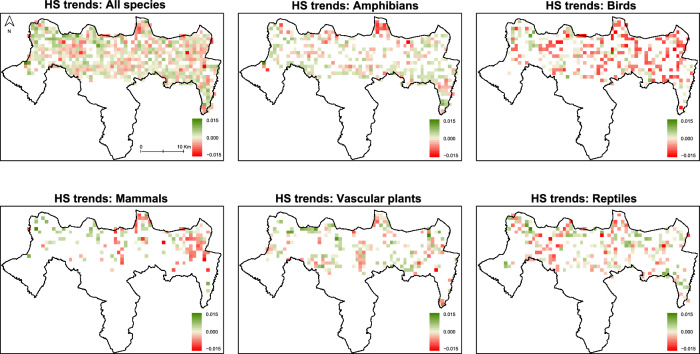
Spatial distribution of mean habitat suitability trends (slopes) over time (2001–2023) for all species and taxonomical groups (from left to right, by row: vascular flora, amphibians, reptiles, birds, and mammals). Red colours indicate negative trends; blue, positive trends; white, no trends

**Fig. 3:**
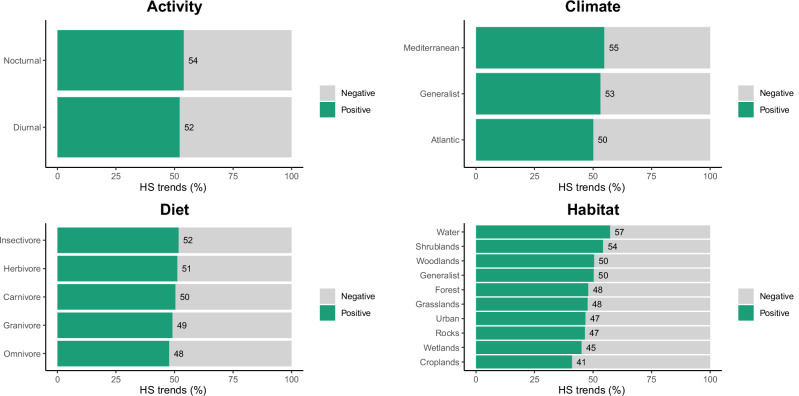
Proportion of significant pixels with positive (green) and negative (grey) habitat suitability trends between 2001 and 2023 for the most relevant functional traits (climatic affinity, activity type, diet and habitat type)

Overall, individual-species models performed relatively well. Empirical models presented higher AUC values than null models, either for training data (Wilcoxon test for paired data: *V* = 3.10e9, *p* < 0.0001; mean AUC empirical: 0.73 ± 0.07; mean AUC null: 0.68 ± 0.06) or test data (Wilcoxon test for paired data: *V* = 3.10e9, *p* < 0.0001; mean AUC empirical: 0.59 ± 0.11; mean AUC null: 0.5 ± 0.11). HS trend analyses revealed that most species and groups have experienced a decline in habitat suitability over the past 20 years in roughly half of their distribution area (Fig. 2; Online Resource 1, 4, 5). With some exceptions, we report a similar percentage of negative habitat trends across functional groups (Fig. 3, Online Resource 3, 5, 6).

Considering all species combined, 48.2% of the analysed grid cells displayed negative HS trends, reflecting a widespread reduction in habitat quality for MNPN biodiversity (Fig. 2). The spatial distribution of these areas reveals a complex and fragmented landscape, with areas of decreasing HS scattered throughout the landscape. Examining each taxonomic group, birds exhibited the highest percentage of negative trends (53.2%), followed closely by reptiles (49.8%), mammals (48.9%), and plants (48.2%). Amphibians showed the lowest levels of habitat degradation (43.2%). Notably, some areas with negative and positive HS trends are consistent across the different groups (Fig. 2).

HS trends were similar for species with distinct climatic affinity, but Atlantic species showed the highest percentage of cells with negative trends, followed by generalist species and Mediterranean species (Fig. 3). This pattern is held when each taxonomic group is examined separately (Online resource 6). In reptiles, the difference between Mediterranean and Atlantic species is more pronounced, with Atlantic species showing negative trends in HS for 60% of the area.

Species with distinct habitat selection exhibited varying degrees of HS decline, with species adapted to cropland areas experiencing the most substantial loss over time (59%). In accordance with our model predictions, generalists and species inhabiting woodlands and shrublands exhibited a lower decrease in HS, although over 40% of their distribution ranges were still affected (Fig. 3). However, HS trends varied significantly across taxonomic groups. For vascular plants, our models indicated that the most impacted species were those inhabiting wetlands and croplands. Amphibians were most affected in woodlands and wetlands, reptiles in woodlands and rocky habitats, and birds in wetlands and forests (Online Resource 6).

The remaining functional groups exhibited similar patterns of HS decline. Activity, feeding, and diet types were not strong differentiators, as all groups exhibited HS declines of around 50% (Fig. 3). However, when examining the taxonomic groups separately, the differences between diet groups became more pronounced. Among mammals and reptiles, carnivores exhibited the most positive HS trends, contrasting sharply with the declines observed for omnivores and insectivores (Online Resource 6). For vascular plants, C3 and CAM photosynthesis types and zoophilous and anemophilous reproduction types also exhibited similar trends. Similarly, for the plants that compose the priority habitat of traditional mountain pastures—lameiros—around half (49.3%) of the area shows a decrease in HS.

**Fig. 4:**
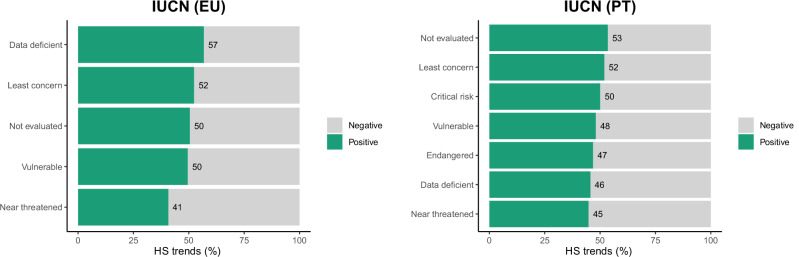
Proportion of significant pixels with positive (green) and negative (grey) habitat suitability trends between 2001 and 2023 per European and regional conservation status

Species classified as Near Threatened (NT) on the European Red List exhibited the most substantial reduction in habitat quality, with 67.1% of grid cells showing negative trends—sharply contrasting with the 28.8% observed for NT species in regional assessments, reflecting differences in species listings. Least Concern (LC) and Vulnerable (VU) species also experienced substantial HS reductions. We observed similar trends for the species listed as Endangered (EN) in regional assessments but not for the Critically Endangered (CR) species, which are experiencing an increase of HS in over two-thirds of their distribution according to our models. Additionally, species with insufficient data (Data Deficient; DD), and particularly those Not Evaluated (NE) in previous assessments, showed high levels of habitat quality decline across the MNPN (Fig. 4).

Looking at the HS trends of individual species, out of the 342 species analysed, only 15 exhibited negative habitat trends in less than 25% of the analysed grid cells within their distribution (cells with non-significant trends were not considered)—all of which were plant species. A total of 207 species experienced declines in HS across more than half of the area, where notably 32 presented habitat declines higher than 75%. Among these, 18 species showed negative trends in all evaluated cells (Online Resource 1).

### Habitat Suitability Trends for Key Species

**Fig. 5:**
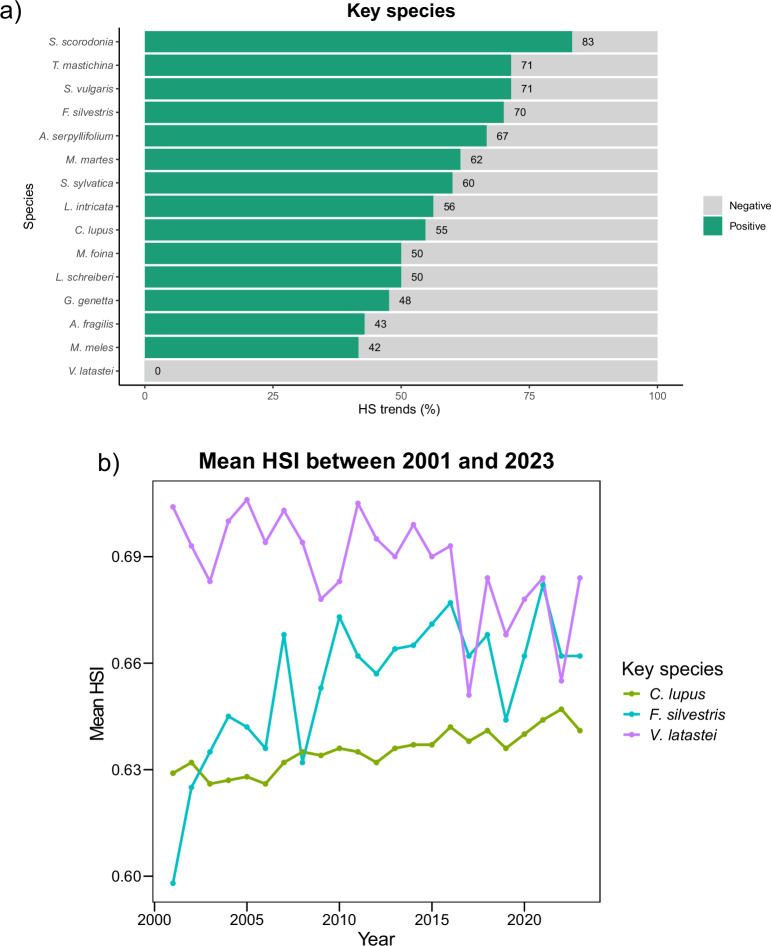
**a** Proportion of significative pixels with positive (green) and negative (grey) habitat suitability trends between 2001 and 2023 for the 15 key species, **b** Mean habitat suitability index between 2001 and 2023 for three key species, *Canis lupus*, *Felis silvestris* and *Vipera latastei*

Among the key species of high conservation value at the regional level, HS trends reveal considerable variation (Fig. 5). For plants, *Scrophularia scorodonia* (Figwort) showed a negative trend in habitat suitability in only 16.7% of the analysed cells, while *Thymus mastichina* (Spanish marjoram) and *Linaria intricata* (Intricate toadflax) exhibited declines in 28.6% and 43.8% of them, respectively. In reptiles, *Lacerta schreiberi* (Schreiber’s green lizard) and *Anguis fragilis* (Slowworm) demonstrated a consistent decline in HS over more than half the grid cells, while *Vipera latastei* (Lataste’s viper) experienced a decrease in HS in all of them. Following our model predictions, key mammal species also reflected concerning trends: *F. silvestris* (Wildcat) and *C. lupus signatus* (Iberian wolf), both with threatened statuses, exhibited HS declines in 30% and 45.3% of the analysed area, respectively. Similar patterns were observed for other key species, such as *M. martes* (Pine marten), *M. foina* (Beech marten), *Genetta genetta* (Common genet), and *Meles meles* (European badger), all showing habitat degradation across a substantial part of their ranges (Fig. 5a).

Across the 2001–2023 period, mean HS values for these key species fluctuated substantially within a narrow range (0.1–0.2) but followed no consistent pattern across species. Overall, HS patterns were highly variable and species-specific, with *M. martes* and *M. foina* being the only two species exhibiting similar profiles over time. Some species, such as *F. silvestris*, experienced pronounced HS fluctuations with sharp annual drops and increases. In contrast, the Iberian Wolf (*C. lupus signatus*) showed a relatively steady increase in mean HS over time, while *V. latastei* showed a consistent decline instead (Fig. 5b).

## Discussion

Our results reveal a significant decline in HS over the past two decades, affecting all taxonomic groups and ecological functions within the MNPN. Additionally, we report a high variability in HS trends among species, taxonomic groups and functional roles that highlight the complexity of biodiversity responses to environmental changes.

### Global and Taxonomic-Level Habitat Suitability Trends

The observed decline in HS across half of the grid cells of the MNPN, considering all species combined and the five main taxonomic groups, indicates widespread habitat degradation between 2001 and 2023. However, the fluctuations in HS indices remain moderate, with annual mean HS varying within a narrow range over the period of 22 years (maximum change of 0.2).

The spatial distribution of areas with positive trends in HS is largely sparse, suggesting low overall habitat connectivity and habitat fragmentation, particularly for species with high vagility, migratory behaviours or large distribution ranges. Nevertheless, a contiguous area in the northwestern region of the park has shown consistent increases in HS over the past two decades, potentially serving as an important refuge for the biodiversity of the MNPN. In contrast, several areas exhibit declines in habitat quality during the same period, with some overlap across taxonomic groups, such as the Montesinho region in the extreme north of the MNPN, a less-surveyed area with fewer species records. Other declines are more taxon-specific: mammals exhibit extensive areas with negative trends, mainly localised in the eastern part of the park (Alta Lombada). In contrast, negative trends for reptiles are primarily located in the western regions of the park. The northwestern part of the MNPN (including the Montesinho region) has been particularly affected by recurring wildfires over the last few decades (Pinho and Mateus 2018). These wildfires pose direct threats to species, especially to those with low mobility such as reptiles, but also drive significant Land Use and Land Cover (LULC) changes contributing to declines in HS (Parente et al. 2023).

Among the five taxonomic groups, birds exhibited the highest percentage of negative trends, being the only group with more than half of the area showing declines in HS, with those areas being widespread across the whole MNPN. These results, although observed at a local scale within a PA, are broadly consistent with global patterns of avian biodiversity loss. Globally, bird populations have experienced a steady deterioration in conservation status over the past three decades (BirdLife International 2020), along with a substantial decline in functional diversity—particularly in Europe and other temperate regions—driven mainly by land-use change, habitat loss, and climate change (Lees et al. 2022).

Birds play critical functional roles in ecosystems, acting as pollinators, seed-dispersers, scavengers, predators and pest controllers, linking ecosystem fluxes and maintaining biodiversity and ecosystem resilience (Lees et al. 2022). Thus, declines in their HS may have cascading effects, jeopardising the stability of the park’s ecosystems. Moreover, with more than 150 species in the MNPN—most of which classified as LC (Garcia et al. 2024a)—birds represent a significant portion of the park’s fauna, making their declines especially alarming.

Contrastingly, amphibians showed comparatively lower declines in HS, a trend that stands in sharp contrast to the current global-scale amphibian extinction crisis reported (Luedtke et al. 2023). Amphibians are recognised as the most threatened vertebrate class, with habitat loss, climate change, and lethal diseases being the primary drivers of their decline worldwide (Stuart et al. 2004, Luedtke et al. 2023). However, the higher percentage of positive trends observed in this study suggests that amphibian species may be less vulnerable in the MNPN, possibly benefitting from the good-quality and abundant water bodies of the region.

### Ecological Specialisation Shapes Species’ Vulnerability

Functional diversity, which encompasses the range of functional traits and ecological roles within an ecosystem, is crucial for maintaining ecosystem stability and resilience in the face of environmental change and is a key indicator of ecosystem health (Cadotte et al. 2011, Bonilla-Valencia et al. 2022). The loss of functional diversity is one of the most significant consequences of climate and landscape change, currently observed across all taxonomic groups on a global scale (Toussaint et al. 2021). Thus, monitoring HS trends of functional groups can offer valuable insights into the conservation state of biodiversity within PAs.

In our study, many functional traits showed consistent HS trends across classes. This suggests that specific traits or ecological roles often do not buffer species from habitat degradation or directly translate into higher extinction risk. However, some ecological traits—such as climatic affinity, trophic level or habitat specialisation—are shown to be associated with variable rates of habitat decline (see Toussaint et al. 2021). We observed a higher vulnerability of Atlantic species compared to Mediterranean species. The MNPN is located at the transition between the Atlantic and Mediterranean bioclimatic regions and hosts species with both climatic affinities (Castro et al. 2010, Garcia et al. 2024a, b); however, Mediterranean conditions predominate in the region and are expected to expand due to climate change (IPCC 2021). This shift comes at the expense of Atlantic species, which are more sensitive to global warming and increasing aridification (Somero 2011, Sousa-Guedes et al. 2020). This pattern is particularly marked in reptiles, where Atlantic species showed habitat suitability declines across nearly 60% of their distribution. As ectotherms, reptiles are highly sensitive to climatic fluctuations since their physiological functions depend on external temperature and water availability (Rohr and Palmer 2013, Greenberg and Palmer 2021). These factors likely exacerbate the vulnerability of Atlantic species, whose optimal environmental conditions are becoming increasingly restricted in time and space (Huey and Kingsolver 2019).

Habitat-specific trends revealed that species associated with croplands are the most affected, with 59% of their distribution areas undergoing a decline in habitat quality. Habitat-specific trends revealed that species associated with croplands are the most affected, with 59% of their distribution areas undergoing a decline in habitat quality. This decline is likely linked to ongoing land-use changes in the MNPN, which include land abandonment (which contributes to the increase of shrublands) and replacement of agricultural areas with agroforestry systems (Castro 2010, Campos et al. 2024). Consistently, species adapted to shrubland areas, particularly plants, reptiles and birds, showed notable improvements in habitat quality during the period analysed, reflecting the expansion of these habitats. Consistently, species adapted to shrubland areas, particularly plants, reptiles and birds, showed notable improvements in habitat quality during the period analysed, reflecting the expansion of these habitats.

Wetland-associated species, including vascular plants, birds and amphibians, also face substantial declines, highlighting the vulnerability of these critical ecosystems that support Iberian endemic species, such as the Iberian tree frog (*Hyla molleri*) and the Iberian painted frog (*Discoglossus galganoi)*. Similarly, reptiles, amphibians and mammals associated with woodlands showed significant declines in habitat quality over the last 20 years in the MNPN. Examples of these species are the Iberian endemic hare (*Lepus granatensis*), the roe deer (*C. capreolus*), and the red deer (*Cervus elaphus*). Moreover, some dietary specialisations play an important role in mediating species responses to habitat changes, particularly when analysing trends of each taxonomic group individually. A carnivorous diet is associated with significantly higher HS in reptiles and mammals. This trend may be linked to conservation initiatives and habitat restoration efforts targeting carnivorous species such as the Iberian wolf (*C. lupus signatus*).

In contrast, insectivorous reptiles and birds have experienced marked declines in habitat quality over the last two decades in the MNPN. These declines may be driven by significant reductions in invertebrate biomass, a phenomenon observed worldwide and largely attributed to LULC changes, climate change and pesticide pollution—prevalent threats in the region. The loss of invertebrates triggers cascading effects throughout the trophic chain, further impacting species that rely heavily on these food resources (Goulson 2019).

### Habitat Suitability Trends Generally Reflect Species Conservation Status

For species listed as VU and NT, the substantial reductions in habitat quality—especially the 67% decline for NT species on the European Red List—align with their vulnerable conservation status and reinforce the need for targeted conservation measures. Although these declines are observed within a PA, they suggest that current conservation actions may not fully mitigate the impacts of ongoing human threats, such as habitat loss and climate change (Arenas-Castro and Sillero 2021). For species classified as LC, the significant proportion of grid cells showing habitat declines indicates that these species, while not presently considered threatened, have faced increased pressures over the last 20 years (Castro 2010, Campos et al. 2024). Notably, CR species experienced improved habitat quality in two-thirds of their distribution, potentially reflecting targeted conservation actions and/or an effect of land abandonment and improved conditions in localised areas. However, the high levels of habitat decline observed for DD and NE species are concerning. These trends underscore the urgent need for comprehensive assessments to determine their conservation needs (Arenas-Castro and Sillero 2021).

### Ongoing Conservation Policies May Not Be Sufficient for Some Key Species

Umbrella species, often central to conservation strategies within protected and conservation areas, play a crucial role in safeguarding biodiversity. Their protection ensures their long-term survival and the persistence of many other species that share their habitats, have similar ecological roles, or rely on similar resources (Roberge et al. 2004, Branton and Richardson 2011). Results from the MNPN suggest a mixed outcome regarding the effectiveness of conservation actions targeting these species, with trends varying considerably among them. These trends differ both spatially, in the proportion of their species ranges experiencing declines or improvements in HS, and temporally, in the fluctuations of HS over time, reflecting varying degrees of sensitivity to environmental changes.

For instance, the steady increase in HS for the Iberian wolf (*C. lupus signatus*) during the last decades suggests that this species may benefit from targeted conservation efforts, including habitat restoration, prey management, and policies limiting human-wildlife conflict, such as the introduction of guard dogs to reduce livestock predation and improve coexistence with rural communities, implemented at the national level and also adopted within the MNPN (Torres and Fonseca 2016).

Conversely, the consistent decline in HS across the entire range of *V. latastei* (Lataste’s viper), a threatened Iberian endemic, raises significant concerns (Miras et al. 2009). Similarly, species such as *L. schreiberi* (Schreiber’s green lizard), *A. fragilis* (Slowworm), *M. meles* (European badger), and *G. genetta* (common genet) show habitat degradation across large portions of their ranges, suggesting potential gaps in conservation actions, despite their ecological importance and susceptibility to climatic and landscape changes. Potentially, future conservation actions may consider functional diversity for more efficient conservation of a broader and diverse spectrum of biodiversity essential for ecosystem resilience.

## Conclusion

Biodiversity monitoring is crucial for understanding how biological communities evolve over time, particularly in response to increasing environmental pressures. Field-based studies provide indispensable insights into population demographic trends and species distributions, but they often require years to build comprehensive datasets. In this context, monitoring frameworks that integrate remote sensing data, ENMs, and analyses of HS trends across both spatial and temporal scales offer a powerful complementary approach. These tools enable the fast identification of areas and species potentially at risk, helping to prioritise where detailed field-based investigation and conservation actions are most urgently needed. Using these methods, our study highlights areas of extensive habitat degradation and species facing the most significant threats within the MNPN, providing practical guidance to support conservation planning. When combined with targeted field surveys, such model-based assessments can improve the efficiency and effectiveness of conservation actions by focusing efforts on the most vulnerable species and regions. The framework here implemented can be applied to other national and international PAs, with the MNPN serving as a valuable case study in biodiversity monitoring, illustrating how predictive modelling can support evidence-based conservation.

### Electronic Supplementary Material

#### Online Resource 1

Summary of functional traits and the proportion of significative pixels showing positive and negative habitat suitability trends for the 342 individual species analysed in this study.

#### Online Resource 2

Summary of the candidate Moderate Resolution Imaging Spectroradiometer (MODIS) satellite-derived variables computed in Google Earth Engine considered in this study, indicating product name, spatial and temporal resolution, and rationale. Variables selected for model fitting are highlighted in bold.

#### Online Resource 3

Analysis of variance (ANOVA) and post hoc Tukey’s HSD tests to compare the proportion of negative habitat suitability trends among taxonomic groups, functional groups, and conservation status categories.

#### Online Resource 4

Maps of habitat suitability trends over time (2001–2023) for each species. Red colours indicate negative trends; green, positive trends; white, no trends.

#### Online Resource 5

Average and standard deviation maps of habitat suitability trends over time (2001-2023) for taxonomic groups (A), functional groups (B) and conservation status (C). Red colours indicate negative trends; green, positive trends; white, no trends.

#### Online Resource 6

Proportion of significative pixels with positive (green) and negative (grey) habitat suitability trends, summarised by functional trait.

## Supplementary information

ESM_1

ESM_2

ESM_3

ESM_4

ESM_5.A

ESM_5.B

ESM_5.C

ESM_6.A

ESM_6.B

ESM_6.C

ESM_6.D

## Data Availability

Occurrence data and scripts are available at https://github.com/SpatialBioLab.
