# Supplementary material for: Time-Series Niche Modelling Reveals Declining Tendencies of Habitat Suitability and Ecological Functions in a Mountainous Protected Area
Source: Environ Manage. 2026 Feb 18;76(3):101. doi: 10.1007/s00267-026-02393-5 (PMC12916538; doi:10.1007/s00267-026-02393-5)
Supplement: Supplementary file 1 — ESM_1 [file 267_2026_2393_MOESM1_ESM.pdf]

Online Resource 1 Summary of functional traits and the proportion of significative pixels showing positive and negative habitat suitability trends for the 342 individual species analysed in this study.

Species	% pixels with negative trend	Taxonomic group	Habitat	Climate	IUCN (EU)	IUCN (PT)	Feeding type	Diet	Activity	Photosynthesis	Reproduction
Alytes.obstetricans	47.92	amphibians	woodlands	atlantic	LC	LC	predator	insectivore	nocturnal		
Bufo.spinosus	44	amphibians	generalist	general	NE	NE	predator	insectivore	nocturnal		
Discoglossus.galganoi	33.33	amphibians	wetlands	mediterranean	LC	NT	predator	insectivore	nocturnal		
Epidalea.calamita	50	amphibians	generalist	mediterranean	LC	LC	predator	insectivore	nocturnal		
Hyla.molleri	55.56	amphibians	wetlands	mediterranean	LC	NE	predator	insectivore	nocturnal		
Lissotriton.boscai	49.25	amphibians	water	mediterranean	LC	LC	predator	insectivore	nocturnal		
Pelophylax.perezi	37.59	amphibians	water	general	LC	NE	predator	insectivore	diurnal		
Rana.iberica	49.09	amphibians	water	atlantic	VU	LC	predator	insectivore	nocturnal		
Salamandra.salamandra	39.47	amphibians	forests	atlantic	LC	LC	predator	insectivore	nocturnal		
Triturus.marmoratus	39.39	amphibians	water	mediterranean	LC	LC	predator	insectivore	nocturnal		
Anguis.fragilis	57.14	Reptiles	forests	atlantic	NE	LC	predator	insectivore	diurnal		
Lacerta.schreiberi	50	Reptiles	forests	atlantic	NT	LC	predator	insectivore	diurnal		
Malpolon.monspessulanus	33.33	Reptiles	shrublands	mediterranean	LC	LC	predator	carnivore	diurnal		
Natrix.astreptophora	61.54	Reptiles	woodlands	atlantic	NE	LC	predator	carnivore	diurnal		
Natrix.maura	31.58	Reptiles	water	mediterranean	LC	LC	predator	carnivore	diurnal		
Podarcis.lusitanicus	64.44	Reptiles	rocks	atlantic	NE	NE	predator	insectivore	diurnal		
Psammodromus.algirus	41.3	Reptiles	shrublands	mediterranean	NE	LC	predator	insectivore	diurnal		
Timon.lepidus	53.13	Reptiles	shrublands	mediterranean	NT	LC	predator	insectivore	diurnal		
Vipera.latastei	100	Reptiles	shrublands	mediterranean	VU	VU	predator	carnivore	diurnal		
Zamenis.scalaris	45.83	Reptiles	shrublands	mediterranean	LC	LC	predator	carnivore	diurnal		

Canis.lupus	45.28	mammals	forests	general	LC	EN	predator	carnivore	nocturnal
Capreolus.capreolus	51.11	mammals	woodlands	atlantic	LC	LC	herbivore	herbivore	nocturnal
Cervus.elaphus	48.65	mammals	woodlands	general	NE	LC	herbivore	herbivore	nocturnal
Felis.silvestris	30	mammals	forests	mediterranean	NE	VU	predator	carnivore	nocturnal
Genetta.genetta	52.38	mammals	forests	mediterranean	LC	LC	predator	omnivore	nocturnal
Lepus.granatensis	45.45	mammals	woodlands	mediterranean	LC	LC	herbivore	herbivore	nocturnal
Martes.foina	50	mammals	woodlands	atlantic	LC	LC	predator	omnivore	nocturnal
Martes.martes	38.46	mammals	forests	atlantic	LC	DD	predator	carnivore	nocturnal
Meles.meles	58.33	mammals	woodlands	general	NE	LC	predator	omnivore	nocturnal
Oryctolagus.cuniculus	60	mammals	generalist	mediterranean	NT	NT	herbivore	herbivore	nocturnal
Sciurus.vulgaris	28.57	mammals	forests	general	LC	LC	herbivore	herbivore	diurnal
Sus.scrofa	51.61	mammals	generalist	general	LC	LC	predator	omnivore	nocturnal
Vulpes.vulpes	44.9	mammals	generalist	general	LC	LC	predator	omnivore	nocturnal
Accipiter.nisus	100	Birds	woodlands	atlantic	LC	LC	predator	carnivore	diurnal
Aegithalos.caudatus	56.41	Birds	woodlands	atlantic	LC	LC	predator	insectivore	diurnal
Alauda.arvensis	44.83	Birds	crops	general	LC	LC	predator	omnivore	diurnal
Alectoris.rufa	56.25	Birds	crops	mediterranean	NT	LC	predator	omnivore	diurnal
Anthus.campestris	60	Birds	grasslands	mediterranean	LC	LC	predator	insectivore	diurnal
Anthus.pratensis	55.56	Birds	grasslands	atlantic	LC	LC	predator	insectivore	diurnal
Anthus.trivialis	75	Birds	woodlands	general	LC	NT	predator	insectivore	diurnal
Apus.apus	61.36	Birds	rocks	general	NT	LC	predator	insectivore	diurnal
Aquila.chrysaetos	66.67	Birds	rocks	atlantic	LC	EN	predator	carnivore	diurnal
Buteo.buteo	55.17	Birds	forests	atlantic	LC	LC	predator	carnivore	diurnal
Sylvia.atricapilla	60	Birds	woodlands	general	LC	LC	predator	omnivore	diurnal
Troglodytes.troglodytes	58.06	Birds	forests	general	LC	LC	predator	insectivore	diurnal
Turdus.merula	53.33	Birds	forests	general	LC	LC	predator	insectivore	diurnal

Turdus.philomelos	59.46	Birds	woodlands	general	LC	NT	predator	omnivore	diurnal
Turdus.viscivorus	60	Birds	woodlands	general	LC	LC	predator	omnivore	diurnal
Upupa.epops	67.65	Birds	grasslands	mediterranean	LC	LC	predator	insectivore	diurnal
Caprimulgus.europaeus	41.67	Birds	shrublands	atlantic	LC	VU	predator	insectivore	nocturnal
Carduelis.carduelis	44.44	Birds	woodlands	mediterranean	LC	LC	herbivore	granivore	diurnal
Cecropis.daurica	60	Birds	rocks	mediterranean	LC	LC	predator	insectivore	diurnal
Certhia.brachydactyla	20	Birds	woodlands	atlantic	LC	LC	predator	insectivore	diurnal
Cettia.cetti	60	Birds	wetlands	mediterranean	LC	LC	predator	insectivore	diurnal
Chloris.chloris	85.71	Birds	woodlands	atlantic	LC	LC	herbivore	granivore	diurnal
Ciconia.ciconia	67.74	Birds	wetlands	mediterranean	LC	LC	predator	carnivore	diurnal
Cinclus.cinclus	46.15	Birds	wetlands	atlantic	LC	LC	predator	insectivore	diurnal
Circaetus.gallicus	52.38	Birds	woodlands	mediterranean	LC	NT	predator	carnivore	diurnal
Circus.cyaneus	50	Birds	shrublands	atlantic	LC	CR	predator	carnivore	diurnal
Circus.pygargus	62.79	Birds	crops	atlantic	LC	EN	predator	carnivore	diurnal
Columba.livia	63.64	Birds	urban	mediterranean	LC	DD	predator	omnivore	diurnal
Columba.palumbus	52.94	Birds	forests	mediterranean	LC	LC	herbivore	granivore	diurnal
Corvus.corax	33.33	Birds	shrublands	atlantic	LC	NT	predator	omnivore	diurnal
Corvus.corone	52.78	Birds	urban	atlantic	LC	LC	predator	omnivore	diurnal
Coturnix.coturnix	62.5	Birds	crops	mediterranean	NT	LC	herbivore	granivore	diurnal
Cuculus.canorus	63.64	Birds	woodlands	atlantic	LC	LC	predator	insectivore	diurnal
Curruca.cantillans	62.16	Birds	shrublands	mediterranean	LC	LC	predator	insectivore	diurnal
Curruca.communis	28.57	Birds	shrublands	mediterranean	LC	LC	predator	insectivore	diurnal
Curruca.melanocephala	47.06	Birds	shrublands	mediterranean	LC	LC	predator	insectivore	diurnal
Curruca.undata	65.52	Birds	shrublands	mediterranean	NT	LC	predator	insectivore	diurnal
Cyanistes.caeruleus	60	Birds	woodlands	atlantic	LC	LC	predator	insectivore	diurnal
Delichon.urbicum	51.95	Birds	rocks	general	LC	LC	predator	insectivore	diurnal

Dendrocopos.major	25	Birds	forests	atlantic	LC	LC	predator	omnivore	diurnal
Dryobates.minor	50	Birds	woodlands	atlantic	LC	LC	predator	insectivore	diurnal
Emberiza.calandra	30.77	Birds	grasslands	mediterranean	LC	LC	herbivore	granivore	diurnal
Emberiza.cia	57.35	Birds	rocks	mediterranean	LC	LC	predator	insectivore	diurnal
Emberiza.cirlus	60	Birds	shrublands	mediterranean	LC	LC	predator	omnivore	diurnal
Erithacus.rubecula	46.15	Birds	woodlands	atlantic	LC	LC	predator	omnivore	diurnal
Falco.peregrinus	54.55	Birds	rocks	general	LC	VU	predator	carnivore	diurnal
Falco.tinnunculus	66.67	Birds	forests	atlantic	LC	LC	predator	insectivore	diurnal
Ficedula.hypoleuca	75	Birds	forests	atlantic	LC	NE	predator	insectivore	diurnal
Fringilla.coelebs	64.1	Birds	forests	general	LC	LC	predator	omnivore	diurnal
Galerida.theklae	71.43	Birds	shrublands	mediterranean	LC	LC	predator	insectivore	diurnal
Garrulus.glandarius	62.5	Birds	forests	atlantic	LC	LC	predator	omnivore	diurnal
Gyps.fulvus	60.87	Birds	rocks	mediterranean	LC	NT	predator	carnivore	diurnal
Hieraaetus.pennatus	71.43	Birds	woodlands	mediterranean	LC	NT	predator	carnivore	diurnal
Hippolais.polyglotta	51.85	Birds	shrublands	mediterranean	LC	LC	predator	insectivore	diurnal
Hirundo.rustica	54.55	Birds	rocks	general	LC	LC	predator	insectivore	diurnal
Jynx.torquilla	61.97	Birds	woodlands	general	LC	DD	predator	insectivore	diurnal
Lanius.collurio	66.67	Birds	shrublands	mediterranean	LC	NT	predator	carnivore	diurnal
Lanius.meridionalis	50	Birds	woodlands	mediterranean	VU	LC	predator	carnivore	diurnal
Lanius.senator	30.77	Birds	shrublands	mediterranean	NT	NT	predator	insectivore	diurnal
Linaria.cannabina	33.33	Birds	shrublands	mediterranean	LC	LC	herbivore	granivore	diurnal
Lophophanes.cristatus	56.25	Birds	forests	atlantic	LC	LC	predator	insectivore	diurnal
Lullula.arborea	44.44	Birds	shrublands	mediterranean	LC	LC	herbivore	granivore	diurnal
Luscinia.megarhynchos	58.33	Birds	shrublands	mediterranean	LC	LC	predator	insectivore	diurnal
Merops.apiaster	62.5	Birds	woodlands	mediterranean	LC	LC	predator	insectivore	diurnal
Milvus.migrans	60.87	Birds	generalist	general	LC	LC	predator	carnivore	diurnal

Milvus.milvus	58.33	Birds	woodlands	mediterranean	LC	LC	predator	carnivore	diurnal
Motacilla.alba	60	Birds	wetlands	general	LC	LC	predator	carnivore	diurnal
Motacilla.cinerea	58.62	Birds	wetlands	general	LC	LC	predator	insectivore	diurnal
Oenanthe.oenanthe	33.33	Birds	rocks	general	LC	LC	predator	insectivore	diurnal
Oriolus.oriolus	71.43	Birds	woodlands	general	LC	LC	predator	omnivore	diurnal
Parus.major	60.87	Birds	forests	general	LC	LC	predator	omnivore	diurnal
Passer.domesticus	66.67	Birds	urban	general	LC	LC	herbivore	insectivore	diurnal
Passer.montanus	66.67	Birds	crops	atlantic	LC	LC	herbivore	insectivore	diurnal
Periparus.ater	44.44	Birds	forests	atlantic	LC	LC	predator	omnivore	diurnal
Pernis.apivorus	57.45	Birds	forests	atlantic	LC	VU	predator	insectivore	diurnal
Petronia.petronia	50	Birds	rocks	mediterranean	LC	LC	herbivore	granivore	diurnal
Phoenicurus.ochruros	52.38	Birds	rocks	general	LC	LC	predator	omnivore	diurnal
Phoenicurus.phoenicurus	52.38	Birds	woodlands	general	LC	LC	herbivore	insectivore	diurnal
Phylloscopus.bonelli	59.46	Birds	woodlands	mediterranean	LC	LC	predator	insectivore	diurnal
Phylloscopus.collybita	72.73	Birds	woodlands	general	LC	LC	predator	insectivore	diurnal
Phylloscopus.ibericus	61.54	Birds	shrublands	mediterranean	LC	LC	predator	insectivore	diurnal
Pica.pica	42.11	Birds	woodlands	atlantic	LC	LC	predator	omnivore	diurnal
Picus.sharpei	56.16	Birds	woodlands	mediterranean	LC	NE	predator	insectivore	diurnal
Picus.viridis	55.56	Birds	forests	atlantic	LC	LC	predator	insectivore	diurnal
Prunella.modularis	59.09	Birds	shrublands	atlantic	LC	LC	predator	insectivore	diurnal
Ptyonoprogne.rupestris	57.14	Birds	rocks	mediterranean	LC	LC	predator	insectivore	diurnal
Pyrrhula.pyrrhula	65.71	Birds	woodlands	atlantic	LC	LC	herbivore	insectivore	diurnal
Regulus.ignicapilla	62.5	Birds	forests	atlantic	LC	LC	predator	insectivore	diurnal
Saxicola.rubicola	55.17	Birds	shrublands	mediterranean	NE	NE	predator	insectivore	diurnal
Serinus.serinus	58.33	Birds	woodlands	mediterranean	LC	LC	herbivore	granivore	diurnal
Sitta.europaea	61.05	Birds	forests	atlantic	LC	LC	predator	insectivore	diurnal

Streptopelia.decaocto	52.81	Birds	shrublands	mediterranean	LC	LC	herbivore	granivore	diurnal		
Streptopelia.turtur	46.43	Birds	woodlands	mediterranean	VU	LC	herbivore	granivore	diurnal		
Strix.aluco	36.84	Birds	forests	atlantic	LC	LC	predator	carnivore	nocturnal		
Sturnus.unicolor	50	Birds	woodlands	mediterranean	LC	LC	predator	omnivore	diurnal		
Achillea.millefolium	50	Plants	grasslands	atlantic	LC	NE				C3	
Adenocarpus.complicatus	33.33	Plants	shrublands	atlantic	LC	NE				C3	
Agrimonia.eupatoria	50	Plants	woodlands	general	LC	NE				C3	zoophily
Agrostis.castellana	57.14	Plants	generalist	general	NE	NE				C3	
Agrostis.curtisii	50	Plants	woodlands	atlantic	NE	NE				C3	
Agrostis.truncatula	53.33	Plants	rocks	atlantic	NE	NE				C3	
Aira.caryophyllea	62.5	Plants	grasslands	general	NE	NE				C3	
Alliaria.petiolata	28.57	Plants	woodlands	atlantic	NE	NE				C3	zoophily
Alnus.glutinosa	37.5	Plants	woodlands	atlantic	LC	NE				C3	anemophily
Alyssum.serpyllifolium	33.33	Plants	rocks	mediterranean	DD	LC				C3	
Euphorbia.amygdaloides	50	Plants	woodlands	atlantic	NE	NE				C3	
Arctium.minus	55.56	Plants	grasslands	general	NE	NE				C3	zoophily
Arnoseris.minima	44.44	Plants	grasslands	atlantic	NE	NE				C3	
Fragaria.vesca	28.57	Plants	woodlands	atlantic	LC	NE				C3	
Frangula.alnus	60	Plants	woodlands	general	LC	NE				C3	
Fraxinus.angustifolia	100	Plants	woodlands	general	LC	NE				C3	anemophily
Galium.aparine	60	Plants	urban	general	LC	NE				C3	zoophily
Galium.broterianum	33.33	Plants	woodlands	atlantic	NE	NE				C3	
Galium.lucidum	50	Plants	shrublands	mediterranean	NE	NE				C3	
Arrhenatherum.elatius	50	Plants	grasslands	atlantic	LC	NE				C3	
Galium.verum	38.46	Plants	woodlands	mediterranean	LC	NE				C3	
Genista.falcata	20	Plants	shrublands	atlantic	LC	NE				C3	

Genista.florida	60	Plants	woodlands	atlantic	NE	NE	C3	
Avena.barbata	50	Plants	grasslands	general	LC	NE	C3	
Bromus.hordeaceus	83.33	Plants	grasslands	general	NE	NE	C3	
Geum.sylvaticum	50	Plants	woodlands	mediterranean	NE	NE	C3	
Geum.urbanum	60	Plants	woodlands	atlantic	LC	NE	C3	zoophily
Halimium.lasianthum	60	Plants	shrublands	atlantic	NE	NE	C3	
Halimium.umbellatum	66.67	Plants	shrublands	atlantic	DD	LC	C3	
Helichrysum.stoechas	16.67	Plants	shrublands	mediterranean	LC	NE	C3	
Helleborus.foetidus	40	Plants	shrublands	mediterranean	LC	NE	C3	
Campanula.lusitanica	20	Plants	grasslands	general	NE	NE	C3	
Hispidella.hispanica	50	Plants	shrublands	atlantic	NE	NE	C3	
Holcus.lanatus	16.67	Plants	missing	missing	NE	NE	C3	
Holcus.mollis	28.57	Plants	missing	missing	NE	NE	C3	
Hordeum.murinum	40	Plants	crops	mediterranean	LC	NE	C3	
Carduus.tenuiflorus	42.86	Plants	grasslands	general	NE	NE	C3	
Hypericum.linariifolium	100	Plants	shrublands	atlantic	NE	NE	C3	
Carum.verticillatum	54.55	Plants	grasslands	general	LC	NE	C3	
Centaurea.nigra	100	Plants	grasslands	atlantic	NE	NE	C3	zoophily
Chamaemelum.nobile	62.5	Plants	grasslands	atlantic	LC	NE	C3	
Jasione.montana	33.33	Plants	generalist	general	NE	NE	C3	
Juncus.effusus	63.64	Plants	wetlands	atlantic	LC	NE	C3	anemophily
Lactuca.serriola	44.44	Plants	urban	atlantic	LC	NE	C3	
Lactuca.viminea	66.67	Plants	rocks	mediterranean	LC	NE	C3	
Cirsium.vulgare	77.78	Plants	grasslands	atlantic	NE	NE	C3	
Lapsana.communis	52.94	Plants	woodlands	atlantic	NE	NE	C3	
Lathyrus.niger	66.67	Plants	woodlands	atlantic	NE	NE	C3	

Lavandula.pedunculata	100	Plants	shrublands	mediterranean	NE	NE	C3	
Leontodon.saxatilis	25	Plants	missing	missing	NE	NE	C3	
Lepidium.heterophyllum	66.67	Plants	rocks	general	LC	NE	C3	
Linaria.intricata	43.75	Plants	shrublands	mediterranean	NE	DD	C3	
Crepis.lampsanoides	66.67	Plants	grasslands	atlantic	NE	NE	C3	
Logfia.minima	33.33	Plants	missing	missing	NE	NE	C3	
Cruciata.glabra	66.67	Plants	grasslands	atlantic	NE	NE	C3	
Lonicera.periclymenum	100	Plants	shrublands	general	NE	NE	C3	zoophily
Lotus.pedunculatus	55.56	Plants	wetlands	atlantic	LC	NE	C3	
Lythrum.salicaria	57.14	Plants	wetlands	general	LC	NE	C3	zoophily
Cruciata.laevipes	53.85	Plants	grasslands	atlantic	NE	NE	C3	zoophily
Cynosurus.cristatus	42.86	Plants	grasslands	atlantic	NE	NE	C3	
Dactylis.glomerata	46.15	Plants	grasslands	general	NE	NE	C3	
Micropyrum.tenellum	45.45	Plants	missing	missing	NE	NE	C3	
Dianthus.laricifolius	66.67	Plants	grasslands	atlantic	LC	NE	C3	
Echium.rosulatum	50	Plants	grasslands	atlantic	NE	NT	C3	
Eryngium.campestre	33.33	Plants	grasslands	mediterranean	NE	NE	C3	anemophily
Oenanthe.crocata	50	Plants	wetlands	mediterranean	LC	NE	C3	
Festuca.elegans	55.56	Plants	grasslands	mediterranean	LC	LC	C3	
Osmunda.regalis	75	Plants	wetlands	atlantic	LC	NE	C3	
Paeonia.broteri	9.09	Plants	woodlands	general	NE	LC	C3	
Pentaglottis.sempervirens	33.33	Plants	woodlands	atlantic	NE	NE	C3	zoophily
Petrorhagia.nanteuilii	50	Plants	shrublands	mediterranean	NE	NE	C3	
Physospermum.cornubiense	62.5	Plants	shrublands	atlantic	NE	NE	C3	
Picris.hieracioides	66.67	Plants	shrublands	atlantic	NE	NE	C3	zoophily
Filipendula.ulmaria	53.85	Plants	grasslands	atlantic	LC	NE	C3	zoophily
Pimpinella.villosa	57.14	Plants	shrublands	mediterranean	NE	NE	C3	

Galium.papillosum	28.57	Plants	grasslands	atlantic	NE	NE		C3	
Geranium.lucidum	100	Plants	grasslands	atlantic	NE	NE		C3	
Geranium.pyrenaicum	60	Plants	grasslands	atlantic	NE	NE		C3	
Heracleum.sphondylium	22.22	Plants	grasslands	atlantic	NE	NE		C3	
Poa.bulbosa	60	Plants	missing	missing	NE	NE		C3	
Poa.trivialis	37.5	Plants	missing	missing	NE	NE		C3	
Hymenocarpos.lotoides	50	Plants	grasslands	mediterranean	NE	NE		C3	
Hypericum.perforatum	66.67	Plants	grasslands	atlantic	LC	NE		C3	zoophily
Hypericum.undulatum	63.64	Plants	grasslands	atlantic	NE	NE		C3	
Primula.acaulis	50	Plants	woodlands	atlantic	NE	NE		C3	
Hypochaeris.radicata	47.06	Plants	grasslands	atlantic	NE	NE		C3	
Prunus.avium	25	Plants	woodlands	atlantic	LC	NE		C3	
Pteridium.aquilinum	33.33	Plants	woodlands	atlantic	LC	NE		C3	
Pterospartum.tridentatum	60	Plants	shrublands	atlantic	NE	NE		C3	
Quercus.pyrenaica	50	Plants	woodlands	atlantic	LC	NE		C3	
Quercus.rotundifolia	50	Plants	woodlands	mediterranean	LC	NE		C3	
Lamium.maculatum	58.33	Plants	grasslands	atlantic	NE	NE		C3	
Rosa.micrantha	45.45	Plants	woodlands	atlantic	NE	NE		C3	
Rubus.brigantinus	37.5	Plants	woodlands	atlantic	NE	NE		C3	
Rubus.ulmifolius	37.5	Plants	urban	general	NE	NE		C3	zoophily
Rubus.vagabundus	28.57	Plants	woodlands	atlantic	NE	NE		C3	
Rumex.acetosa	100	Plants	woodlands	atlantic	NE	NE		C3	
Linum.bienne	33.33	Plants	grasslands	general	NE	NE		C3	zoophily
Ruscus.aculeatus	71.43	Plants	woodlands	general	LC	LC		C3	
Salix.atrocinerea	50	Plants	wetlands	atlantic	LC	NE		C3	
Lolium.perenne	66.67	Plants	grasslands	atlantic	LC	NE		C3	anemophily

Sambucus.nigra	66.67	Plants	woodlands	atlantic	LC	NE		C3	
Anagallis.arvensis	33.33	Plants	crops	general	LC	NE		C3	zoophily
Anarrhinum.bellidifolium	44.44	Plants	shrublands	general	NE	NE		C3	
Andryala.integrifolia	40	Plants	crops	general	NE	NE		C3	
Anthemis.arvensis	71.43	Plants	crops	atlantic	NE	NE		C3	zoophily
Anthoxanthum.odoratum	28.57	Plants	woodlands	atlantic	NE	NE		C3	
Anthoxanthum.ovatum	33.33	Plants	missing	missing	NE	NE		C3	
Anthyllis.vulneraria	42.86	Plants	rocks	mediterranean	DD	LC		C3	zoophily
Aquilegia.vulgaris	83.33	Plants	woodlands	atlantic	NE	NE		C3	zoophily
Arbutus.unedo	50	Plants	shrublands	mediterranean	LC	NE		C3	zoophily
Malva.sylvestris	50	Plants	grasslands	general	LC	NE		C3	
Malva.tournefortiana	14.29	Plants	grasslands	atlantic	NE	NE		C3	
Santolina.semidentata	14.29	Plants	rocks	mediterranean	LC	LC		C3	
Saponaria.officinalis	40	Plants	wetlands	atlantic	LC	NE		C3	zoophily
Saxifraga.fragosoi	47.83	Plants	rocks	atlantic	NE	NE		C3	
Scrophularia.scorodonia	16.67	Plants	woodlands	atlantic	NE	NE		C3	
Sedum.arenarium	46.67	Plants	rocks	atlantic	NE	NE		CAM	
Sedum.brevifolium	50	Plants	rocks	general	NE	NE		CAM	
Sedum.forsterianum	57.14	Plants	woodlands	general	NE	NE		CAM	
Sedum.hirsutum	28.57	Plants	rocks	atlantic	NE	NE		CAM	
Mentha.suaveolens	50	Plants	grasslands	general	LC	NE		C3	
Senecio.sylvaticus	37.5	Plants	woodlands	atlantic	NE	NE		C3	
Sonchus.asper	40	Plants	crops	general	NE	NE		C3	
Stachys.sylvatica	40	Plants	woodlands	atlantic	NE	LC		C3	
Myosotis.discolor	50	Plants	grasslands	general	NE	NE		C3	
Stellaria.holostea	33.33	Plants	shrublands	atlantic	NE	NE		C3	
Tamus.communis	33.33	Plants	woodlands	general	LC	NE		C3	

Teucrium.scorodonia	100	Plants	woodlands	atlantic	LC	NE	C3	
Thalictrum.speciosissimum	25	Plants	wetlands	atlantic	NE	NE	C3	
Thymus.mastichina	28.57	Plants	crops	mediterranean	LC	NE	C3	
Myosotis.ramosissima	100	Plants	grasslands	general	NE	NE	C3	
Tordylium.maximum	55.56	Plants	crops	atlantic	NE	NE	C3	
Torilis.arvensis	72.73	Plants	crops	mediterranean	NE	NE	C3	
Nardus.stricta	75	Plants	grasslands	atlantic	NE	NE	C3	
Omphalodes.nitida	75	Plants	grasslands	atlantic	NE	NE	C3	
Pilosella.pseudopilosella	56.25	Plants	grasslands	mediterranean	NE	NE	C3	
Plantago.coronopus	55.56	Plants	grasslands	general	NE	NE	C3	anemophily
Plantago.holosteum	60.87	Plants	grasslands	atlantic	NE	NE	C3	
Plantago.lanceolata	20	Plants	grasslands	atlantic	LC	NE	C3	anemophily
Plantago.major	75	Plants	grasslands	atlantic	LC	NE	C3	anemophily
Ulmus.minor	57.14	Plants	woodlands	atlantic	DD	NE	C3	
Urtica.dioica	38.46	Plants	woodlands	atlantic	LC	NE	C3	
Vincetoxicum.nigrum	55.56	Plants	woodlands	atlantic	NE	NE	C3	anemophily
Viola.riviniana	61.54	Plants	woodlands	atlantic	NE	NE	C3	
Vulpia.muralis	50	Plants	crops	mediterranean	NE	NE	C3	
Arenaria.montana	50	Plants	woodlands	atlantic	NE	NE	C3	zoophily
Aristolochia.paucinervis	33.33	Plants	crops	mediterranean	NE	NE	C3	
Polygala.vulgaris	100	Plants	grasslands	atlantic	NE	NE	C3	
Arrhenatherum.album	16.67	Plants	missing	missing	NE	NE	C3	
Potentilla.erecta	72.73	Plants	grasslands	atlantic	LC	NE	C3	zoophily
Asplenium.trichomanes	33.33	Plants	rocks	atlantic	LC	NE	C3	
Athyrium.filix-femina	50	Plants	woodlands	atlantic	LC	NE	C3	
Potentilla.sterilis	60	Plants	grasslands	atlantic	NE	NE	C3	

Brachypodium.rupestre	33.33	Plants	woodlands	atlantic	NE	NE		C3	
Brachypodium.sylvaticum	33.33	Plants	woodlands	atlantic	NE	NE		C3	
Briza.maxima	66.67	Plants	crops	general	NE	NE		C3	
Bromus.diandrus	33.33	Plants	missing	missing	NE	NE		C3	
Prunella.vulgaris	100	Plants	grasslands	atlantic	LC	NE		C3	
Bromus.madritensis	40	Plants	crops	general	NE	NE		C3	
Bromus.sterilis	80	Plants	missing	missing	NE	NE		C3	
Bromus.tectorum	63.64	Plants	crops	atlantic	NE	NE		C3	
Bryonia.dioica	37.5	Plants	woodlands	general	NE	NE		C3	
Calluna.vulgaris	33.33	Plants	shrublands	general	LC	NE		C3	zoophily
Ranunculus.repens	50	Plants	grasslands	atlantic	LC	NE		C3	
Campanula.rapunculus	66.67	Plants	woodlands	mediterranean	NE	NE		C3	
Carduus.carpetanus	26.67	Plants	crops	atlantic	NE	NE		C3	
Rumex.acetosella	42.86	Plants	grasslands	atlantic	LC	NE		C3	
Carex.elata	80	Plants	wetlands	atlantic	LC	NE		C3	anemophily
Carex.leporina	50	Plants	wetlands	atlantic	NE	NE		C3	
Carex.muricata	100	Plants	woodlands	atlantic	NE	NE		C3	
Salix.salviifolia	100	Plants	grasslands	general	LC	LC		C3	
Castanea.sativa	83.33	Plants	woodlands	atlantic	LC	NE		C3	zoophily
Sanguisorba.minor	50	Plants	grasslands	atlantic	NE	NE		C3	zoophily
Cerastium.fontanum	47.06	Plants	forests	atlantic	NE	NE		C3	
Chaerophyllum.temulum	20	Plants	woodlands	atlantic	NE	NE		C3	
Senecio.jacobaea	33.33	Plants	grasslands	general	DD	NE		C3	
Chelidonium.majus	50	Plants	urban	atlantic	LC	NE		C3	
Chondrilla.juncea	55.56	Plants	crops	mediterranean	NE	NE		C3	
Cirsium.arvense	33.33	Plants	crops	mediterranean	NE	NE		C3	
Cirsium.palustre	20	Plants	wetlands	atlantic	NE	NE		C3	

Stellaria.graminea	47.62	Plants	grasslands	atlantic	NE	NE	C3	
Cistus.ladanifer	100	Plants	shrublands	mediterranean	NE	NE	C3	
Cistus.psilosepalus.//.inflatus	33.33	Plants	shrublands	atlantic	LC	LC	C3	
Clinopodium.vulgare	36.36	Plants	woodlands	general	NE	NE	C3	zoophily
Convolvulus.arvensis	40	Plants	crops	general	NE	NE	C3	
Corylus.avellana	66.67	Plants	woodlands	atlantic	LC	NE	C3	
Crataegus.monogyna	57.14	Plants	shrublands	general	LC	NE	C3	
Crepis.capillaris	33.33	Plants	missing	missing	NE	NE	C3	
Thymus.pulegioides	33.33	Plants	grasslands	atlantic	NE	NE	C3	
Crepis.vesicaria	33.33	Plants	crops	mediterranean	NE	NE	C3	
Trifolium.angustifolium	38.46	Plants	grasslands	general	LC	NE	C3	
Trifolium.arvense	33.33	Plants	grasslands	general	LC	NE	C3	
Trifolium.campestre	25	Plants	grasslands	general	NE	LC	C3	
Cucubalus.baccifer	58.33	Plants	woodlands	atlantic	NE	NE	C3	
Cynosurus.echinatus	42.86	Plants	crops	general	NE	NE	C3	
Cytisus.multiflorus	100	Plants	shrublands	atlantic	LC	NE	C3	
Cytisus.scoparius	40	Plants	shrublands	atlantic	NE	NE	C3	
Trifolium.dubium	37.5	Plants	grasslands	general	NE	NE	C3	
Daphne.gnidium	30	Plants	woodlands	general	NE	NE	C3	
Daucus.carota	50	Plants	generalist	general	LC	NT	C3	
Digitalis.purpurea	14.29	Plants	rocks	atlantic	LC	LC	C3	zoophily
Draba.muralis	100	Plants	rocks	atlantic	NE	NE	C3	
Trifolium.pratense	60	Plants	grasslands	general	LC	NE	C3	
Erica.arborea	75	Plants	shrublands	atlantic	LC	NE	C3	
Erica.australis	30	Plants	shrublands	atlantic	NE	NE	C3	
Erica.cinerea	50	Plants	shrublands	atlantic	LC	NE	C3	
Erica.tetralix	42.86	Plants	shrublands	atlantic	NE	NE	C3	

Erica.umbellata	60	Plants	shrublands	atlantic	NE	NE	C3	
Erodium.cicutarium	70	Plants	generalist	general	NE	NE	C3	zoophily
Trifolium.repens	42.86	Plants	grasslands	general	LC	NE	C3	
Erysimum.linifolium	38.46	Plants	rocks	atlantic	NE	NE	C3	
Tuberaria.guttata	25	Plants	grasslands	general	NE	NE	C3	
