# Supplementary material for: Time-Series Niche Modelling Reveals Declining Tendencies of Habitat Suitability and Ecological Functions in a Mountainous Protected Area
Source: Environ Manage. 2026 Feb 18;76(3):101. doi: 10.1007/s00267-026-02393-5 (PMC12916538; doi:10.1007/s00267-026-02393-5)
Supplement: Supplementary file 2 — ESM_2 [file 267_2026_2393_MOESM2_ESM.pdf]

Online Resource 2. Summary of the candidate Moderate Resolution Imaging Spectroradiometer (MODIS) satellite-derived variables computed in Google Earth Engine considered in this study, indicating product name, spatial and temporal resolution, and rationale. Variables selected for model fitting are highlighted in bold.

Product name	Code	MODIS product	Pixel size	Rationale
Albebo NIR-White	ALB-NIRW	MCD43B3.006	500 m	Surrogate for surface properties
Albebo NIR-Black	ALB-NIRB	MCD43B3.006	500 m	Surrogate for surface properties
Albebo VIS-White	ALB-VISW	MCD43B3.006	500 m	Surrogate for surface properties
Albebo VIS-Black	ALB-VISB	MCD43B3.006	500 m	Surrogate for surface properties
**Enhanced Vegetation Index**	EVI	MOD13Q1.061	250 m	Proxy for the vegetation cover quality, productivity, and status
Evapotranspiration	ET	MOD16A2.006	500 m	Proxy for water balance between the vegetation cover and hydrological cycle
Fraction of absorbed photosynthetically active radiation	FPAR	MCD15A3H.061	500 m	Proxy for the vegetation cover quality, productivity, and status
Gross Primary Productivity	GPP	MOD17A2H.006	500 m	Proxy for carbon, water cycle processes, and biogeochemistry of vegetation
Leaf Area Infex	LAI	MCD15A3H.061	500 m	Proxy for the vegetation cover quality, productivity, and status
**Land Surface Temperature (Day)**	LST-Day	MOD11A2.061	1 km	Proxy of the energy balance on the earth's surface
**Land Surface Temperature (Night)**	LST-Night	MOD11A2.061	1 km	Proxy of the energy balance on the earth's surface
Normalized Difference Vegetation Index	NDVI	MOD13Q1.061	250 m	Proxy for the vegetation cover quality, productivity, and status.
**Surface reflectance (620-670nm)**	SR-Band1	MOD09Q1.061	250 m	Proxy for changes detection and monitoring on the Earth's surface
Surface reflectance (841-876nm)	SR-Band2	MOD09Q1.061	250 m	Proxy for changes detection and monitoring on the Earth's surface
**Area Annually Burned**	AAB	MCD64A1 (derived)	500 m	Proxy for identifying burned areas
Fire Recurrence	FR	MCD64A1 (derived)	500 m	Proxy for identify recurrences on burned areas
Normalized Burn Ratio	NBR	MOD09GA.061 (derived)	500 m	Proxy for index the severity of burned areas
Normalized Difference Water Index	NDWI	MOD09GA.061 (derived)	500 m	Proxy for water body detection Proxy for human disturbances
**Time-Since Fire**	TSF	MCD64A1.061 (derived)	500 m	Proxy for changes caused by fire
