# Supplementary material for: Time-Series Niche Modelling Reveals Declining Tendencies of Habitat Suitability and Ecological Functions in a Mountainous Protected Area
Source: Environ Manage. 2026 Feb 18;76(3):101. doi: 10.1007/s00267-026-02393-5 (PMC12916538; doi:10.1007/s00267-026-02393-5)
Supplement: Supplementary file 3 — ESM_3 [file 267_2026_2393_MOESM3_ESM.pdf]

Online Resource 3 Analysis of variance (ANOVA) and post hoc Tukey's Honest Significant Difference (HSD) tests to compare the proportion of negative habitat suitability trends among taxonomic groups, functional groups, and conservation status categories.

ANOVA for taxonomic groups, functional traits and IUCN categories:

Comparison	Anova	Df	Sum Sq	Mean Sq	F value	Pr(>F)
Taxonomic group	model	4	2875	718.7	2.171	0.072
	Residuals	336	111253	331.1		

Comparison	group	Anova	Df	Sum Sq	Mean Sq	F value	Pr(>F)
Diet type	Global	model	4	600	150.0	0.895	0.469
		Residuals	123	20610	167.6		
	Reptiles	model	1	4	3.9	0.009	0.926
		Residuals	8	3462	432.8		
	Birds	model	3	853	284.3	1.944	0.128
		Residuals	91	13305	146.2		
	Mammals	model	2	343.7	171.87	2.32	0.149
		Residuals	10	740.8	74.08		
Habitat type	Global	model	12	1860	155	0.463	0.935
		Residuals	316	105851	335		
	Mammals	model	3	521.2	173.7	2.775	0.103
		Residuals	9	563.4	62.6		
	Amphibians	model	4	51.3	12.82	0.168	0.946
		Residuals	5	380.8	76.16		
	Birds	model	9	1022	113.6	0.735	0.676
		Residuals	85	13136	154.5		
	Reptiles	model	4	671.2	167.8	0.3	0.867
		Residuals	5	2795.0	559.0		
	Plants	model	9	2719	302.2	0.692	0.715
		Residuals	191	83354	436.4		
Climatic affinity	Global	model	3	463	154.2	0.467	0.705
		Residuals	325	107249	330.0		
	Plants	model	2	710	355.2	0.824	0.44
		Residuals	198	85363	431.1		
	Mammals	model	2	1.3	0.65	0.006	0.994
		Residuals	10	1083.2	108.32		
	Birds	model	2	187	93.63	0.617	0.542
		Residuals	92	13971	151.85		
	Amphibians	model	3	48.4	16.12	0.252	0.857
		Residuals	6	383.7	63.95		
	Reptiles	model	1	132	132.1	0.317	0.589

		Residuals	8	3334	416.8			
Feeding type	Global	model	1	74	73.68	0.439	0.509	
		Residuals	126	21137	167.75			
	Birds	model	1	69	69.35	0.458	0.5	
		Residuals	93	14088	151.49			
	Mammals	model	1	0.5	0.46	0.005	0.947	
		Residuals	11	1084.1	98.55			
Activity type	Global	model	3	1849	616.4	3.948	**0.00995**	**
		Residuals	124	19361	156.1			
	Mammals	model	2	359.0	179.52	2.474	0.134	
		Residuals	10	725.5	72.55			
	Amphibians	model	2	61.5	30.75	0.581	0.584	
		Residuals	7	370.5	52.94			
	Reptiles	model	1	125	125.1	0.3	0.599	
		Residuals	8	3341	417.6			
	Birds	model	3	597	199	1.336	0.268	
		Residuals	91	13561	149			
Photosynthesis type	Plants	model	1	116	116.3	0.267	0.606	
		Residuals	211	91996	436.0			
Reproduction type	Plants	model	1	118	118.1	0.253	0.618	
		Residuals	37	17276	466.9			
IUCN (European)	Global	model	6	1763	293.8	0.873	0.515	
		Residuals	334	112365	336.4			
	Birds	model	3	137	45.77	0.297	0.827	
		Residuals	91	14020	154.07			
	Mammals	model	2	197.2	98.59	1.111	0.367	
		Residuals	10	887.4	88.74			
	Reptiles	model	3	3021	1007.1	13.58	**0.0044**	*
		Residuals	6	445	74.2			
	Amphibians	model	2	22.8	11.40	0.195	0.827	
		Residuals	7	409.2	58.46			
	Plants	model	4	728	182.0	0.414	0.798	
		Residuals	208	91385	439.3			
IUCN (Regional)	Global	model	7	1471	210.2	0.621	0.738	
		Residuals	333	112657	338.3			
	Plants	model	4	1287	321.9	0.737	0.568	
		Residuals	208	90825	436.7			
	Birds	model	6	461	76.88	0.494	0.811	
		Residuals	88	13697	155.64			
	Mammals	model	4	538.0	134.50	1.969	0.192	
		Residuals	8	546.5	68.32			
	Reptiles	model	2	2647.4	1324	11.32	**0.00641**	*
		Residuals	7	818.7	117			
	Amphibians	model	2	140.2	70.08	1.681	0.253	
		Residuals	7	291.9	41.70			

Tukey multiple comparisons of means:

a) IUCN (European): Reptiles

Levels	diff	lwr	upr	p	
NE-LC	19.19167	-3.576838	41.96017	0.0949214	
NT-LC	14.65167	-12.561902	41.86524	0.3330778	
VU-LC	63.08667	28.663923	97.50941	**0.0029269**	*
NT-NE	-4.54000	-30.357058	21.27706	0.9256415	
VU-NE	43.89500	10.565322	77.22468	**0.0151184**	*
VU-NT	48.43500	11.924167	84.94583	**0.0146141**	*

b) IUCN (Regional): Reptiles

Levels	diff	lwr	upr	p	
NE-LC	17.70875	-16.073820	51.49132	0.3291273	
VU-LC	53.26875	19.486180	87.05132	**0.0057986**	*
VU-NE	35.56000	-9.483427	80.60343	0.1171963	
