# Supplementary material for: Time-Series Niche Modelling Reveals Declining Tendencies of Habitat Suitability and Ecological Functions in a Mountainous Protected Area
Source: Environ Manage. 2026 Feb 18;76(3):101. doi: 10.1007/s00267-026-02393-5 (PMC12916538; doi:10.1007/s00267-026-02393-5)

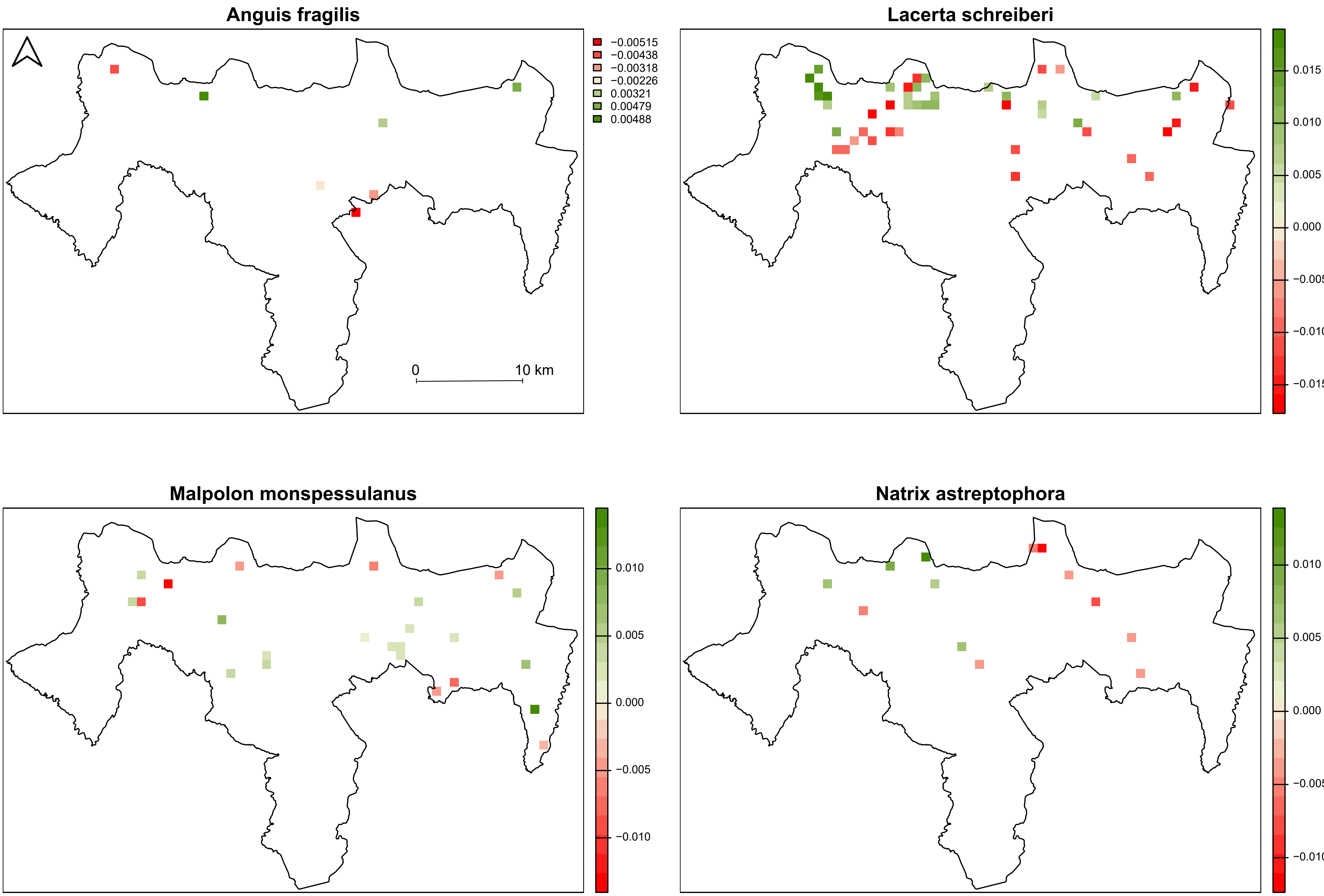
Anguis fragilis
−0.00515
−0.00438
−0.00318
−0.00226
0.00321
0.00479
0.00488
0
10 km
Lacerta schreiberi
0.015
0.010
0.005
0.000
Malpolon monspessulanus
0.010
0.005
0.000
−0.005
−0.010
Natrix astreptophora
0.010
0.005
0.000

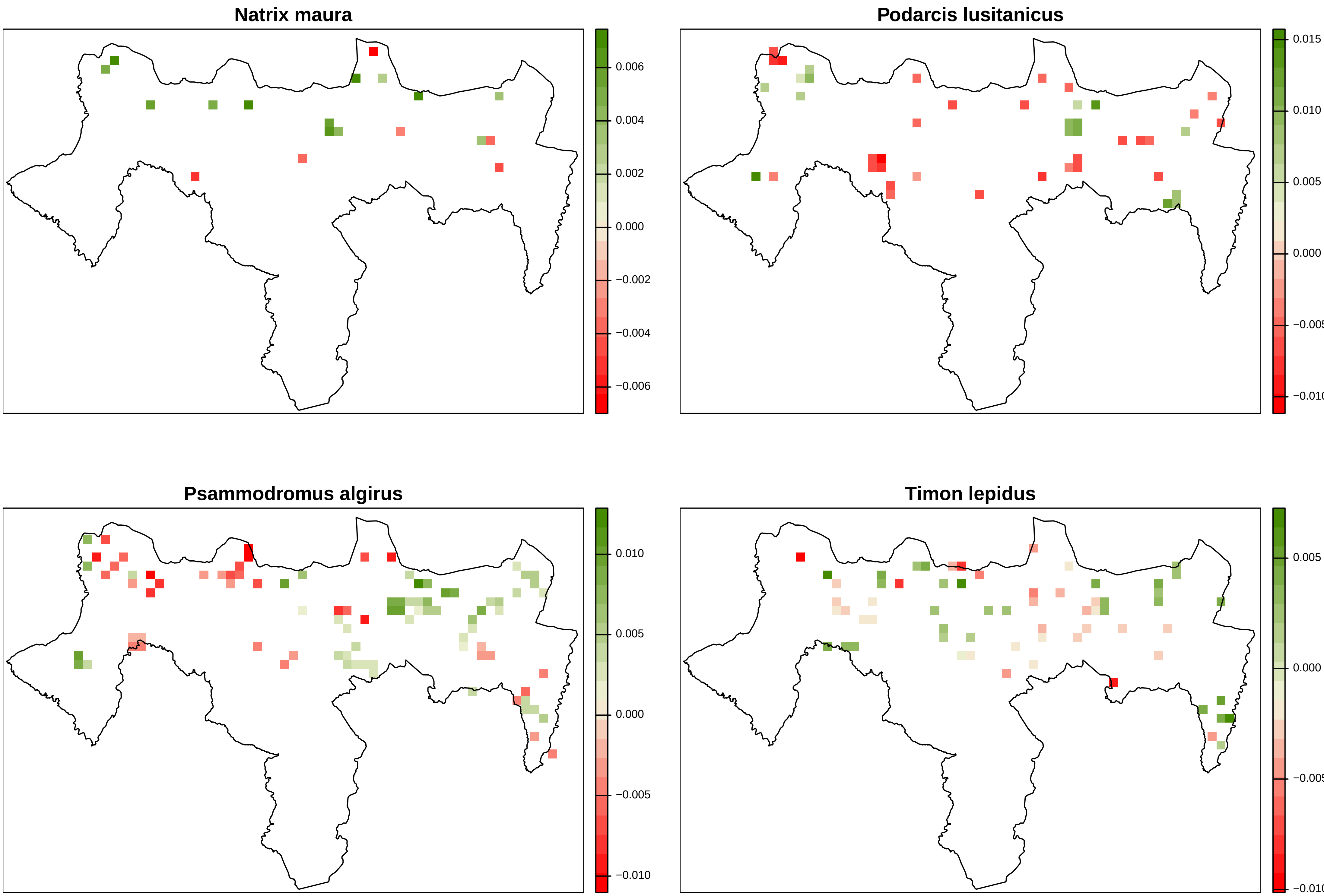
Natrix maura
0.006
0.004
0.002
0.000
−0.002
−0.004
−0.006
Podarcis lusitanicus
0.015
0.010
0.005
0.000
Psammodromus algirus
0.010
0.005
0.000
−0.005
−0.010
Timon lepidus
0.005
0.000

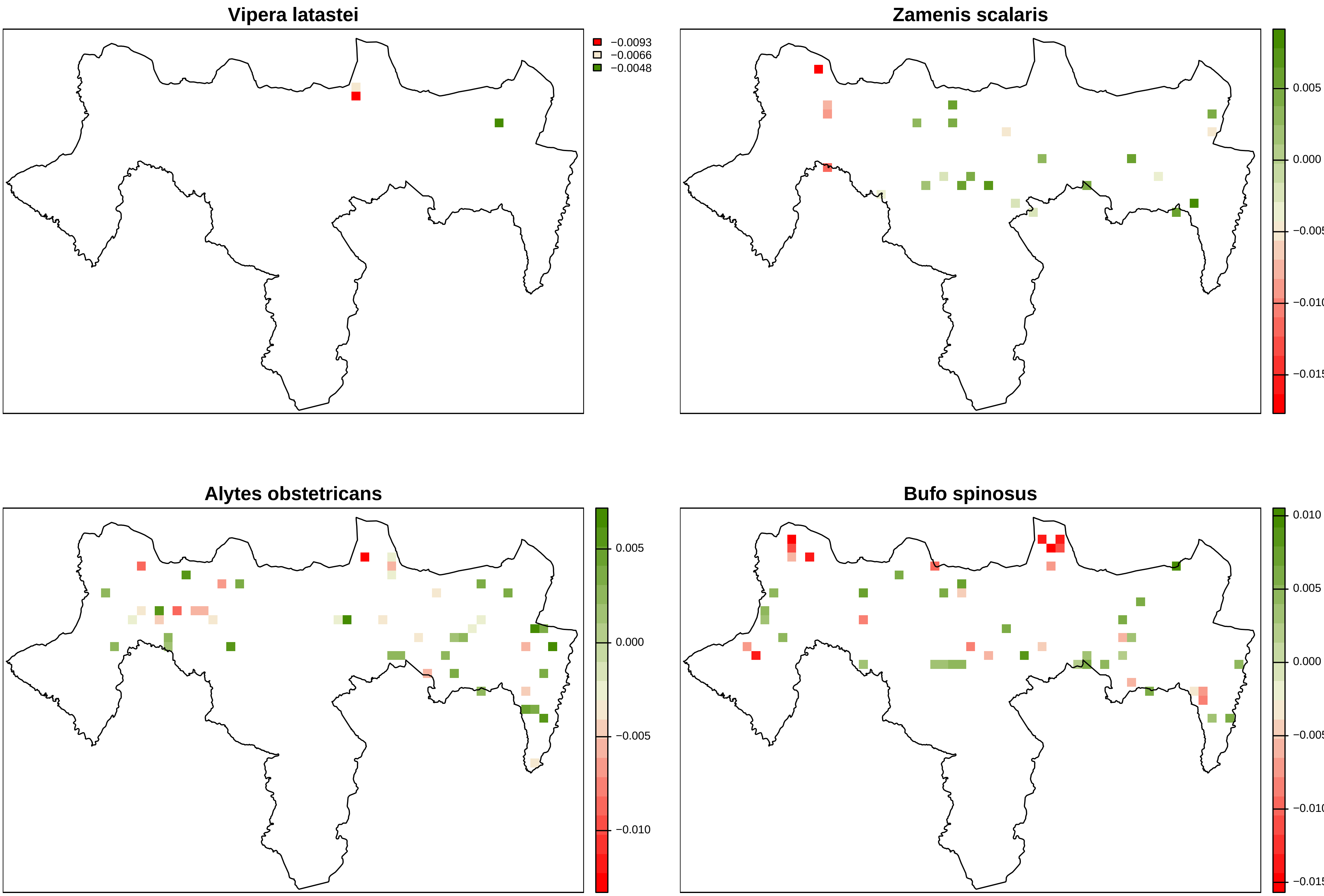

Vipera latastei
−0.0093
−0.0066
−0.0048
Zamenis scalaris
0.005
0.000
Alytes obstetricans
0.005
0.000
−0.005
−0.010
Bufo spinosus
0.010
0.005
0.000

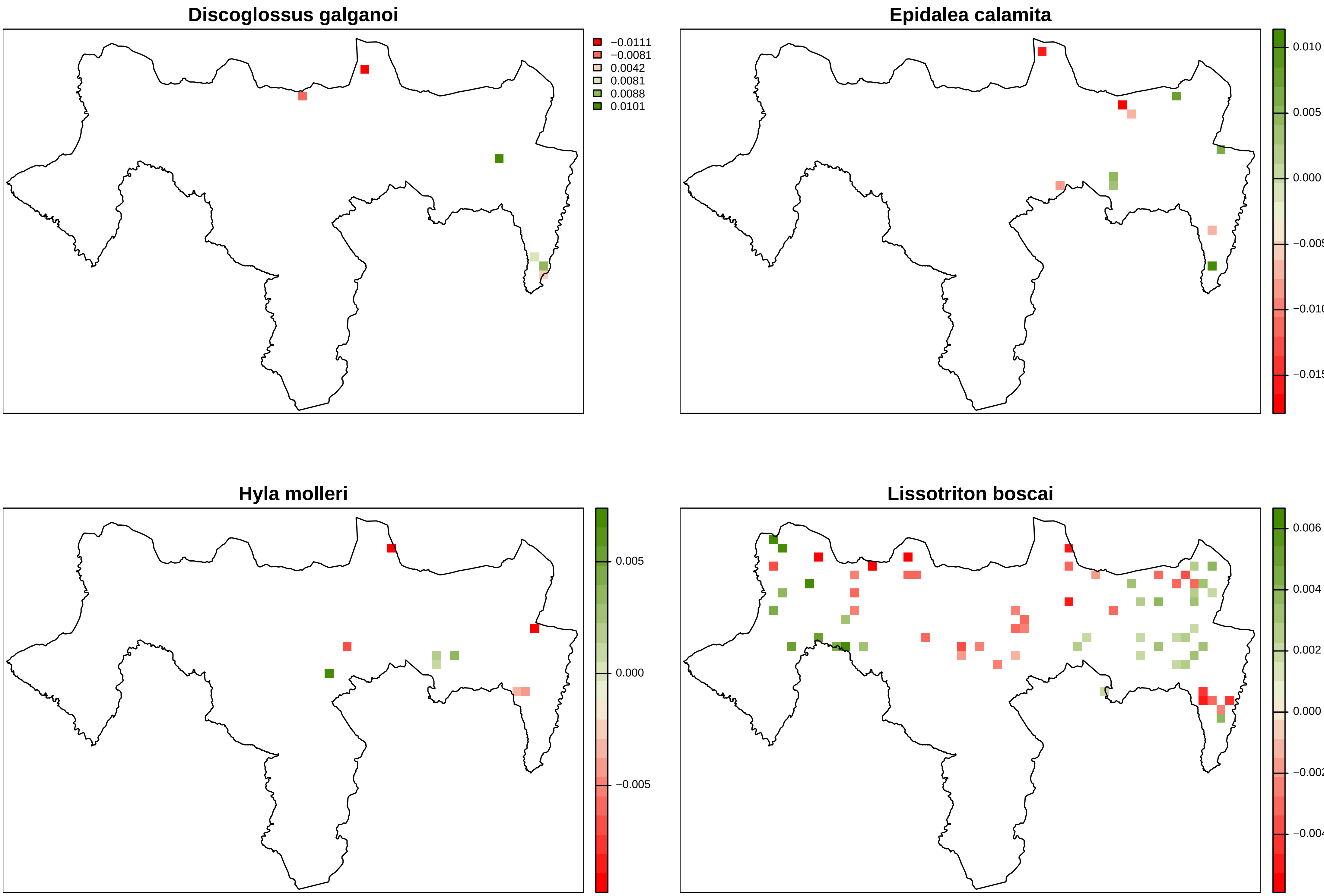

Discoglossus galganoi
−0.0111
−0.0081
0.0042
0.0081
0.0088
0.0101
Epidalea calamita
0.010
0.005
0.000
Hyla molleri
0.005
0.000
−0.005
Lissotriton boscai
0.006
0.004
0.002
0.000

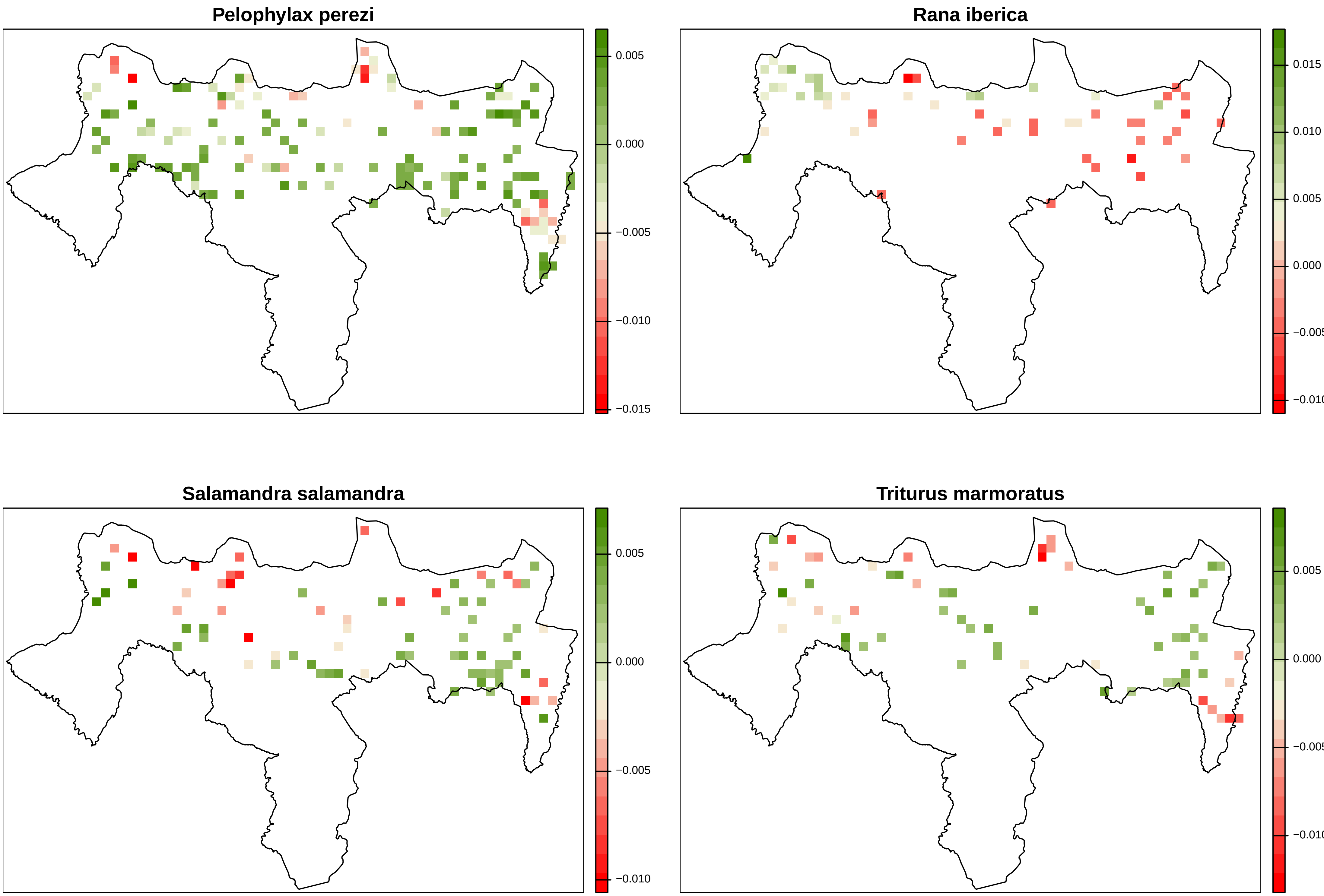
Pelophylax perezi
0.005
0.000
−0.005
−0.010
−0.015
Rana iberica
0.015
0.010
0.005
0.000
Salamandra salamandra
0.005
0.000
−0.005
−0.010
Triturus marmoratus
0.005
0.000

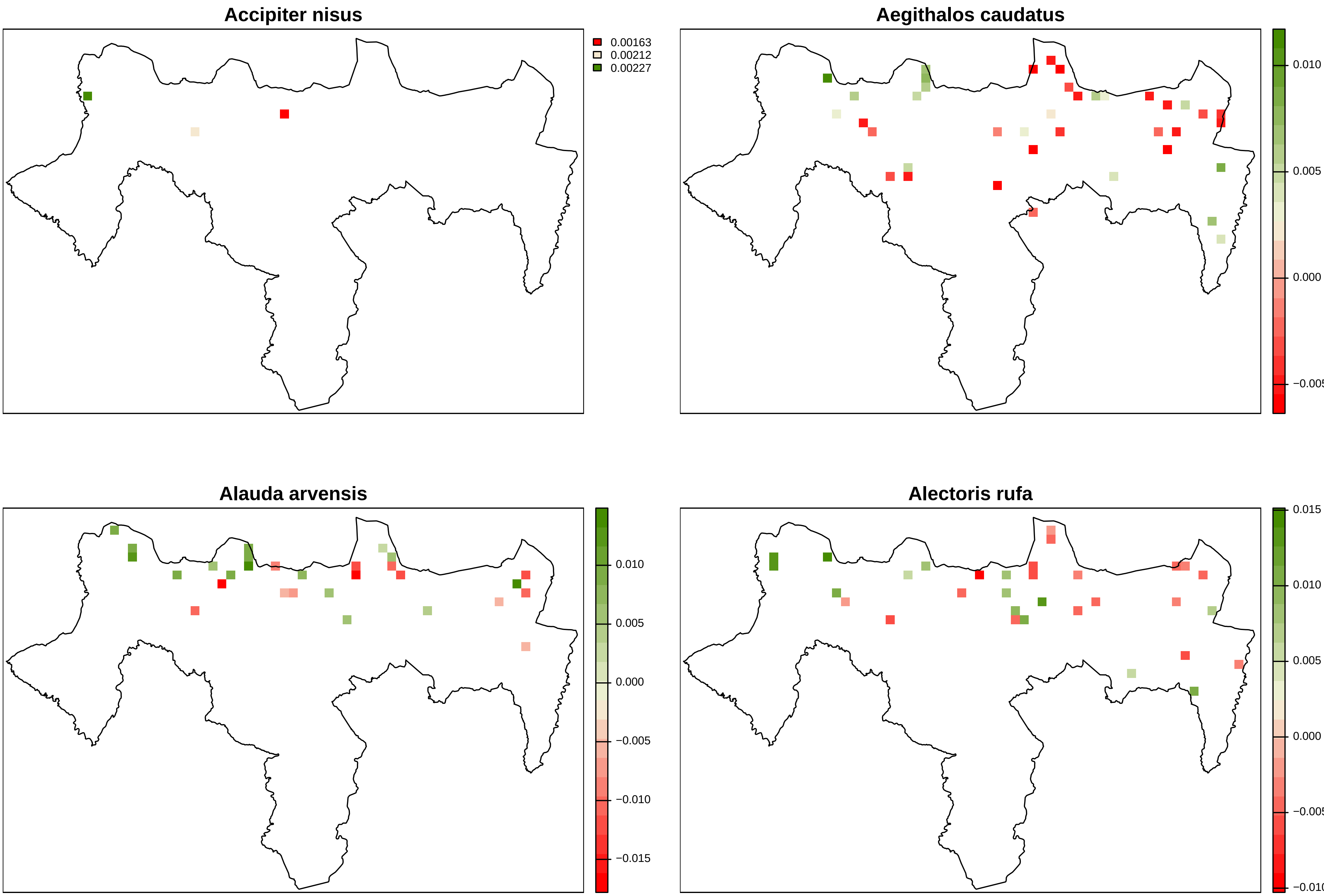
Accipiter nisus
0.00163
0.00212
0.00227
Aegithalos caudatus
0.010
0.005
0.000
Alauda arvensis
0.010
0.005
0.000
−0.005
−0.010
−0.015
Alectoris rufa
0.015
0.010
0.005
0.000

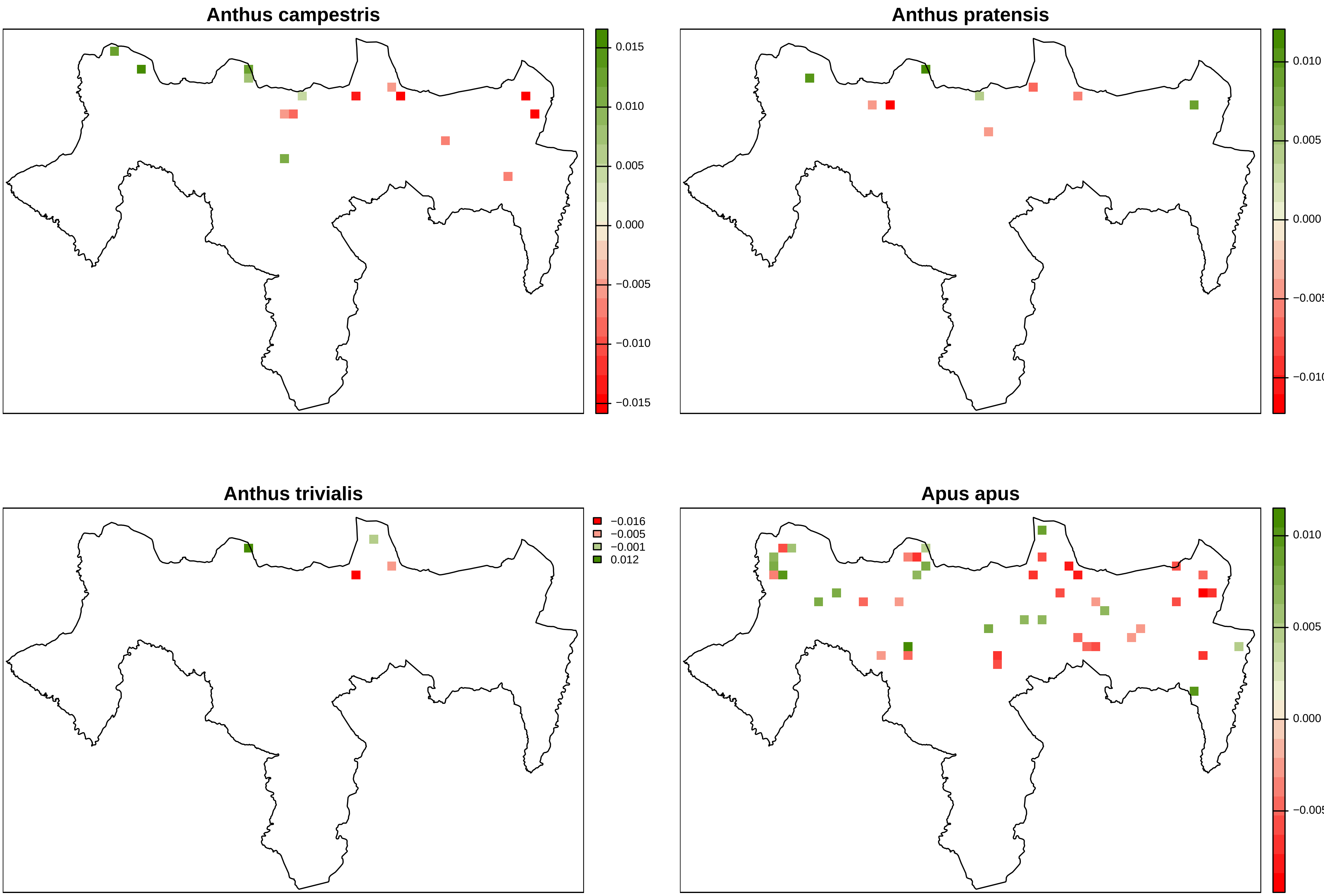
Anthus campestris
0.015
0.010
0.005
0.000
−0.005
−0.010
−0.015
Anthus pratensis
0.010
0.005
0.000
Anthus trivialis
−0.016
−0.005
−0.001
0.012
Apus apus
0.010
0.005
0.000

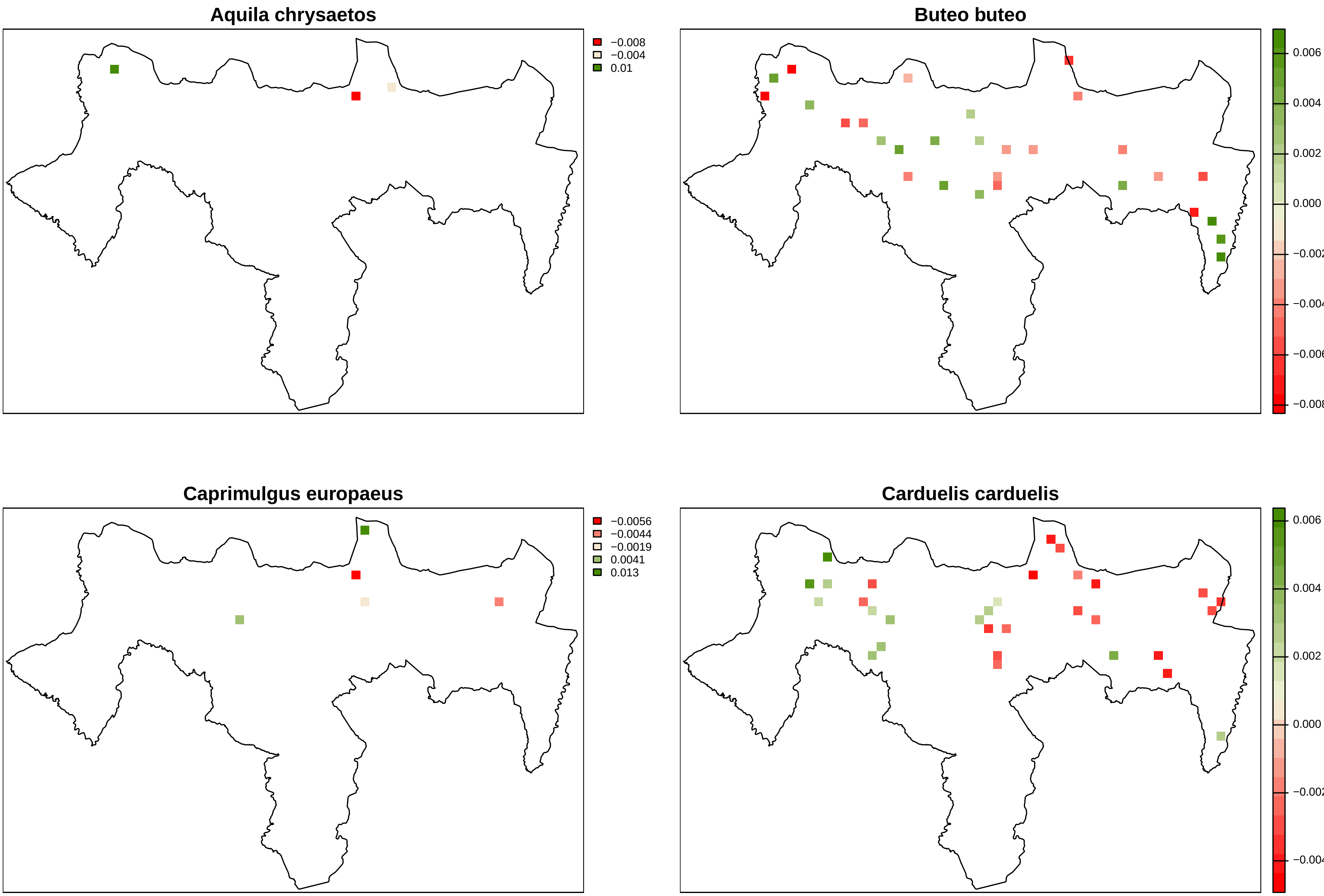
Aquila chrysaetos
−0.008
−0.004
0.01
Buteo buteo
0.006
0.004
0.002
0.000
Caprimulgus europaeus
−0.0056
−0.0044
−0.0019
0.0041
0.013
Carduelis carduelis
0.006
0.004
0.002
0.000

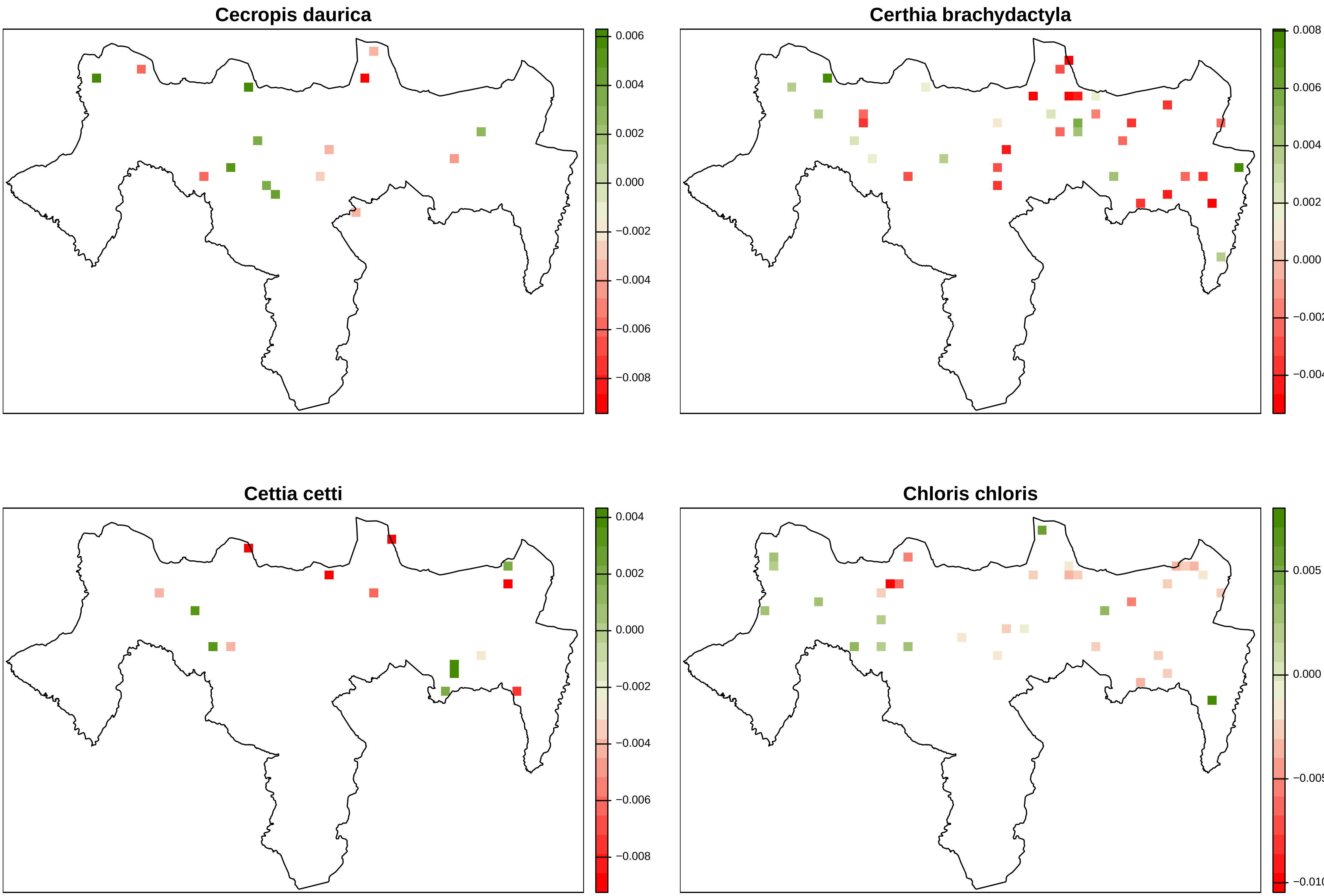
Cecropis daurica
0.006
0.004
0.002
0.000
−0.002
−0.004
−0.006
−0.008
Certhia brachydactyla
0.008
0.006
0.004
0.002
0.000
Cettia cetti
0.004
0.002
0.000
−0.002
−0.004
−0.006
−0.008
Chloris chloris
0.005
0.000

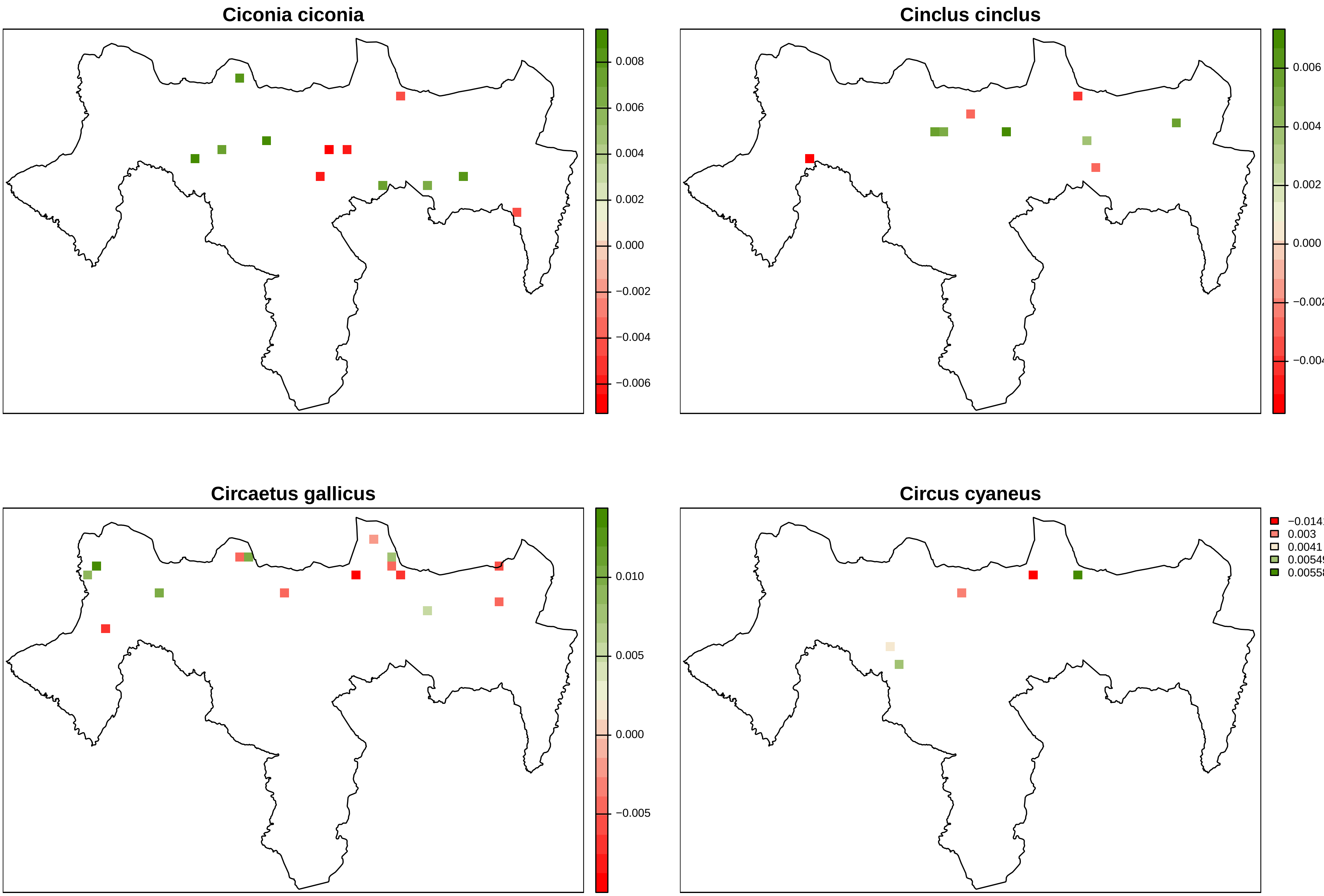

Ciconia ciconia
0.008
0.006
0.004
0.002
0.000
−0.002
−0.004
−0.006
Cinclus cinclus
0.006
0.004
0.002
0.000
Circaetus gallicus
0.010
0.005
0.000
−0.005
Circus cyaneus
0.003
0.0041

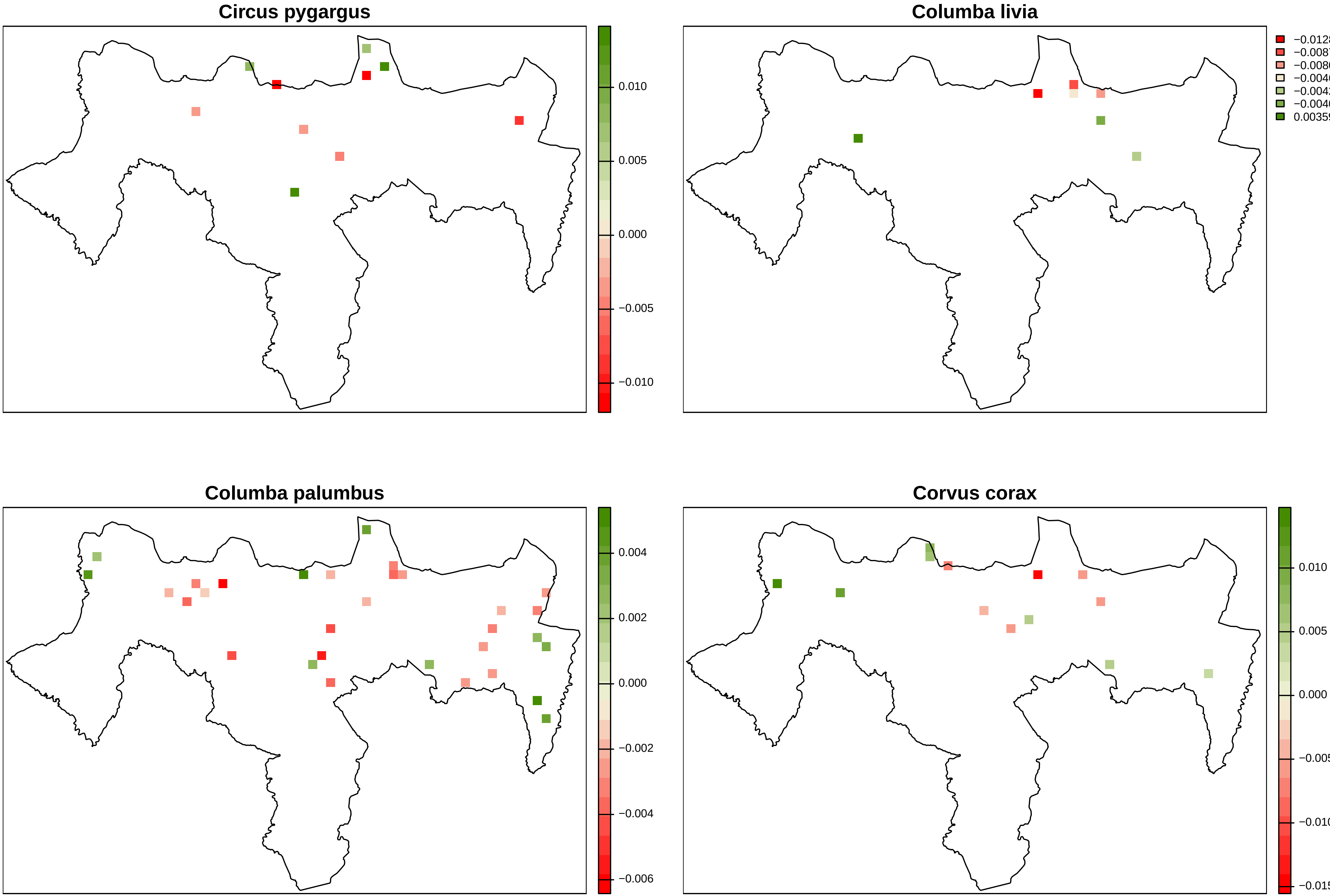
Circus pygargus
0.010
0.005
0.000
−0.005
−0.010
Columba livia
−0.0128
−0.0087
−0.0080
−0.0040
−0.0042
−0.0040
0.00359
Columba palumbus
0.004
0.002
0.000
−0.002
−0.004
−0.006
Corvus corax
0.010
0.005
0.000
−0.005
−0.010
−0.015

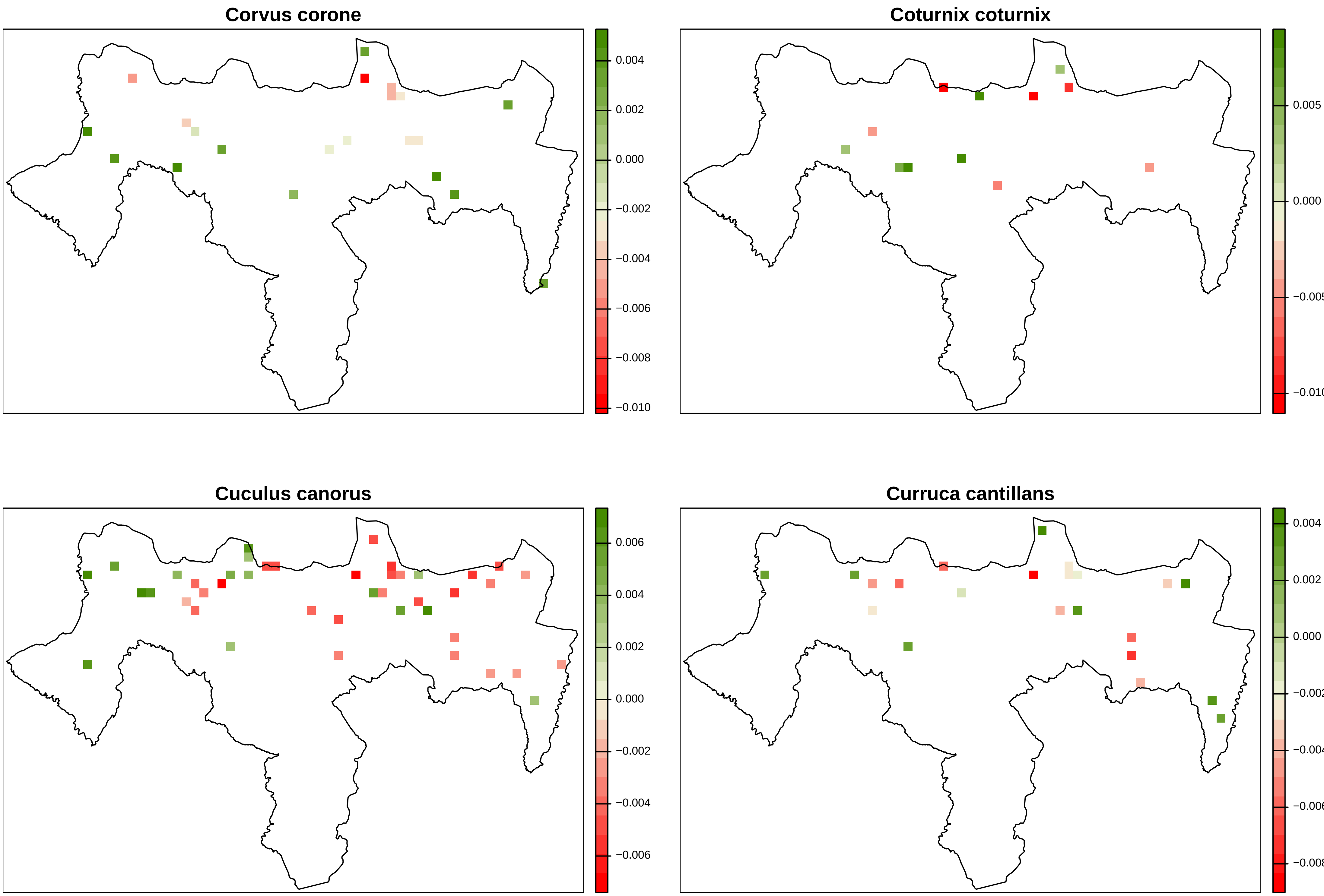
Corvus corone
0.004
0.002
0.000
−0.002
−0.004
−0.006
−0.008
−0.010
Coturnix coturnix
0.005
0.000
Cuculus canorus
0.006
0.004
0.002
0.000
−0.002
−0.004
−0.006
Curruca cantillans
0.004
0.002
0.000

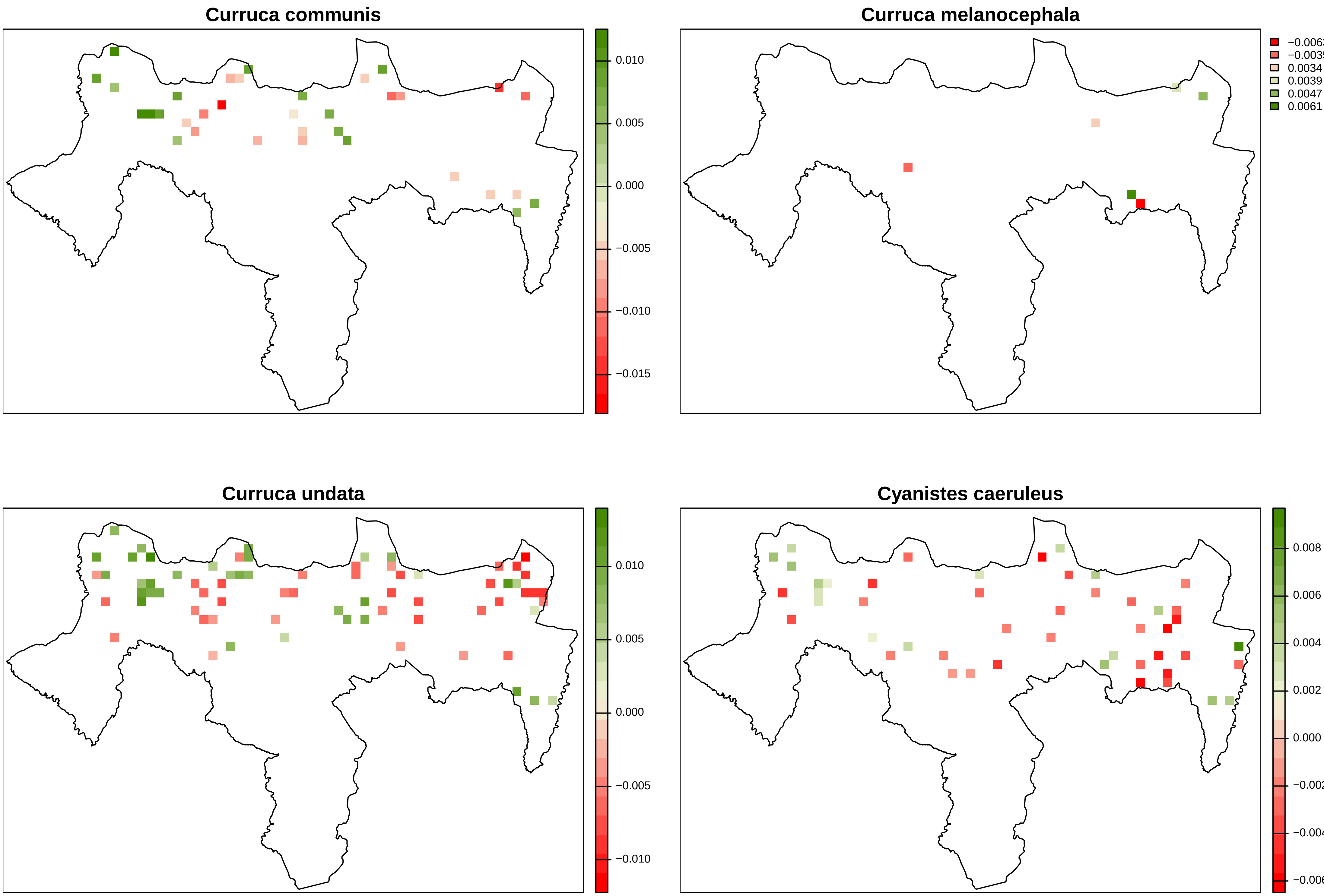
Curruca communis
0.010
0.005
0.000
−0.005
−0.010
−0.015
Curruca melanocephala
0.0034
0.0039
0.0047
0.0061
Curruca undata
0.010
0.005
0.000
−0.005
−0.010
Cyanistes caeruleus
0.008
0.006
0.004
0.002
0.000

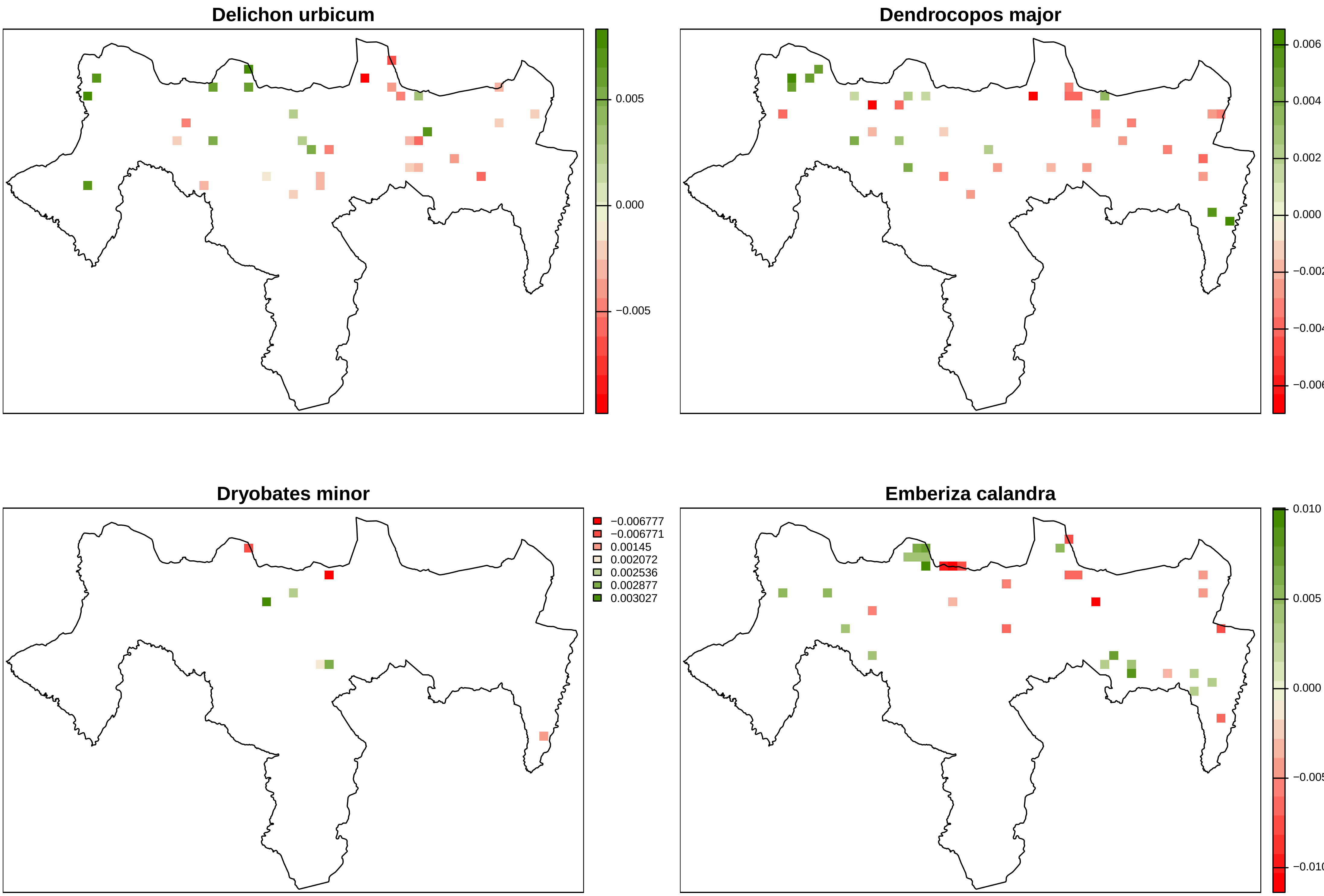

Delichon urbicum
0.005
0.000
−0.005
Dendrocopos major
0.006
0.004
0.002
0.000
Dryobates minor
−0.006777
−0.006771
0.00145
0.002072
0.002536
0.002877
0.003027
Emberiza calandra
0.010
0.005
0.000

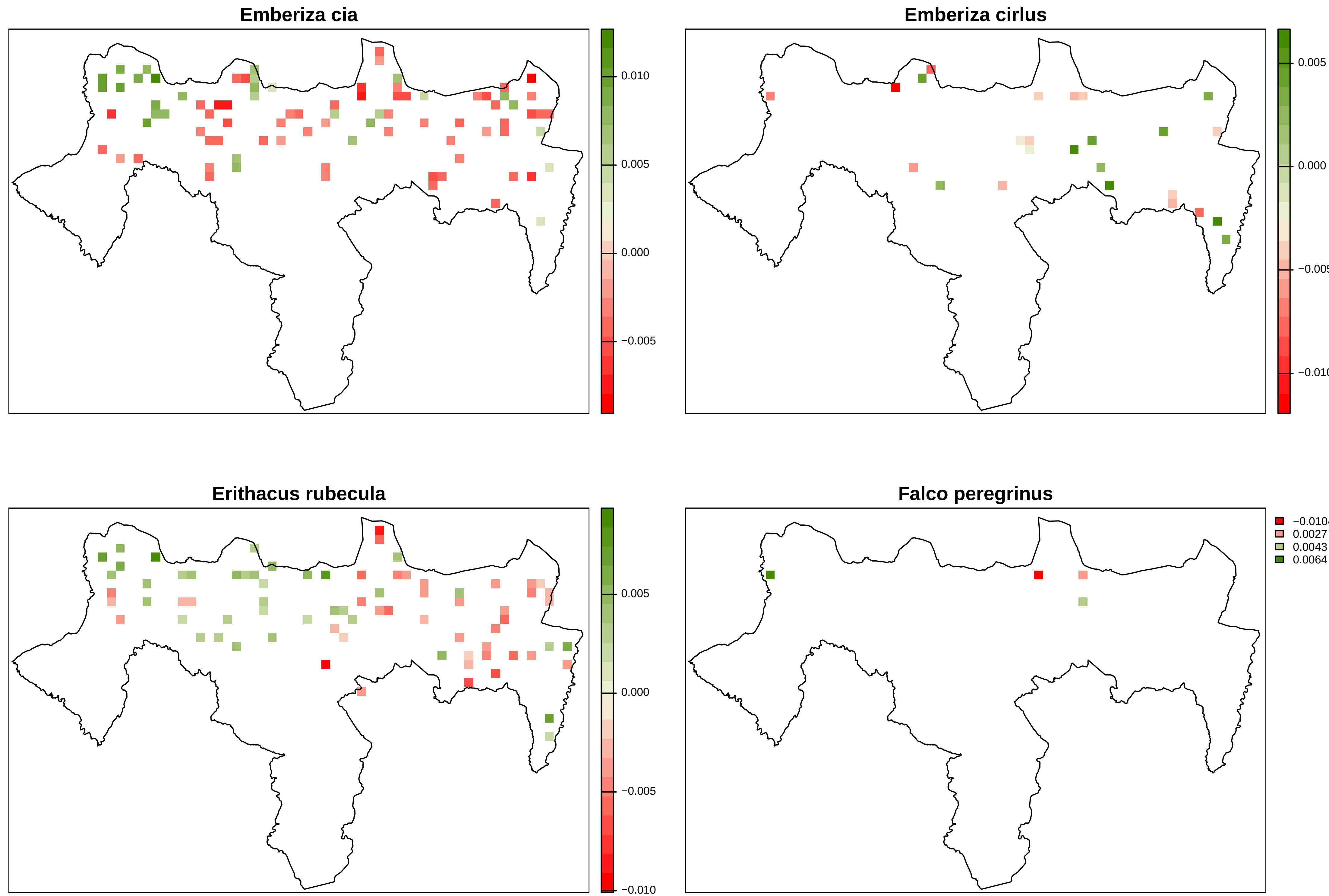

Emberiza cia
0.010
0.005
0.000
−0.005
Emberiza cirlus
0.005
0.000
Erithacus rubecula
0.005
0.000
−0.005
−0.010
Falco peregrinus
0.0027
0.0043
0.0064

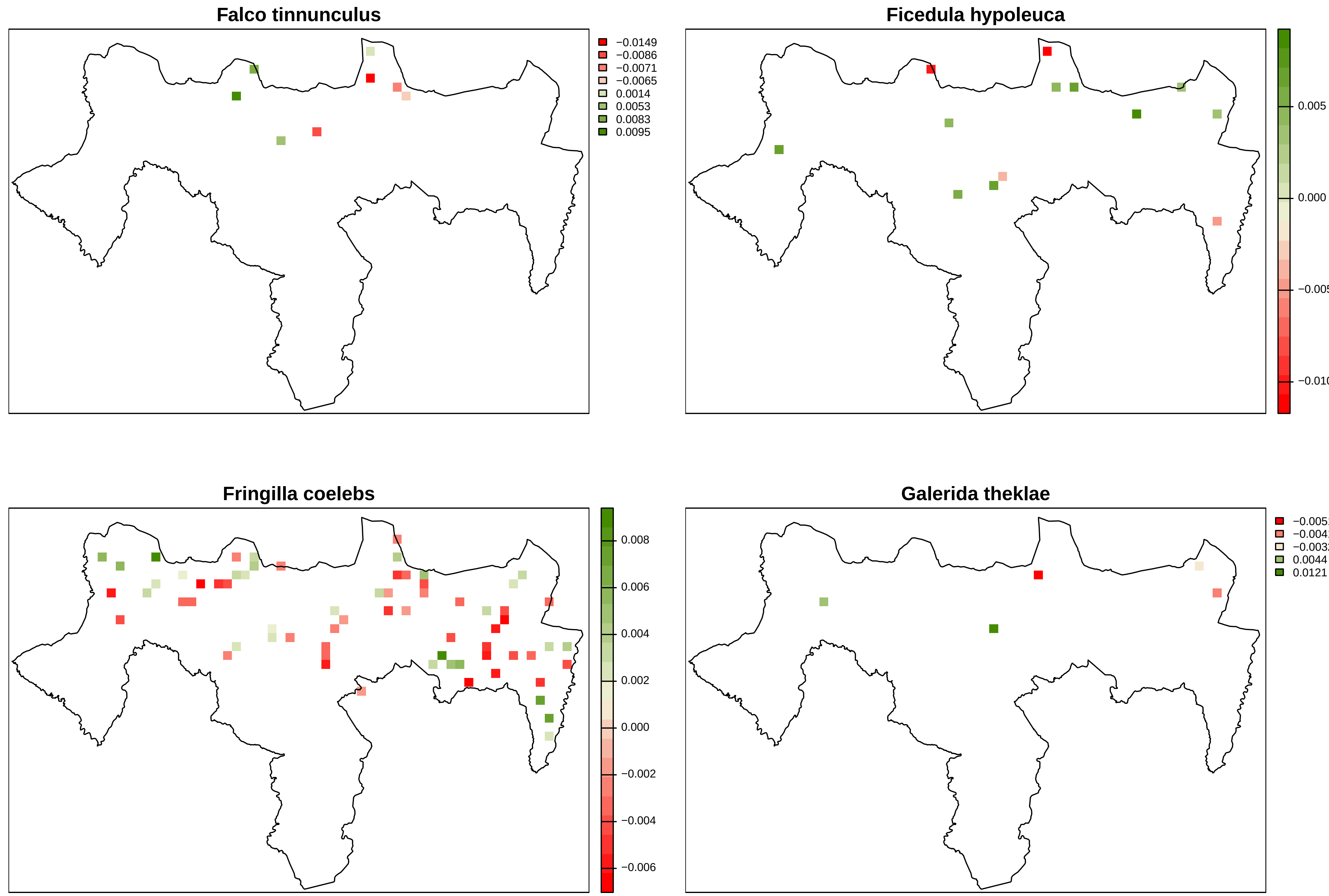
Falco tinnunculus
−0.0149
−0.0086
−0.0071
−0.0065
0.0014
0.0053
0.0083
0.0095
Ficedula hypoleuca
0.005
0.000
Fringilla coelebs
0.008
0.006
0.004
0.002
0.000
−0.002
−0.004
−0.006
Galerida theklae
0.0044
0.0121

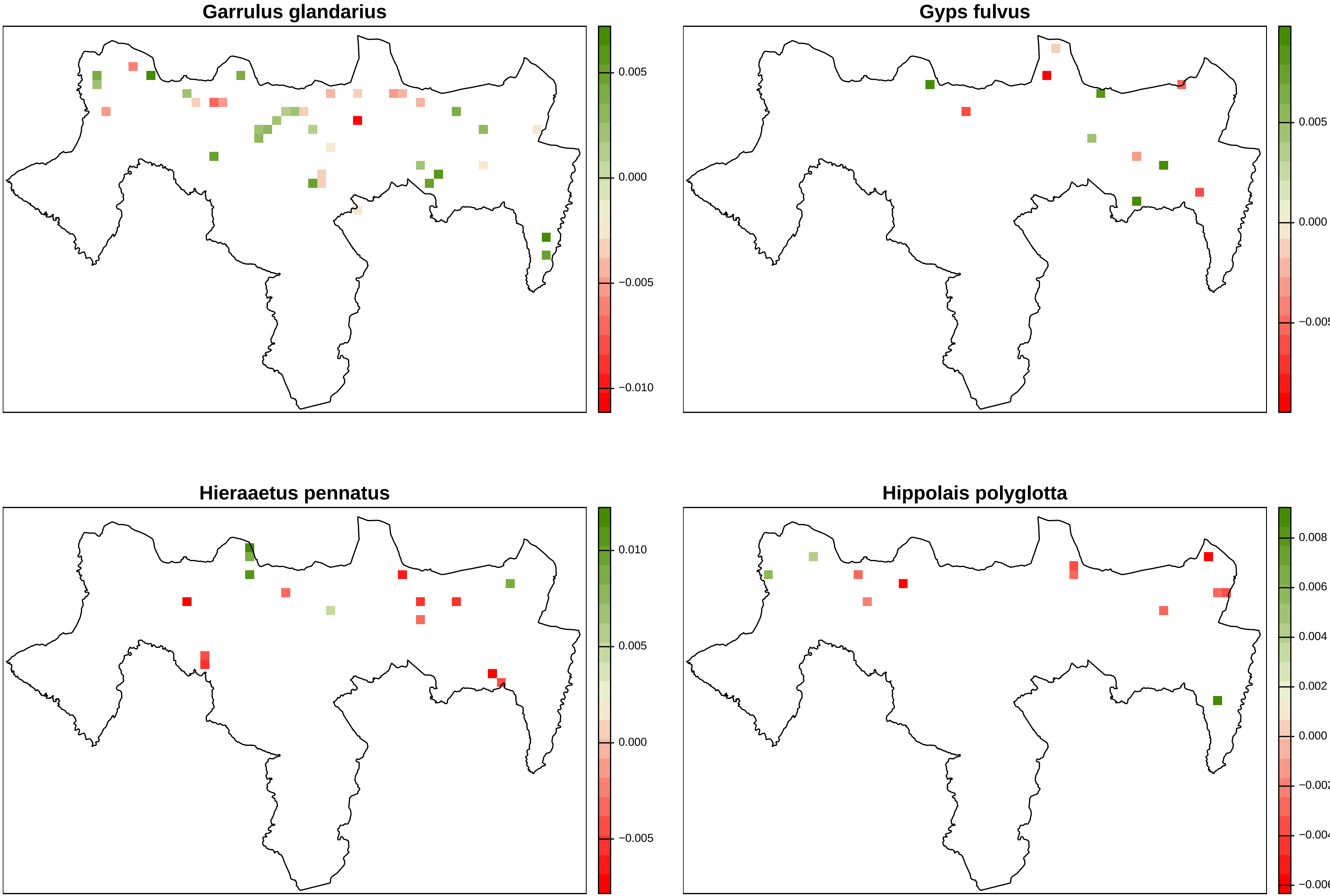
Garrulus glandarius
0.005
0.000
−0.005
−0.010
Gyps fulvus
0.005
0.000
Hieraaetus pennatus
0.010
0.005
0.000
−0.005
Hippolais polyglotta
0.008
0.006
0.004
0.002
0.000

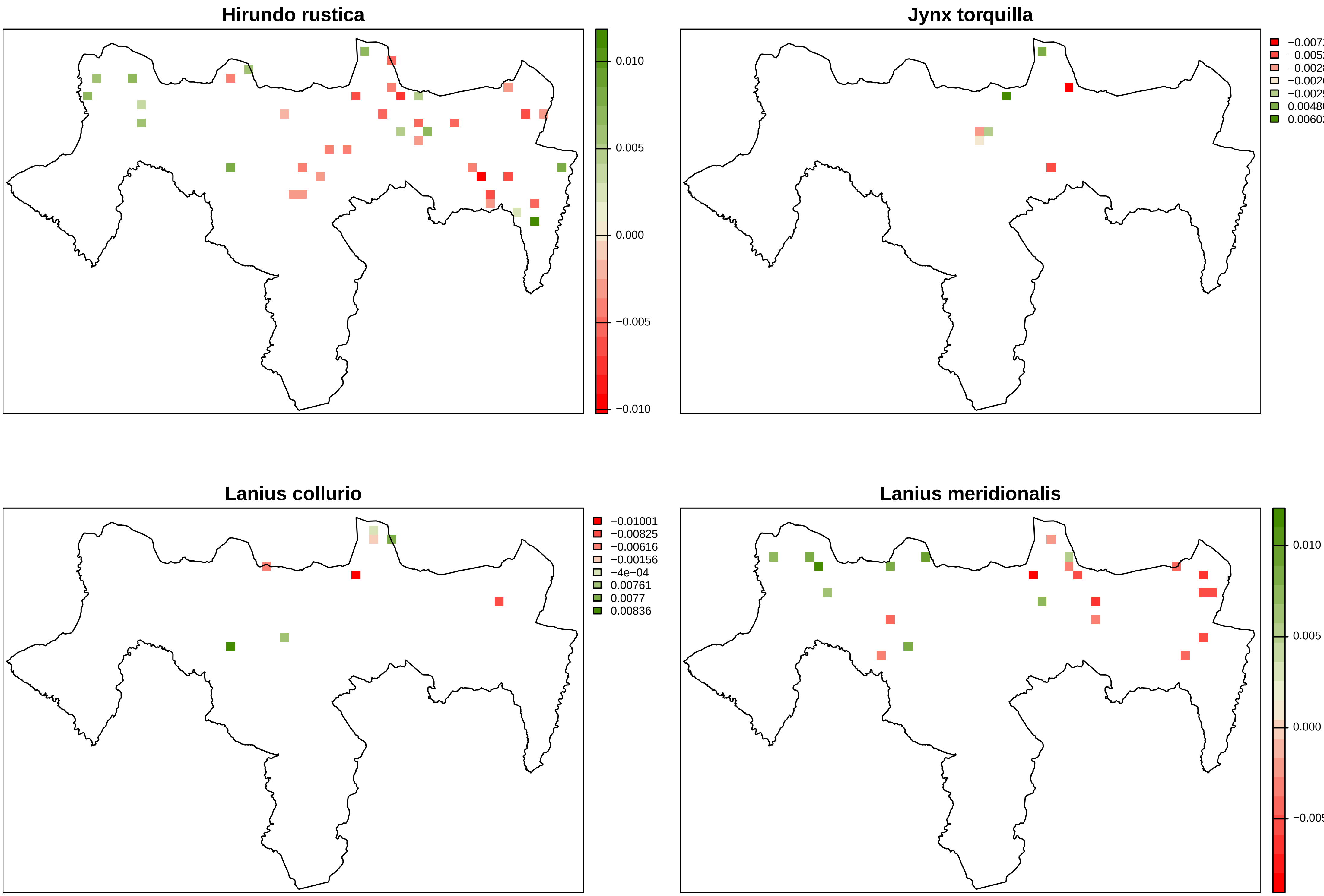
Hirundo rustica
0.010
0.005
0.000
−0.005
−0.010
Jynx torquilla
Lanius collurio
−0.01001
−0.00825
−0.00616
−0.00156
−4e−04
0.00761
0.0077
0.00836
Lanius meridionalis
0.010
0.005
0.000

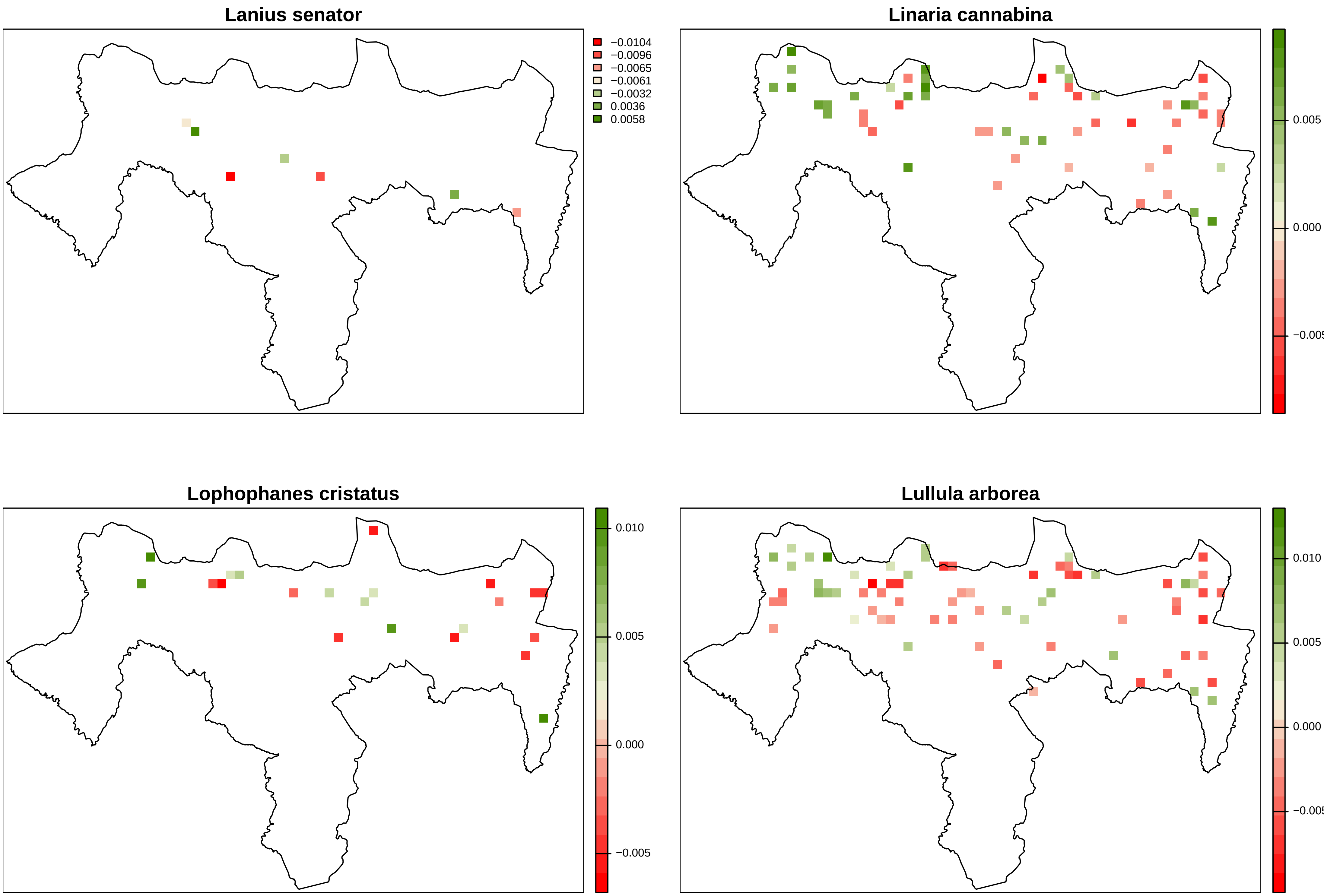

Lanius senator
−0.0104
−0.0096
−0.0065
−0.0061
−0.0032
0.0036
0.0058
Linaria cannabina
0.005
0.000
Lophophanes cristatus
0.010
0.005
0.000
−0.005
Lullula arborea
0.010
0.005
0.000

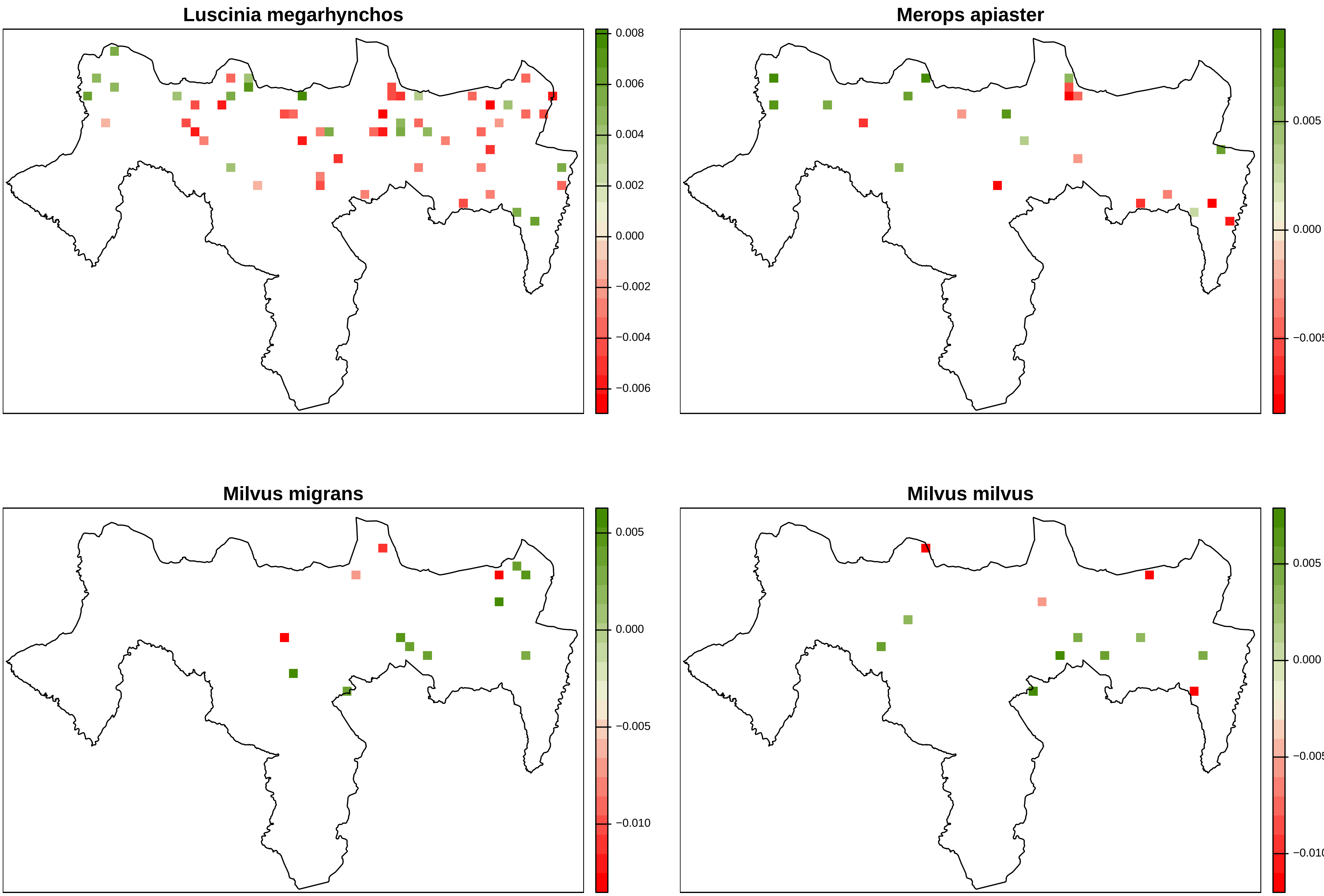
Luscinia megarhynchos
0.008
0.006
0.004
0.002
0.000
−0.002
−0.004
−0.006
Merops apiaster
0.005
0.000
Milvus migrans
0.005
0.000
−0.005
−0.010
Milvus milvus
0.005
0.000

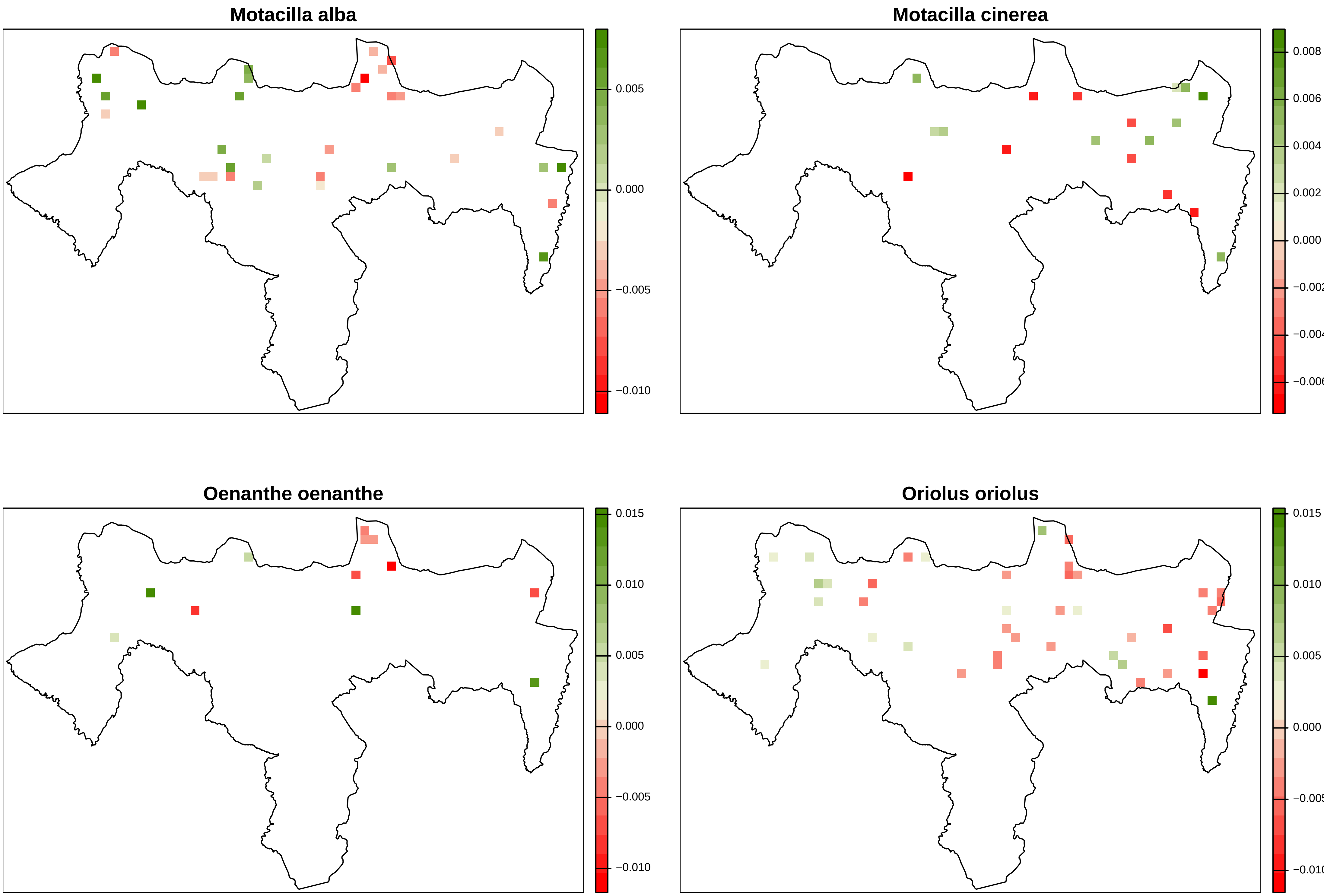
Motacilla alba
0.005
0.000
−0.005
−0.010
Motacilla cinerea
0.008
0.006
0.004
0.002
0.000
Oenanthe oenanthe
0.015
0.010
0.005
0.000
−0.005
−0.010
Oriolus oriolus
0.015
0.010
0.005
0.000

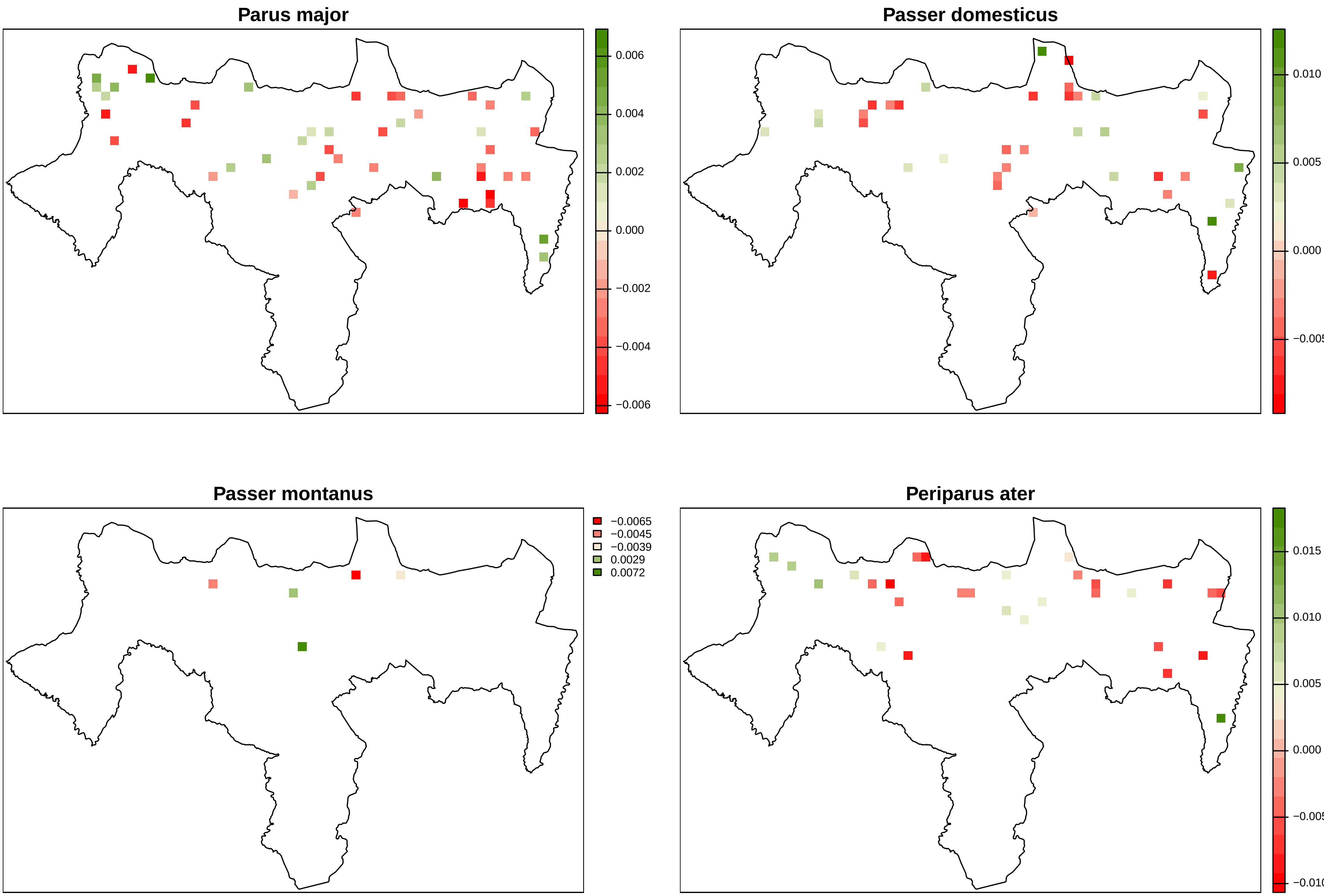

Parus major
0.006
0.004
0.002
0.000
−0.002
−0.004
−0.006
Passer domesticus
0.010
0.005
0.000
Passer montanus
−0.0065
−0.0045
−0.0039
0.0029
0.0072
Periparus ater
0.015
0.010
0.005
0.000

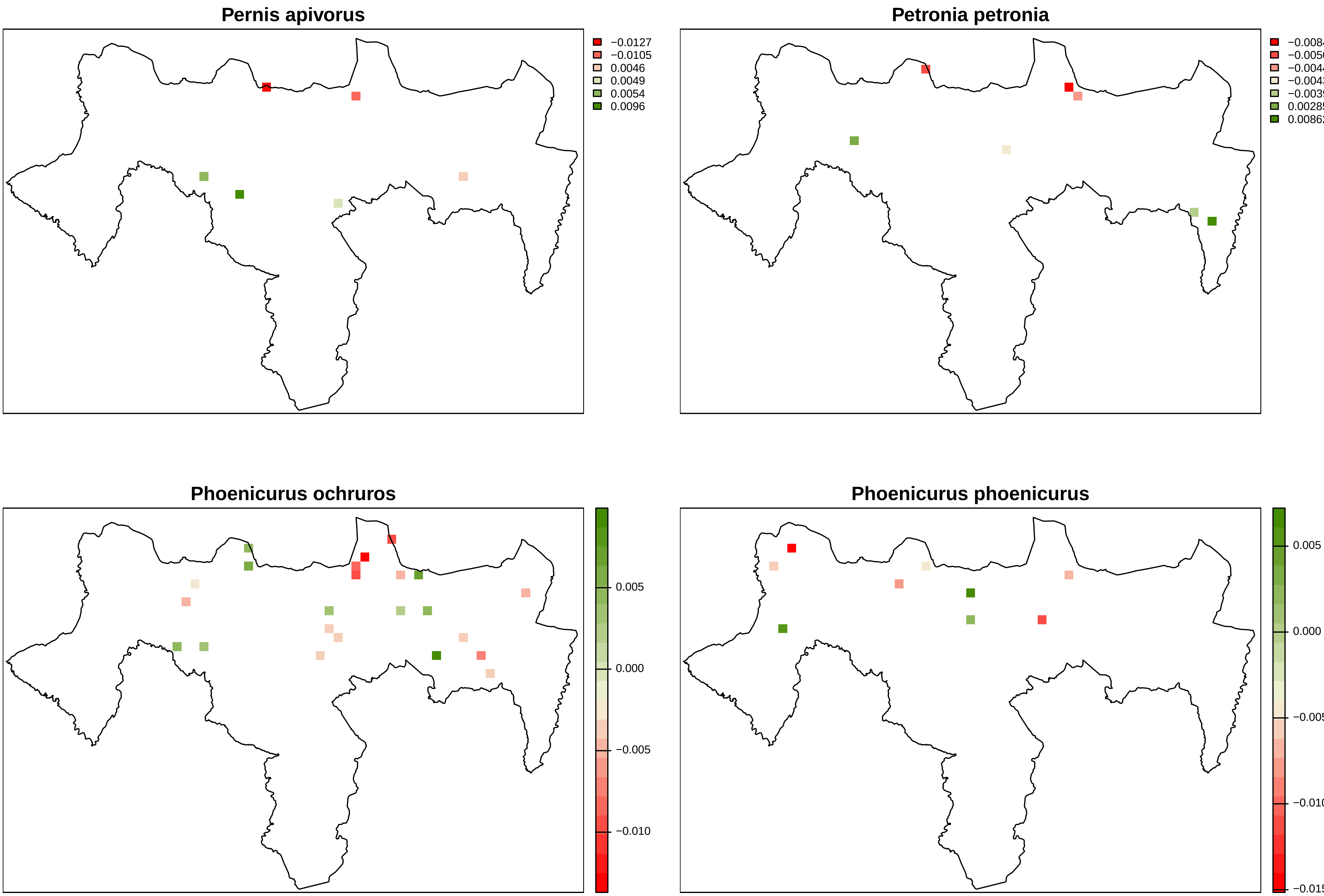
Pernis apivorus
−0.0127
−0.0105
0.0046
0.0049
0.0054
0.0096
Petronia petronia
−0.0084
−0.0050
−0.0044
−0.0043
−0.0039
0.00285
0.00862
Phoenicurus ochruros
0.005
0.000
−0.005
−0.010
Phoenicurus phoenicurus
0.005
0.000
−0.005
−0.010
−0.015

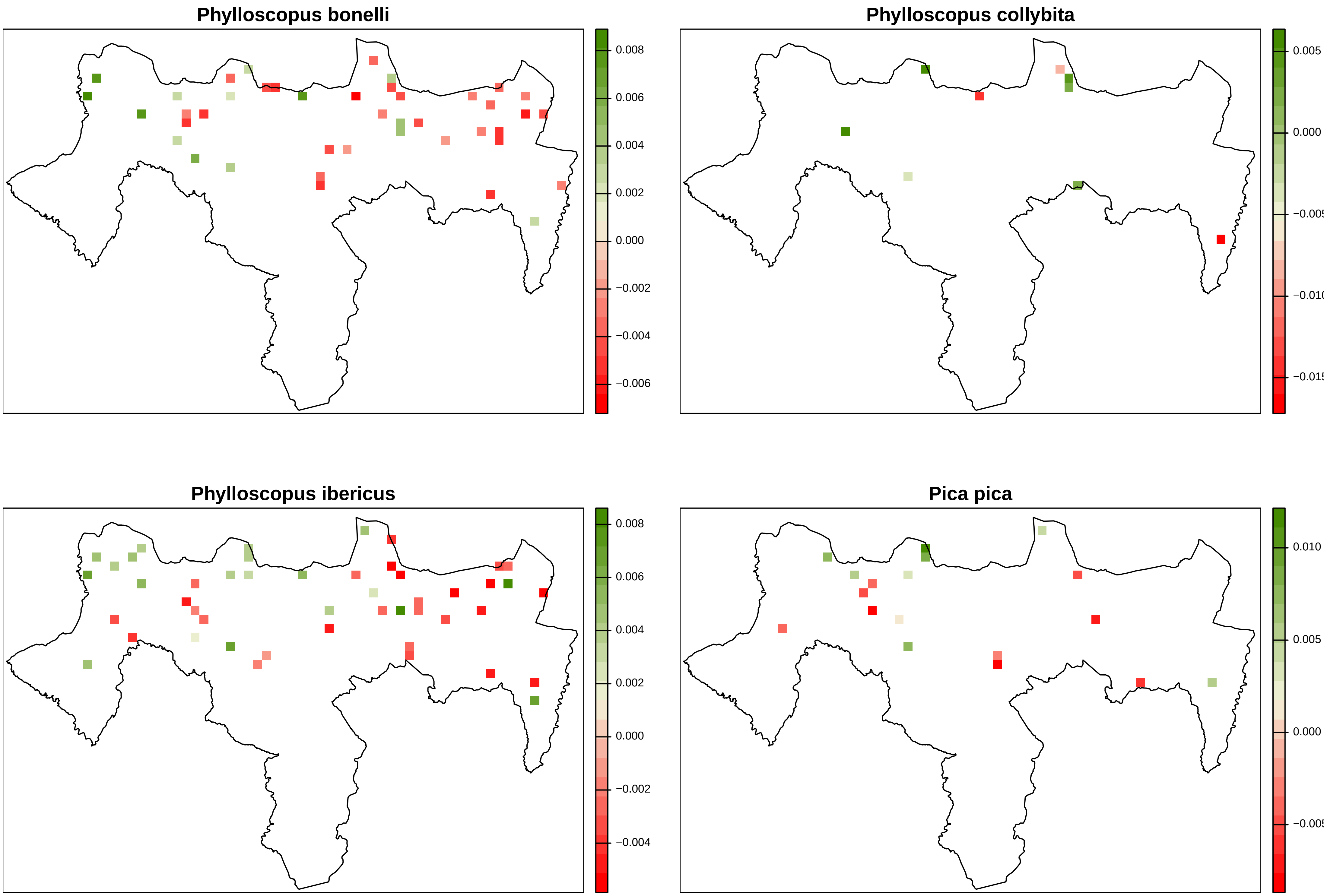

Phylloscopus bonelli
0.008
0.006
0.004
0.002
0.000
−0.002
−0.004
−0.006
Phylloscopus collybita
0.005
0.000
Phylloscopus ibericus
0.008
0.006
0.004
0.002
0.000
−0.002
−0.004
Pica pica
0.010
0.005
0.000

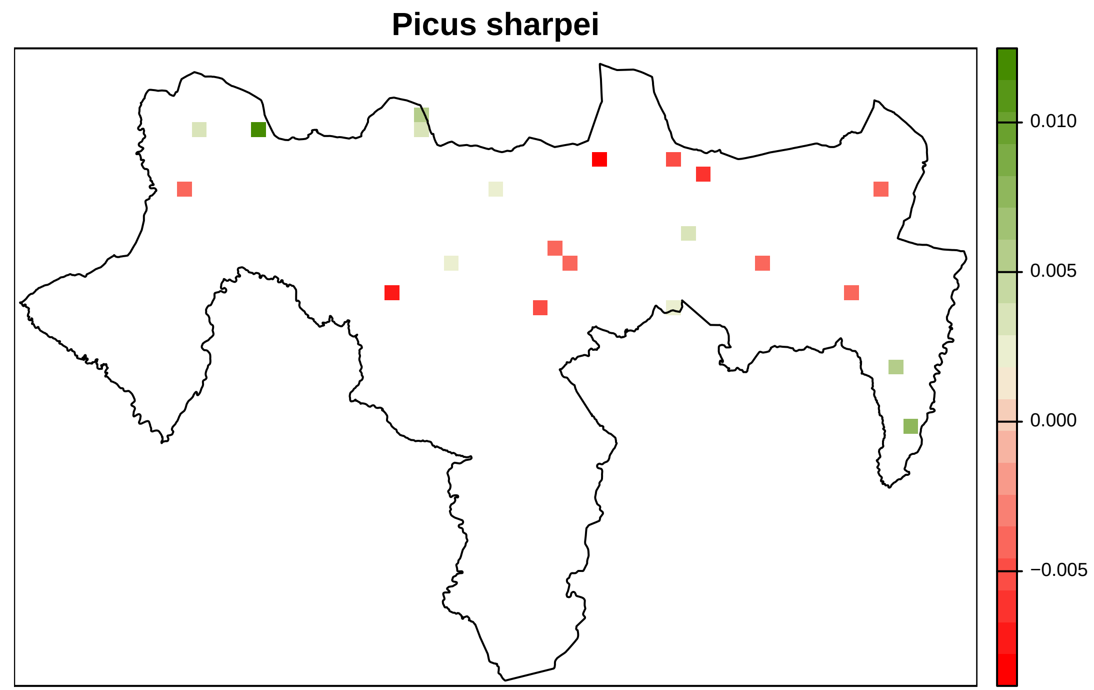
Picus sharpei
0.010
0.005
0.000
−0.005

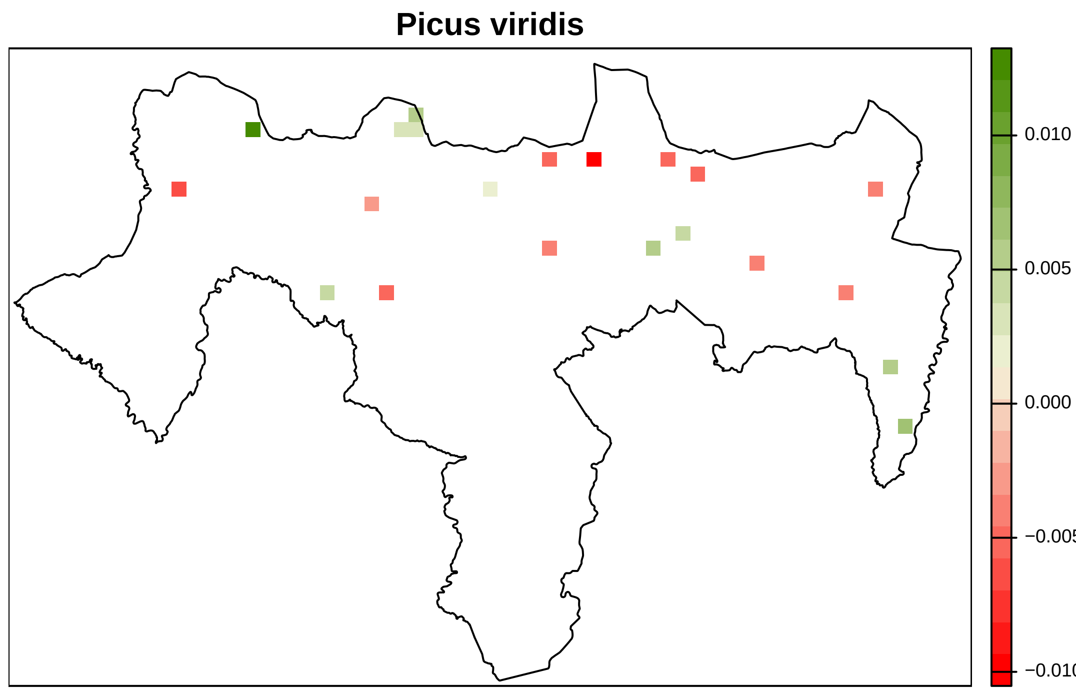
Picus viridis
0.010
0.005
0.000

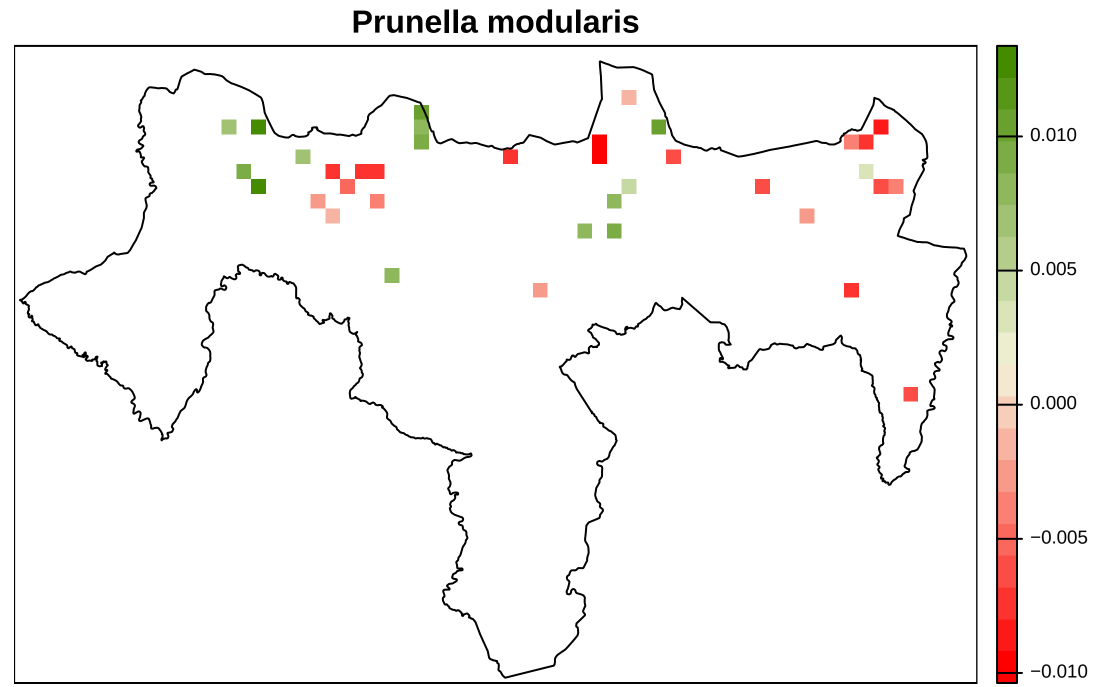
Prunella modularis
0.010
0.005
0.000
−0.005
−0.010

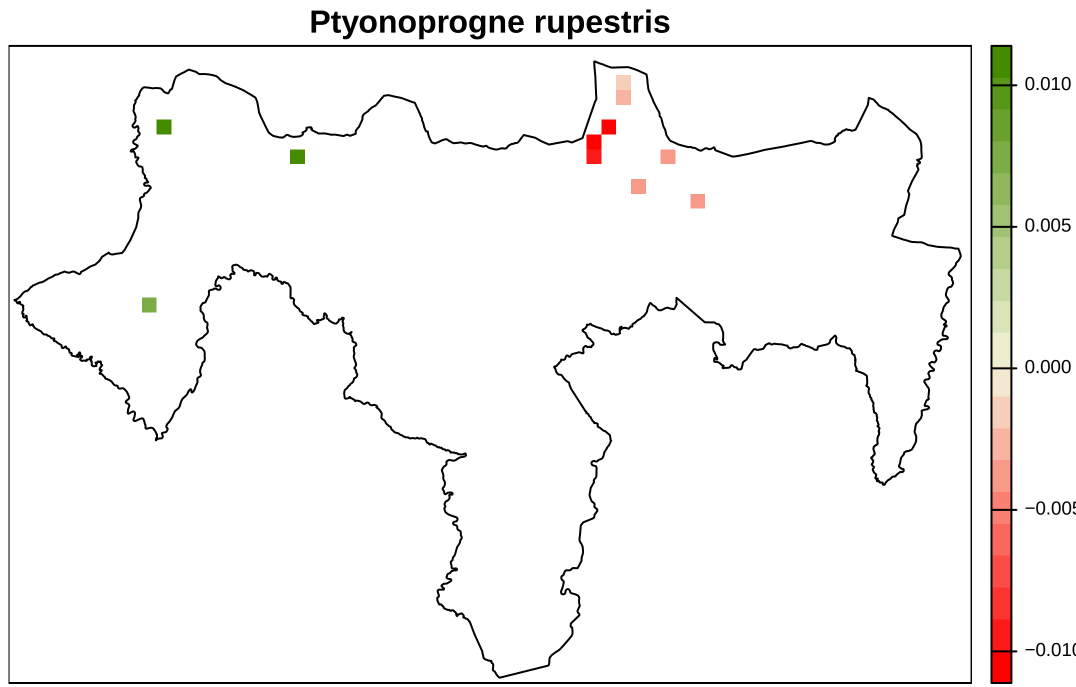
Ptyonoprogne rupestris
0.010
0.005
0.000

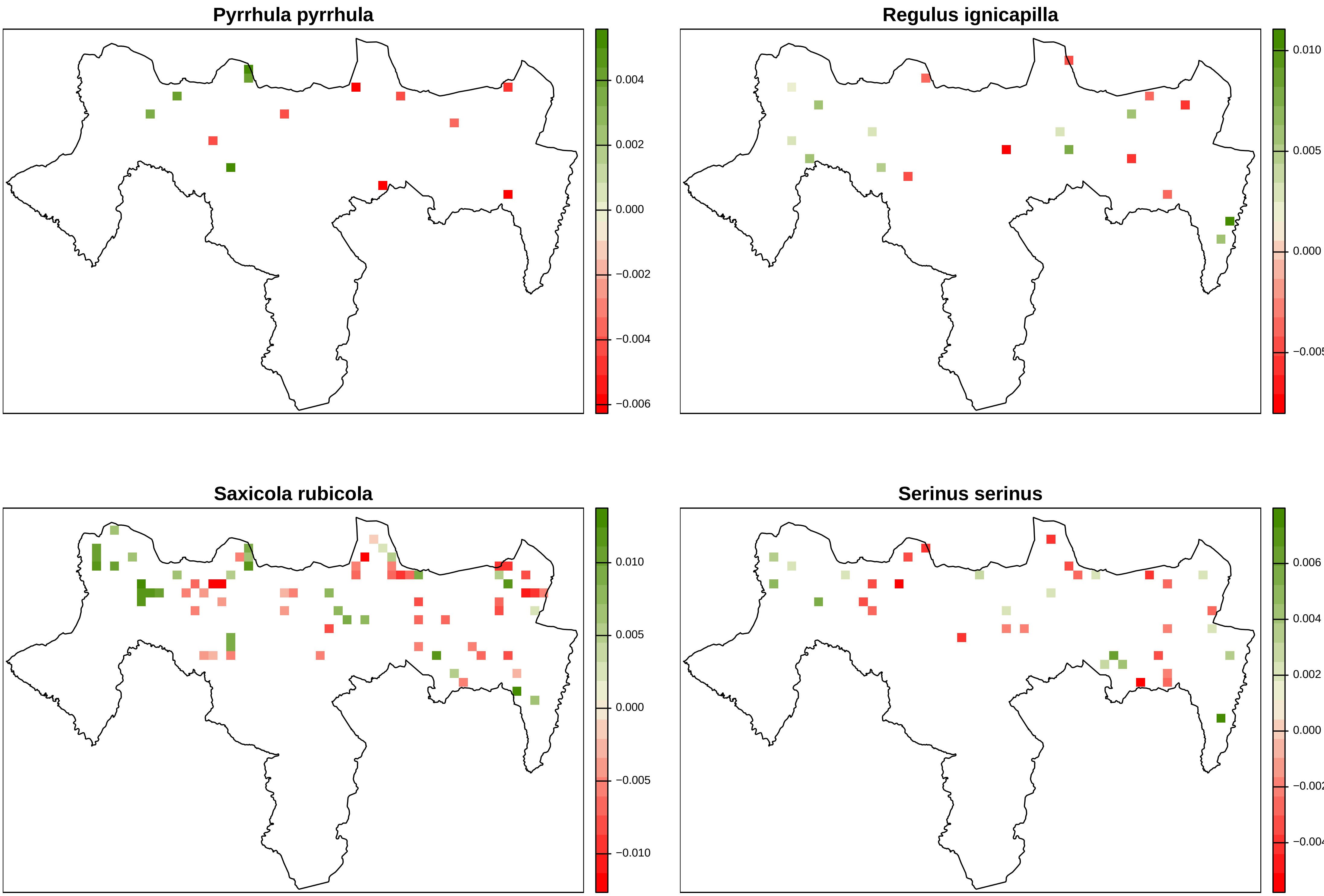

Pyrrhula pyrrhula
0.004
0.002
0.000
−0.002
−0.004
−0.006
Regulus ignicapilla
0.010
0.005
0.000
Saxicola rubicola
0.010
0.005
0.000
−0.005
−0.010
Serinus serinus
0.006
0.004
0.002
0.000

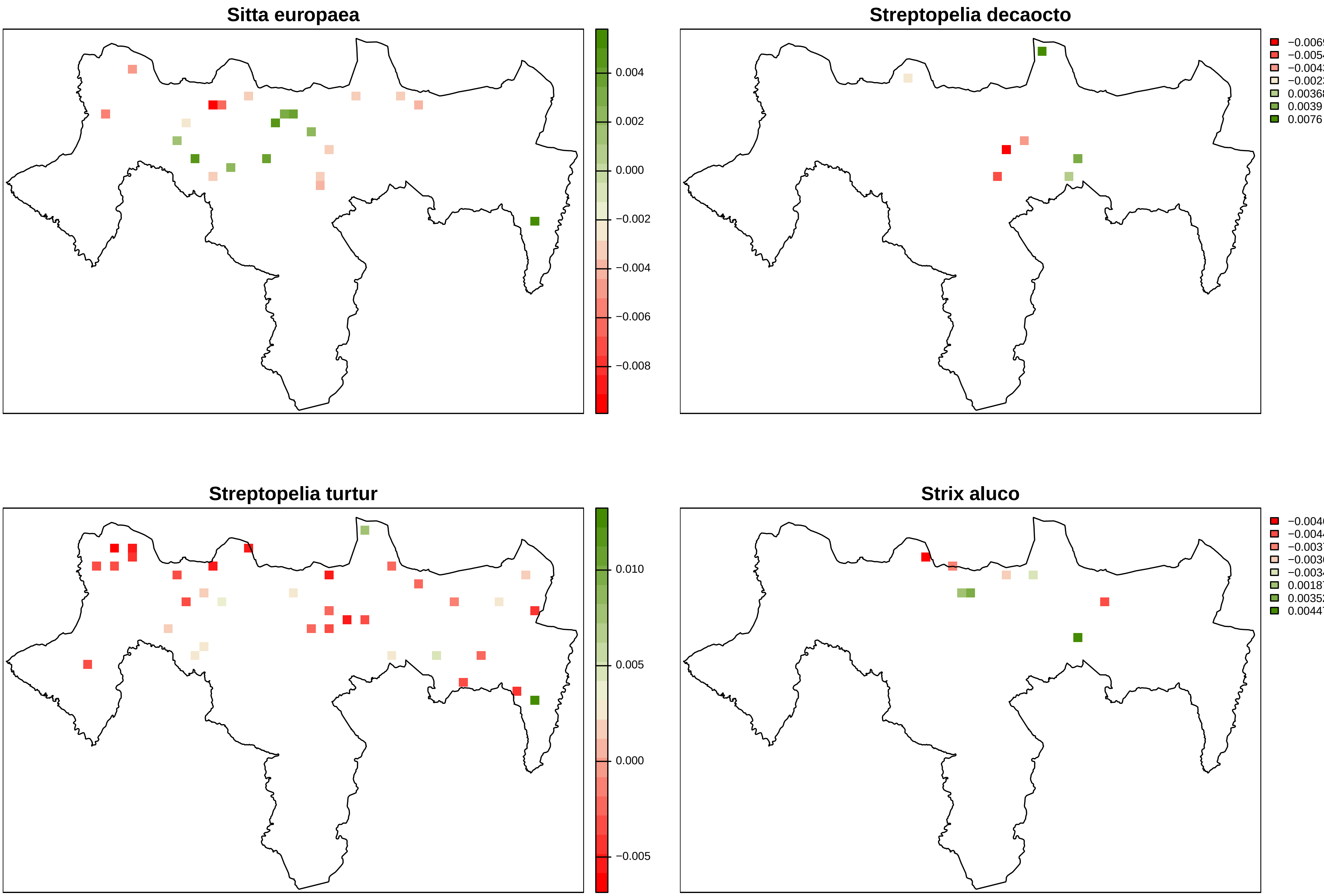
Sitta europaea
0.004
0.002
0.000
−0.002
−0.004
−0.006
−0.008
Streptopelia decaocto
Streptopelia turtur
0.010
0.005
0.000
−0.005
Strix aluco

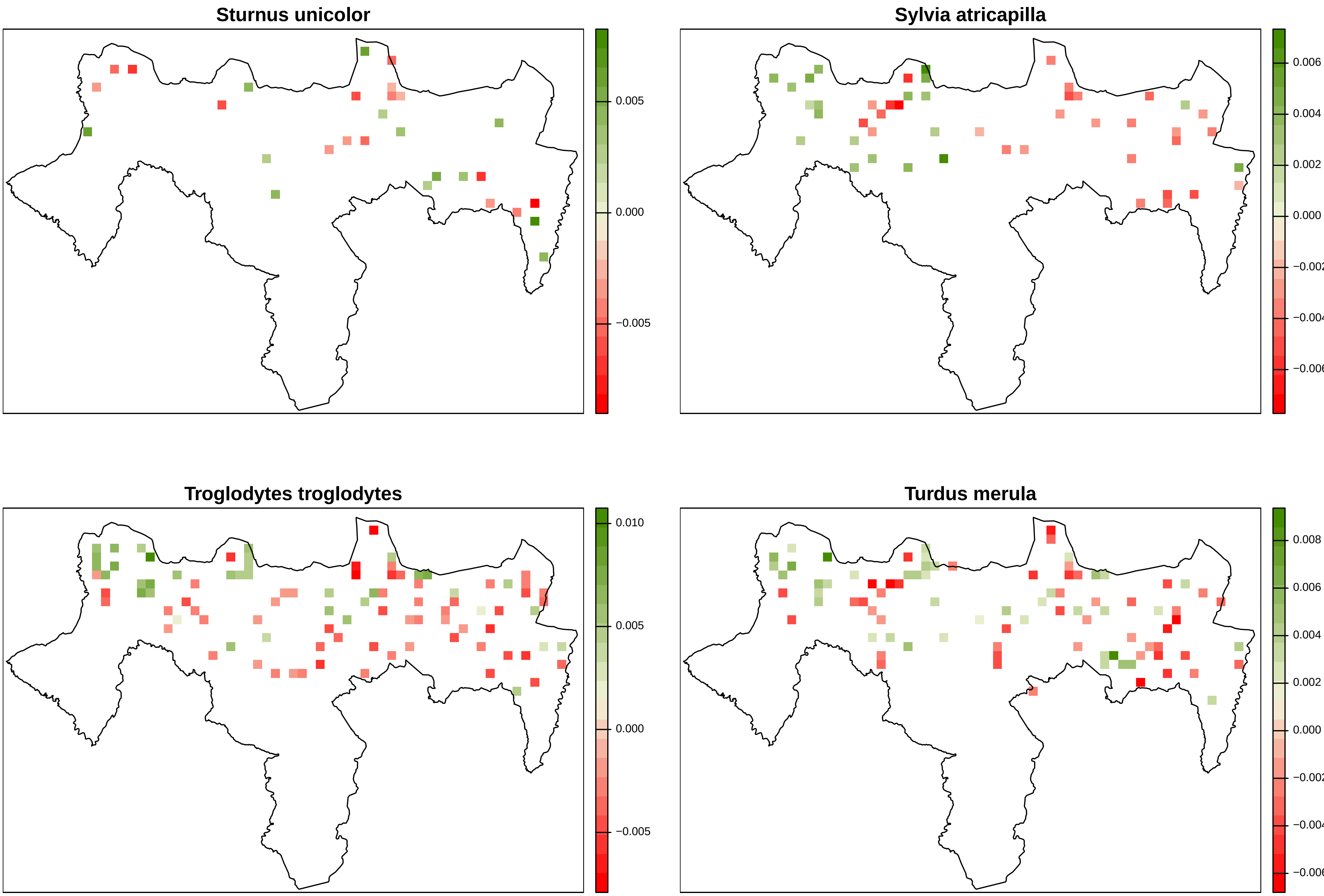

Sturnus unicolor
0.005
0.000
−0.005
Sylvia atricapilla
0.006
0.004
0.002
0.000
Troglodytes troglodytes
0.010
0.005
0.000
−0.005
Turdus merula
0.008
0.006
0.004
0.002
0.000

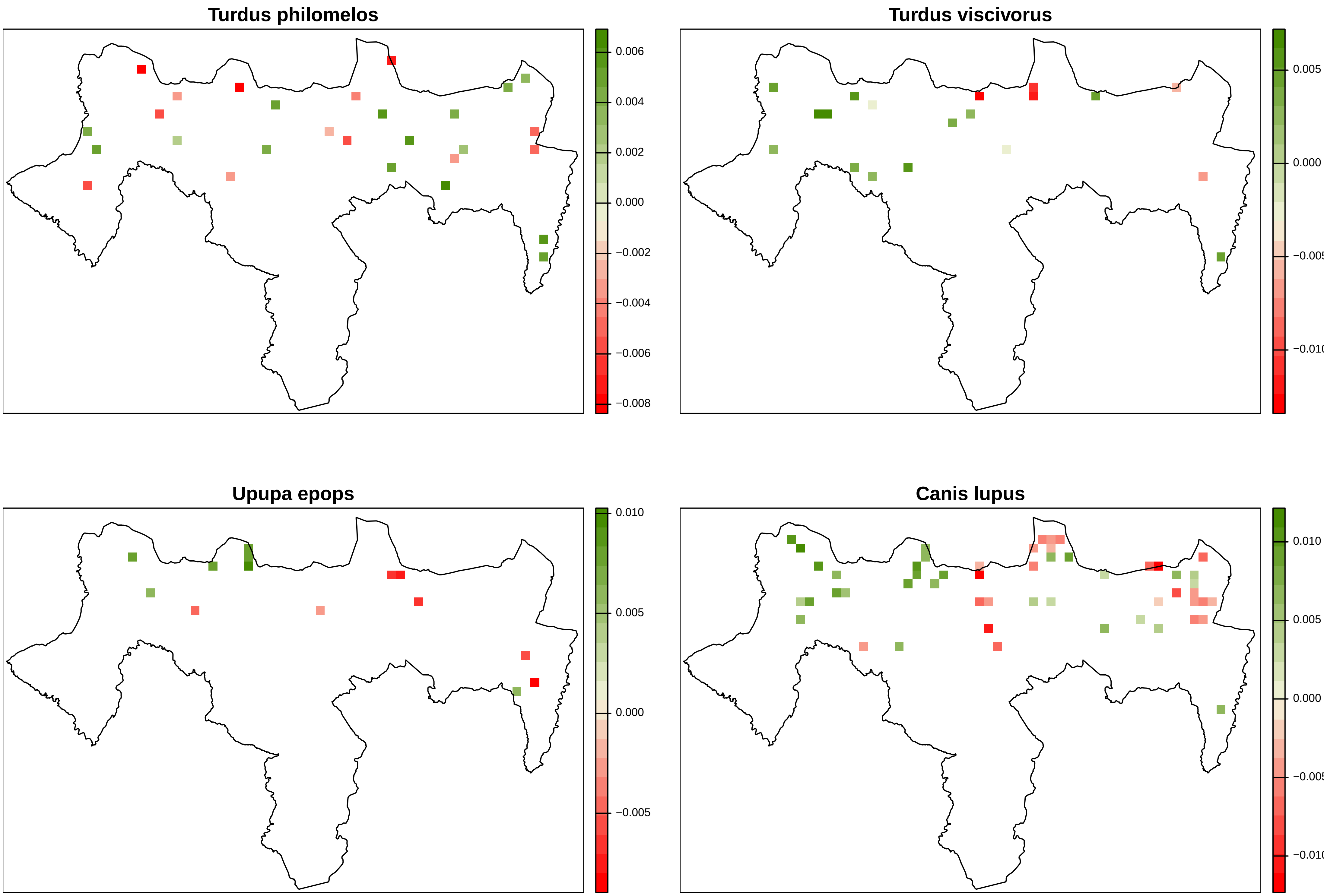
Turdus philomelos
0.006
0.004
0.002
0.000
−0.002
−0.004
−0.006
−0.008
Turdus viscivorus
0.005
0.000
Upupa epops
0.010
0.005
0.000
−0.005
Canis lupus
0.010
0.005
0.000

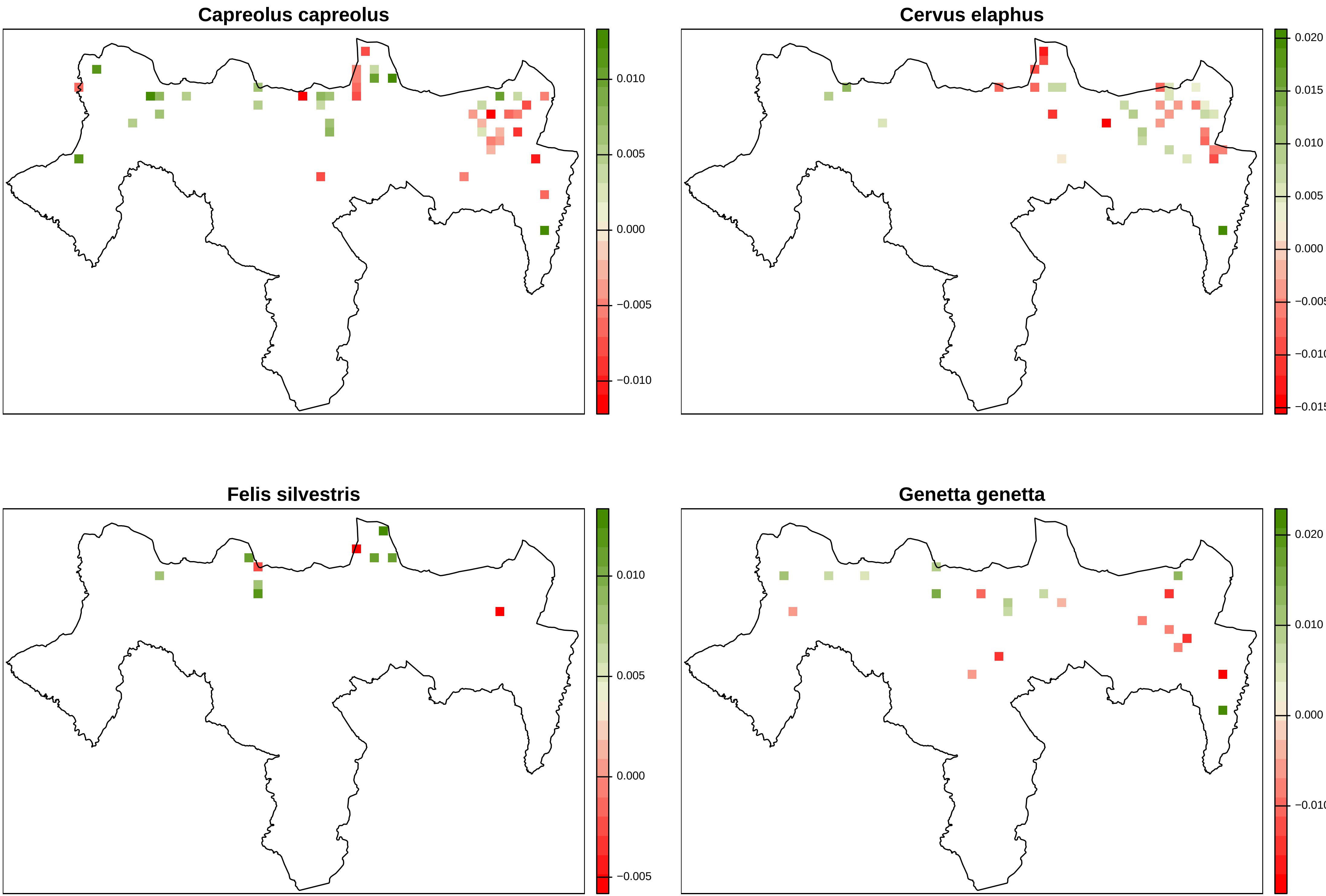

Capreolus capreolus
0.010
0.005
0.000
−0.005
−0.010
Cervus elaphus
0.020
0.015
0.010
0.005
0.000
Felis silvestris
0.010
0.005
0.000
−0.005
Genetta genetta
0.020
0.010
0.000

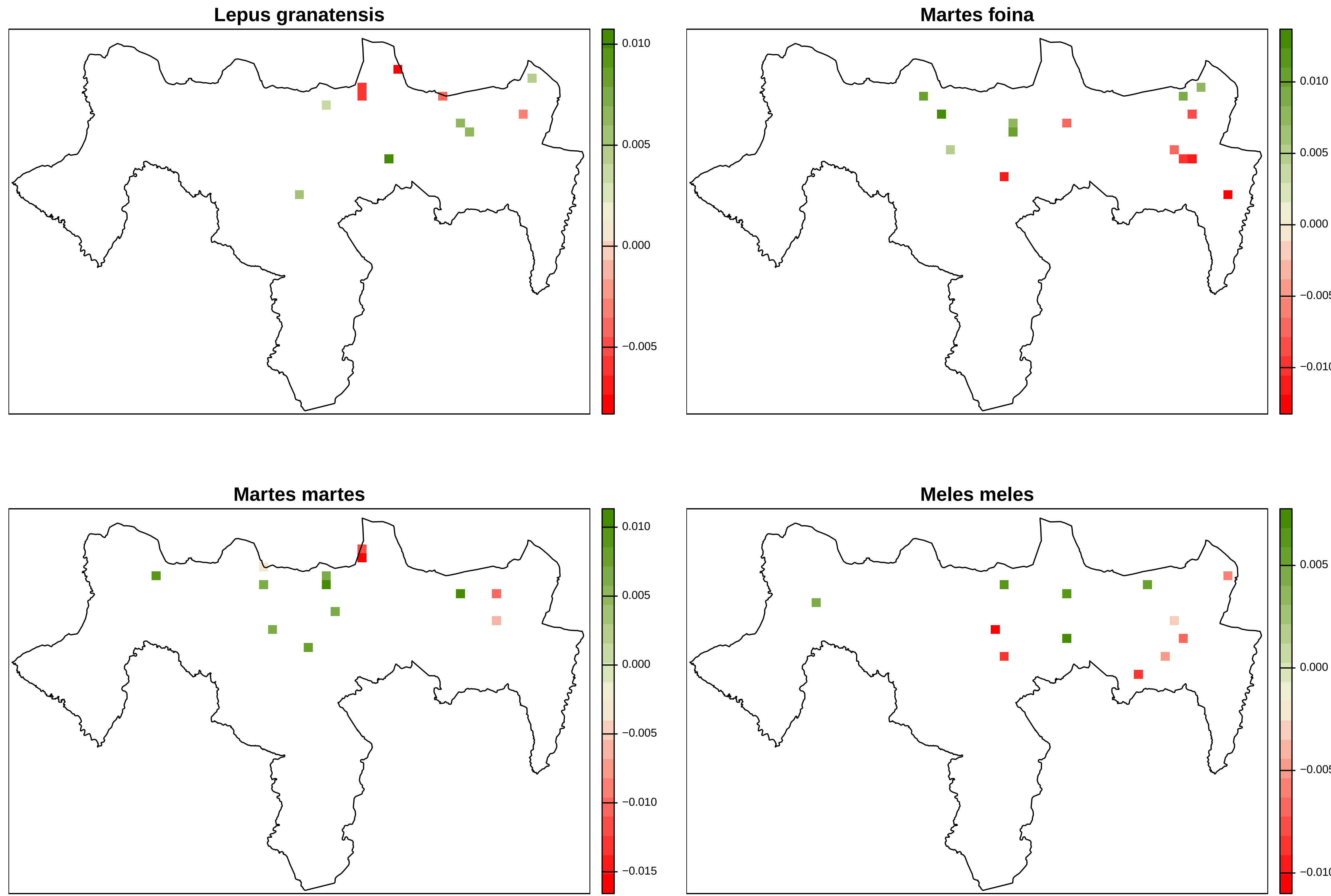
Lepus granatensis
0.010
0.005
0.000
−0.005
Martes foina
0.010
0.005
0.000
Martes martes
0.010
0.005
0.000
−0.005
−0.010
−0.015
Meles meles
0.005
0.000

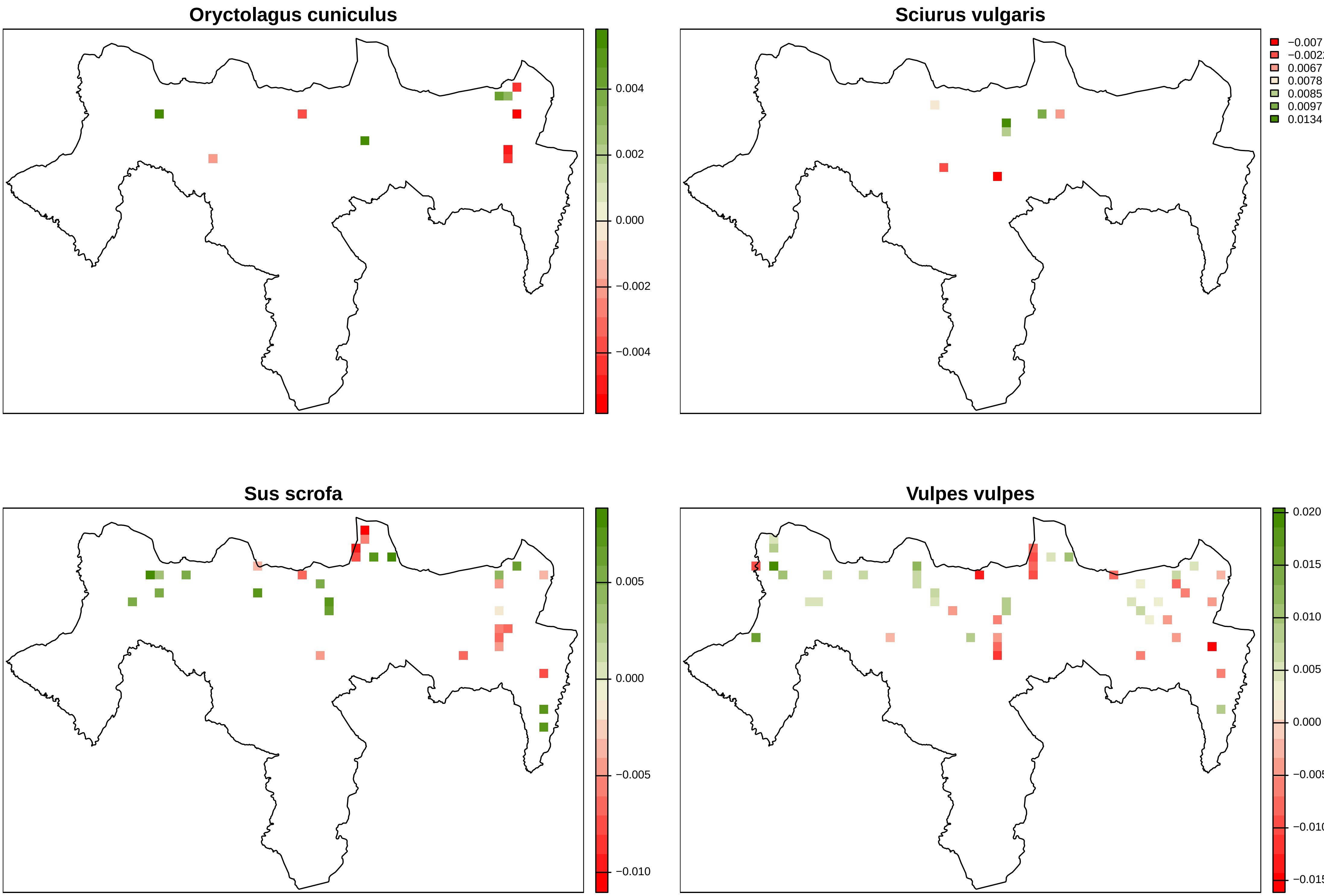

Oryctolagus cuniculus
0.004
0.002
0.000
−0.002
−0.004
Sciurus vulgaris
−0.007
0.0067
0.0078
0.0085
0.0097
0.0134
Sus scrofa
0.005
0.000
−0.005
−0.010
Vulpes vulpes
0.020
0.015
0.010
0.005
0.000

Achillea millefolium

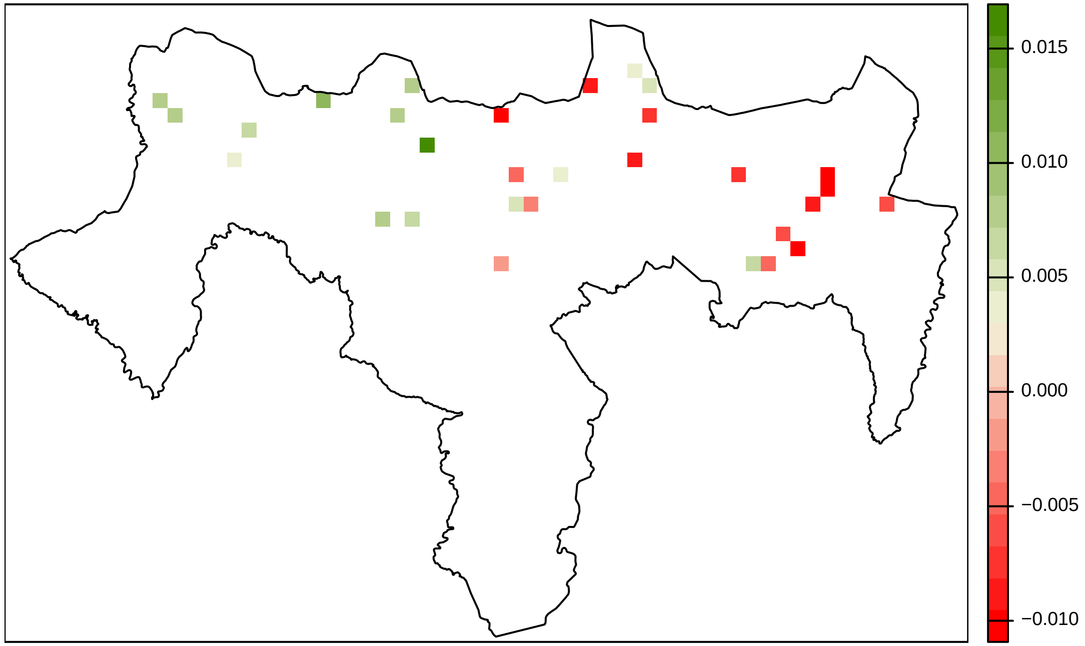

Adenocarpus complicatus

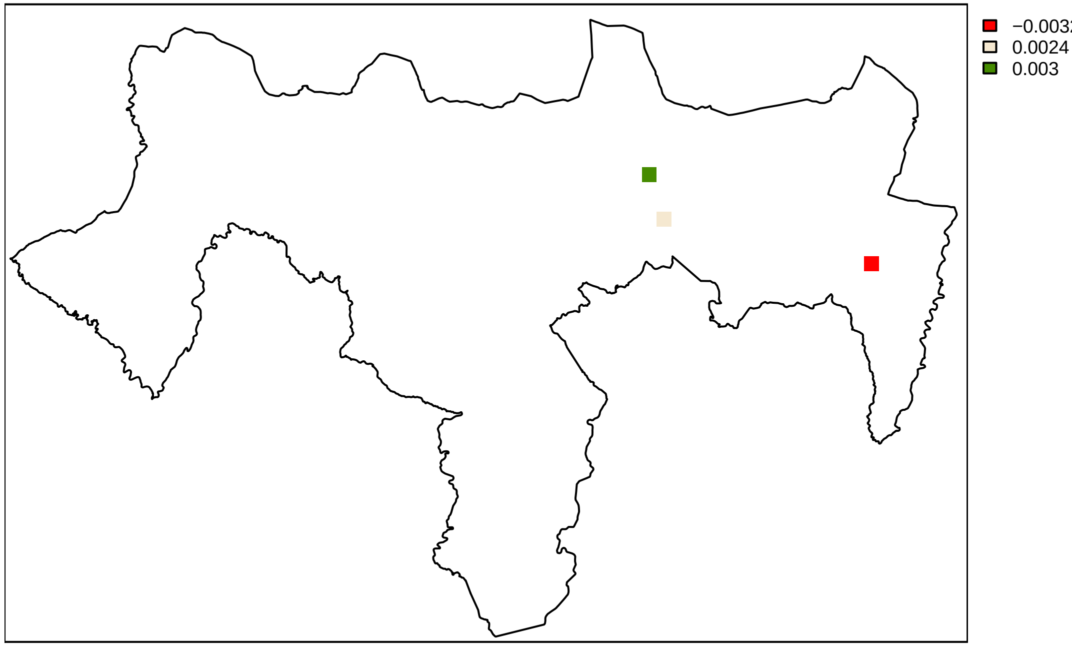

Agrimonia eupatoria

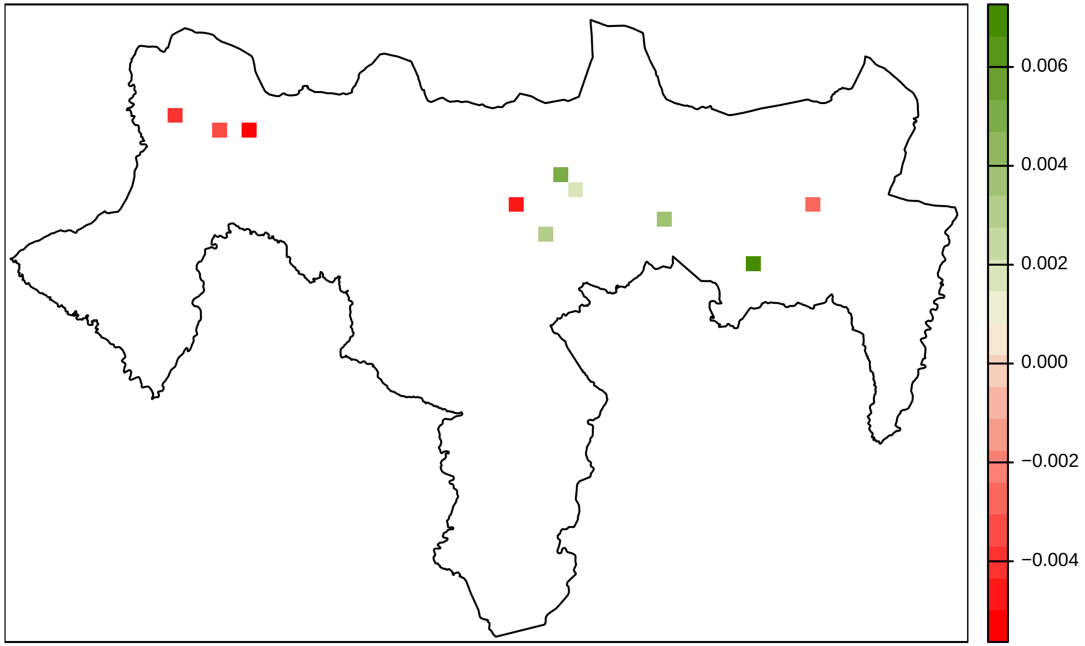

Agrostis castellana

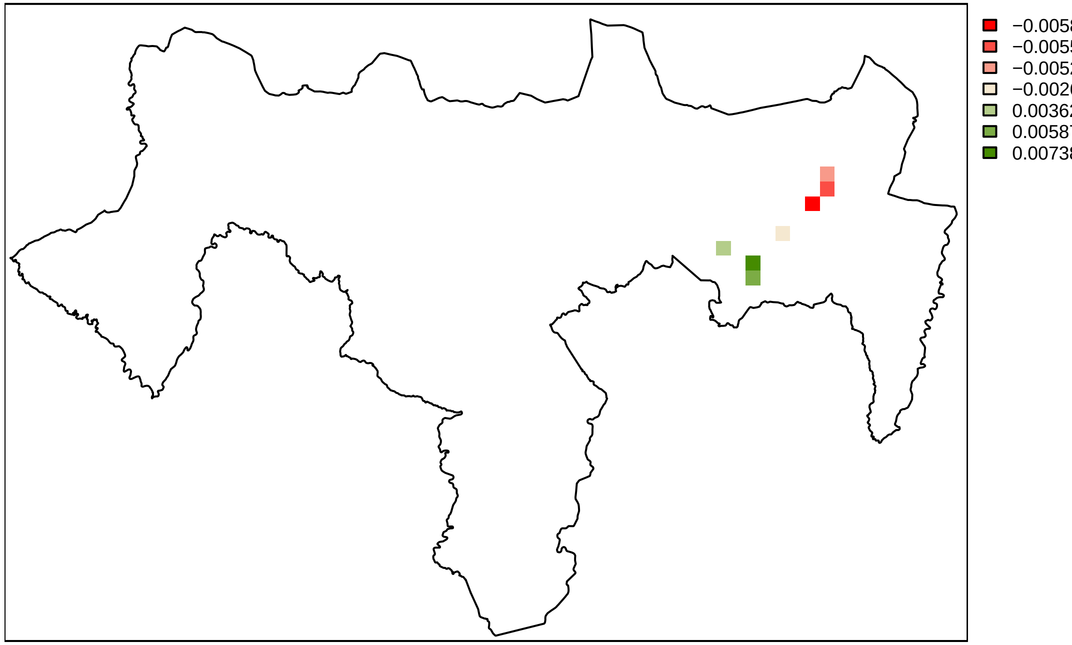

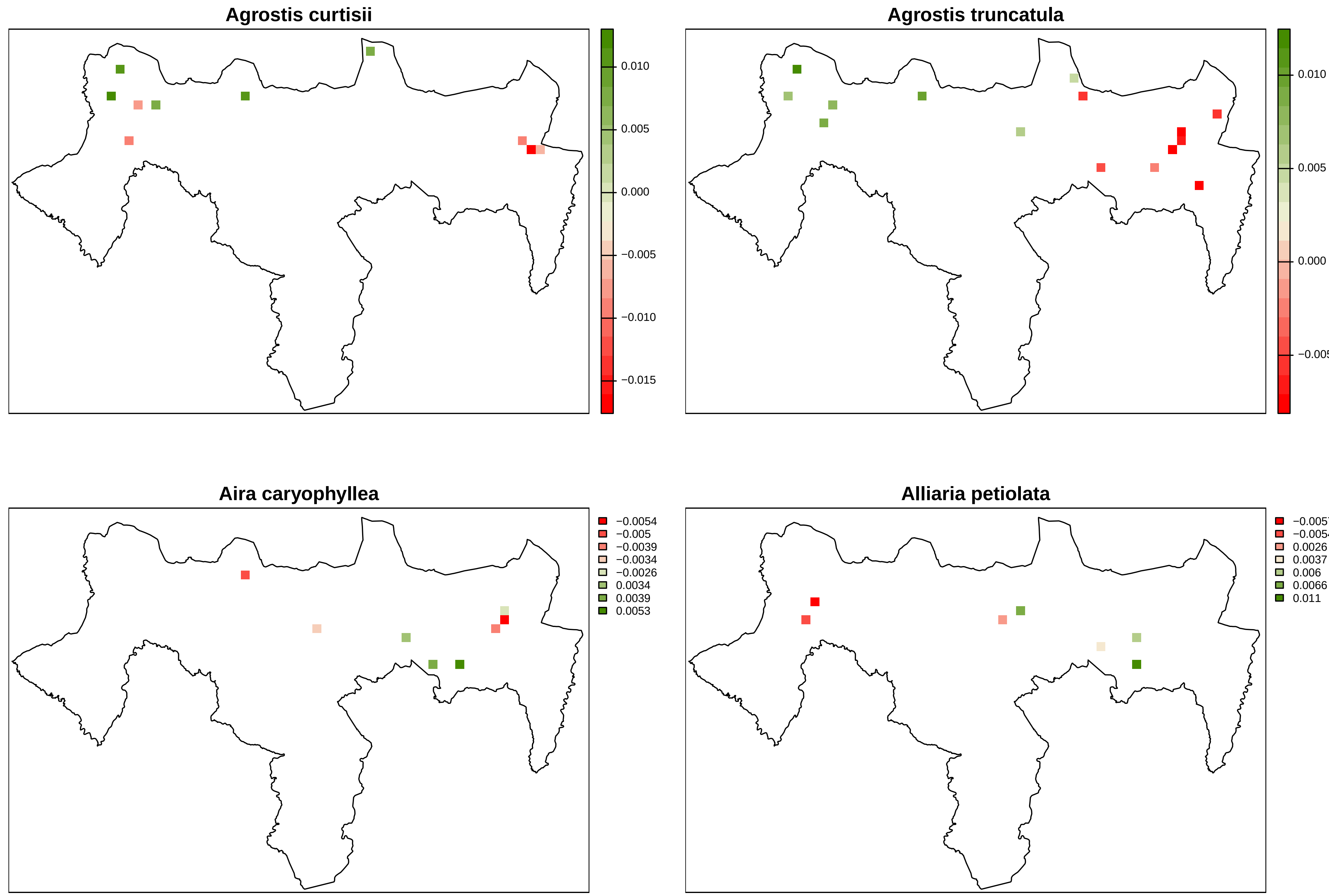
Agrostis curtisii
0.010
0.005
0.000
−0.005
−0.010
−0.015
Agrostis truncatula
0.010
0.005
0.000
Aira caryophyllea
−0.0054
−0.005
−0.0039
−0.0034
−0.0026
0.0034
0.0039
0.0053
Alliaria petiolata
0.0026
0.0037
0.006
0.0066
0.011

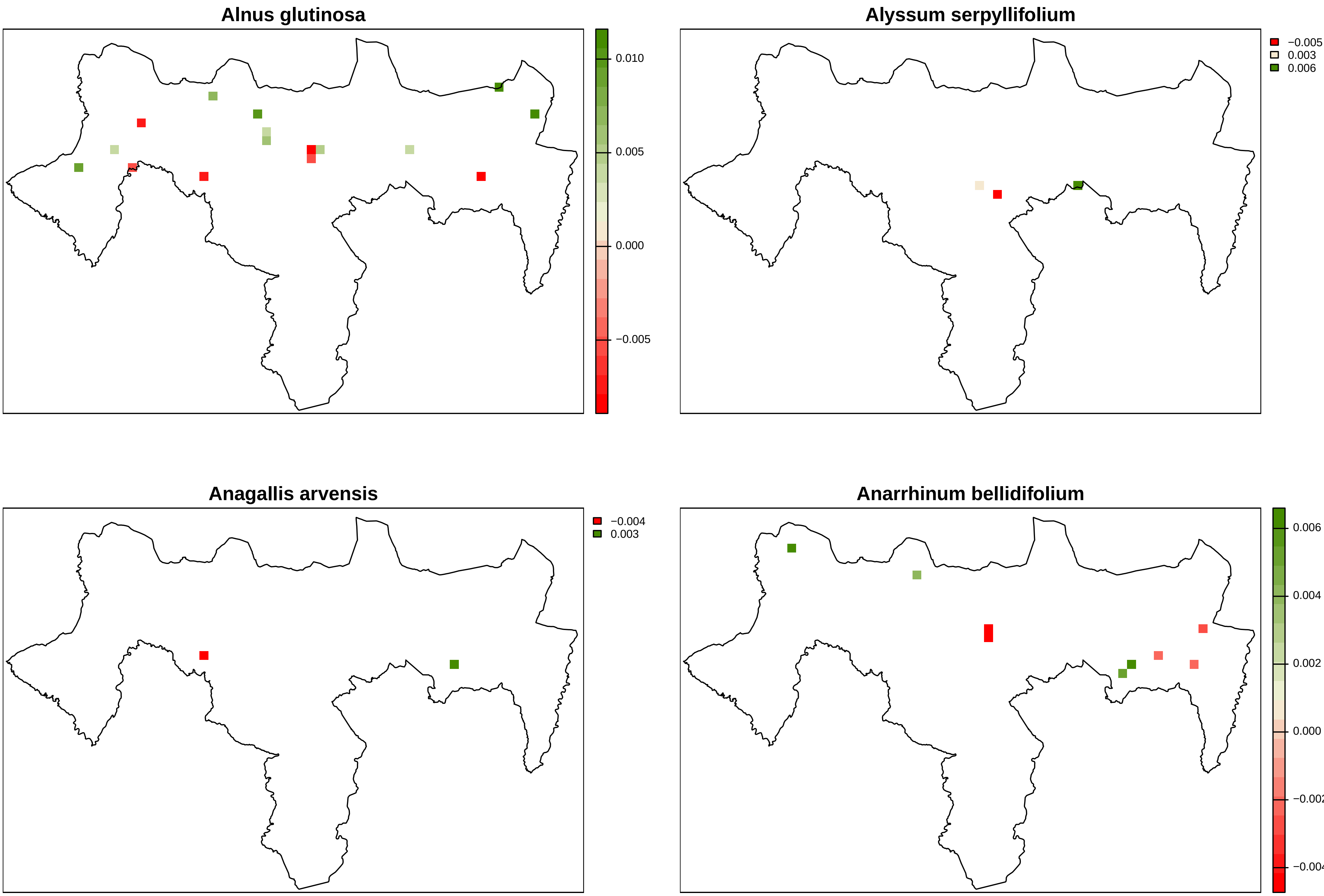
Alnus glutinosa
0.010
0.005
0.000
−0.005
Alyssum serpyllifolium
−0.005
0.003
0.006
Anagallis arvensis
−0.004
0.003
Anarrhinum bellidifolium
0.006
0.004
0.002
0.000

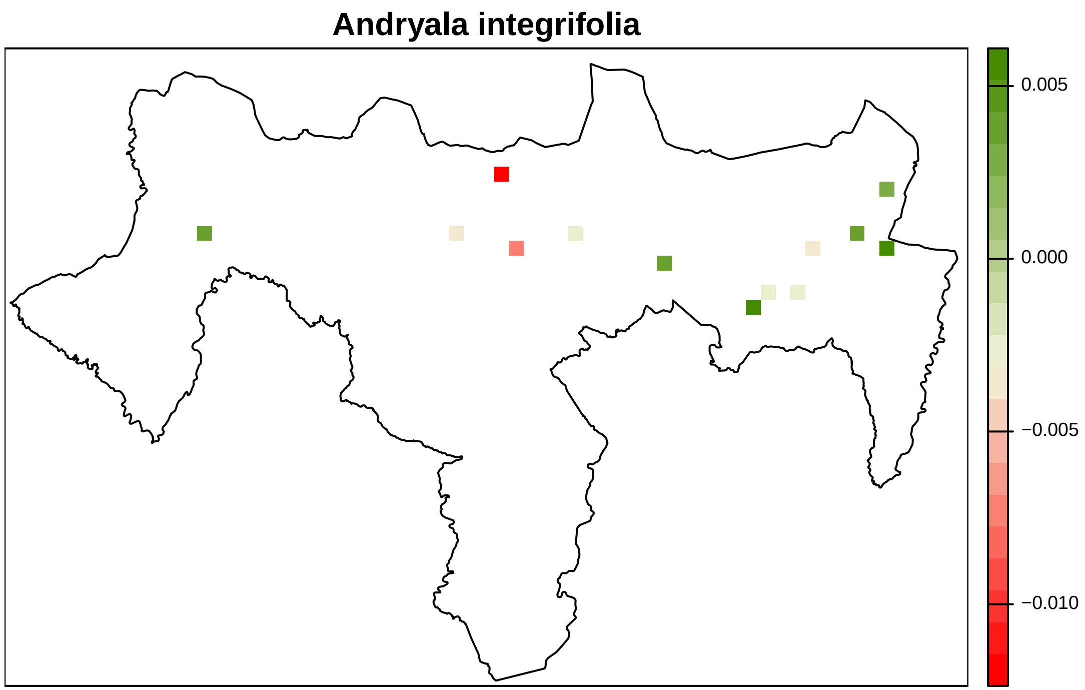
Andryala integrifolia
0.005
0.000
−0.005
−0.010

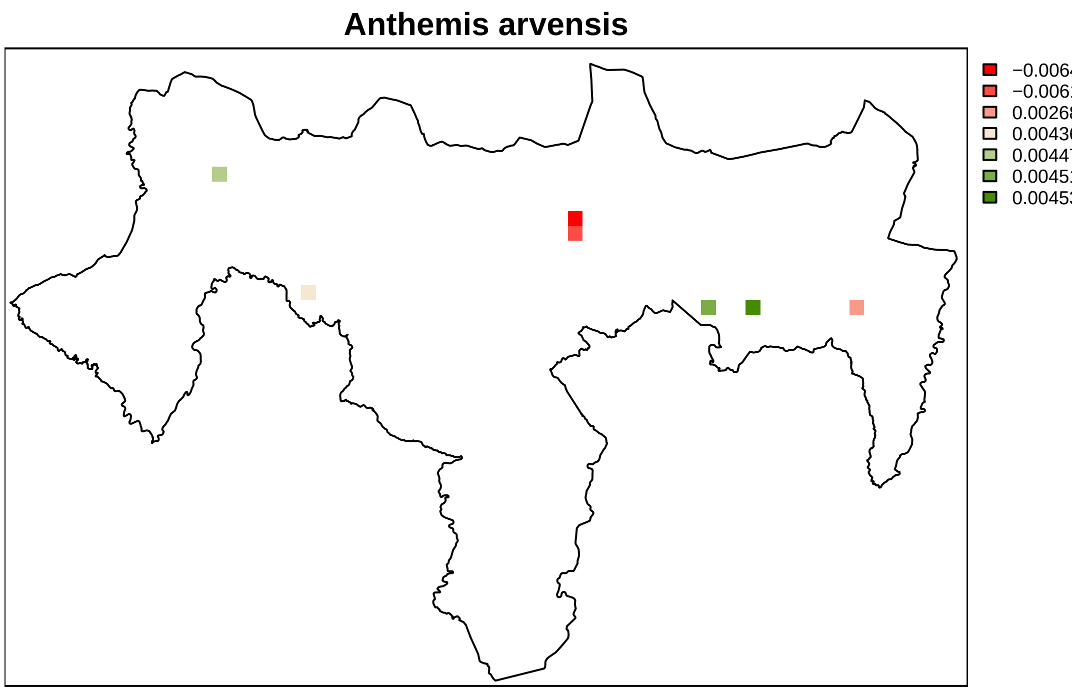
Anthemis arvensis
−0.006
−0.006
0.0026
0.0043
0.0044
0.0045
0.0045

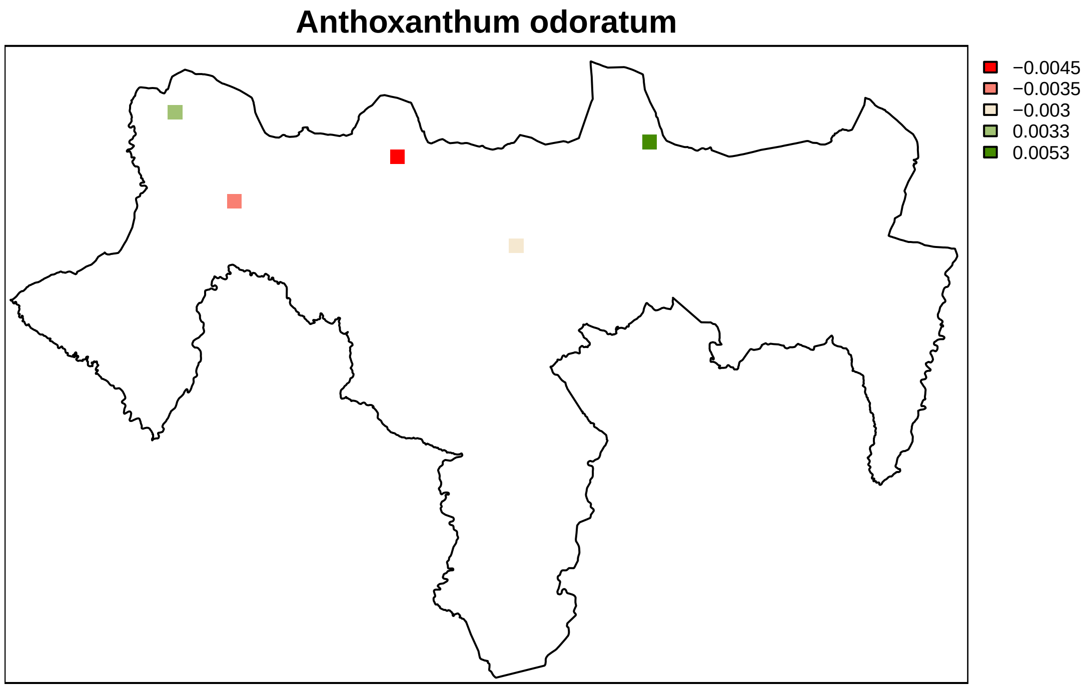
Anthoxanthum odoratum
−0.0045
−0.0035
−0.003
0.0033
0.0053

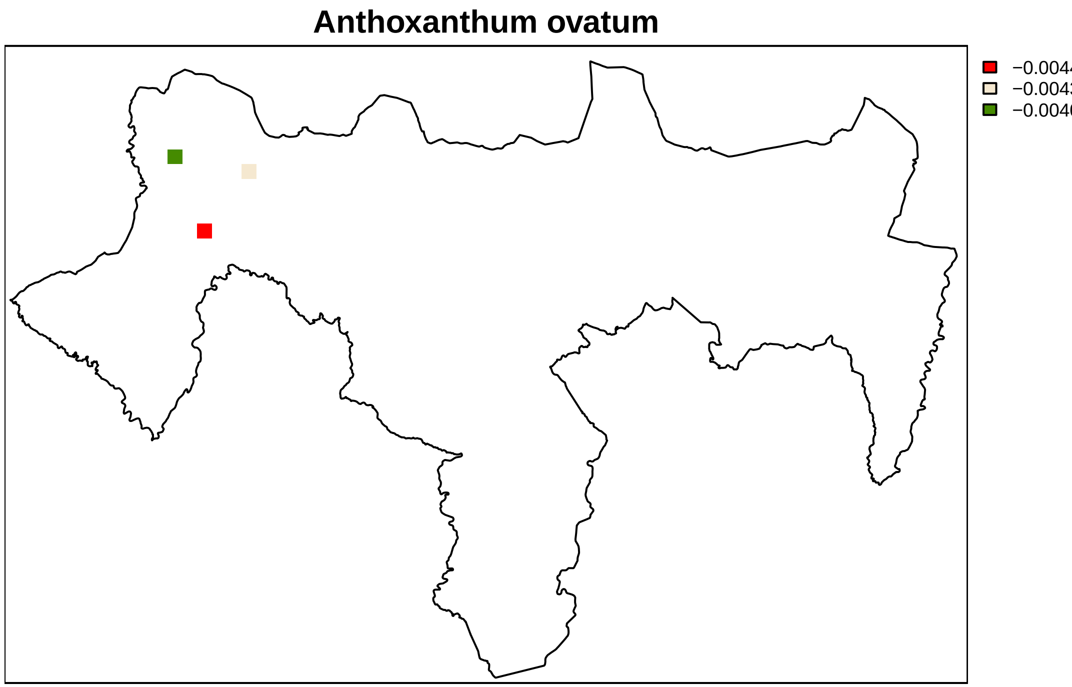
Anthoxanthum ovatum
−0.004
−0.004
−0.004

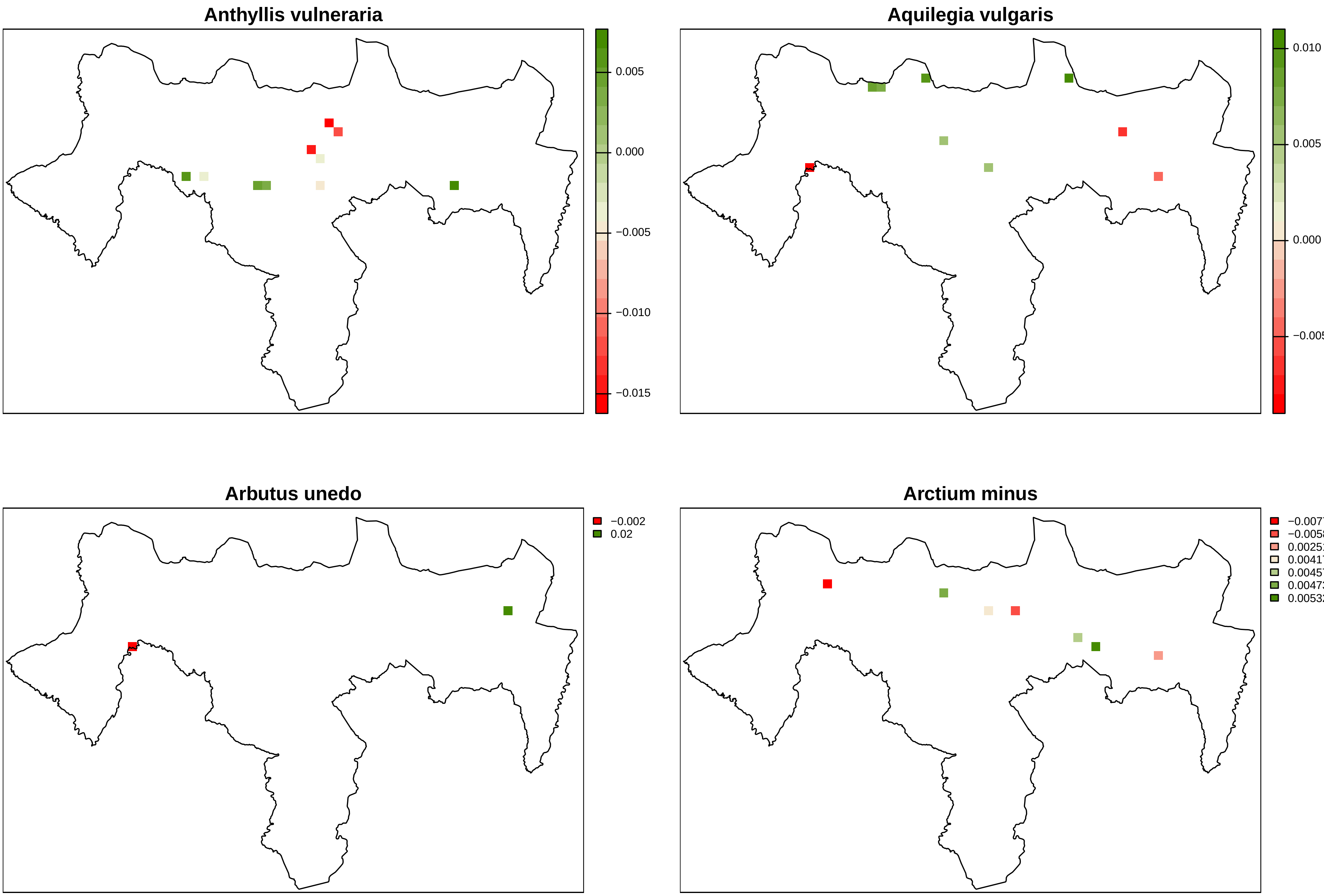

Anthyllis vulneraria
0.005
0.000
−0.005
−0.010
−0.015
Aquilegia vulgaris
0.010
0.005
0.000
Arbutus unedo
−0.002
0.02
Arctium minus

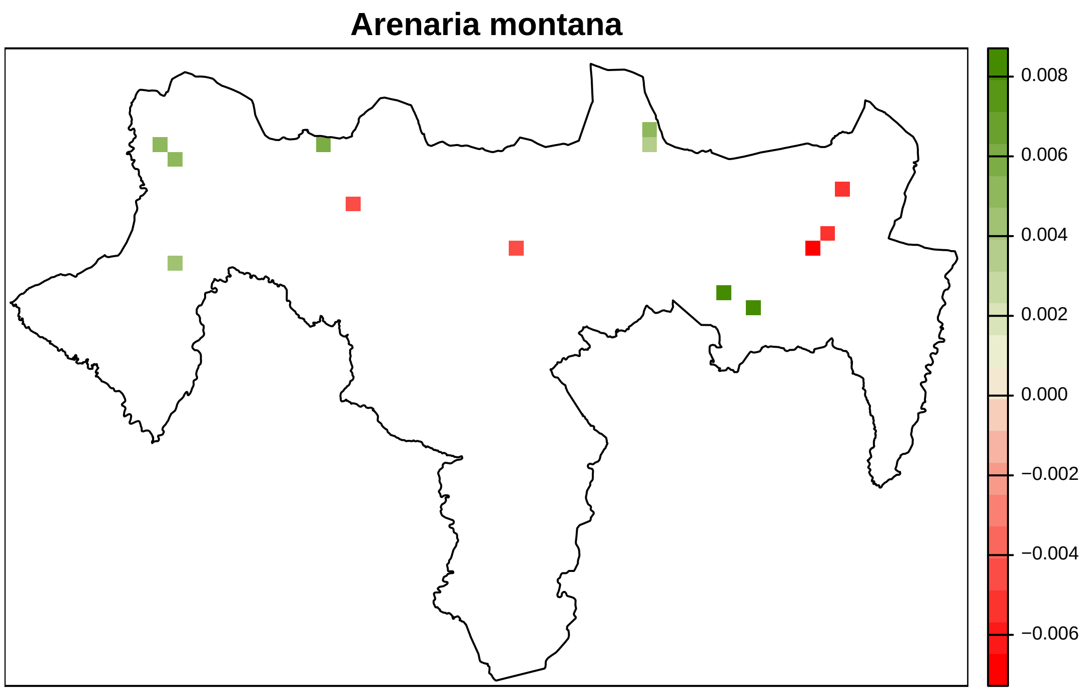
Arenaria montana
0.008
0.006
0.004
0.002
0.000
−0.002
−0.004
−0.006

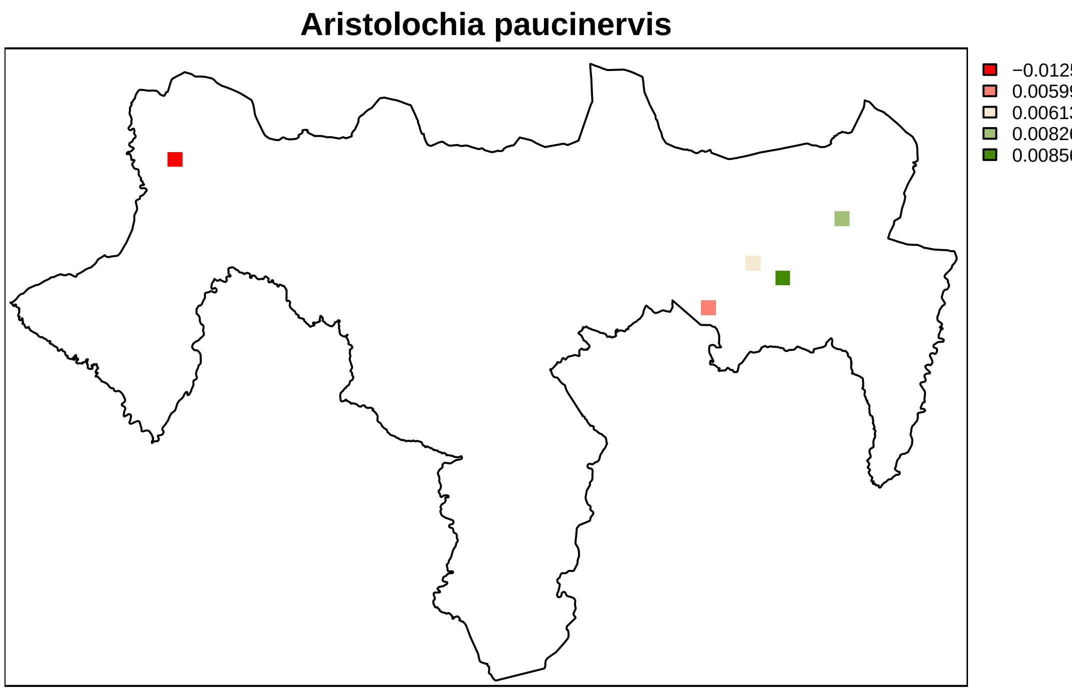
Aristolochia paucinervis
−0.012
0.0059
0.0061
0.0082
0.0085

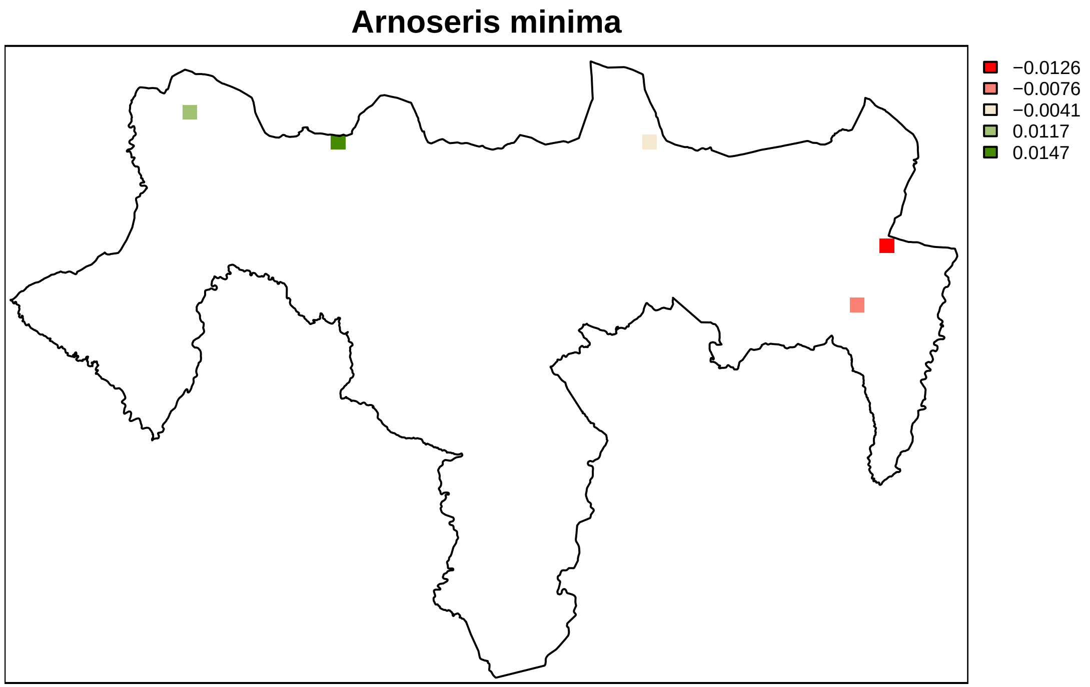
Arnoseris minima
−0.0126
−0.0076
−0.0041
0.0117
0.0147

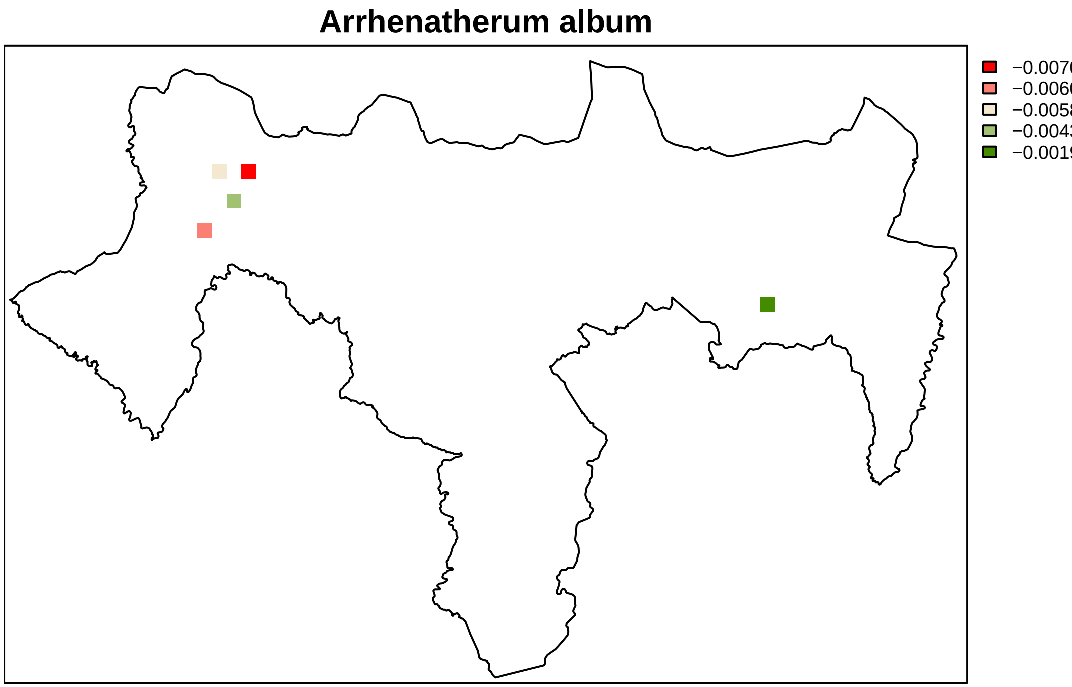
Arrhenatherum album
−0.007
−0.006
−0.005
−0.004
−0.001

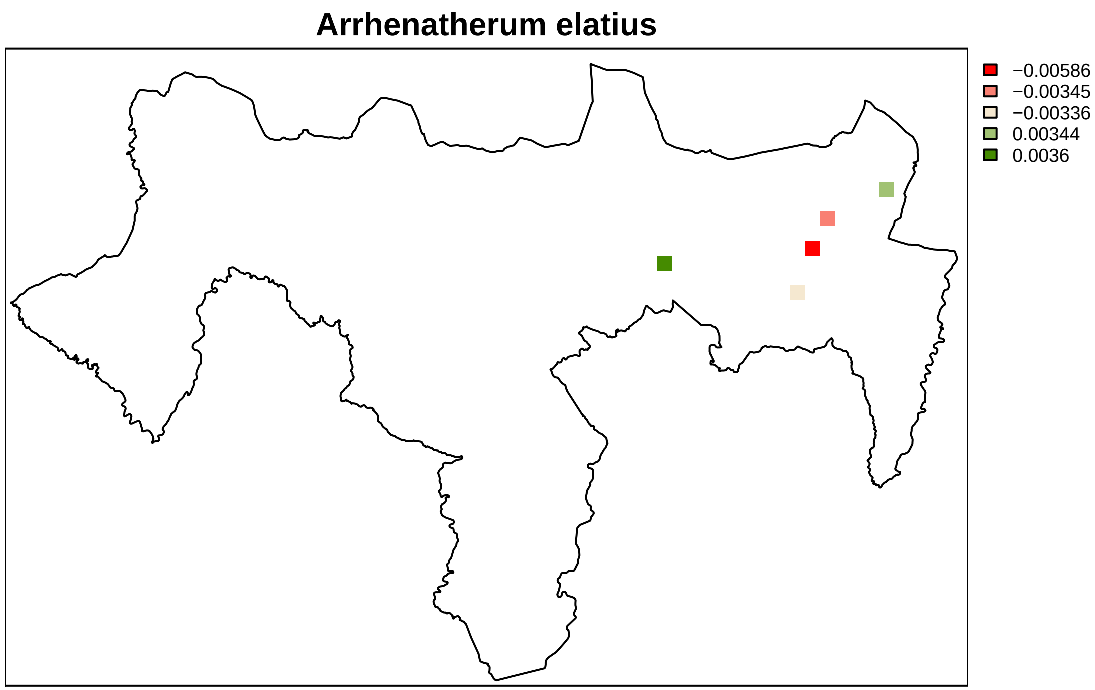
Arrhenatherum elatius
−0.00586
−0.00345
−0.00336
0.00344
0.0036

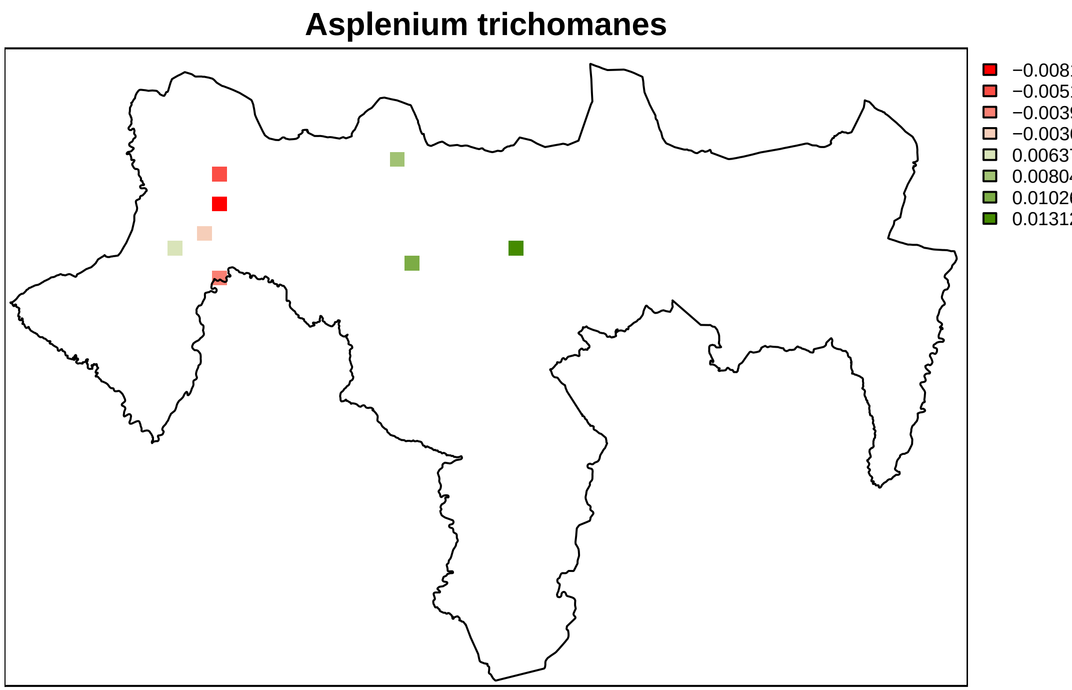
Asplenium trichomanes
−0.008
−0.005
−0.003
−0.003
0.0063
0.0080
0.0102
0.0131

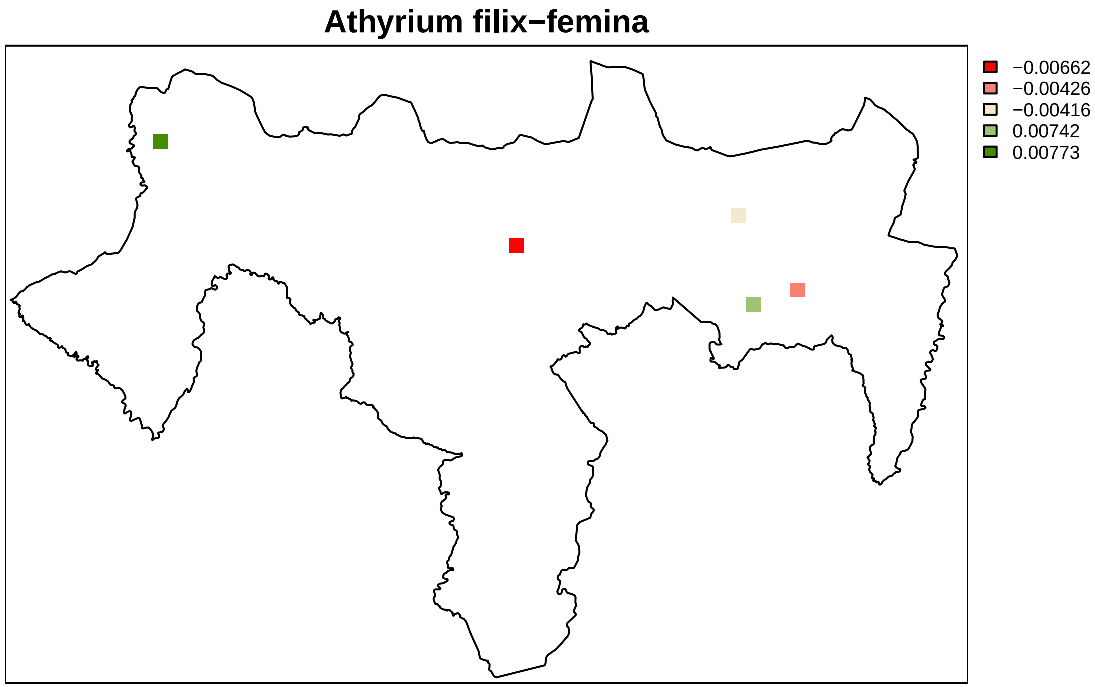
Athyrium filix−femina
−0.00662
−0.00426
−0.00416
0.00742
0.00773

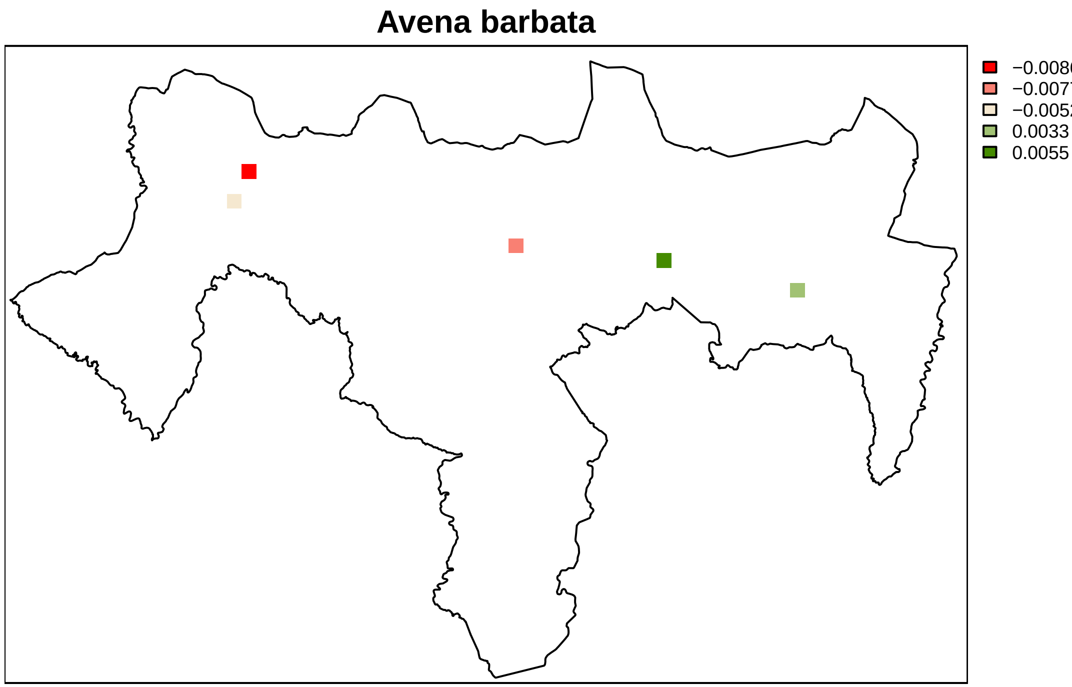
Avena barbata
−0.008
−0.007
−0.005
0.0033
0.0055

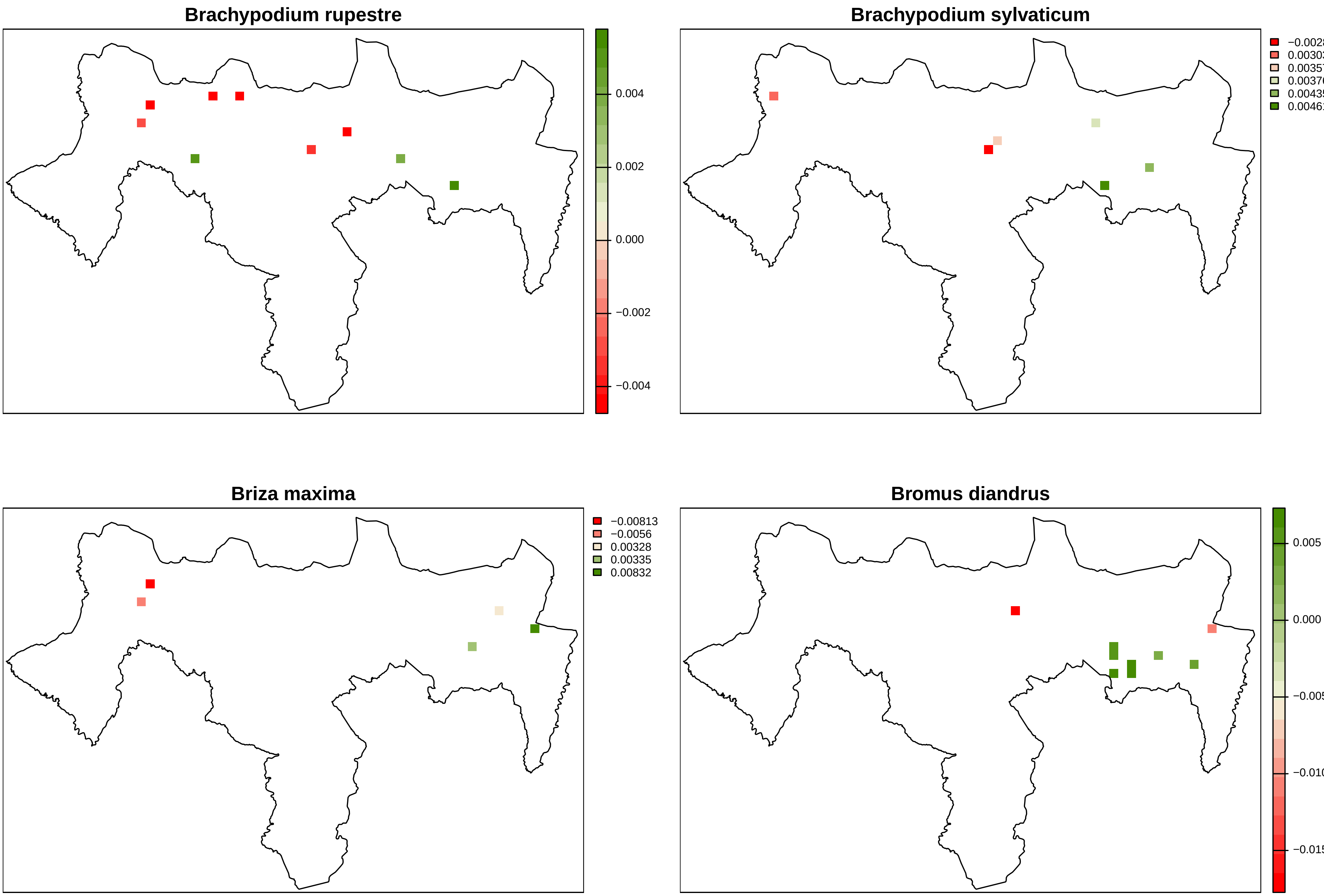
Brachypodium rupestre
0.004
0.002
0.000
−0.002
−0.004
Brachypodium sylvaticum
Briza maxima
−0.00813
−0.0056
0.00328
0.00335
0.00832
Bromus diandrus
0.005
0.000

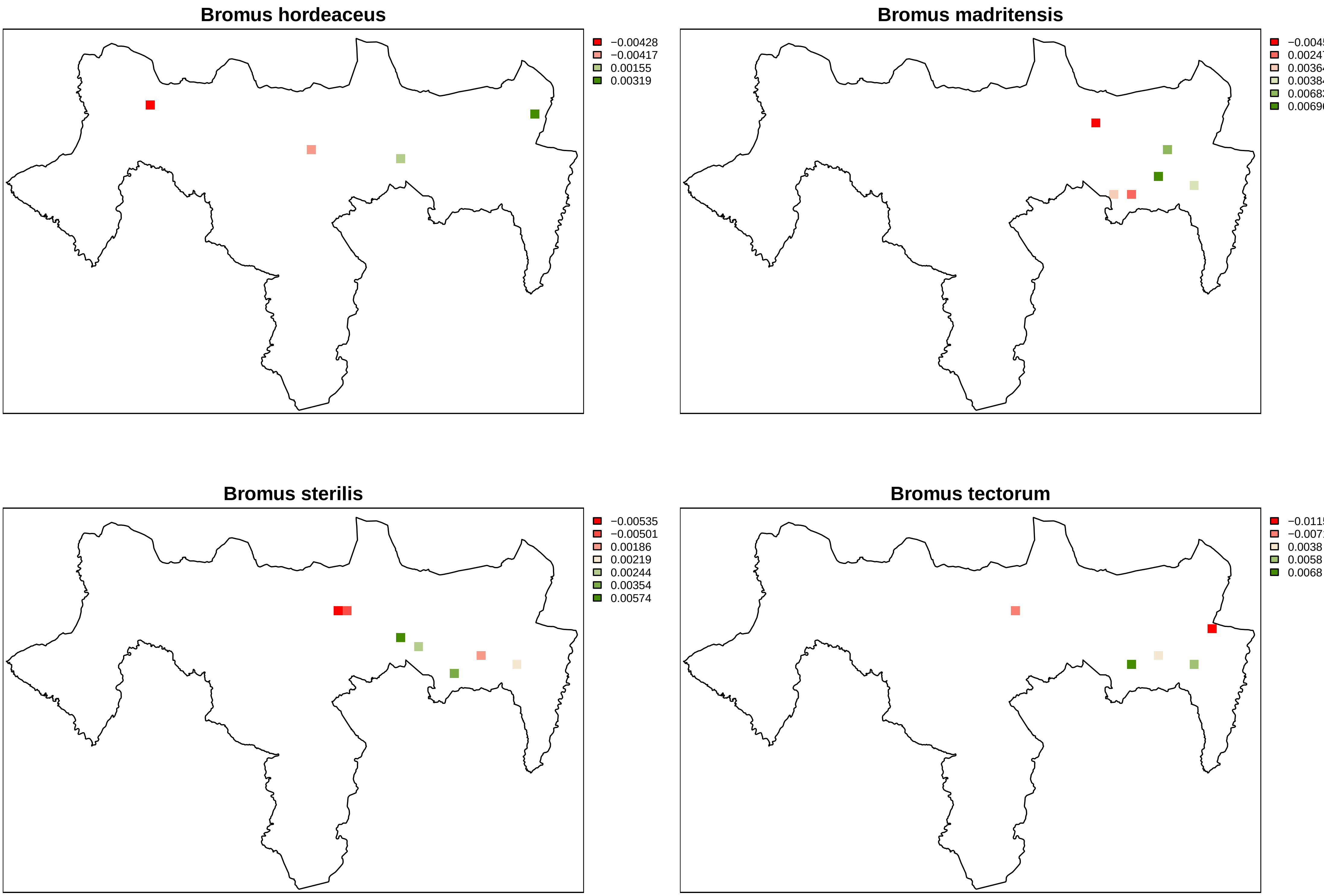

Bromus hordeaceus
−0.00428
−0.00417
0.00155
0.00319
Bromus madritensis
Bromus sterilis
−0.00535
−0.00501
0.00186
0.00219
0.00244
0.00354
0.00574
Bromus tectorum

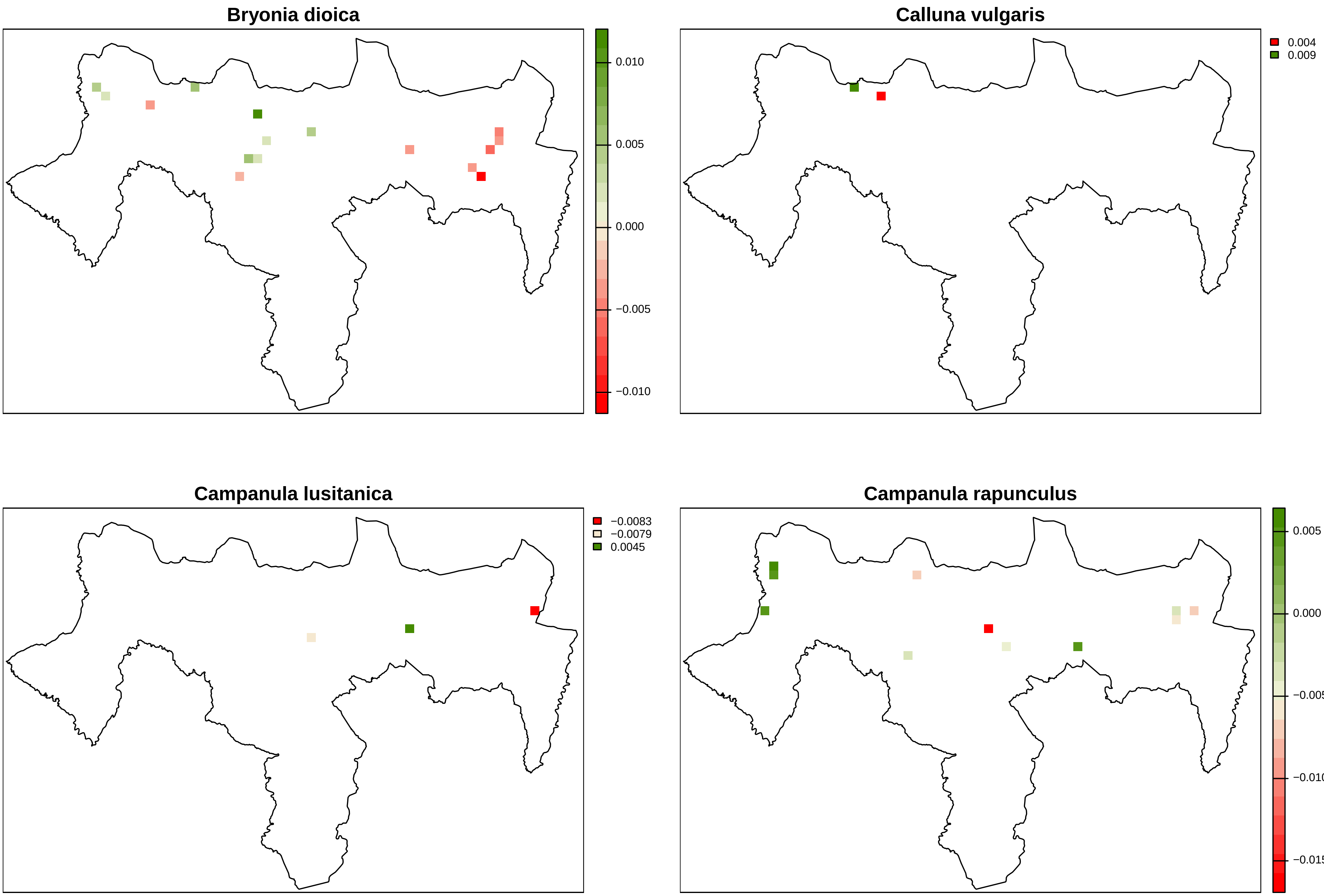
Bryonia dioica
0.010
0.005
0.000
−0.005
−0.010
Calluna vulgaris
0.004
0.009
Campanula lusitanica
−0.0083
−0.0079
0.0045
Campanula rapunculus
0.005
0.000

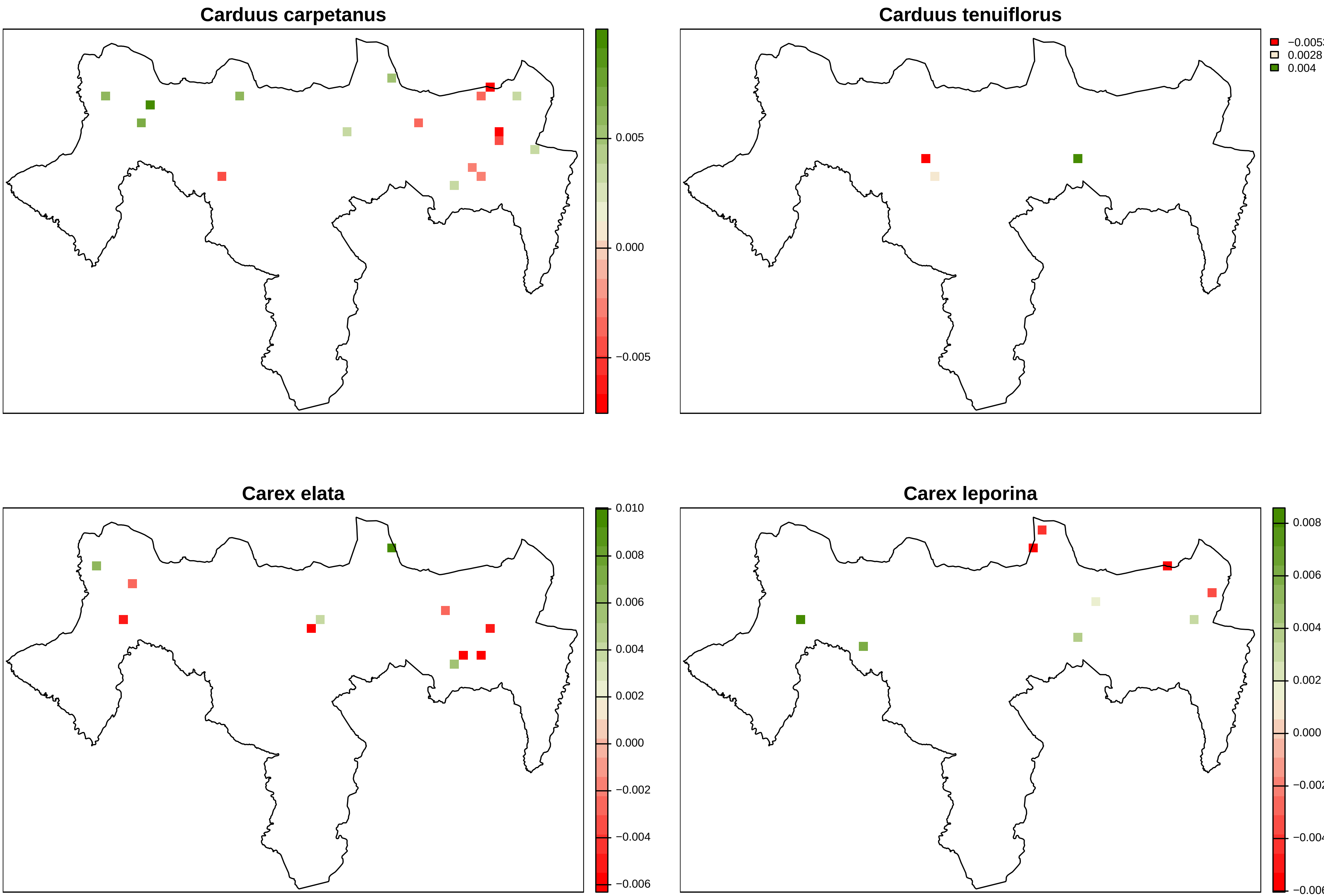
Carduus carpetanus
0.005
0.000
−0.005
Carduus tenuiflorus
0.0028
0.004
Carex elata
0.010
0.008
0.006
0.004
0.002
0.000
−0.002
−0.004
−0.006
Carex leporina
0.008
0.006
0.004
0.002
0.000

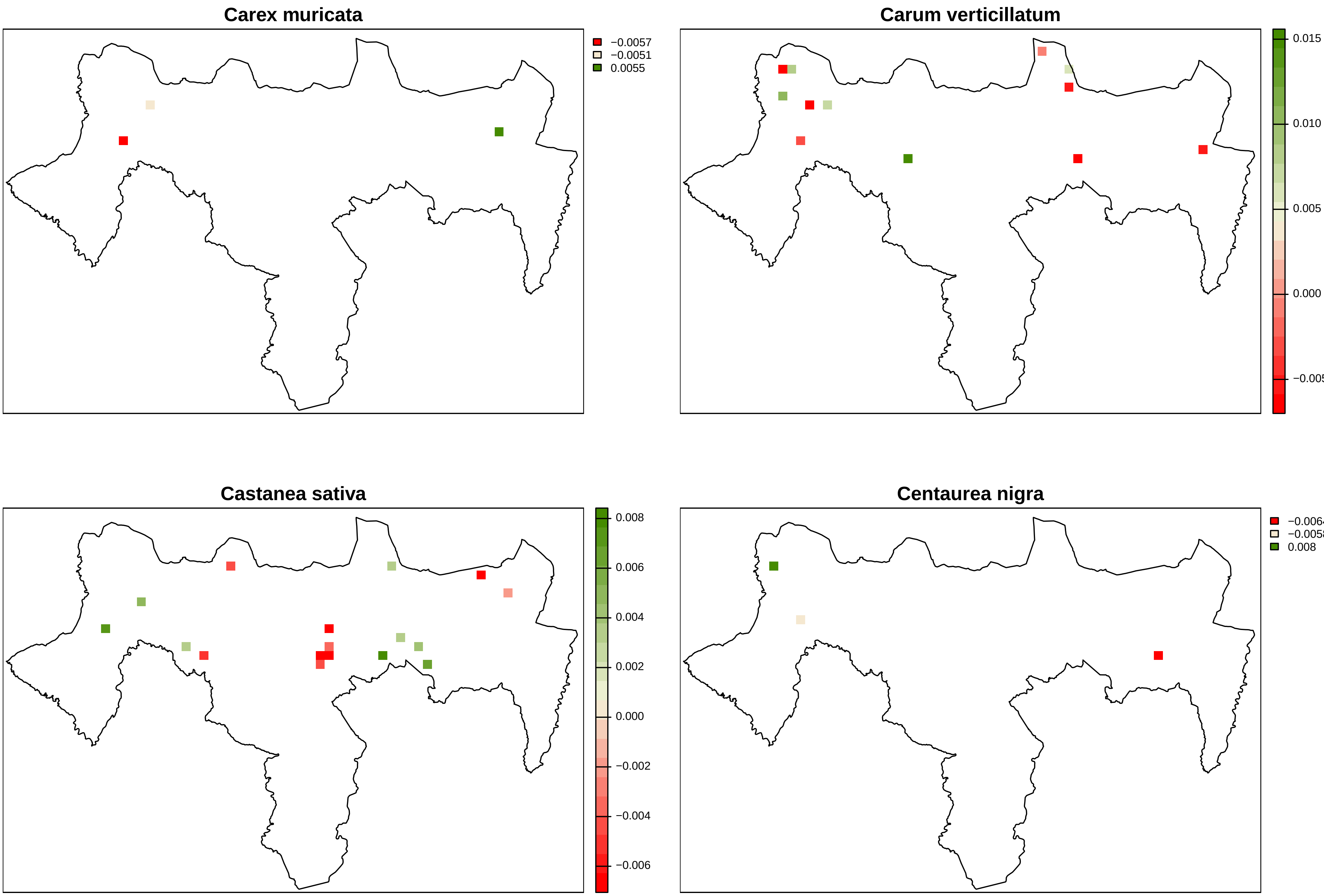
Carex muricata
−0.0057
−0.0051
0.0055
Carum verticillatum
0.015
0.010
0.005
0.000
Castanea sativa
0.008
0.006
0.004
0.002
0.000
−0.002
−0.004
−0.006
Centaurea nigra
0.008

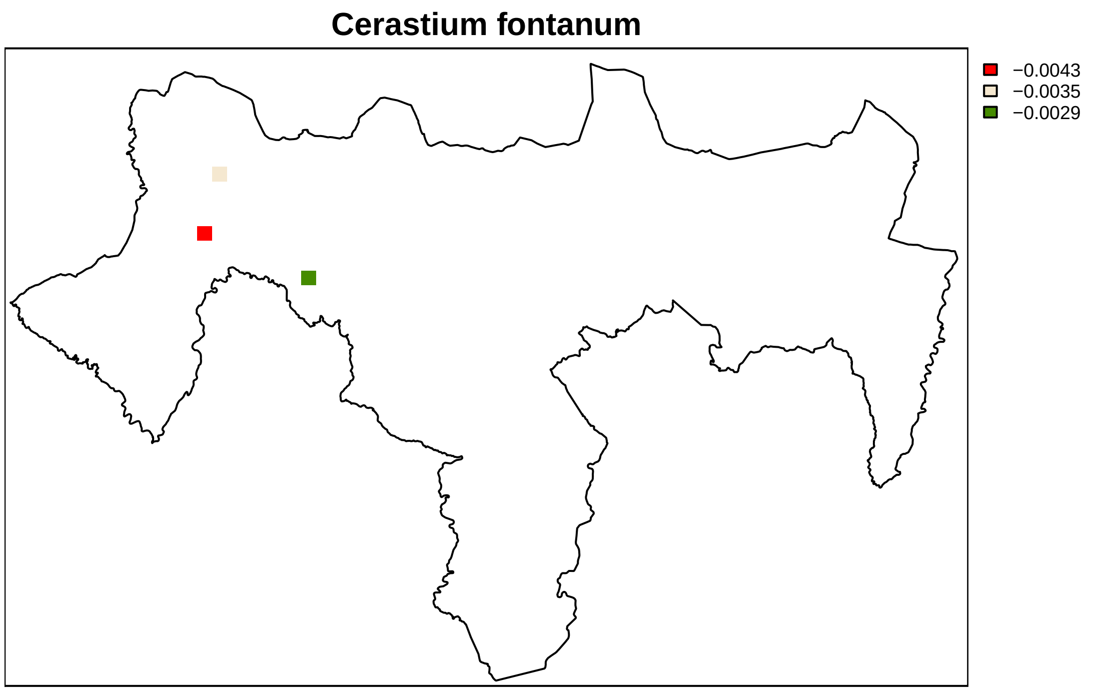
Cerastium fontanum
−0.0043
−0.0035
−0.0029

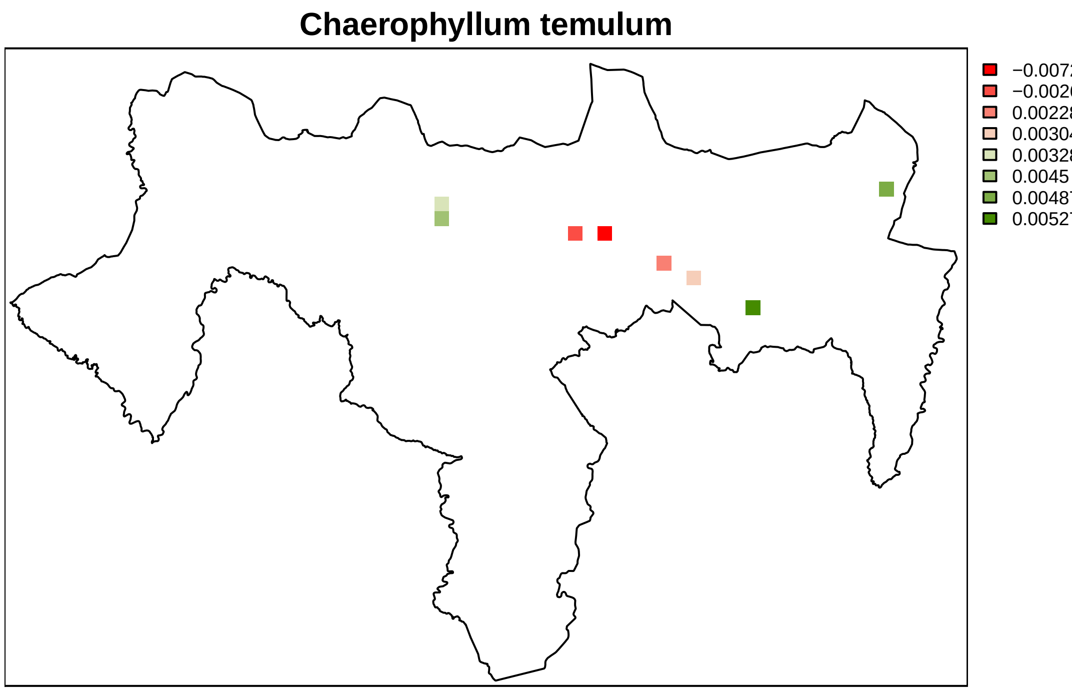
Chaerophyllum temulum

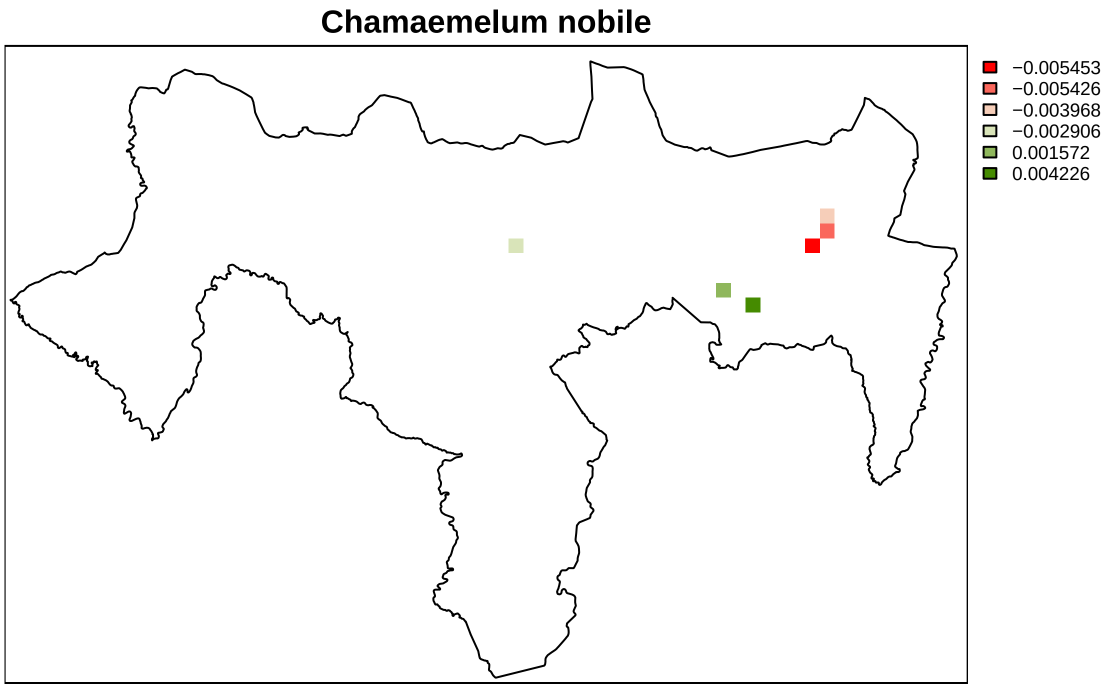
Chamaemelum nobile
−0.005453
−0.005426
−0.003968
−0.002906
0.001572
0.004226

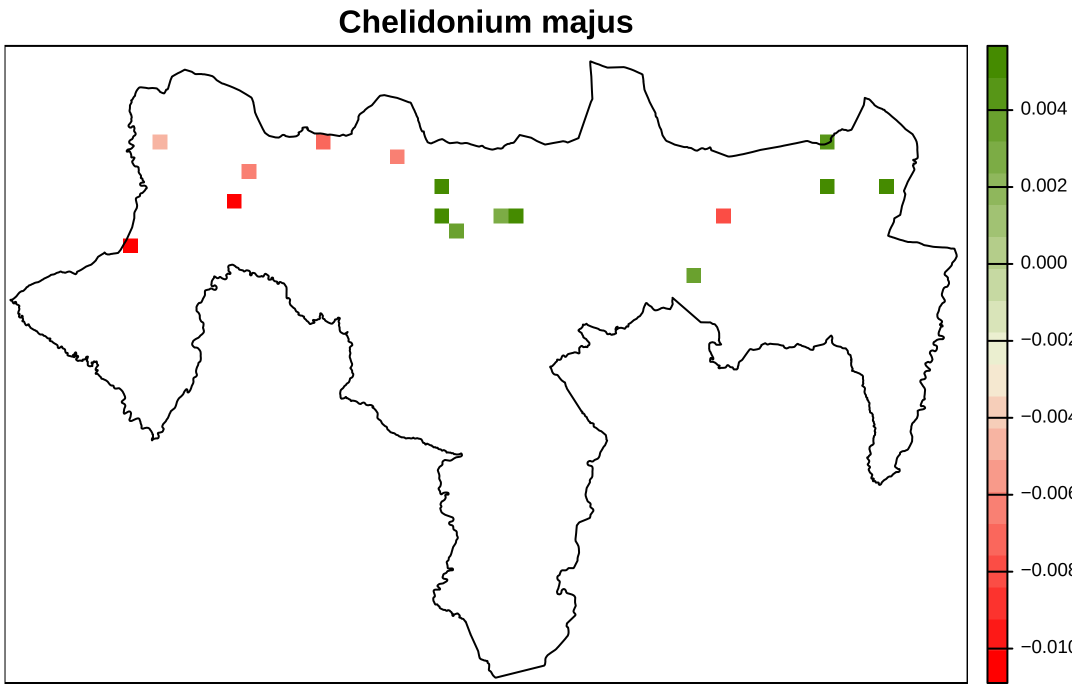
Chelidonium majus
0.004
0.002
0.000

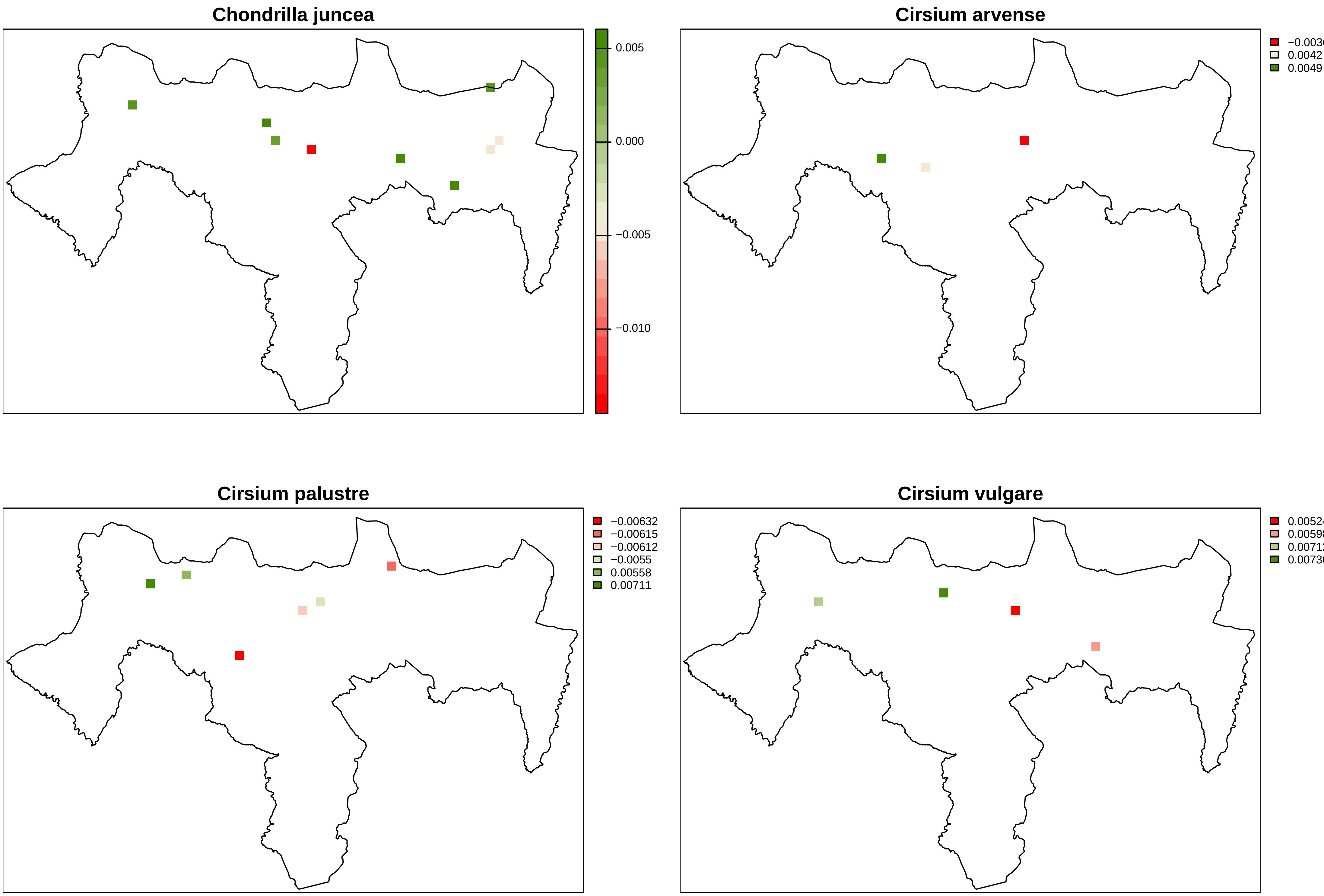
Chondrilla juncea
0.005
0.000
−0.005
−0.010
Cirsium arvense
−0.003
0.0042
0.0049
Cirsium palustre
−0.00632
−0.00615
−0.00612
−0.0055
0.00558
0.00711
Cirsium vulgare
0.0052
0.0059
0.0071
0.0073

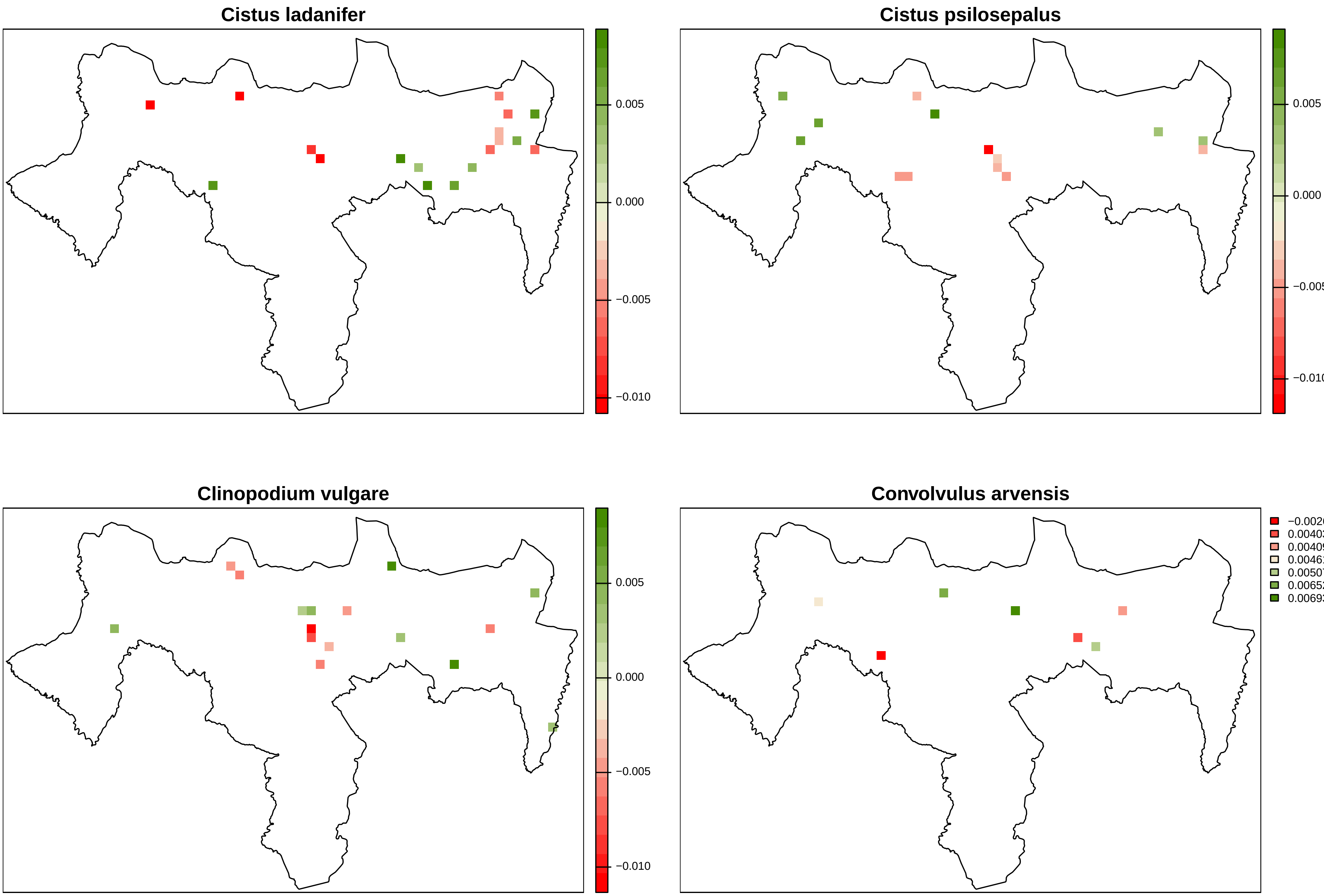
Cistus ladanifer
0.005
0.000
−0.005
−0.010
Cistus psilosepalus
0.005
0.000
Clinopodium vulgare
0.005
0.000
−0.005
−0.010
Convolvulus arvensis
−0.002
0.0040
0.0040
0.0046
0.0050
0.0065
0.0069

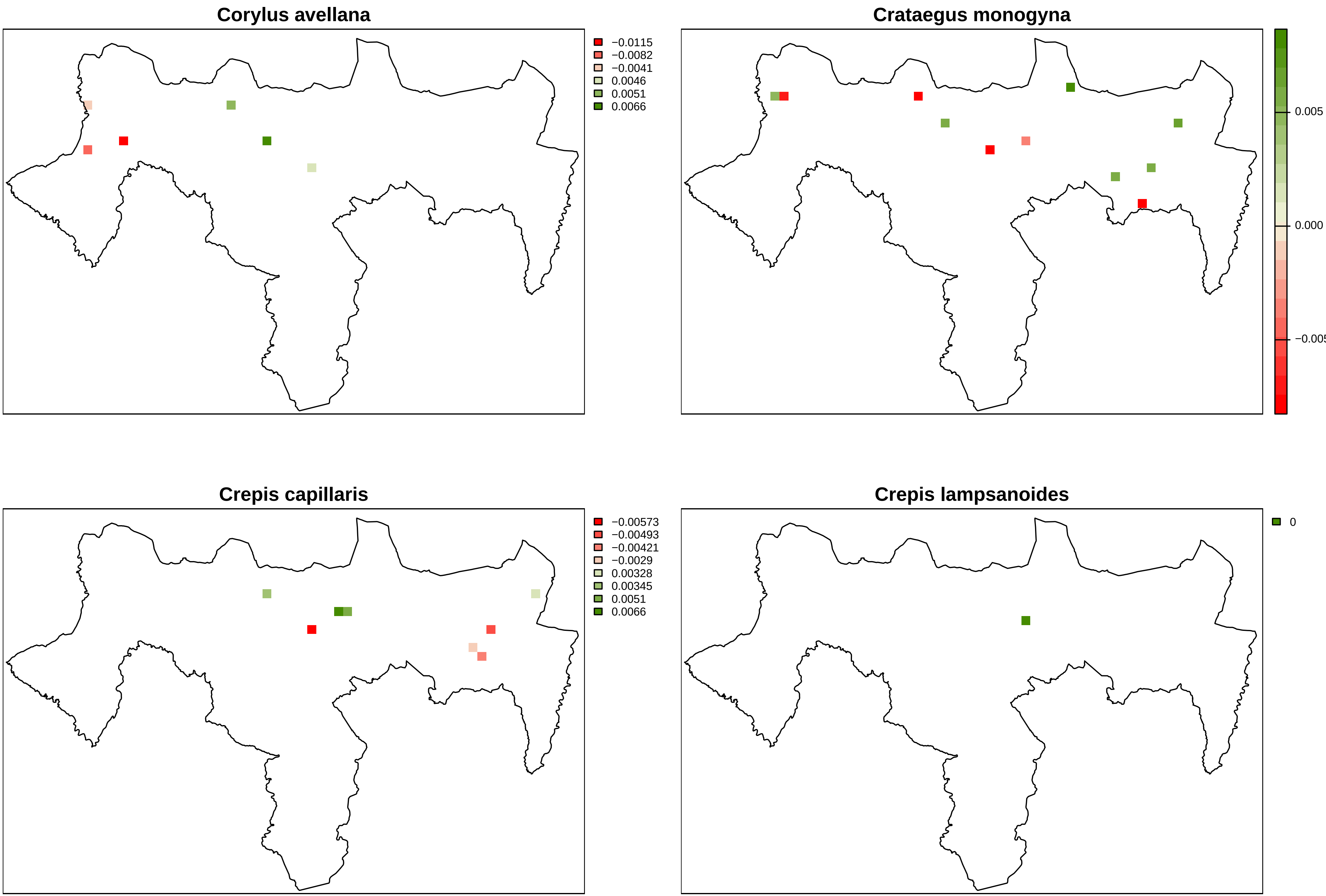
Corylus avellana
−0.0115
−0.0082
−0.0041
0.0046
0.0051
0.0066
Crataegus monogyna
0.005
0.000
Crepis capillaris
−0.00573
−0.00493
−0.00421
−0.0029
0.00328
0.00345
0.0051
0.0066
Crepis lampsanoides
0

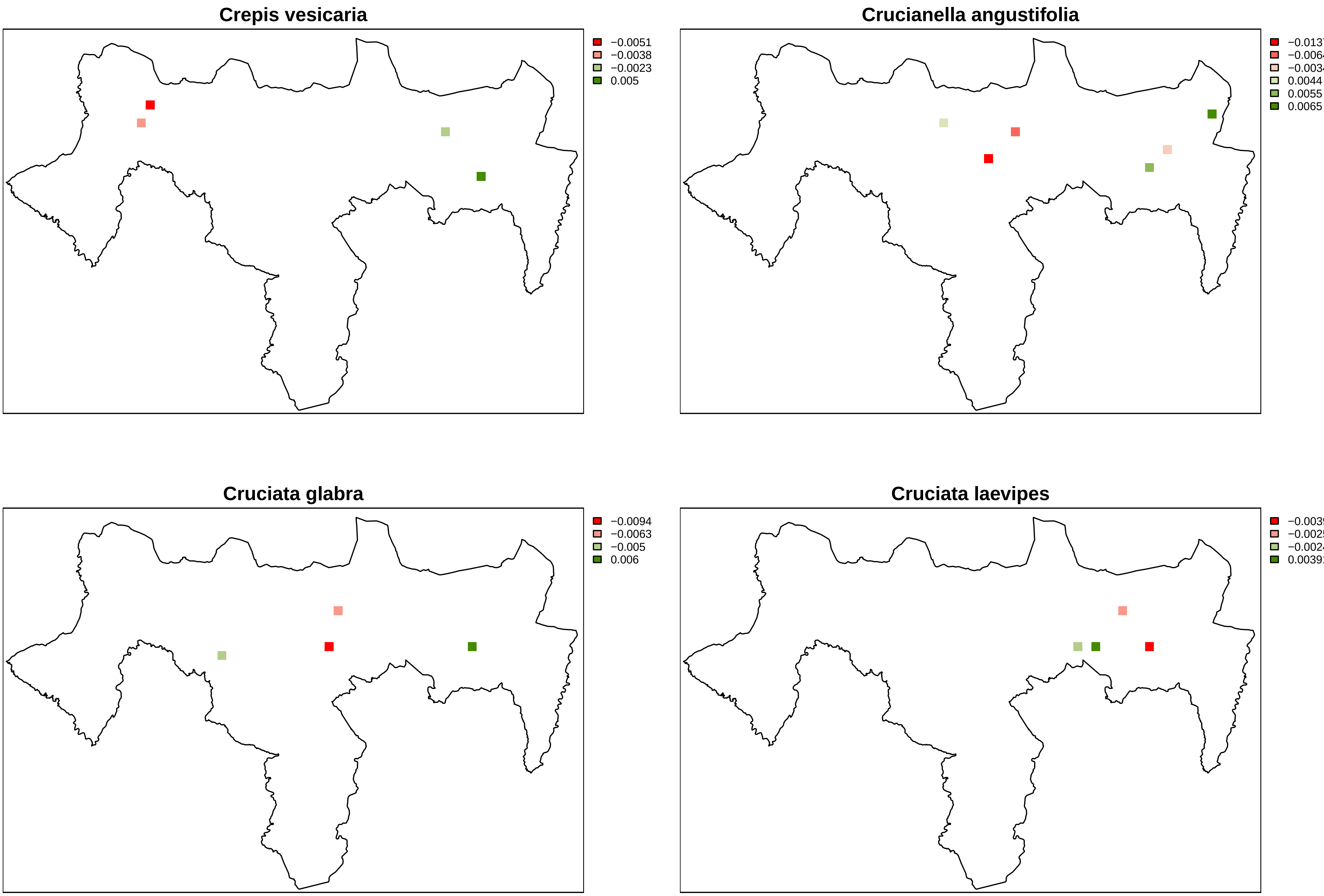
Crepis vesicaria
−0.0051
−0.0038
−0.0023
0.005
Crucianella angustifolia
0.0044
0.0055
0.0065
Cruciata glabra
−0.0094
−0.0063
−0.005
0.006
Cruciata laevipes

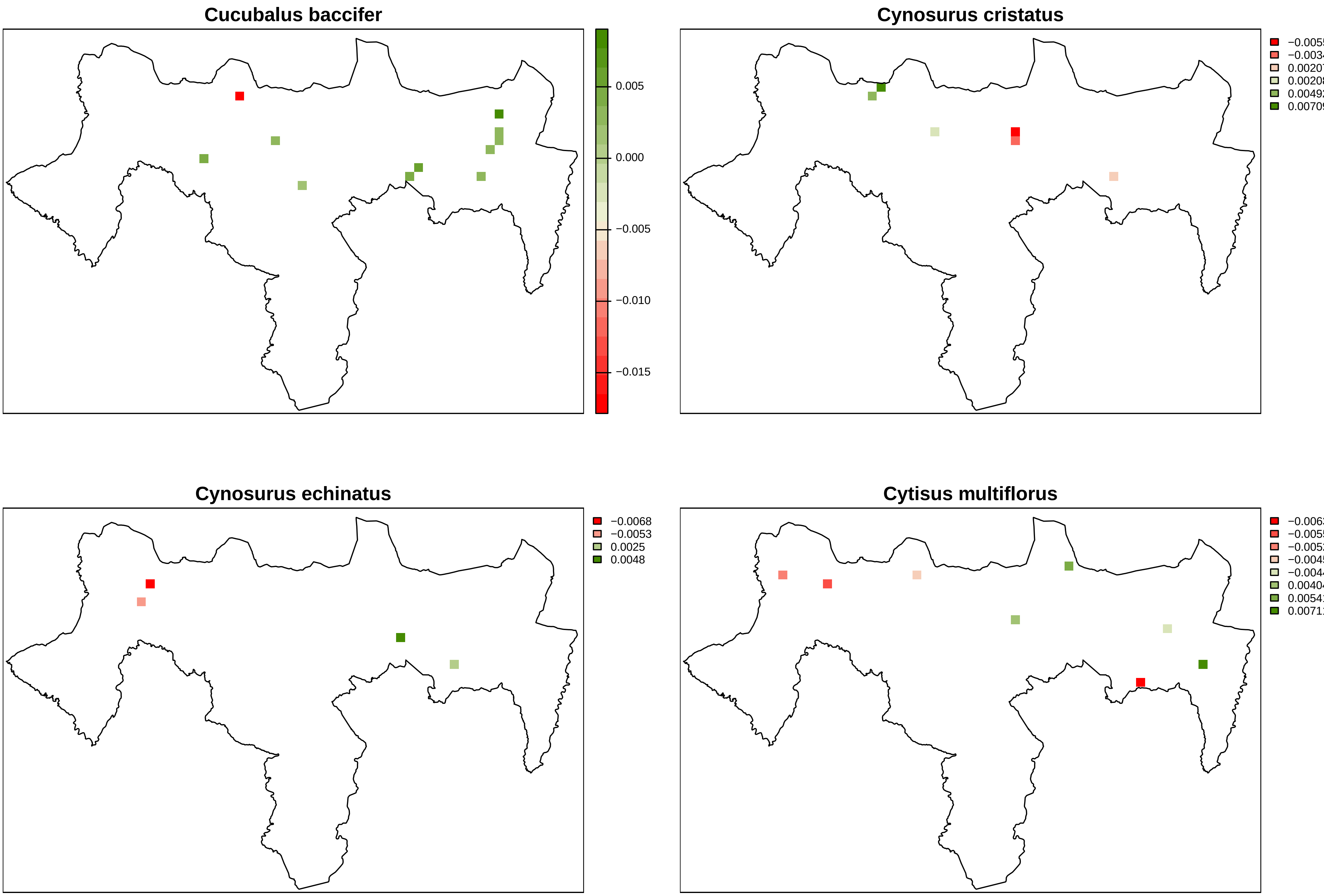

Cucubalus baccifer
0.005
0.000
−0.005
−0.010
−0.015
Cynosurus cristatus
−0.0055
−0.0034
0.00207
0.00208
0.00492
0.00709
Cynosurus echinatus
−0.0068
−0.0053
0.0025
0.0048
Cytisus multiflorus
−0.0063
−0.0055
−0.0052
−0.0045
−0.0044
0.00404
0.00541
0.00711

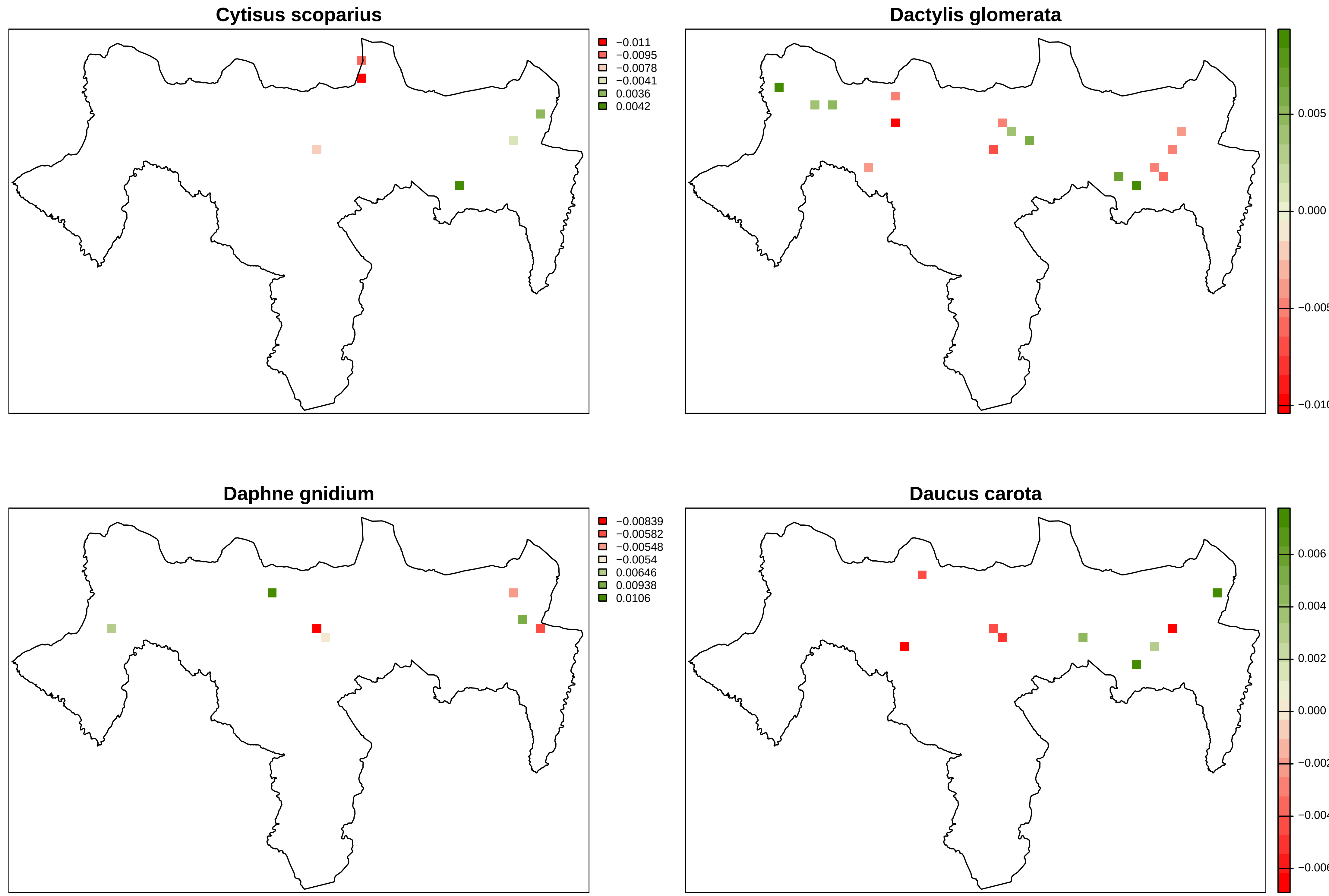
Cytisus scoparius
−0.011
−0.0095
−0.0078
−0.0041
0.0036
0.0042
Dactylis glomerata
0.005
0.000
Daphne gnidium
−0.00839
−0.00582
−0.00548
−0.0054
0.00646
0.00938
0.0106
Daucus carota
0.006
0.004
0.002
0.000

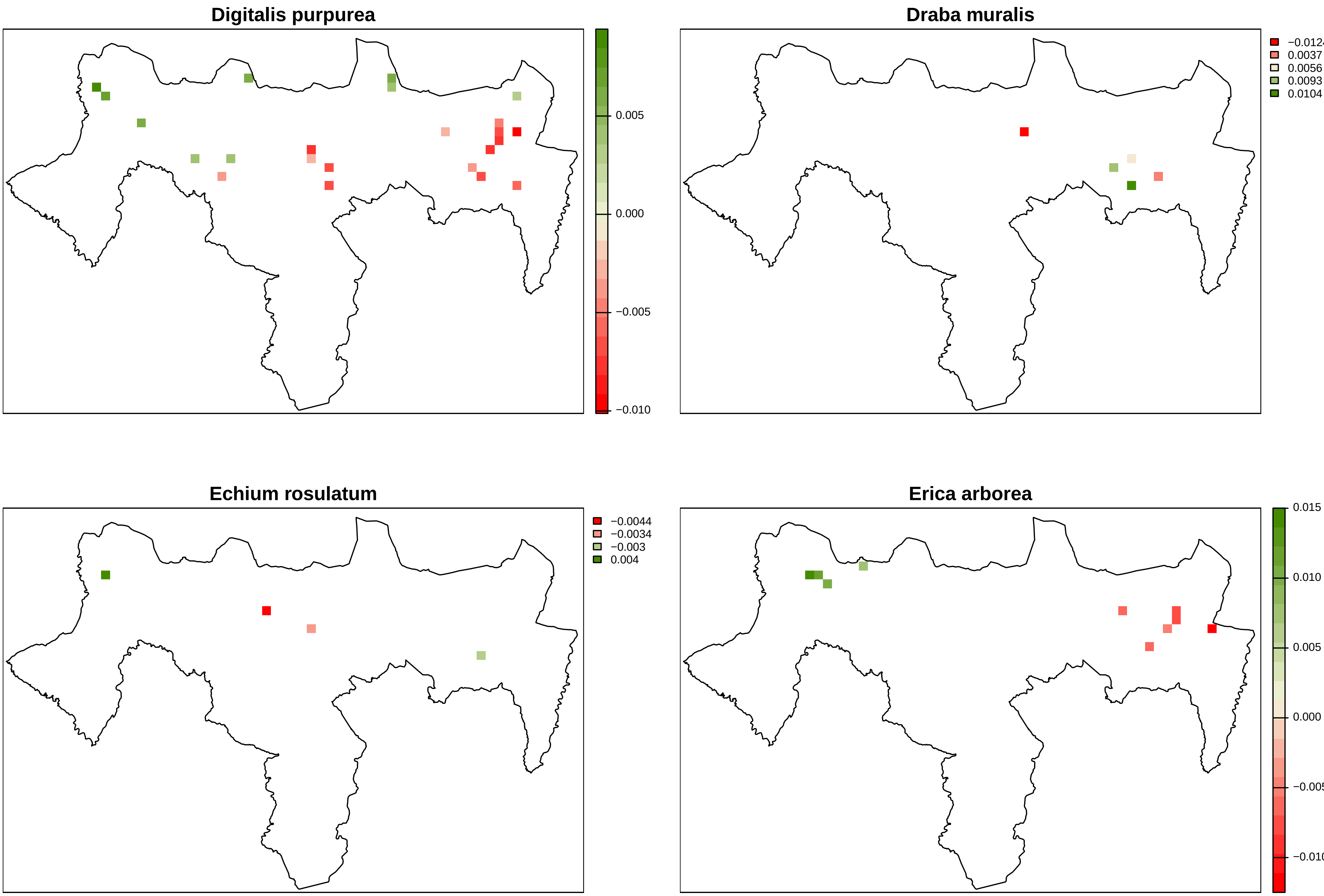

Digitalis purpurea
0.005
0.000
−0.005
−0.010
Draba muralis
−0.012
0.0037
0.0056
0.0093
0.0104
Echium rosulatum
−0.0044
−0.0034
−0.003
0.004
Erica arborea
0.015
0.010
0.005
0.000
−0.00
−0.01

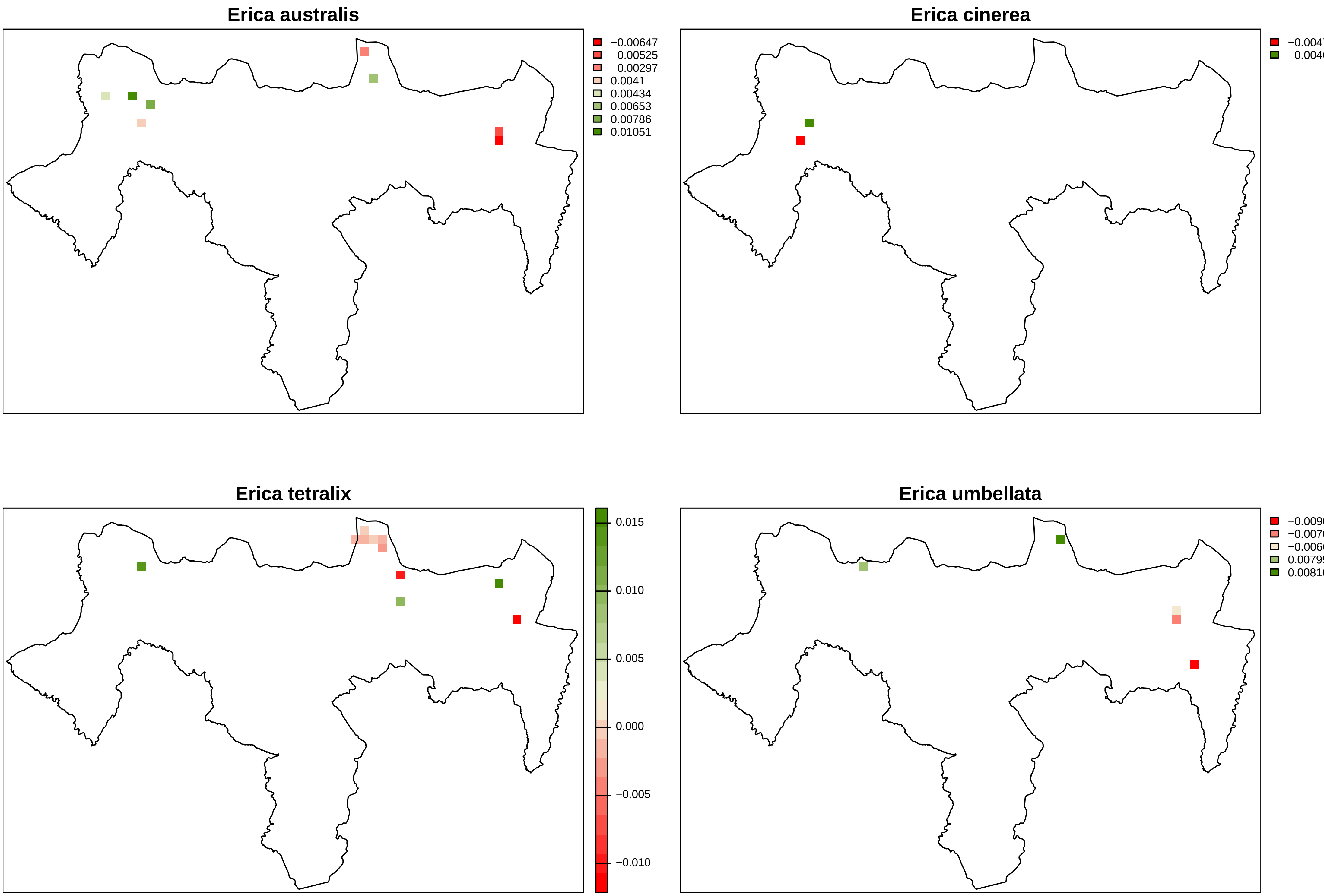
Erica australis
−0.00647
−0.00525
−0.00297
0.0041
0.00434
0.00653
0.00786
0.01051
Erica cinerea
Erica tetralix
0.015
0.010
0.005
0.000
−0.005
−0.010
Erica umbellata

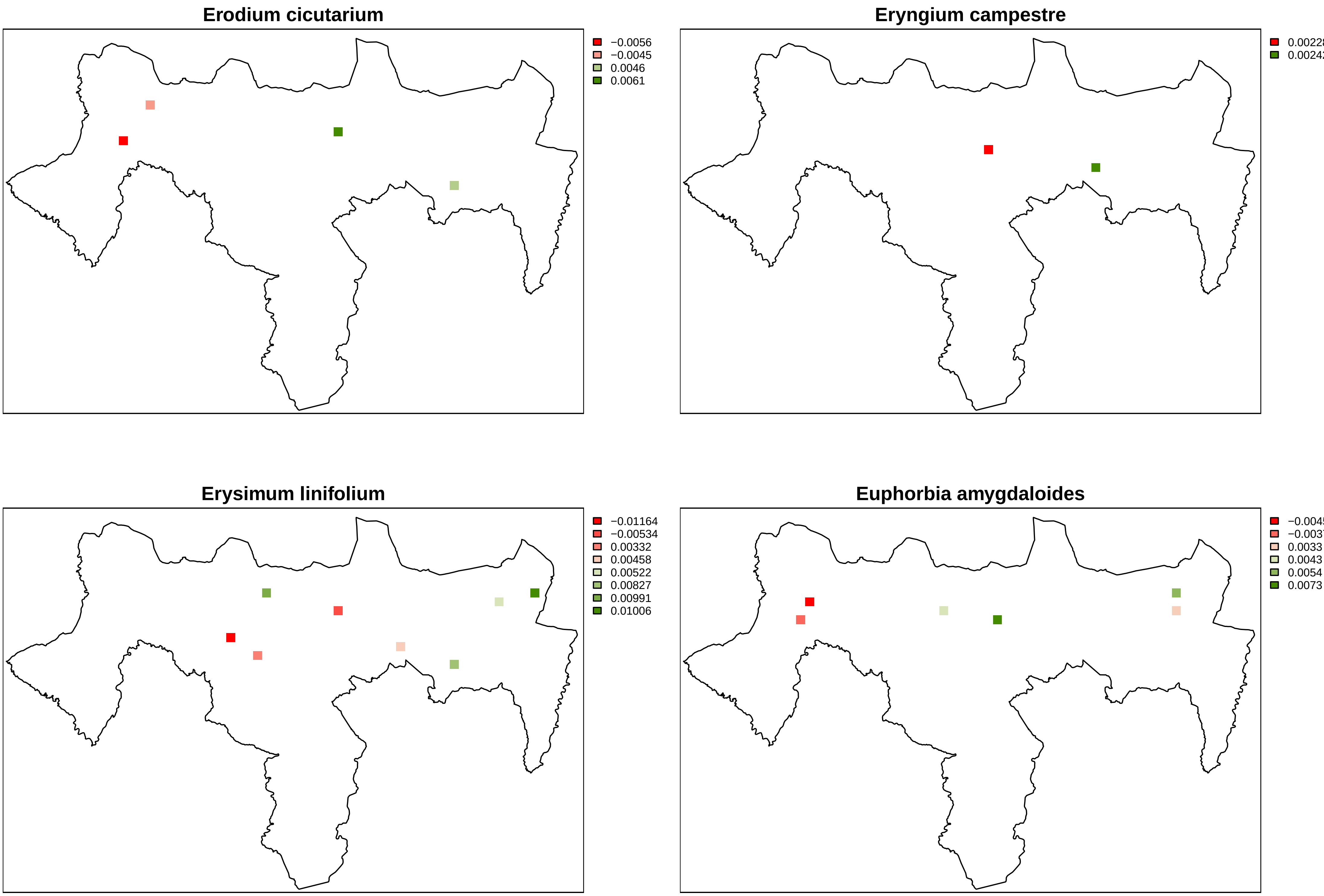
Erodium cicutarium
−0.0056
−0.0045
0.0046
0.0061
Eryngium campestre
0.0022
0.0024
Erysimum linifolium
−0.01164
−0.00534
0.00332
0.00458
0.00522
0.00827
0.00991
0.01006
Euphorbia amygdaloides
−0.004
−0.003
0.0033
0.0043
0.0054
0.0073

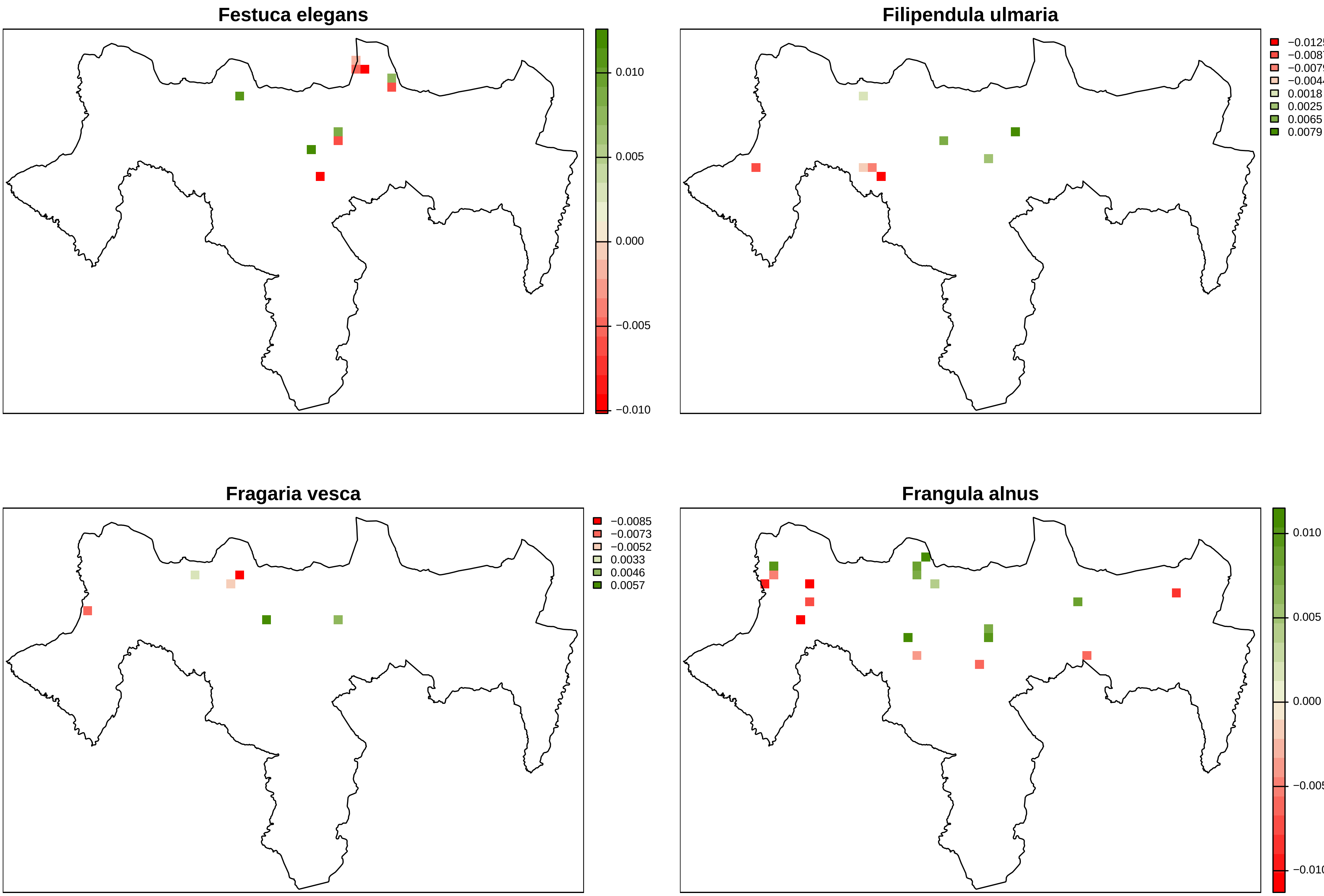

Festuca elegans
0.010
0.005
0.000
−0.005
−0.010
Filipendula ulmaria
−0.012
−0.008
−0.007
−0.004
0.0018
0.0025
0.0065
0.0079
Fragaria vesca
−0.0085
−0.0073
−0.0052
0.0033
0.0046
0.0057
Frangula alnus
0.010
0.005
0.000
−0.00
−0.01

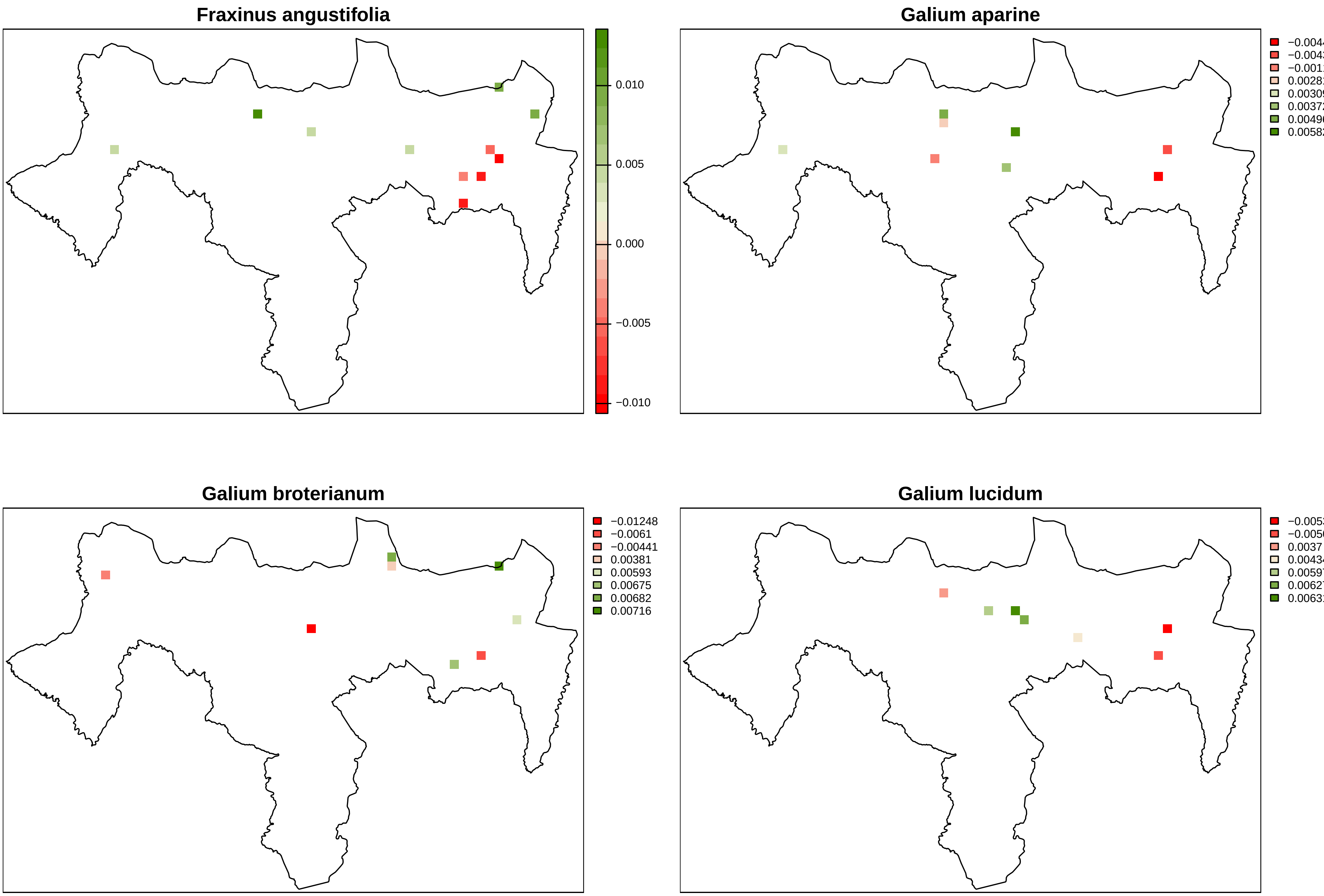
Fraxinus angustifolia
0.010
0.005
0.000
−0.005
−0.010
Galium aparine
Galium broterianum
−0.01248
−0.0061
−0.00441
0.00381
0.00593
0.00675
0.00682
0.00716
Galium lucidum

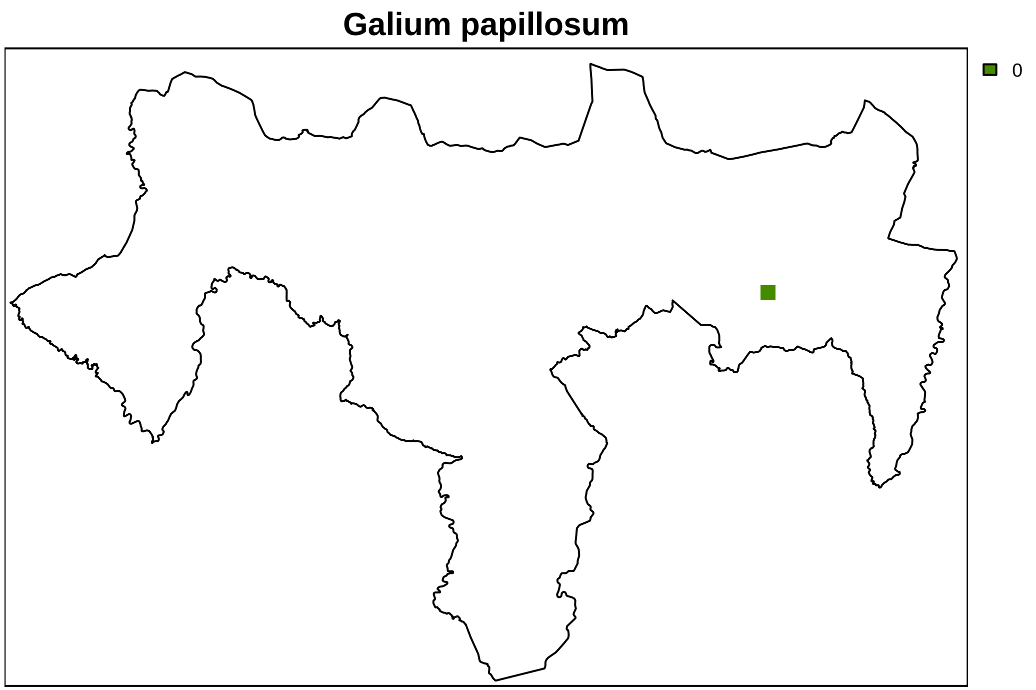
Galium papillosum
0

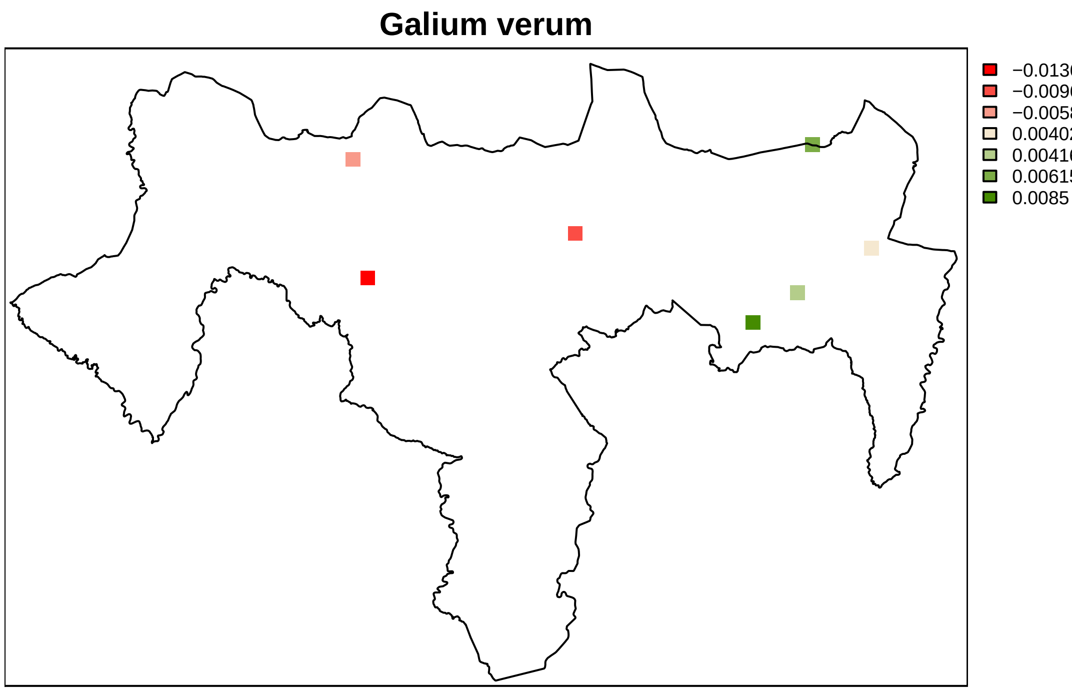
Galium verum
−0.013
−0.009
−0.005
0.0040
0.0041
0.0061
0.0085

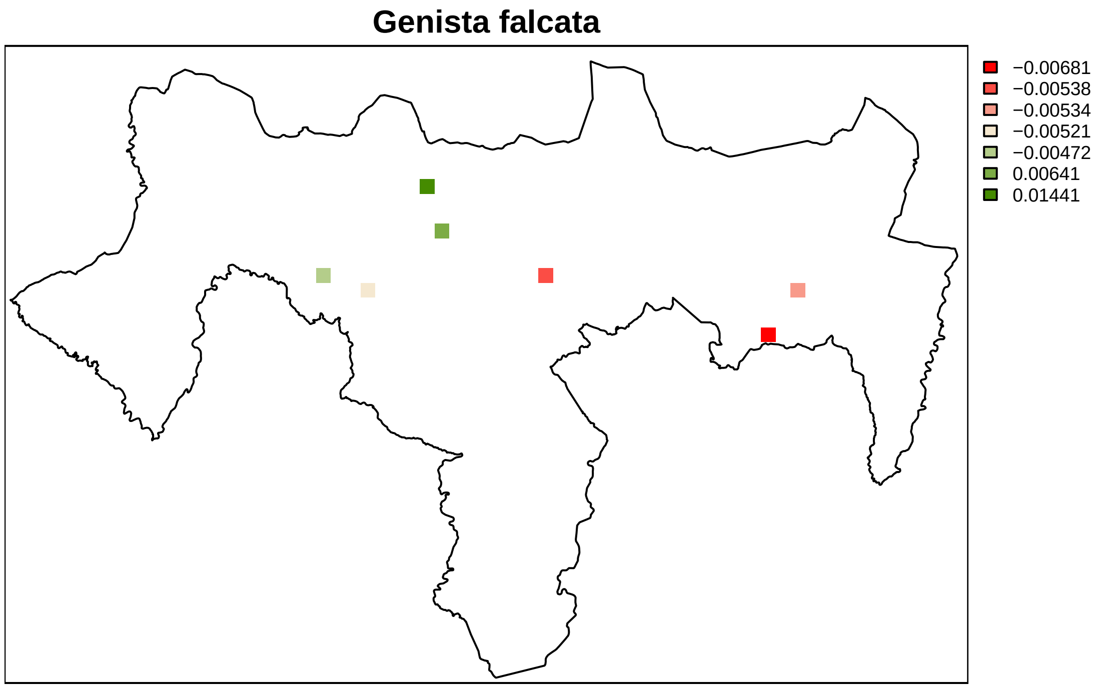
Genista falcata
−0.00681
−0.00538
−0.00534
−0.00521
−0.00472
0.00641
0.01441

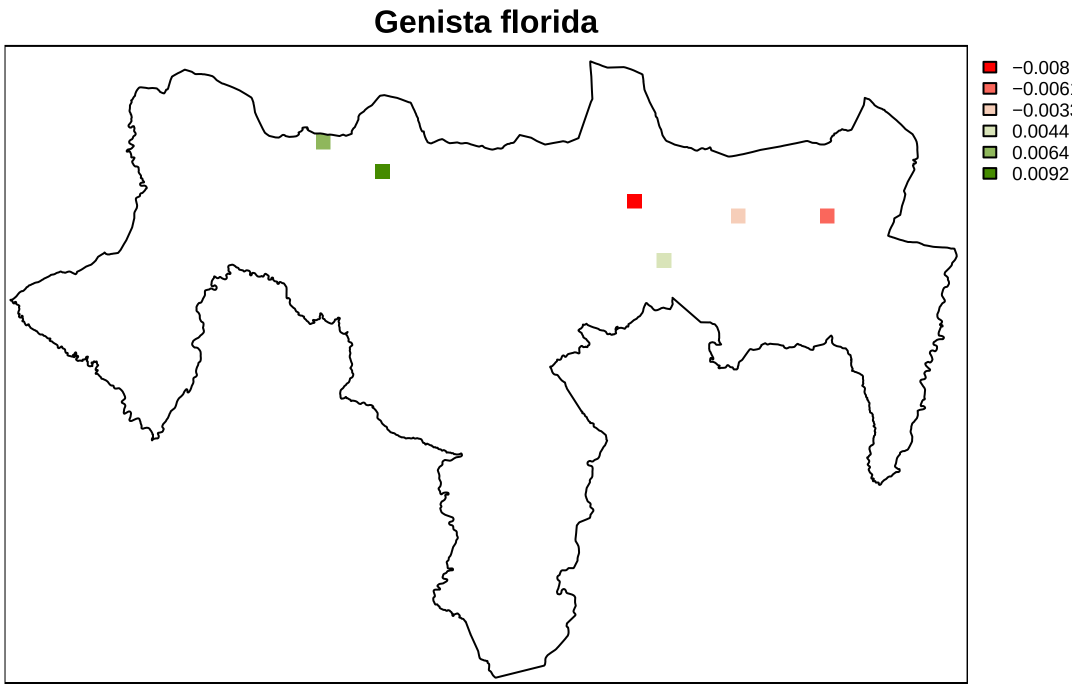
Genista florida
−0.008
−0.006
−0.003
0.0044
0.0064
0.0092

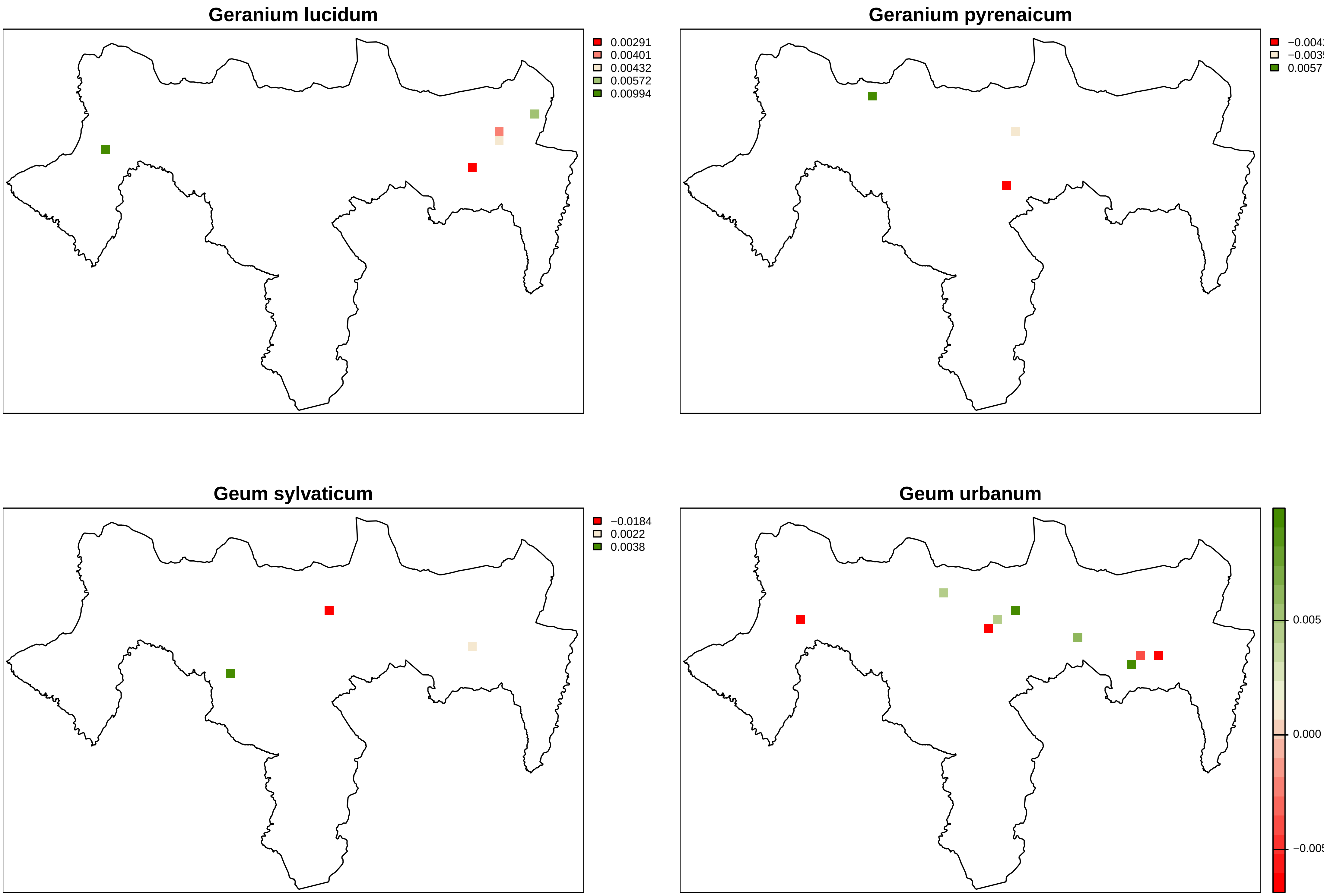
Geranium lucidum
0.00291
0.00401
0.00432
0.00572
0.00994
Geranium pyrenaicum
−0.004
−0.003
0.0057
Geum sylvaticum
−0.0184
0.0022
0.0038
Geum urbanum
0.005
0.000
−0.00

Halimium lasianthum

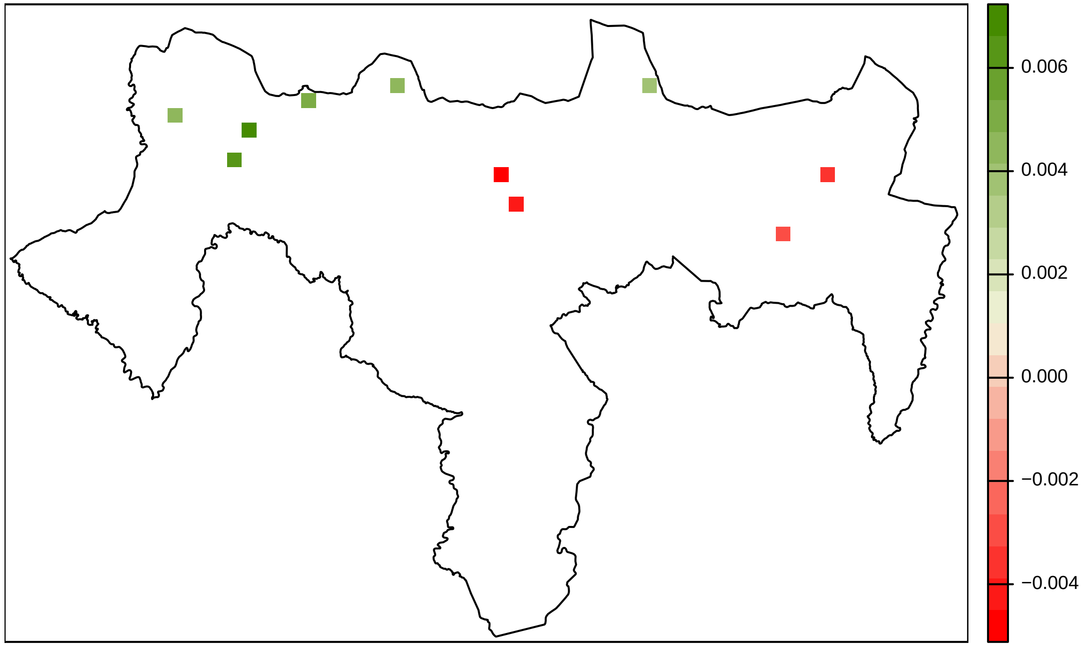

Halimium umbellatum

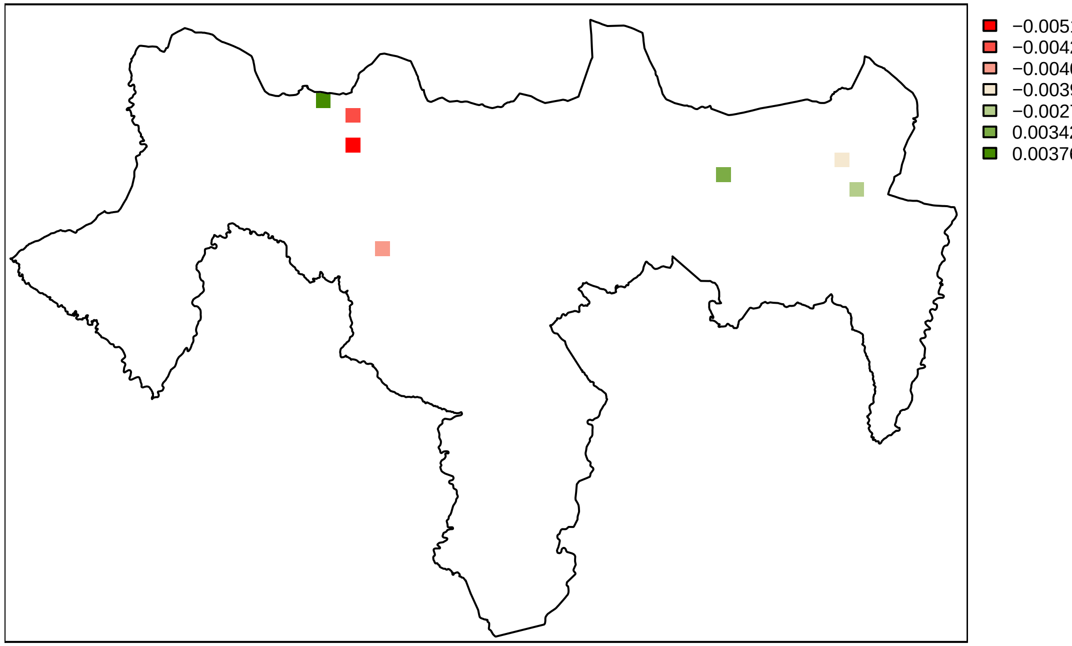

Helichrysum stoechas

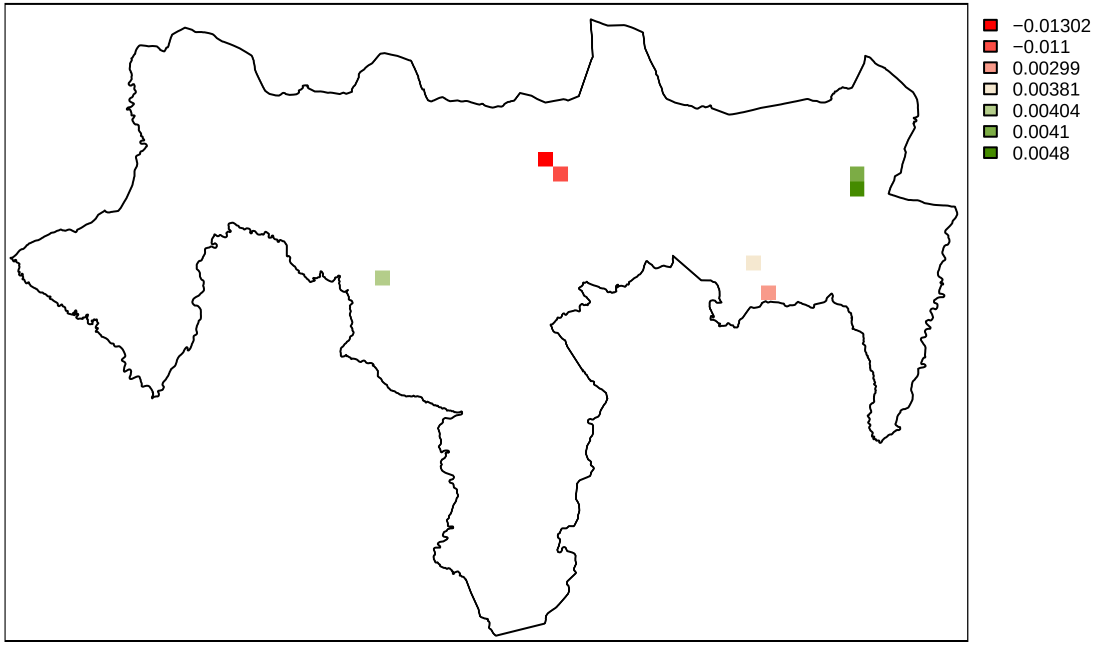

Helleborus foetidus

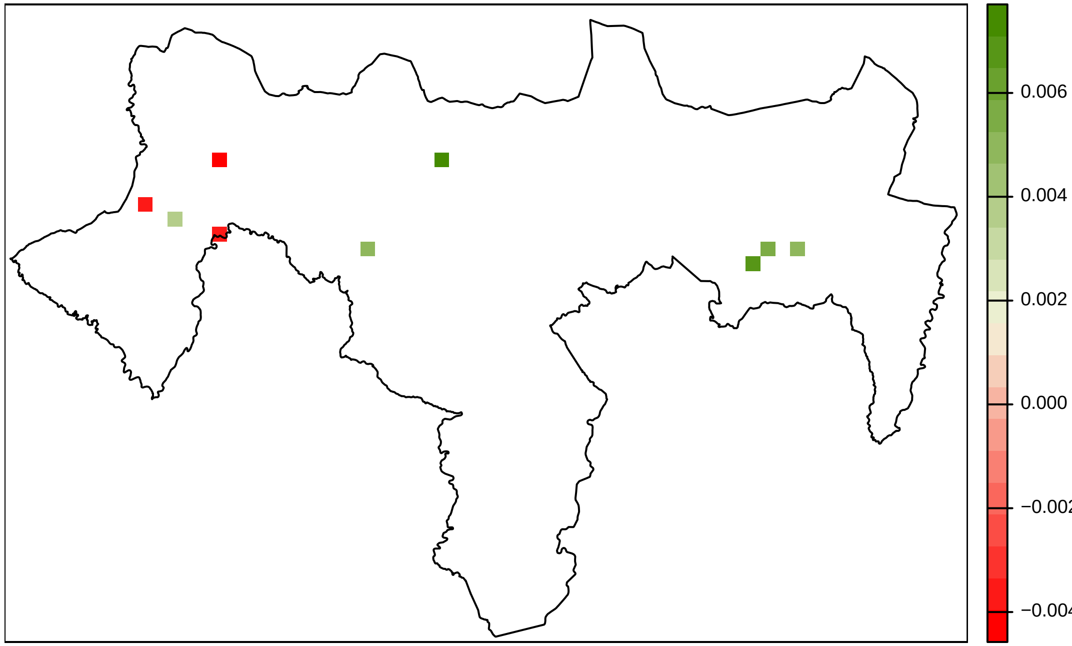

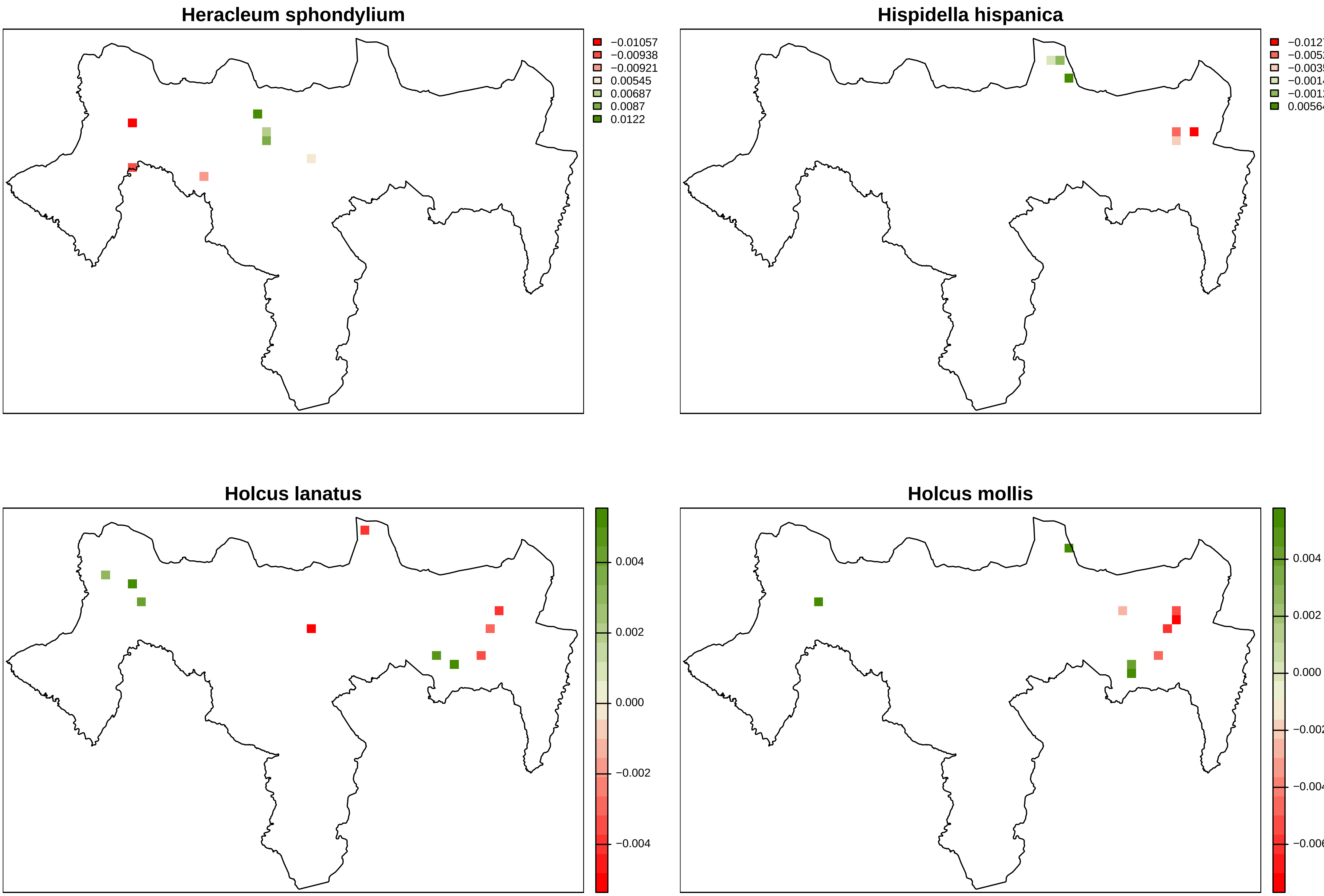

Heracleum sphondylium
−0.01057
−0.00938
−0.00921
0.00545
0.00687
0.0087
0.0122
Hispidella hispanica
Holcus lanatus
0.004
0.002
0.000
−0.002
−0.004
Holcus mollis
0.004
0.002
0.000

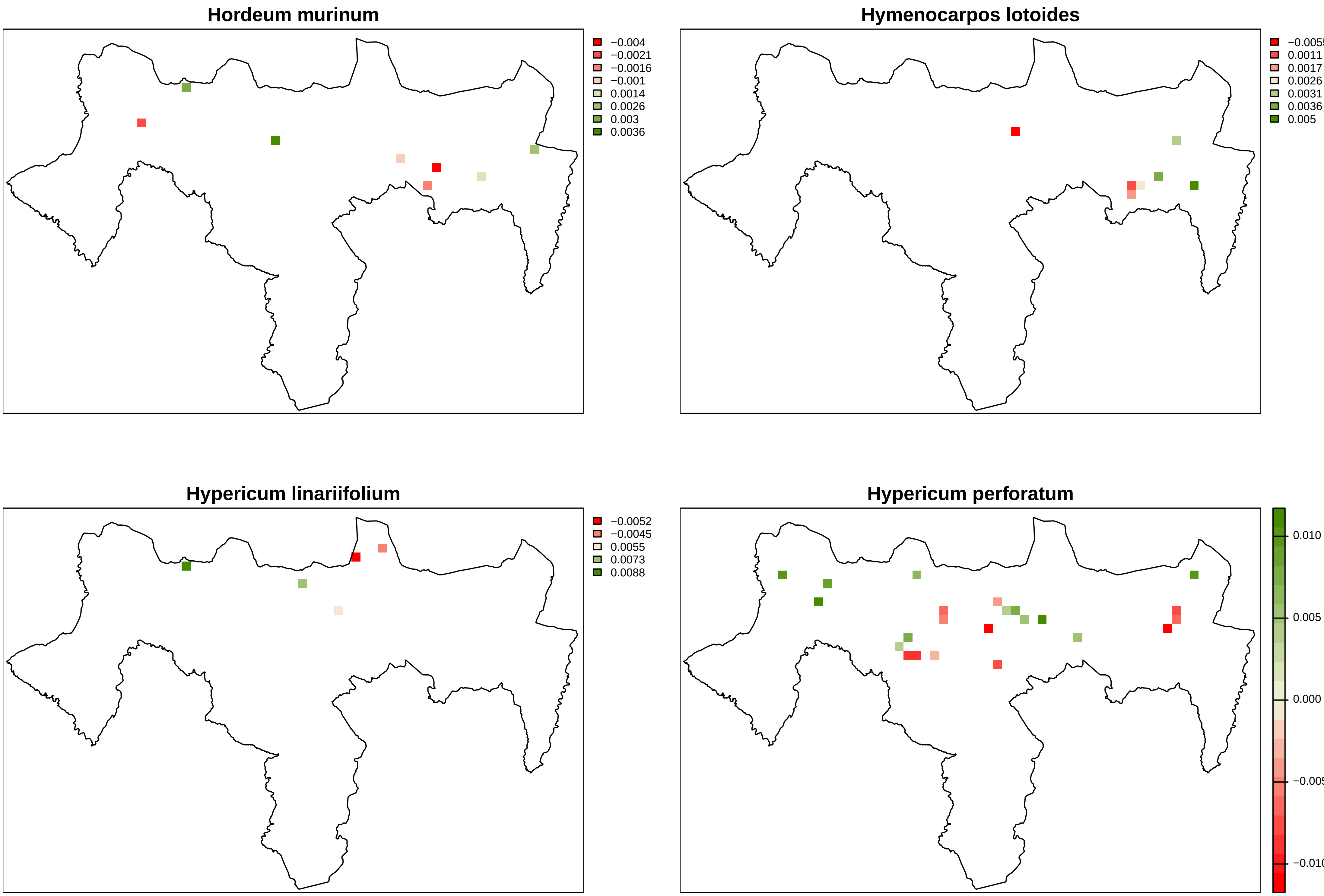
Hordeum murinum
−0.004
−0.0021
−0.0016
−0.001
0.0014
0.0026
0.003
0.0036
Hymenocarpos lotoides
−0.005
0.0011
0.0017
0.0026
0.0031
0.0036
0.005
Hypericum linariifolium
−0.0052
−0.0045
0.0055
0.0073
0.0088
Hypericum perforatum
0.010
0.005
0.000
−0.005
−0.01

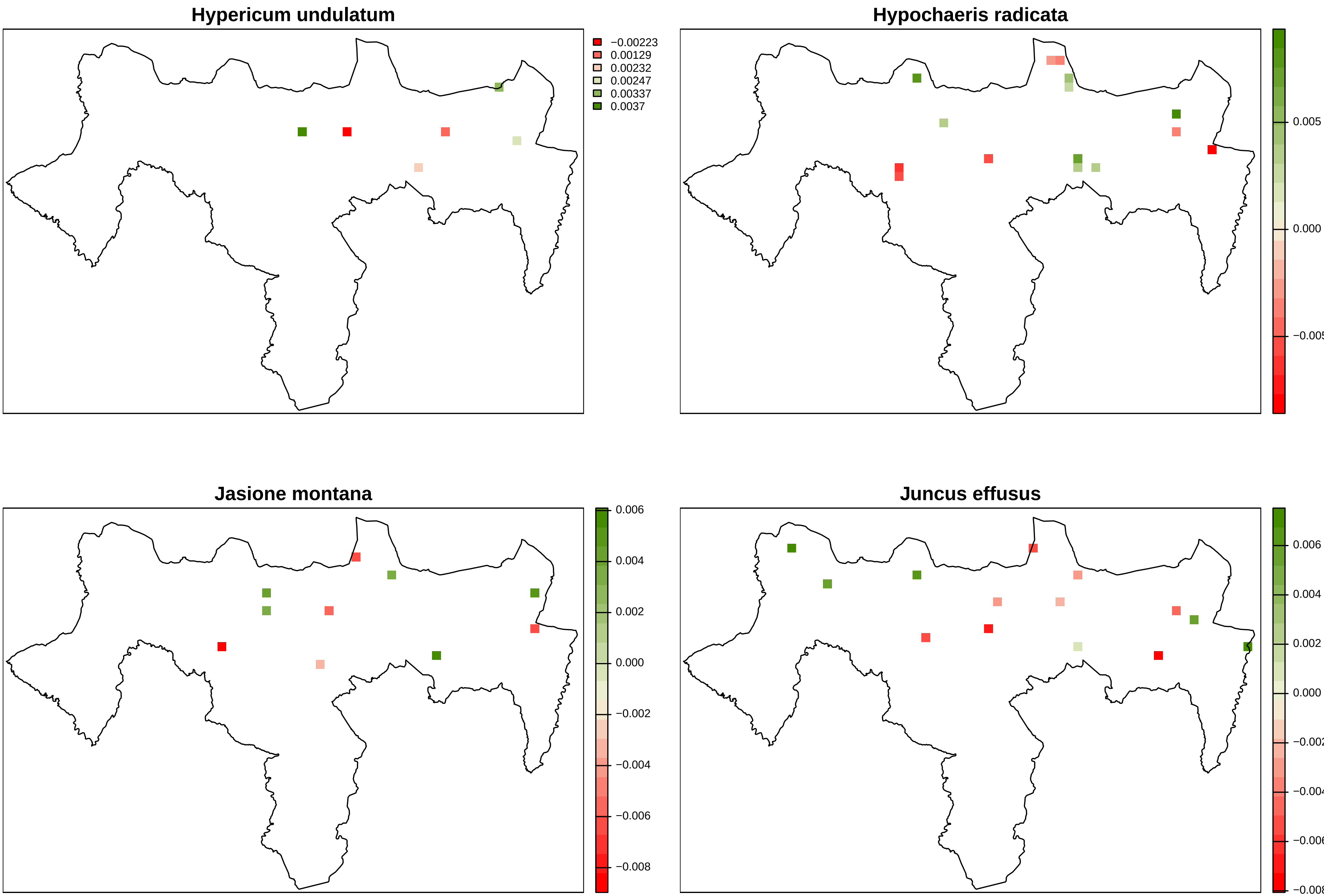
Hypericum undulatum
−0.00223
0.00129
0.00232
0.00247
0.00337
0.0037
Hypochaeris radicata
0.005
0.000
Jasione montana
0.006
0.004
0.002
0.000
−0.002
−0.004
−0.006
−0.008
Juncus effusus
0.006
0.004
0.002
0.000

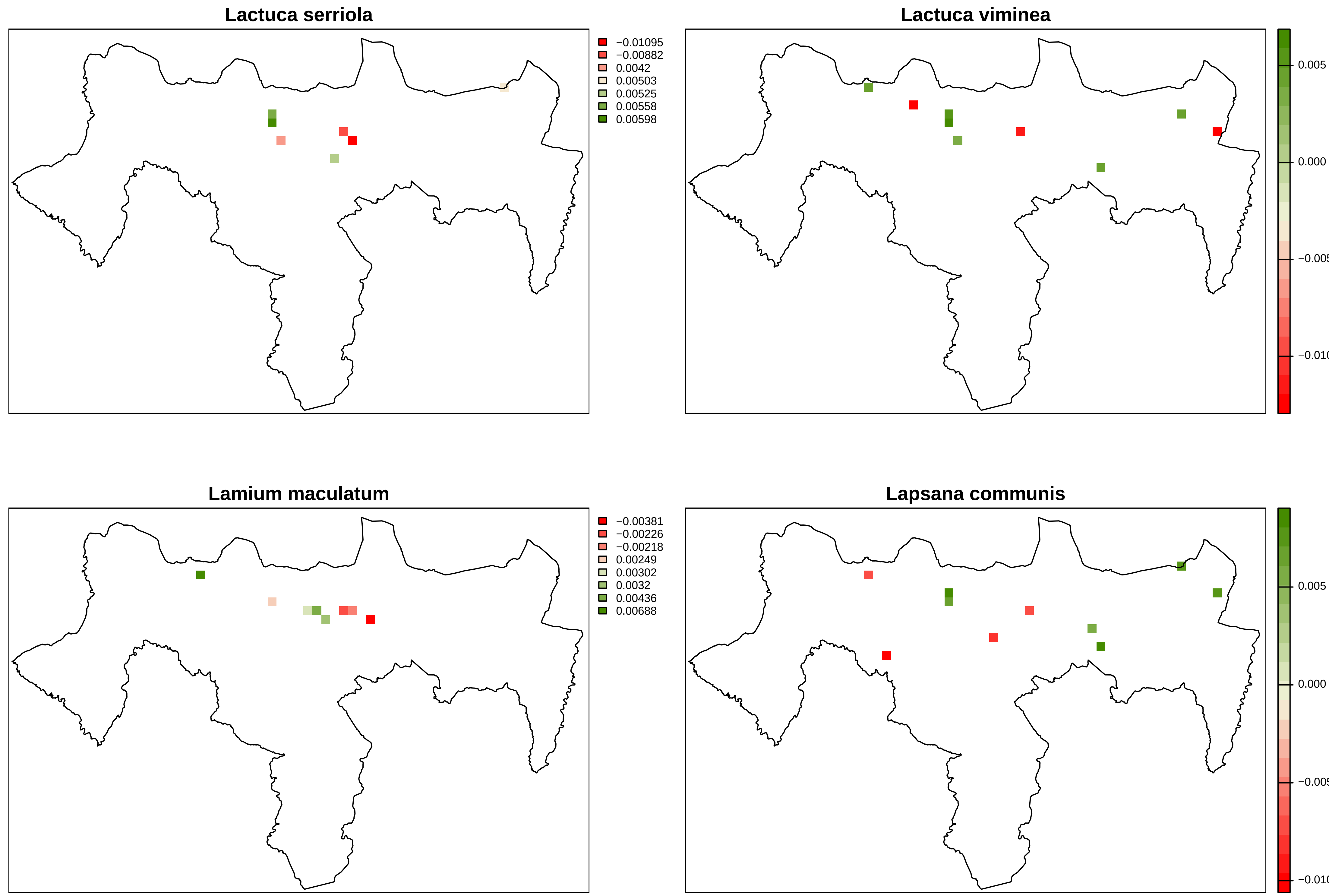
Lactuca serriola
−0.01095
−0.00882
0.0042
0.00503
0.00525
0.00558
0.00598
Lactuca viminea
0.005
0.000
Lamium maculatum
−0.00381
−0.00226
−0.00218
0.00249
0.00302
0.0032
0.00436
0.00688
Lapsana communis
0.005
0.000

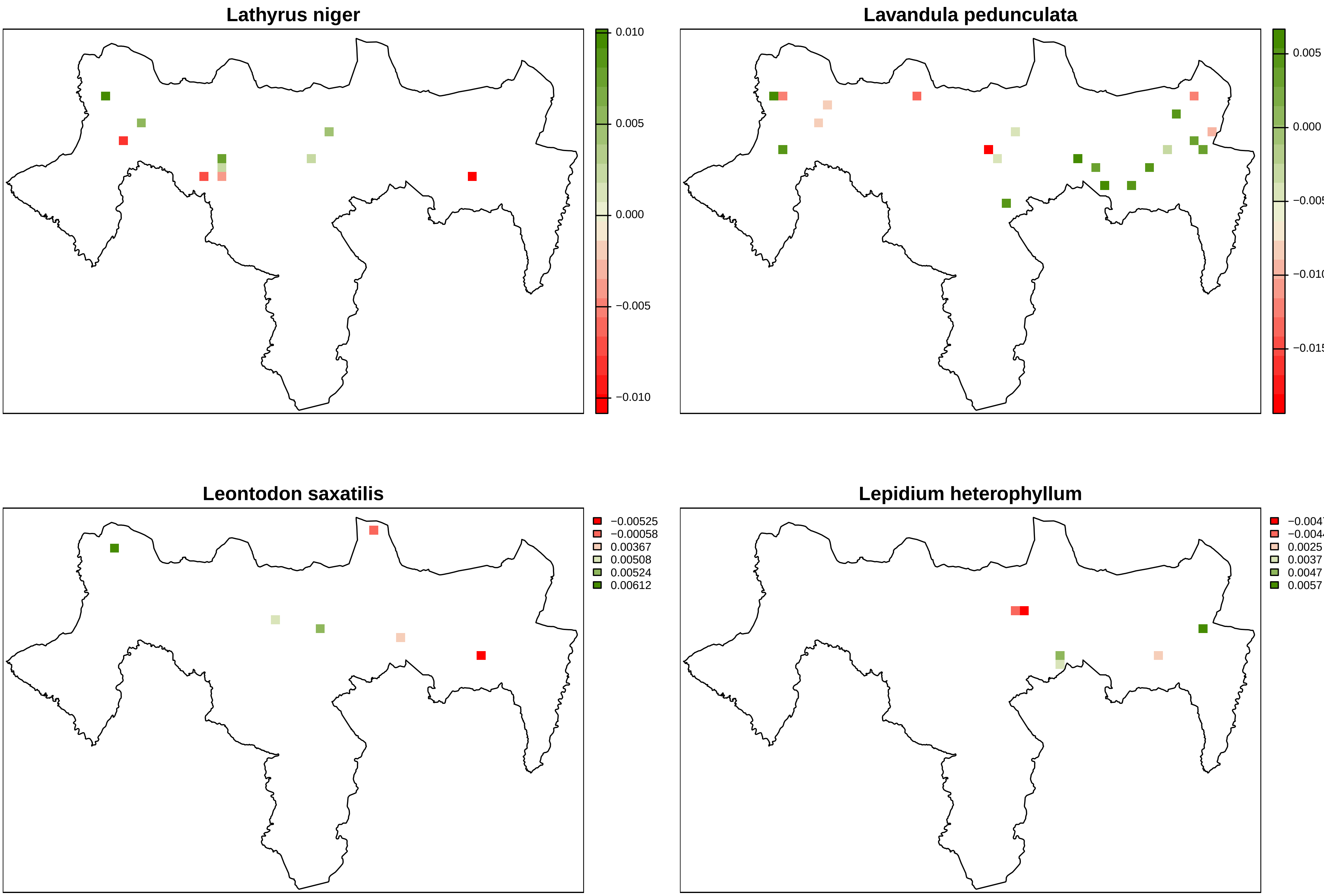

Lathyrus niger
0.010
0.005
0.000
−0.005
−0.010
Lavandula pedunculata
0.005
0.000
Leontodon saxatilis
−0.00525
−0.00058
0.00367
0.00508
0.00524
0.00612
Lepidium heterophyllum

Linaria intricata

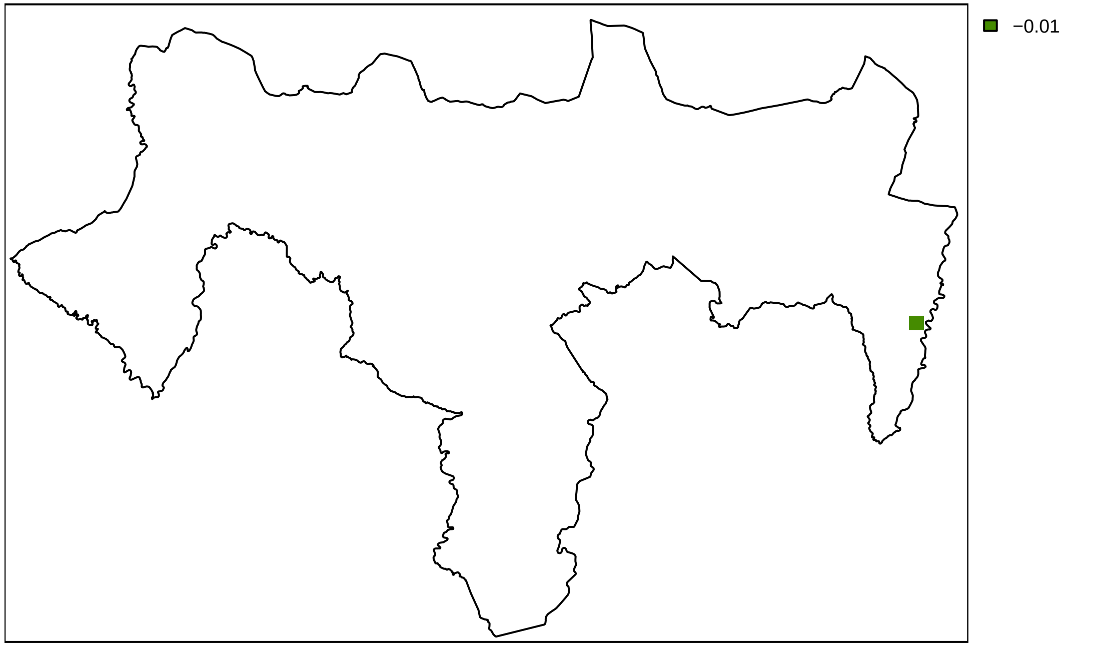

Linum bienne

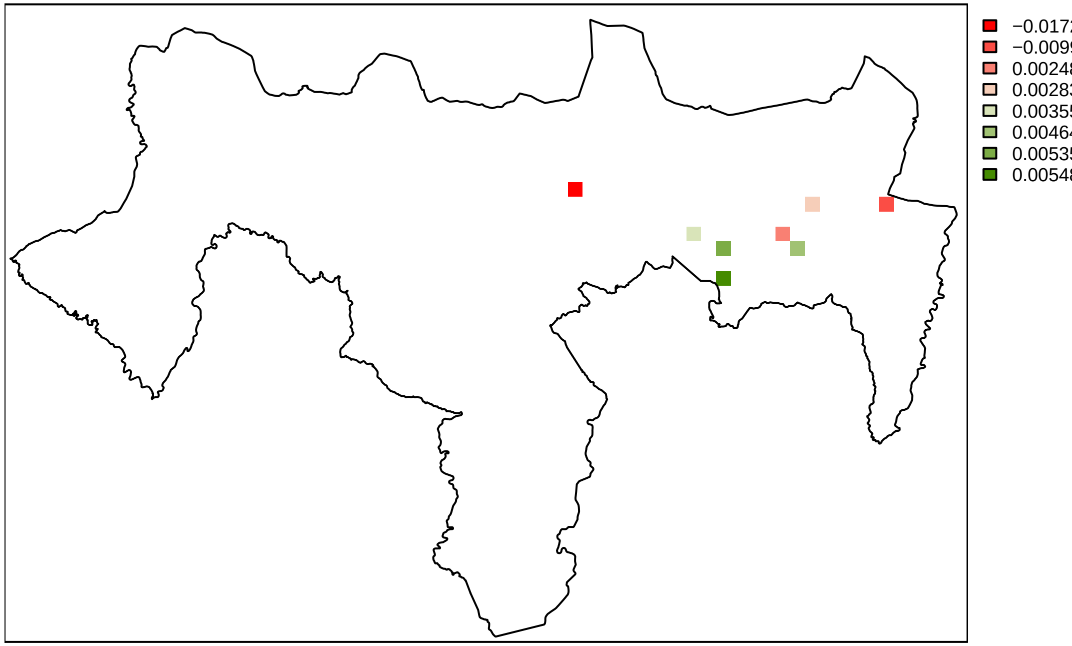

Logfia minima

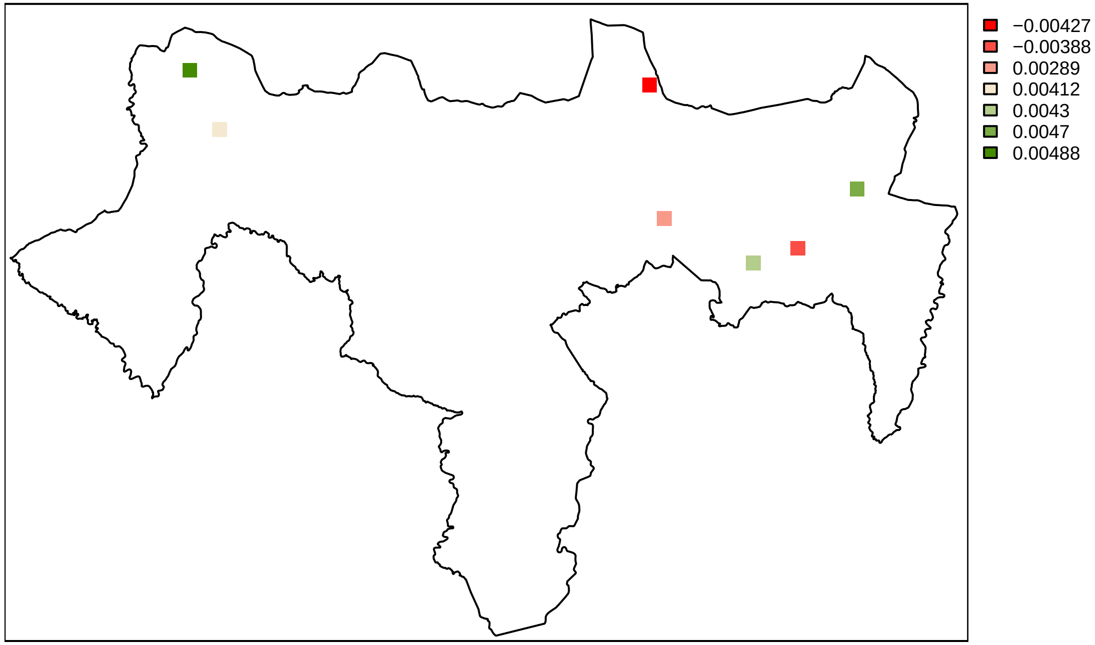

Lolium perenne

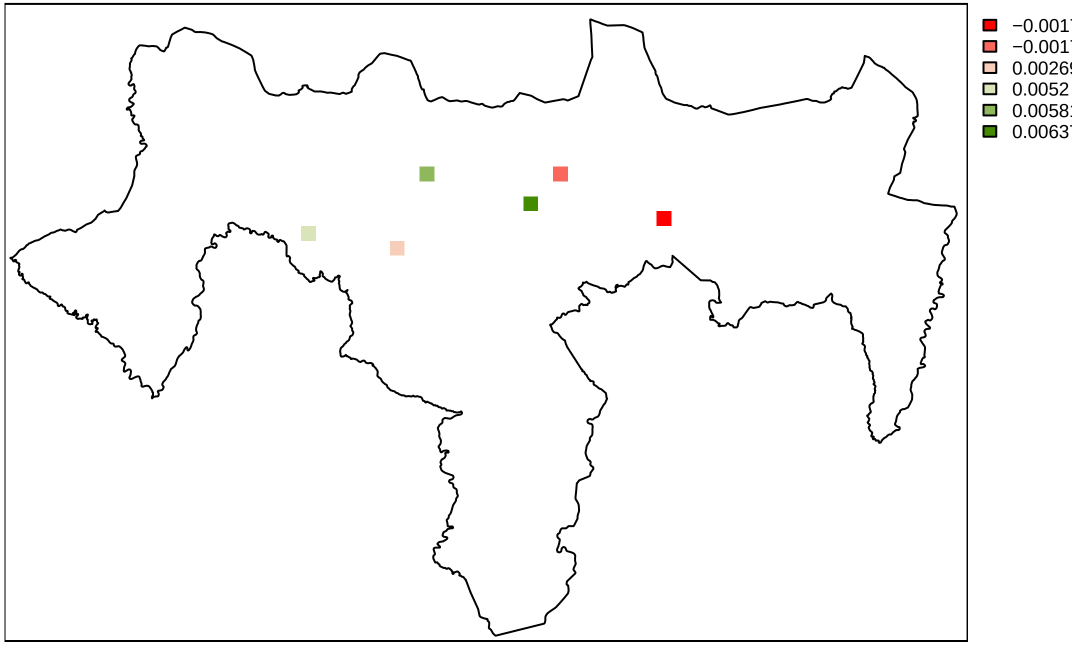

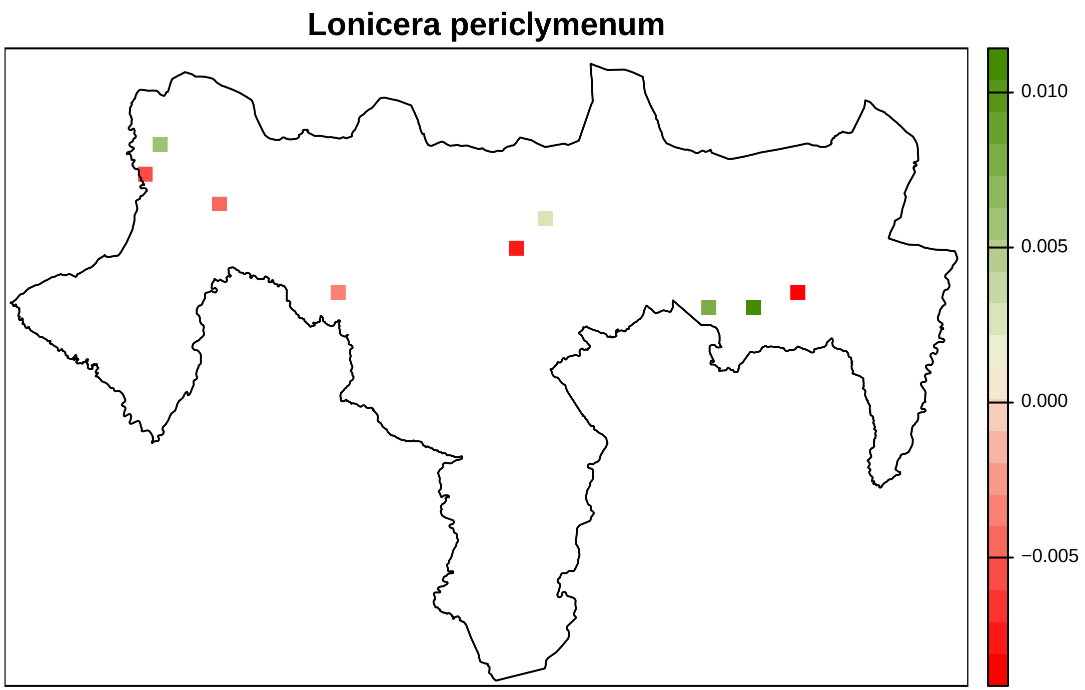
Lonicera periclymenum
0.010
0.005
0.000
−0.005

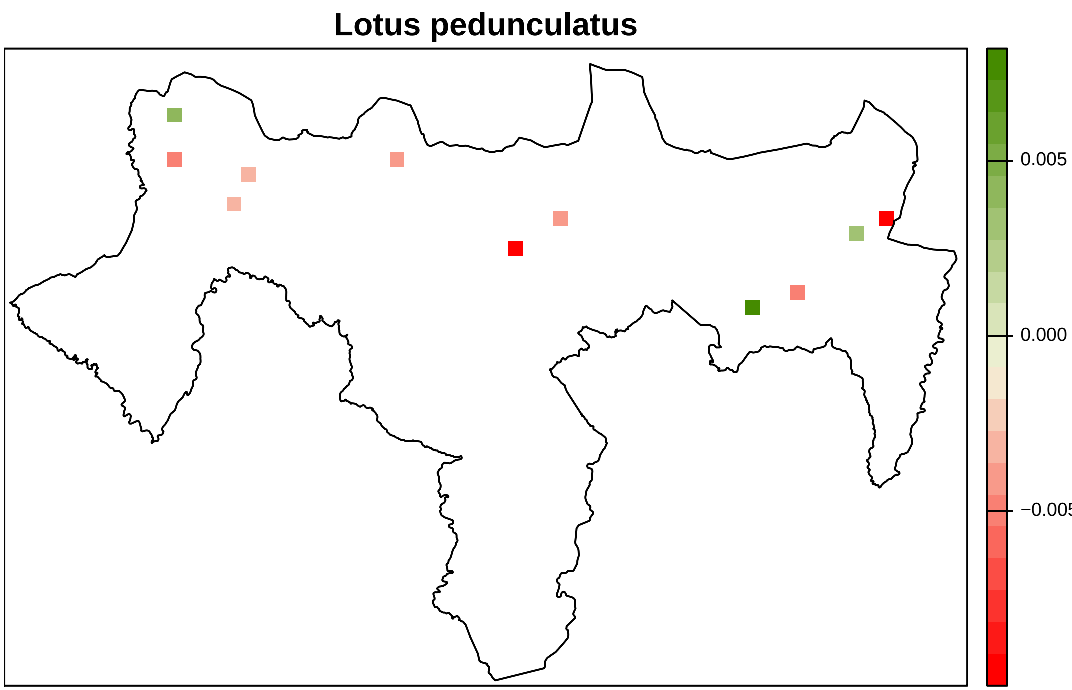
Lotus pedunculatus
0.005
0.000
−0.005

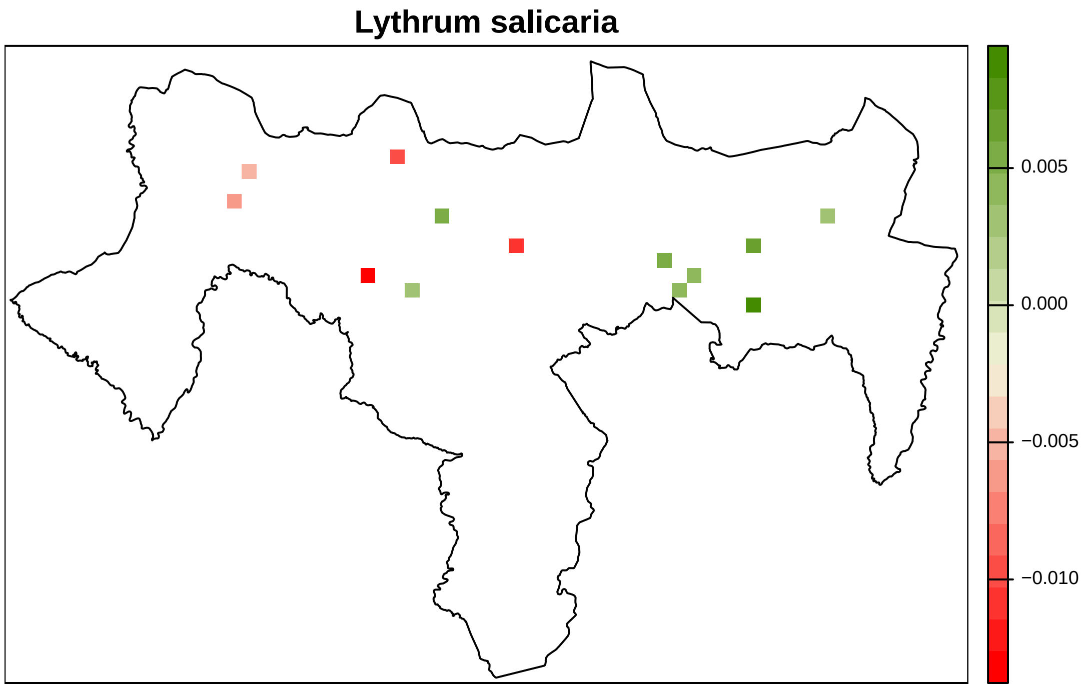
Lythrum salicaria
0.005
0.000
−0.005
−0.010

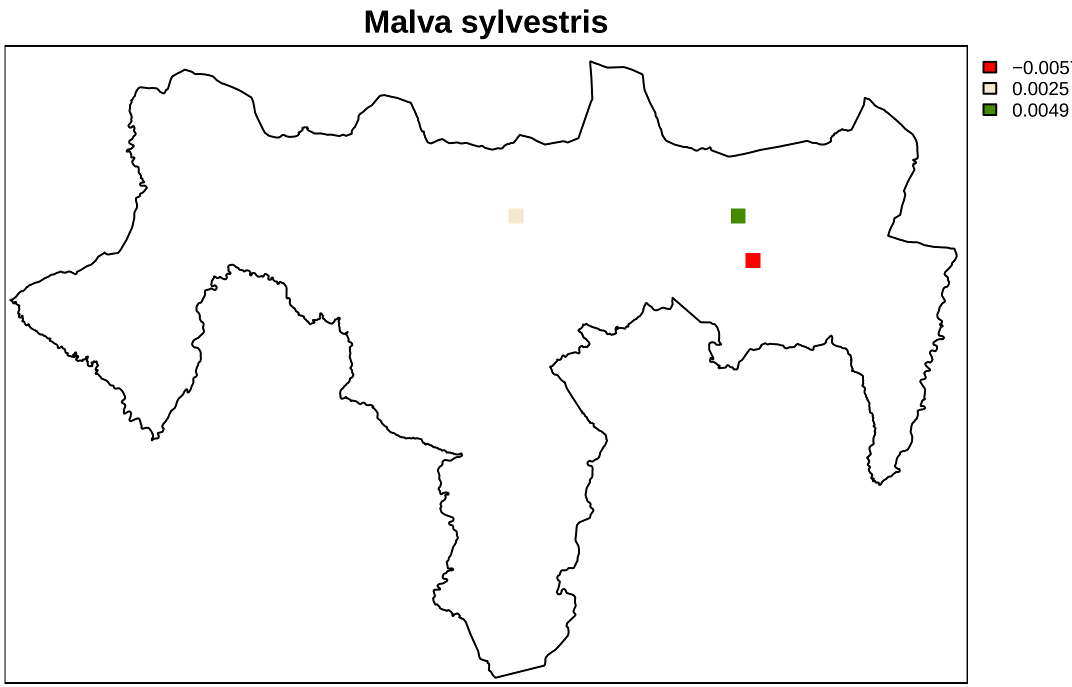
Malva sylvestris
−0.005
0.0025
0.0049

Malva tournefortiana

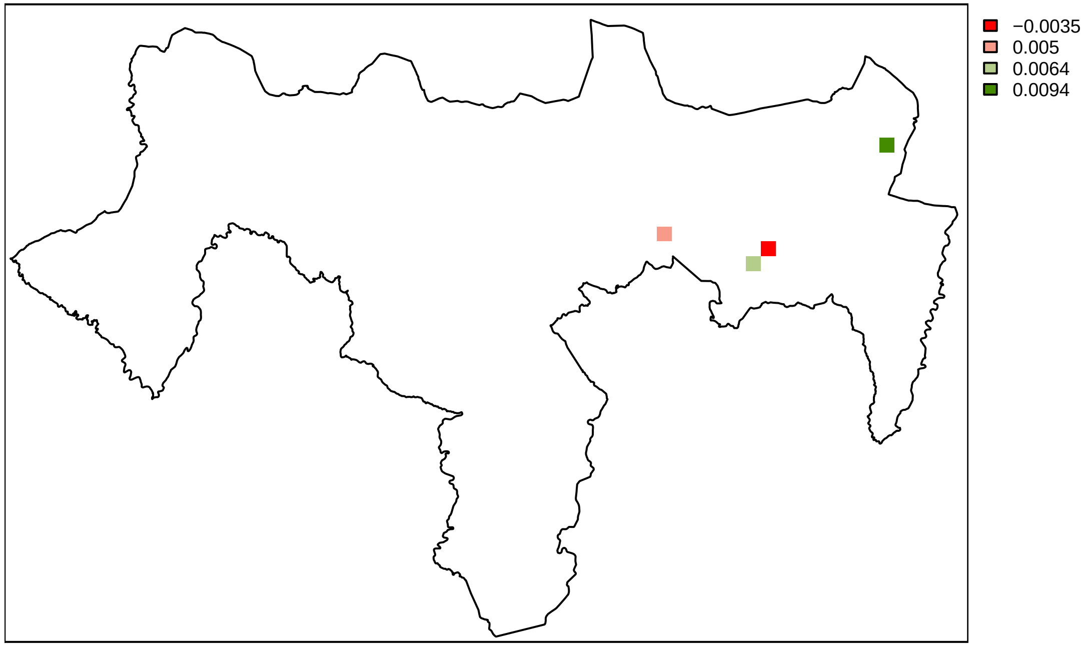

Mentha suaveolens

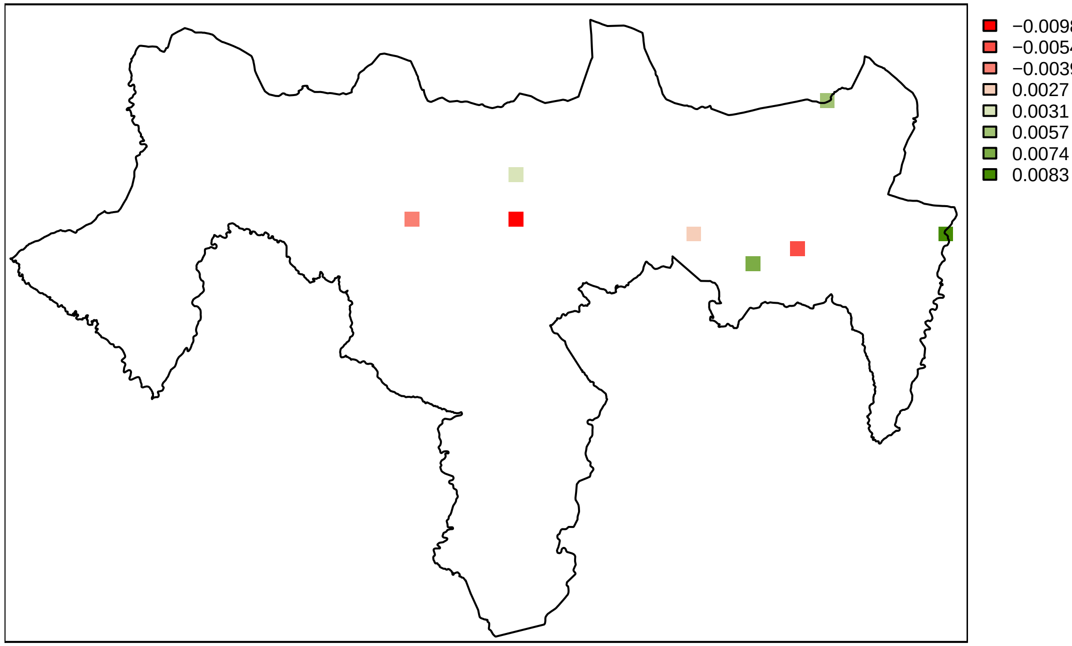

Micropyrum tenellum

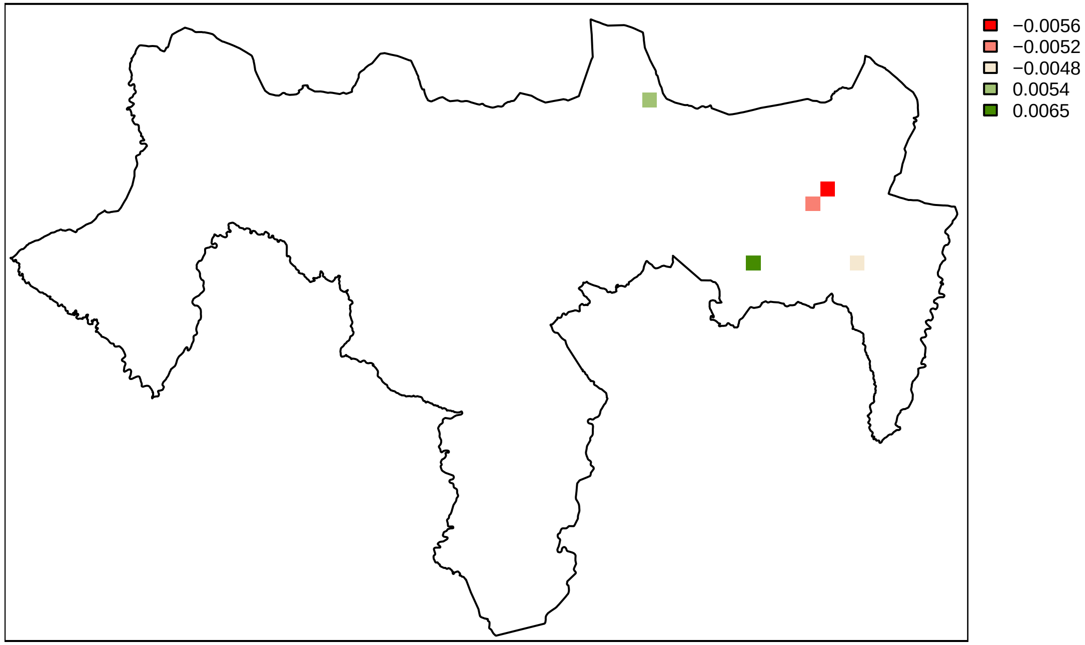

Myosotis discolor

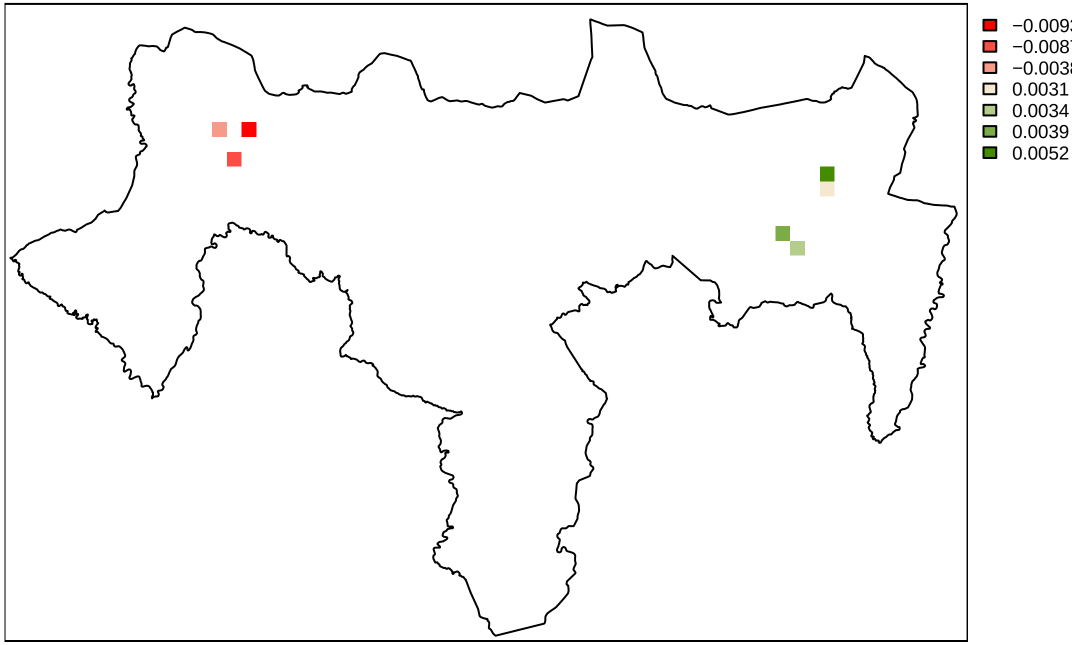

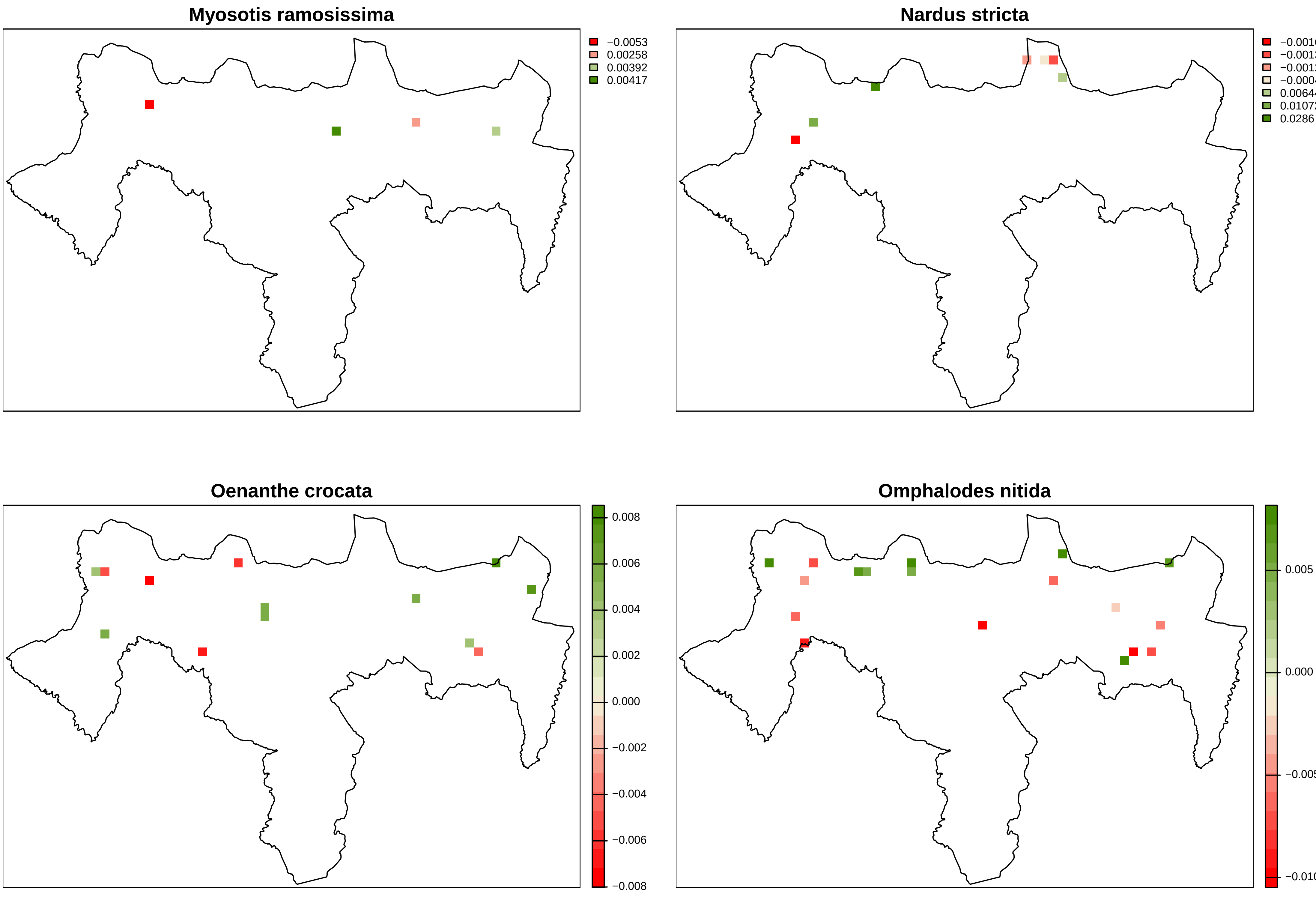

Myosotis ramosissima
−0.0053
0.00258
0.00392
0.00417
Nardus stricta
0.0286
Oenanthe crocata
0.008
0.006
0.004
0.002
0.000
−0.002
−0.004
−0.006
−0.008
Omphalodes nitida
0.005
0.000

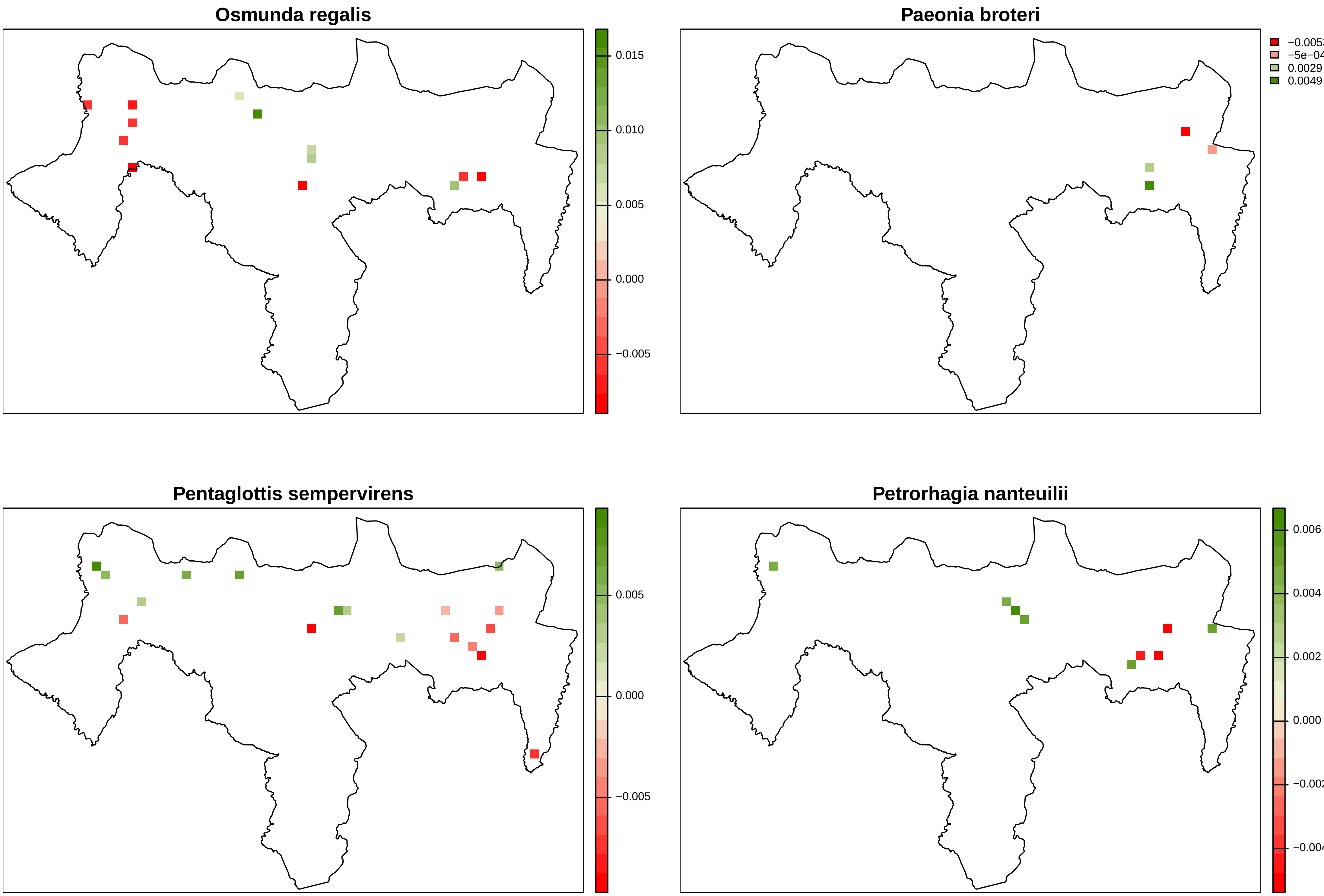
Osmunda regalis
0.015
0.010
0.005
0.000
−0.005
Paeonia broteri
0.0029
0.0049
Pentaglottis sempervirens
0.005
0.000
−0.005
Petrorhagia nanteuilii
0.006
0.004
0.002
0.000

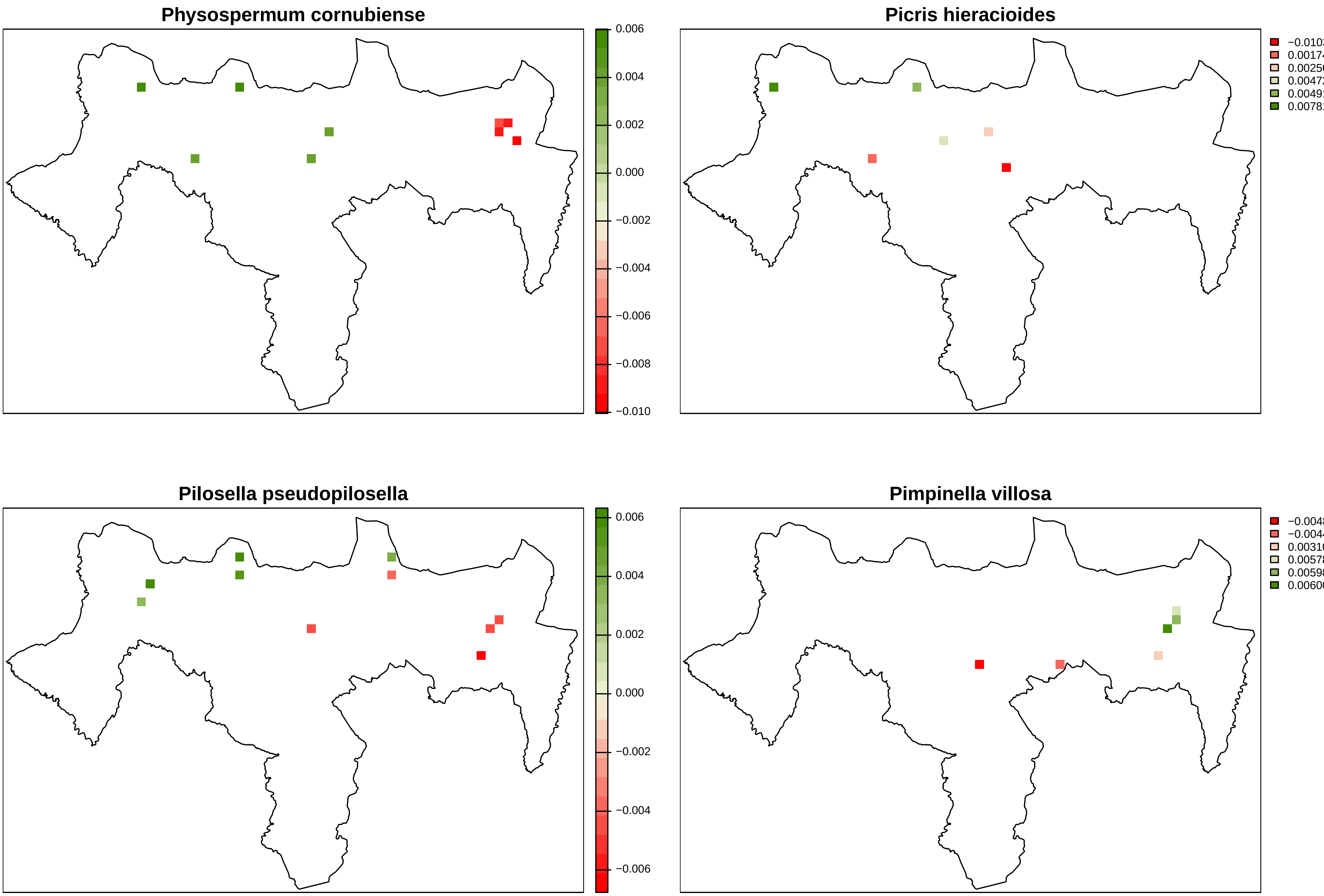

Physospermum cornubiense
0.006
0.004
0.002
0.000
−0.002
−0.004
−0.006
−0.008
−0.010
Picris hieracioides
−0.010
0.0017
0.0025
0.0047
0.0049
0.0078
Pilosella pseudopilosella
0.006
0.004
0.002
0.000
−0.002
−0.004
−0.006
Pimpinella villosa
−0.004
−0.004
0.0031
0.0057
0.0059
0.0060

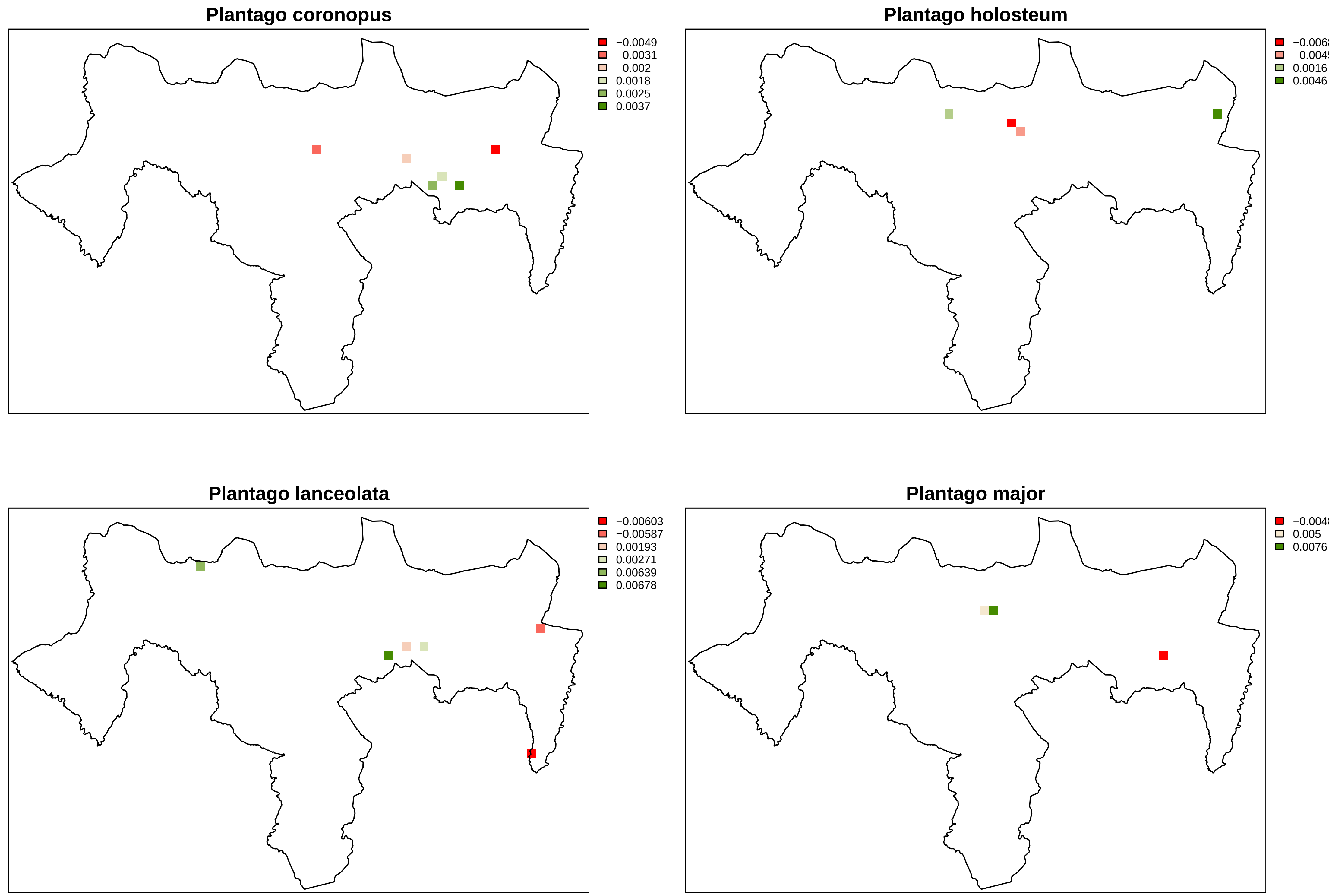
Plantago coronopus
−0.0049
−0.0031
−0.002
0.0018
0.0025
0.0037
Plantago holosteum
0.0016
0.0046
Plantago lanceolata
−0.00603
−0.00587
0.00193
0.00271
0.00639
0.00678
Plantago major
0.005
0.0076

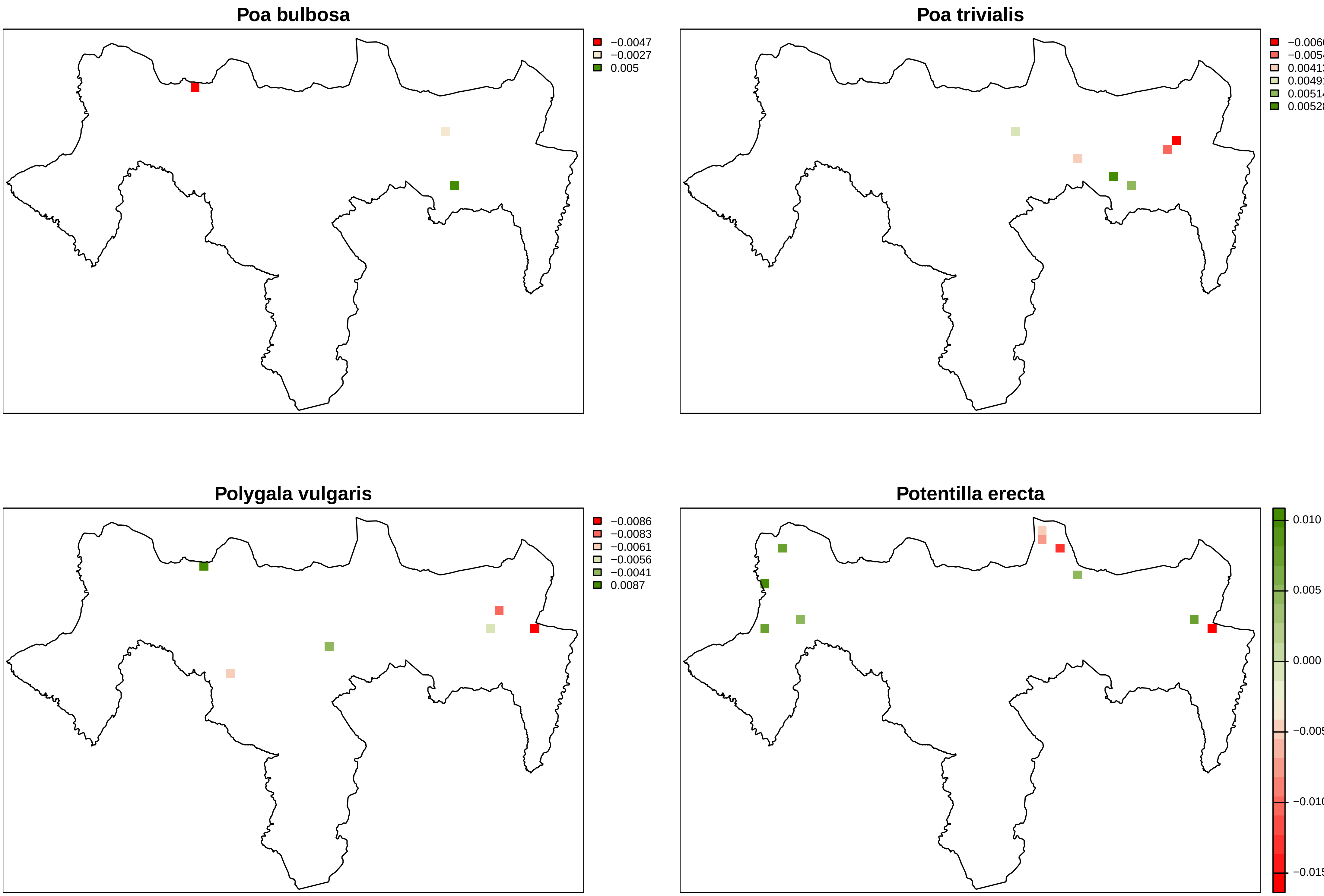

Poa bulbosa
−0.0047
−0.0027
0.005
Poa trivialis
Polygala vulgaris
−0.0086
−0.0083
−0.0061
−0.0056
−0.0041
0.0087
Potentilla erecta
0.010
0.005
0.000

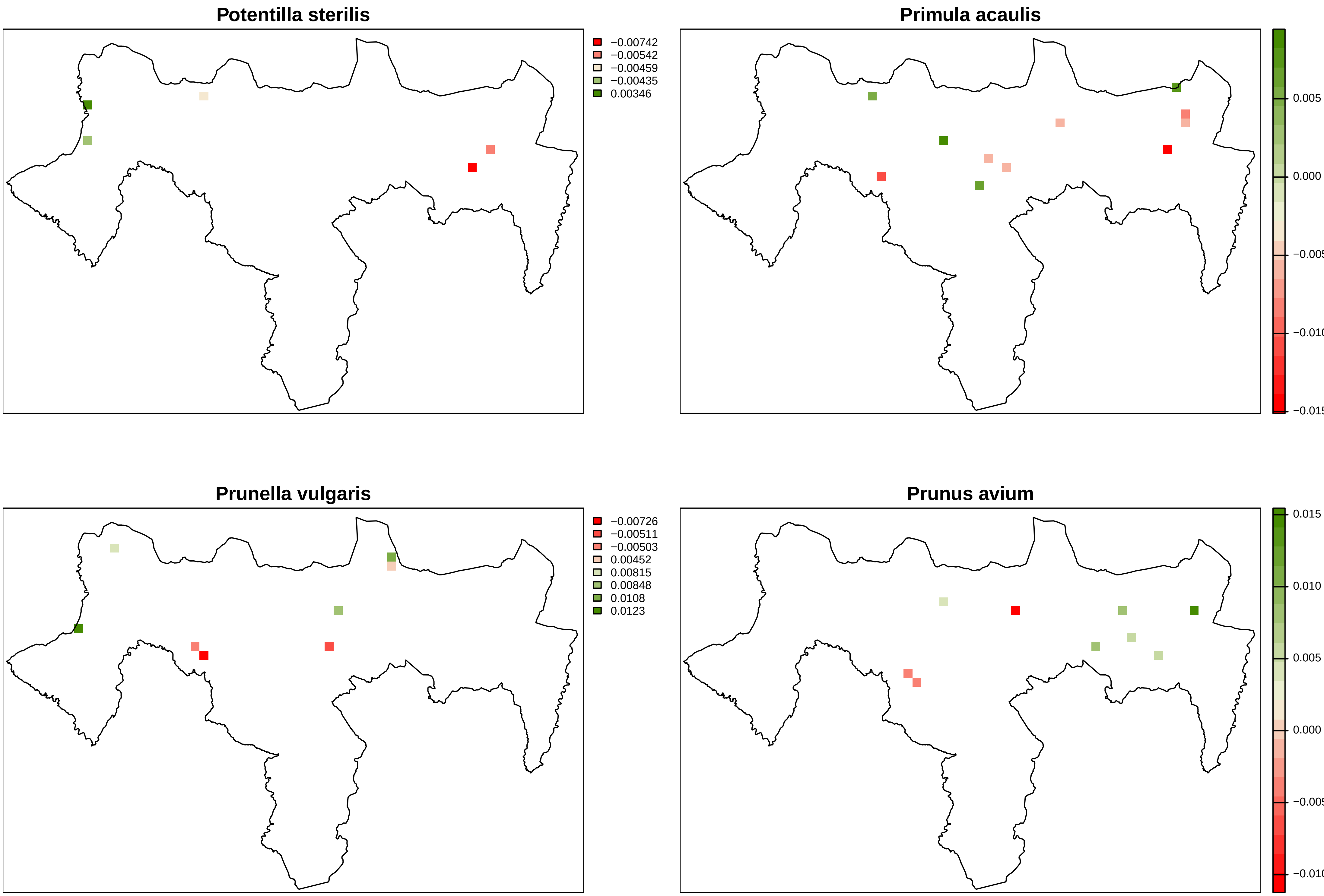
Potentilla sterilis
−0.00742
−0.00542
−0.00459
−0.00435
0.00346
Primula acaulis
0.005
0.000
−0.005
−0.010
−0.015
Prunella vulgaris
−0.00726
−0.00511
−0.00503
0.00452
0.00815
0.00848
0.0108
0.0123
Prunus avium
0.015
0.010
0.005
0.000
−0.005
−0.010

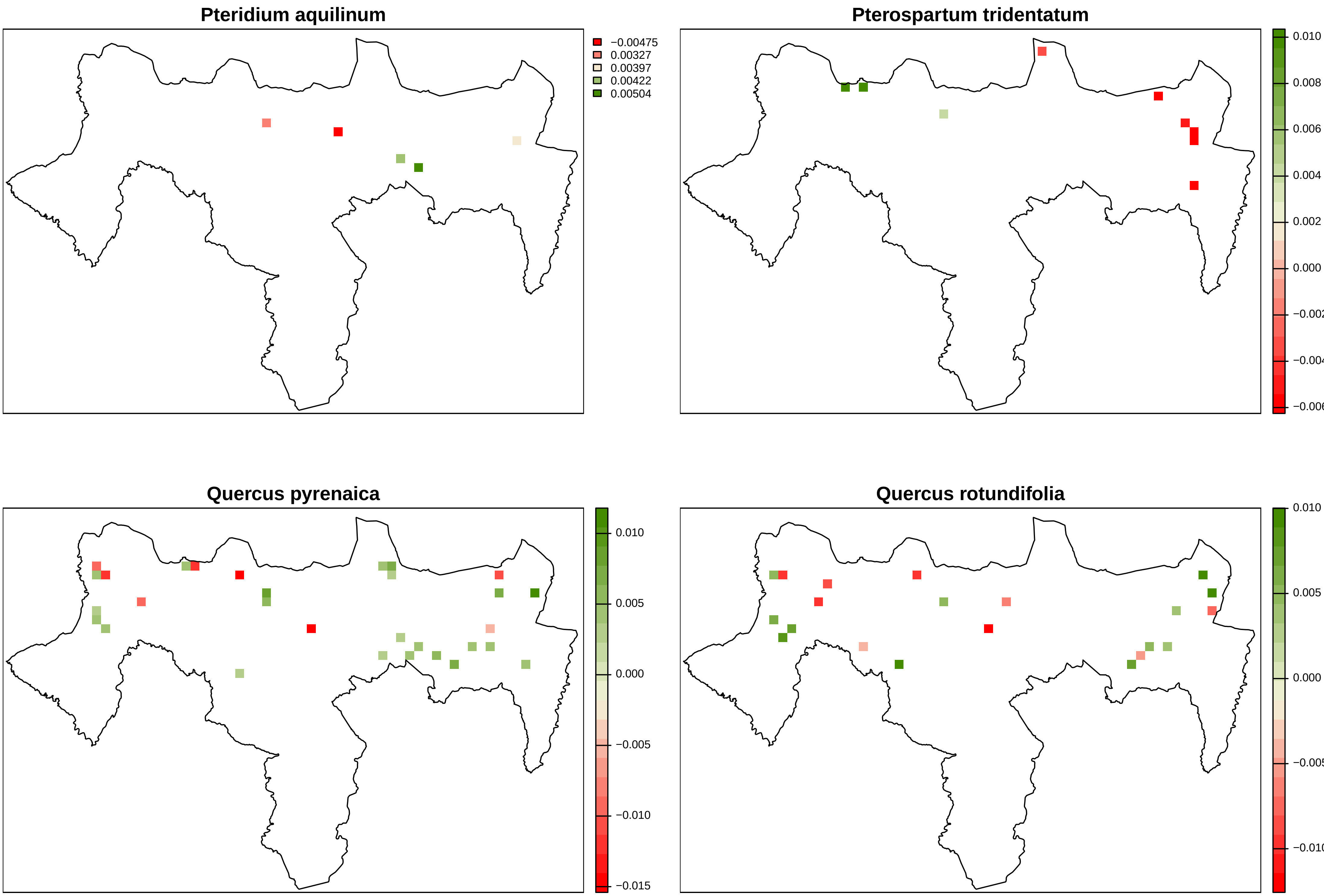
Pteridium aquilinum
−0.00475
0.00327
0.00397
0.00422
0.00504
Pterospartum tridentatum
0.010
0.008
0.006
0.004
0.002
0.000
Quercus pyrenaica
0.010
0.005
0.000
−0.005
−0.010
−0.015
Quercus rotundifolia
0.010
0.005
0.000

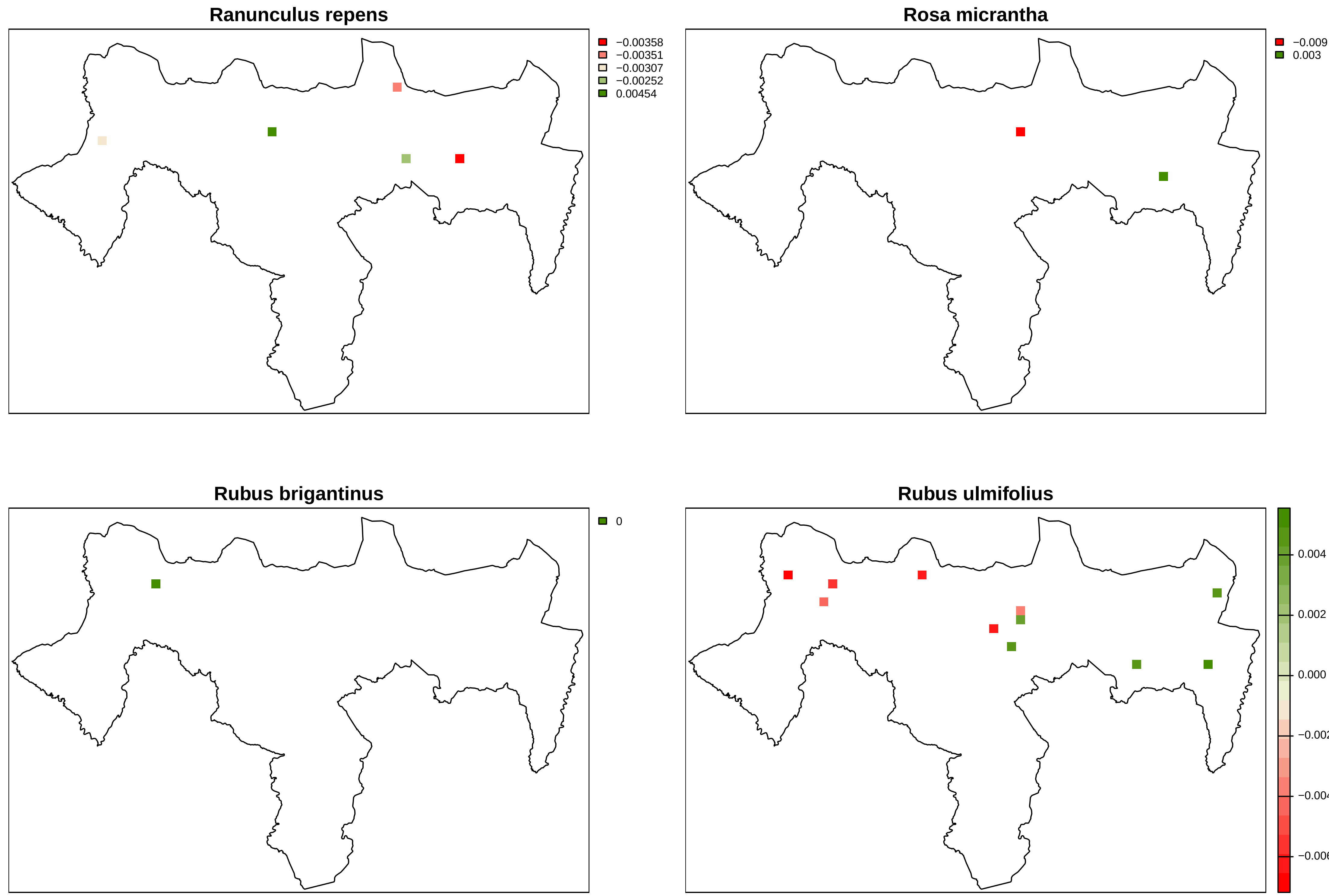
Ranunculus repens
−0.00358
−0.00351
−0.00307
−0.00252
0.00454
Rosa micrantha
−0.009
0.003
Rubus brigantinus
0
Rubus ulmifolius
0.004
0.002
0.000

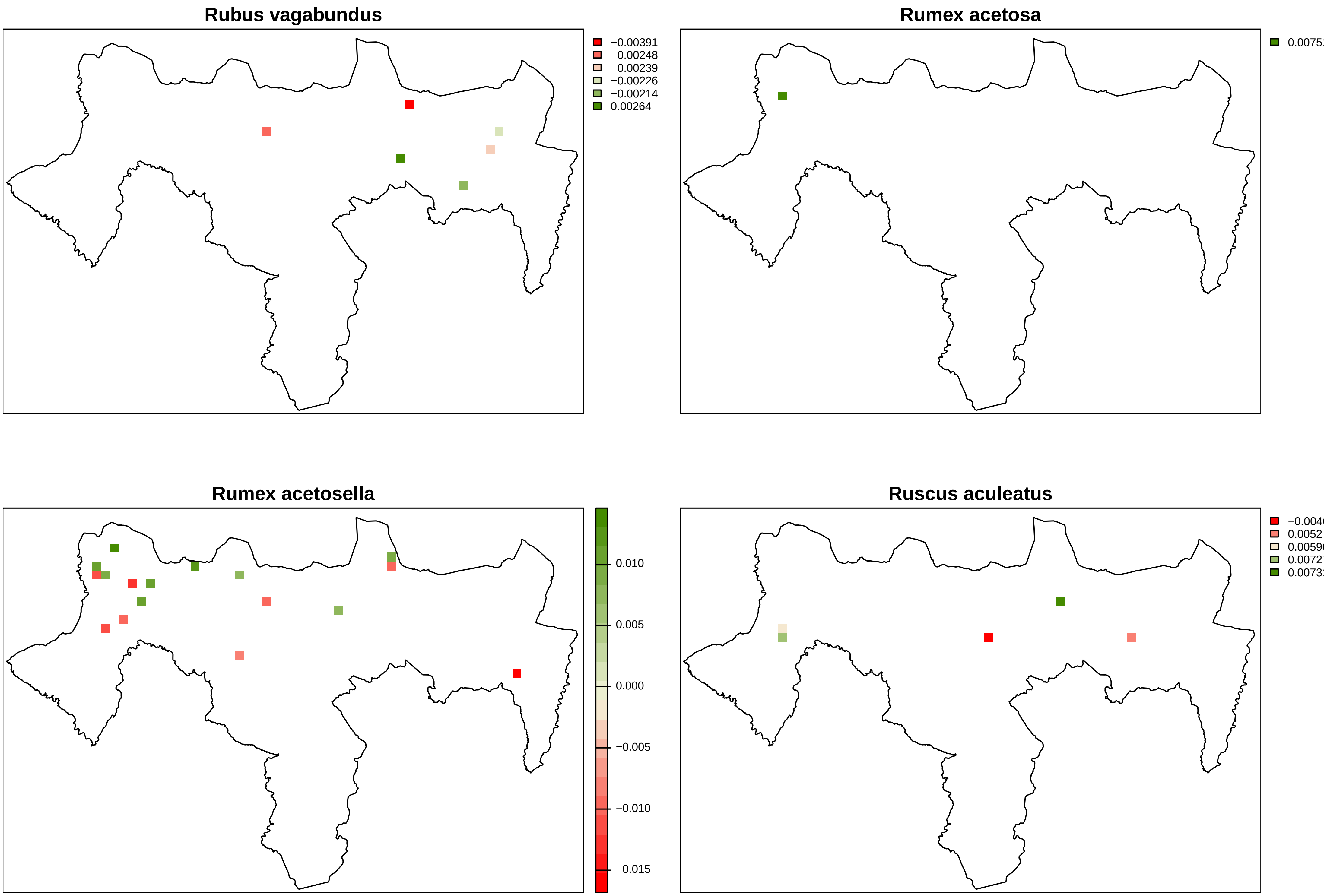

Rubus vagabundus
−0.00391
−0.00248
−0.00239
−0.00226
−0.00214
0.00264
Rumex acetosa
Rumex acetosella
0.010
0.005
0.000
−0.005
−0.010
−0.015
Ruscus aculeatus

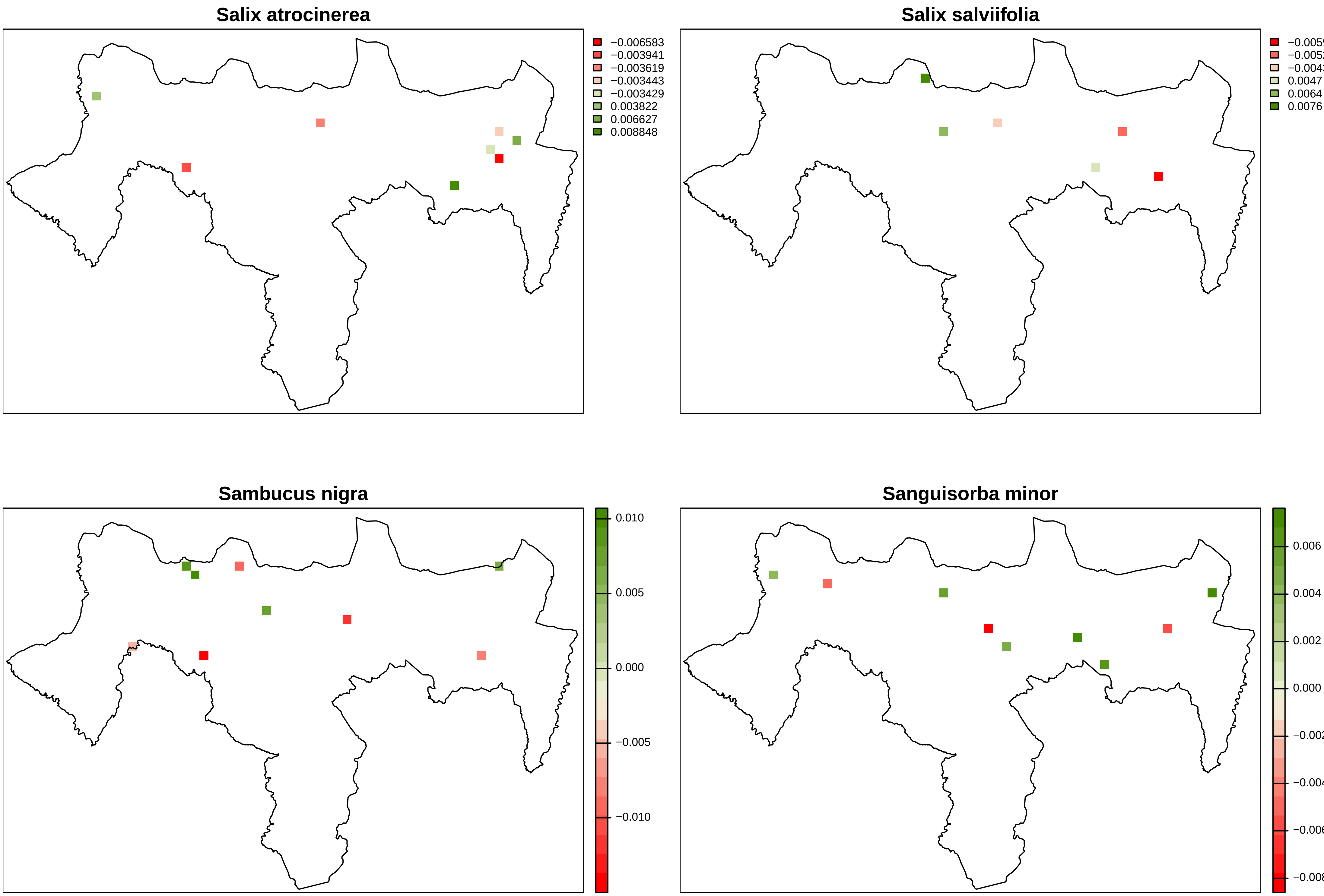
Salix atrocinerea
−0.006583
−0.003941
−0.003619
−0.003443
−0.003429
0.003822
0.006627
0.008848
Salix salviifolia
0.0047
Sambucus nigra
0.010
0.005
0.000
−0.005
−0.010
Sanguisorba minor
0.006
0.004
0.002
0.000

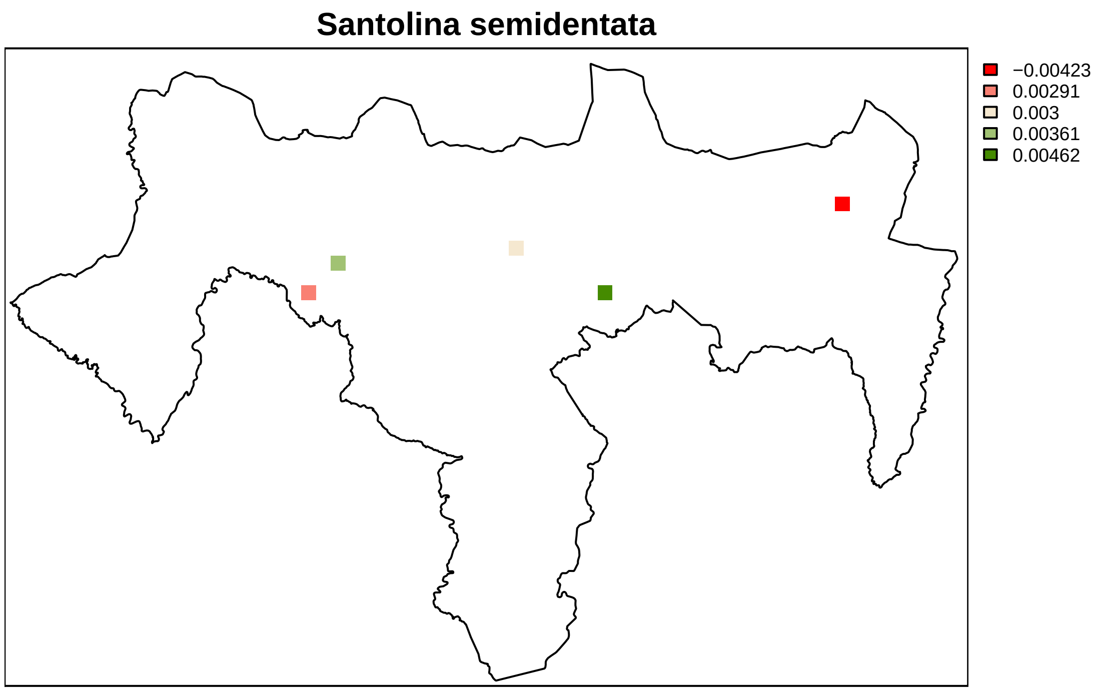
Santolina semidentata
−0.00423
0.00291
0.003
0.00361
0.00462

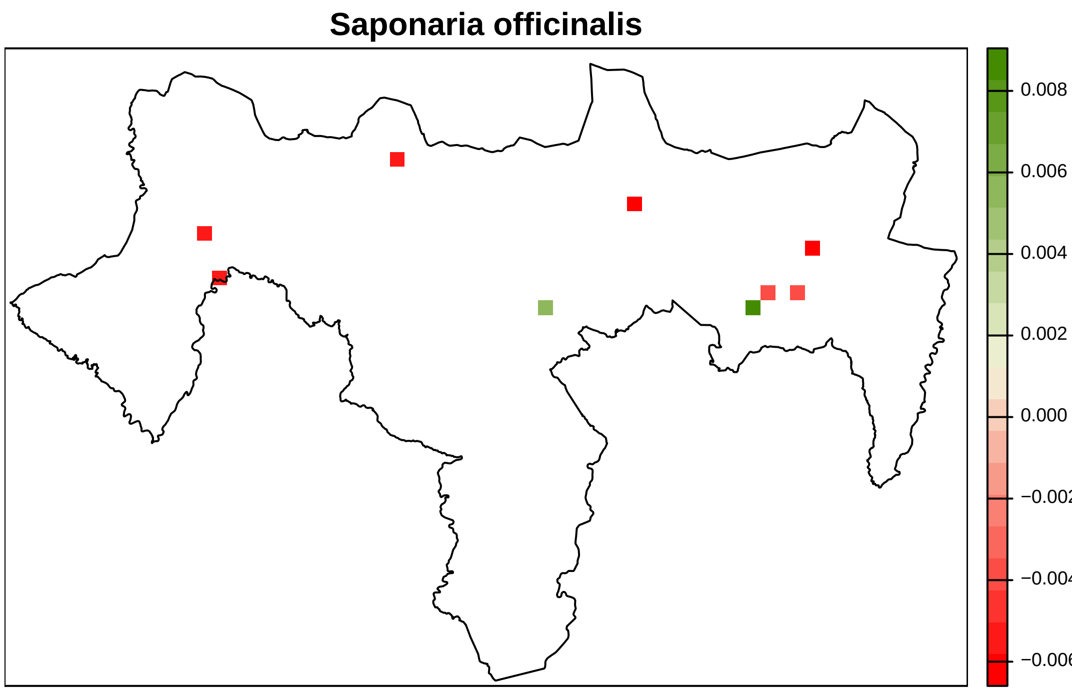
Saponaria officinalis
0.008
0.006
0.004
0.002
0.000
−0.002
−0.004
−0.006

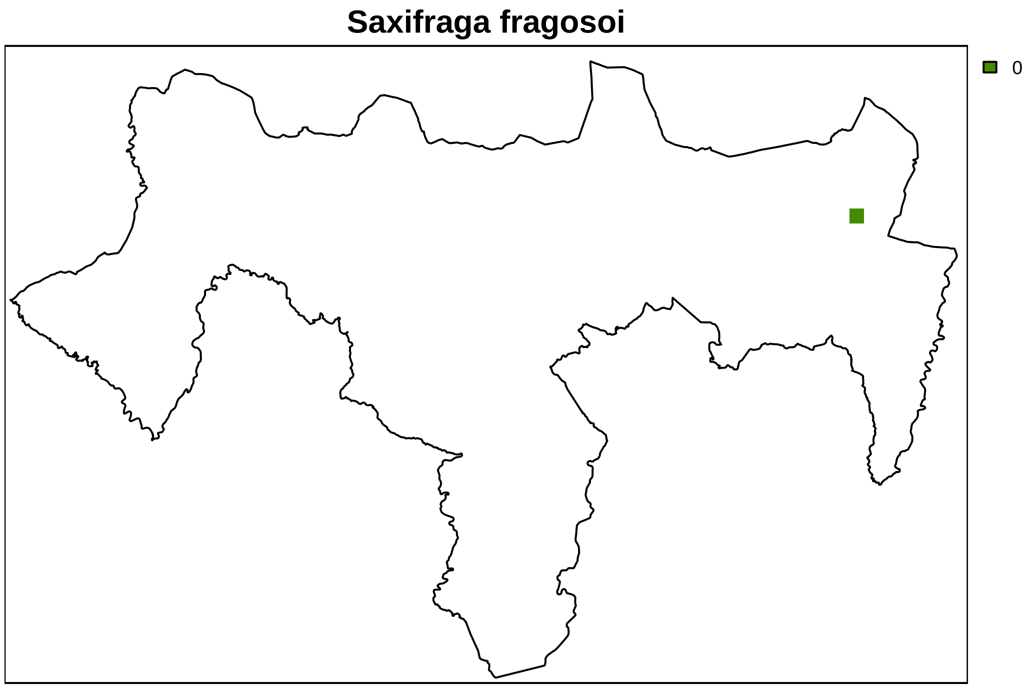
Saxifraga fragosoi
0

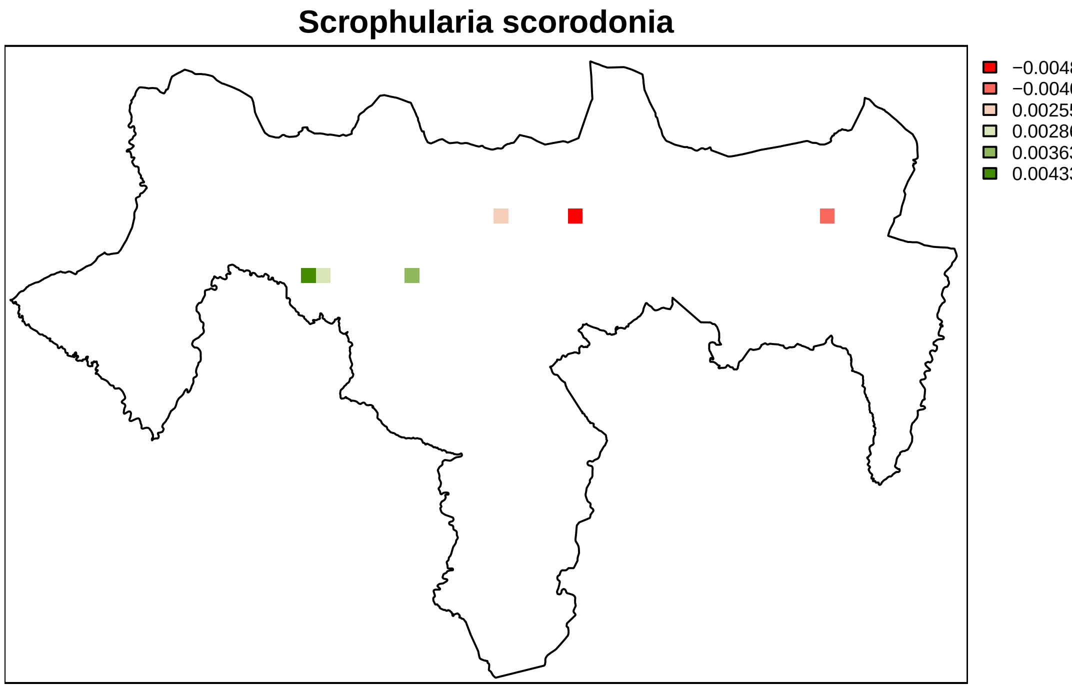
Scrophularia scorodonia
−0.004
−0.004
0.0025
0.0028
0.0036
0.0043

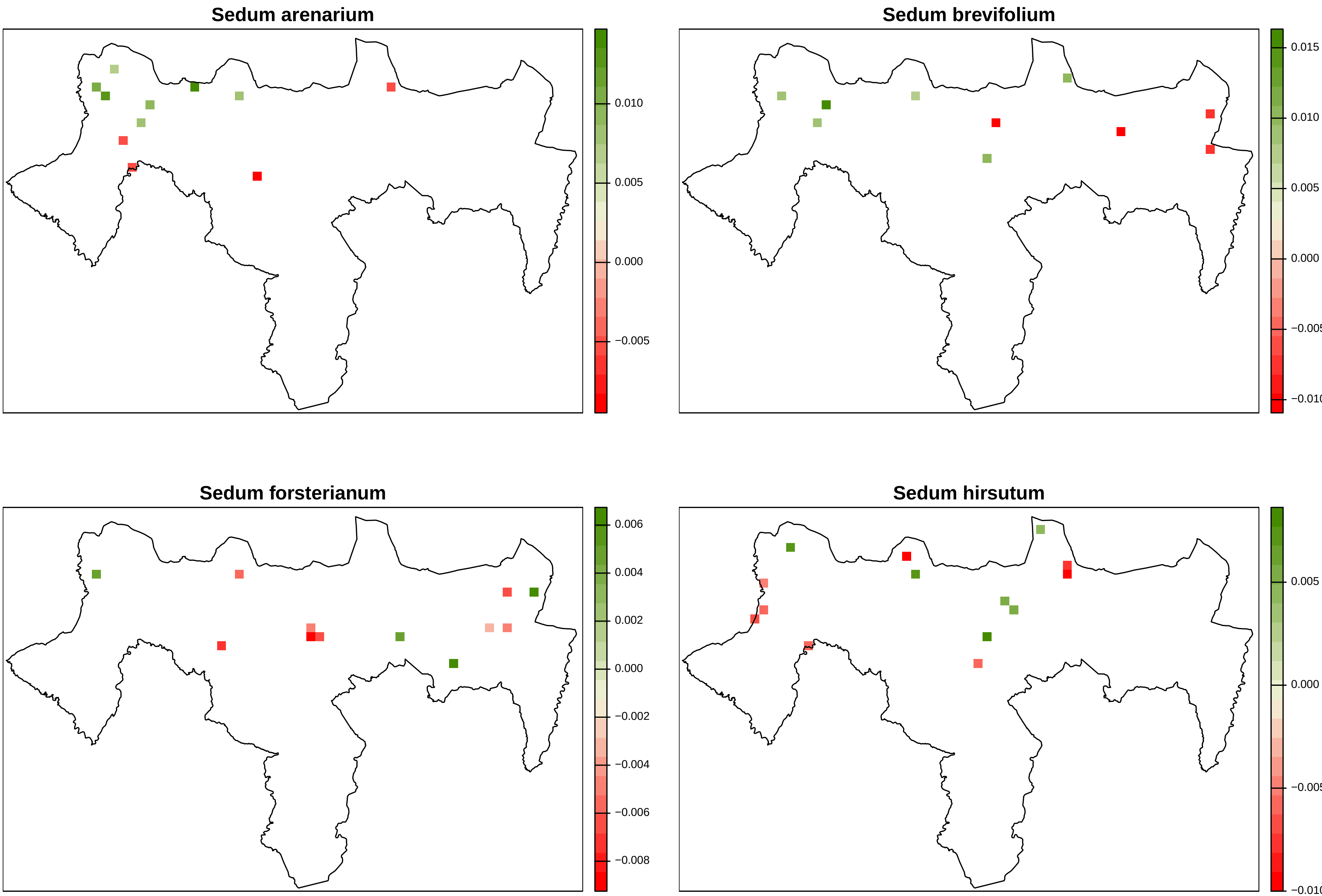
Sedum arenarium
0.010
0.005
0.000
−0.005
Sedum brevifolium
0.015
0.010
0.005
0.000
Sedum forsterianum
0.006
0.004
0.002
0.000
−0.002
−0.004
−0.006
−0.008
Sedum hirsutum
0.005
0.000

Senecio jacobaea

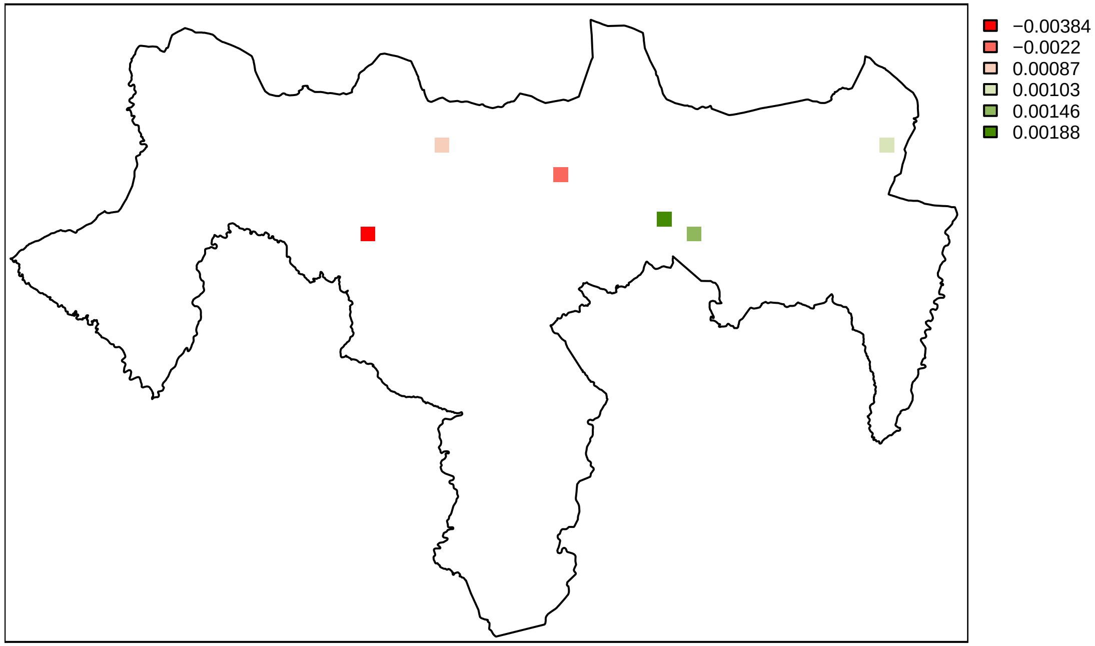

Senecio sylvaticus

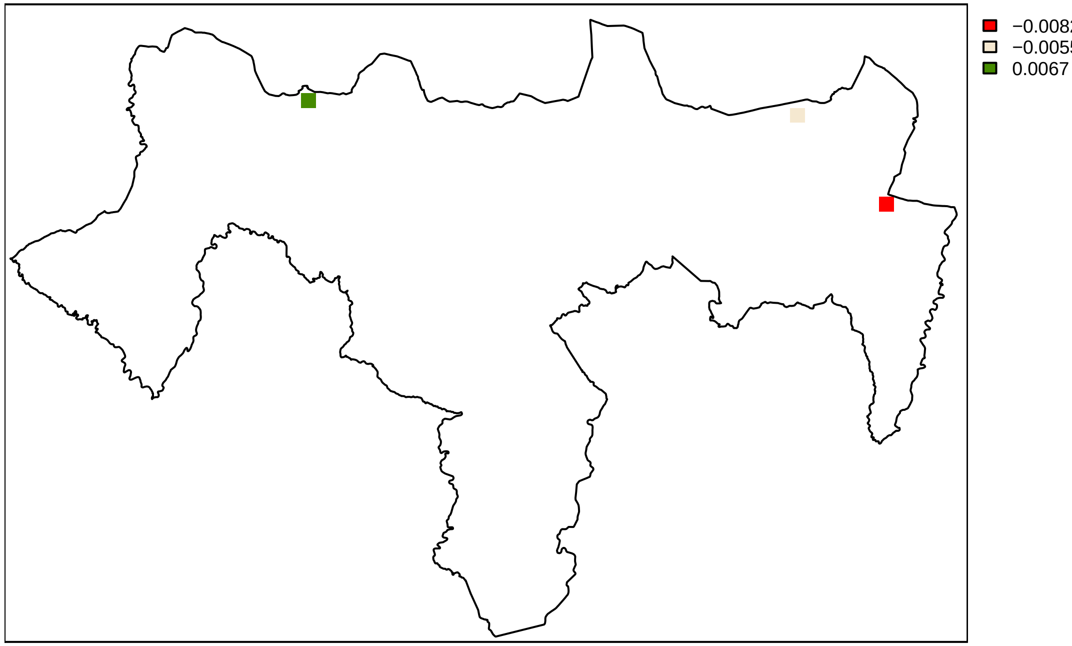

Sonchus asper

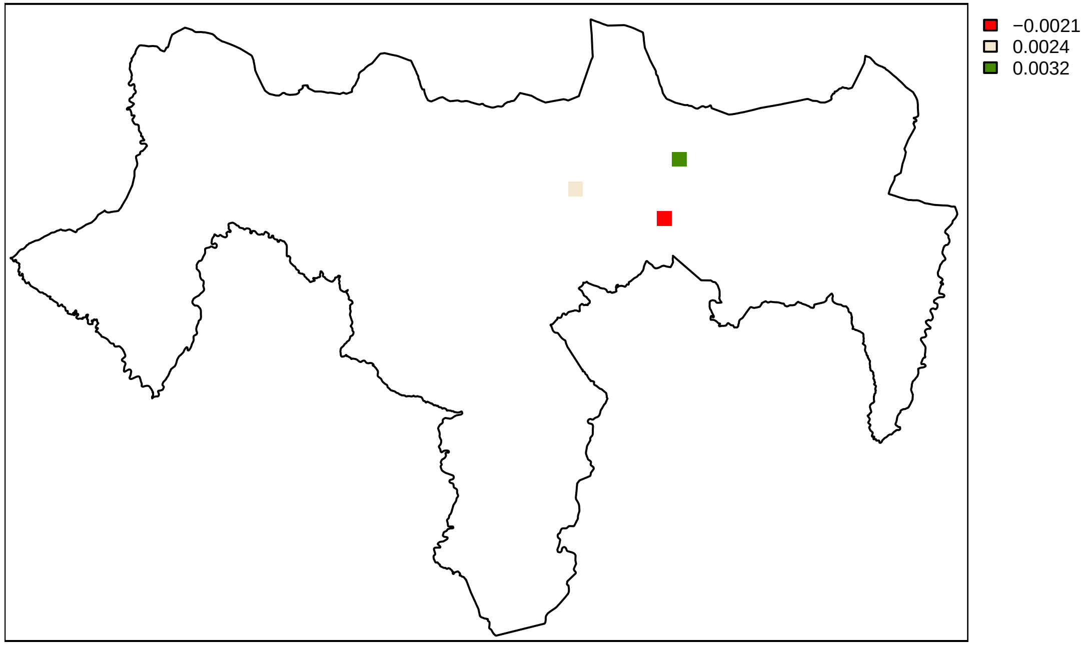

Stachys sylvatica

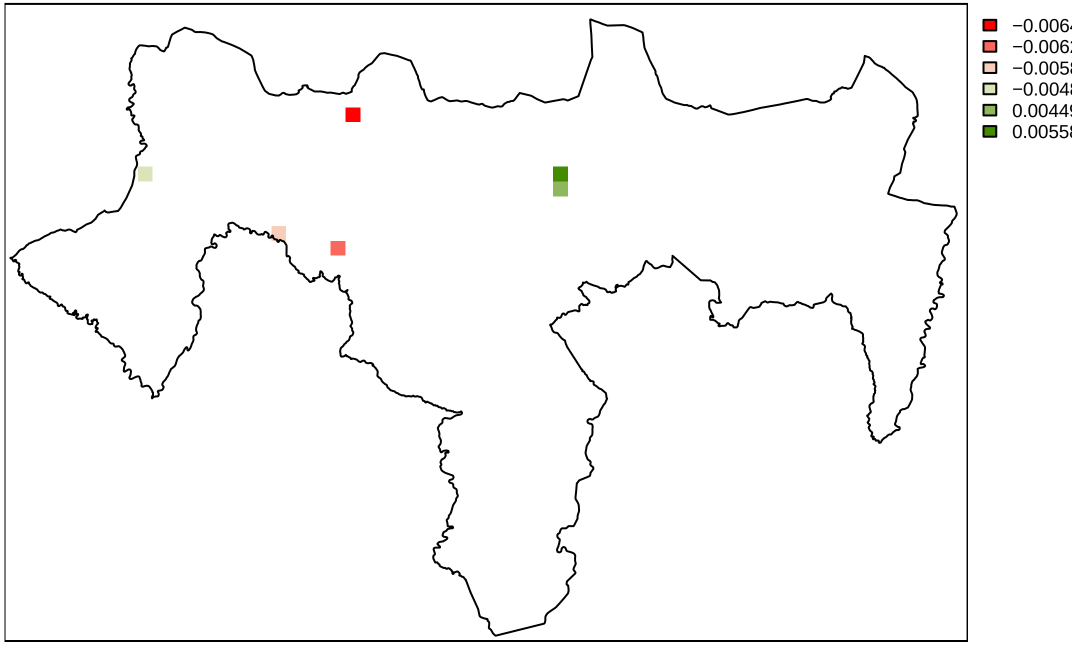

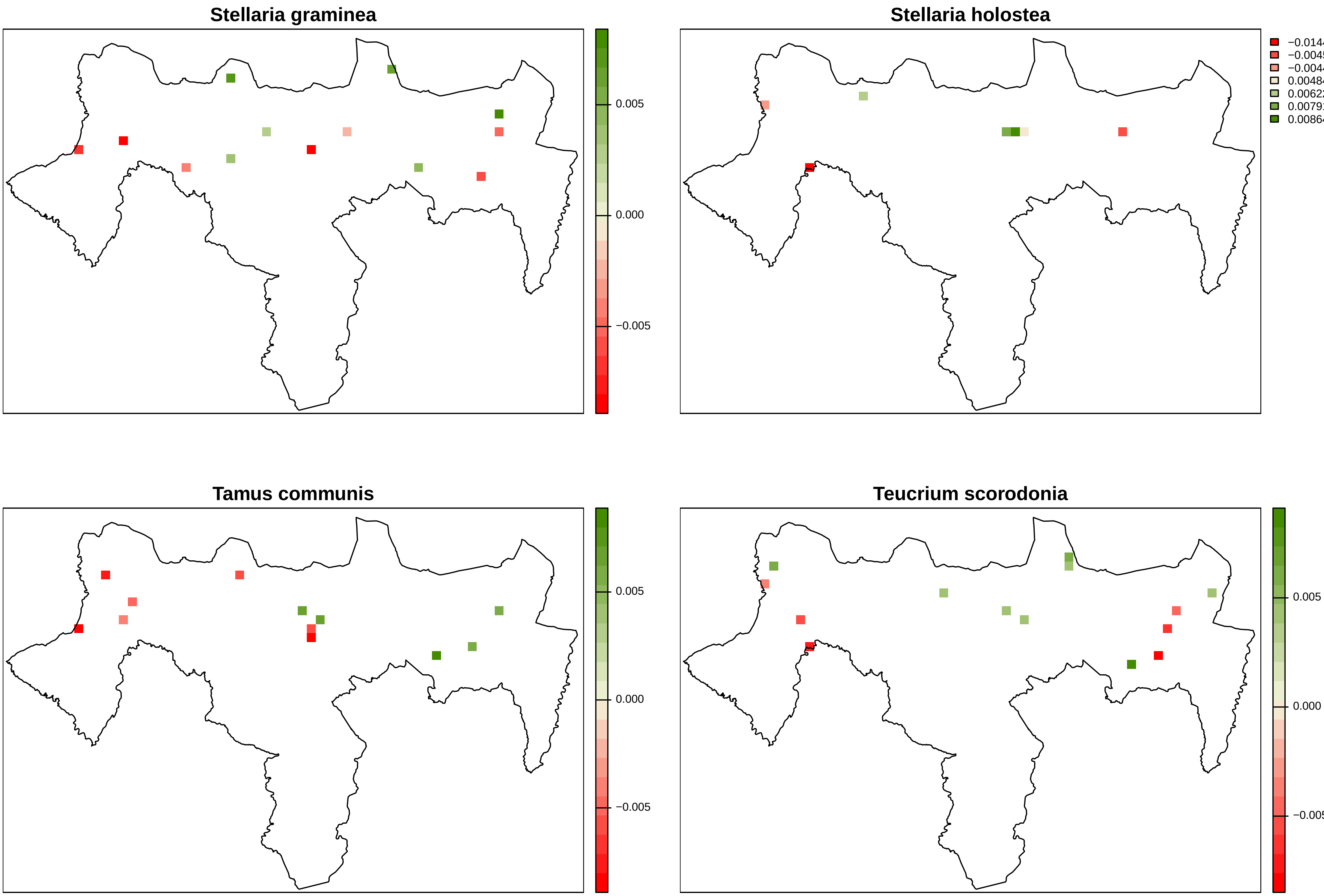

Stellaria graminea
0.005
0.000
−0.005
Stellaria holostea
Tamus communis
0.005
0.000
−0.005
Teucrium scorodonia
0.005
0.000

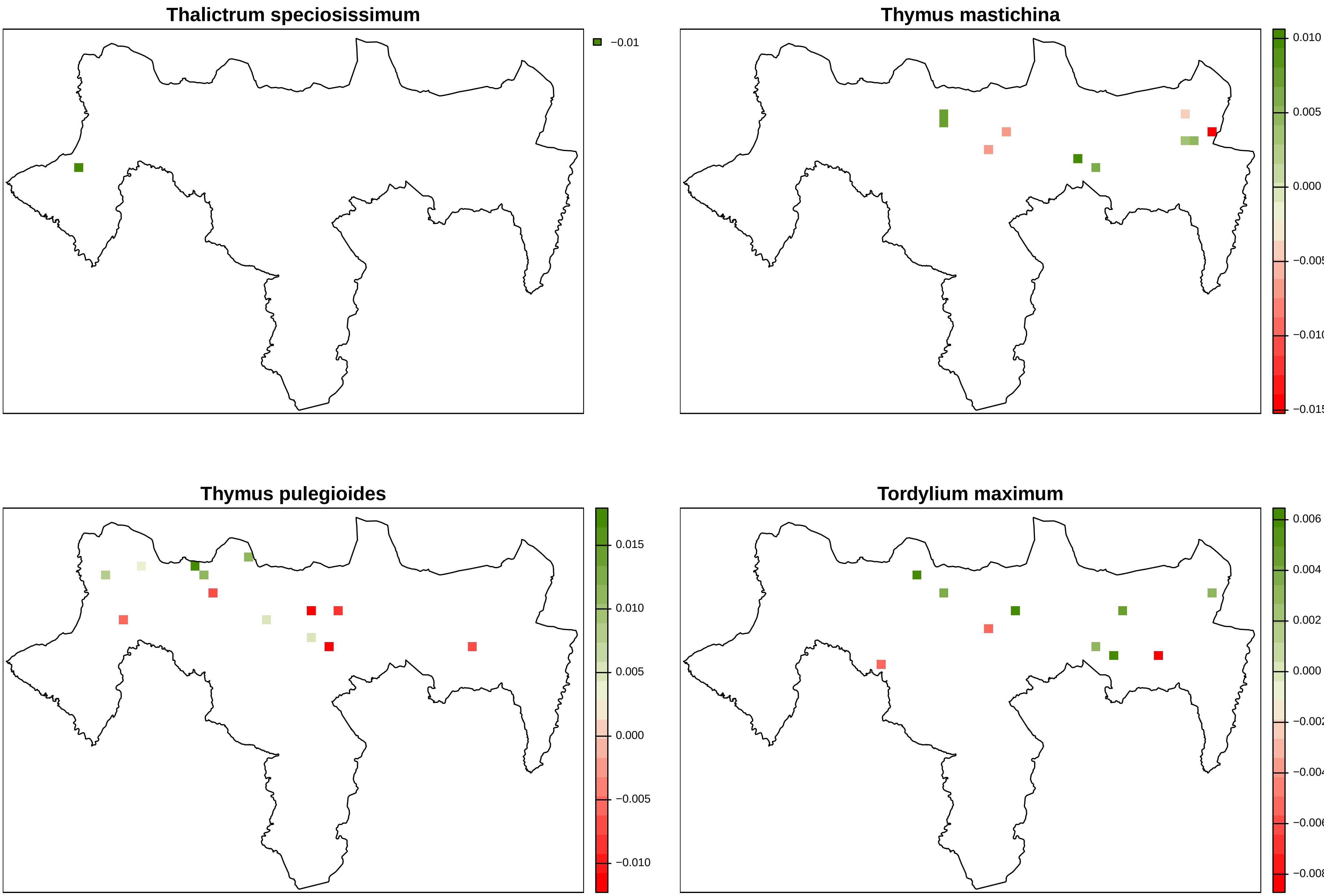
Thalictrum speciosissimum
−0.01
Thymus mastichina
0.010
0.005
0.000
Thymus pulegioides
0.015
0.010
0.005
0.000
−0.005
−0.010
Tordylium maximum
0.006
0.004
0.002
0.000

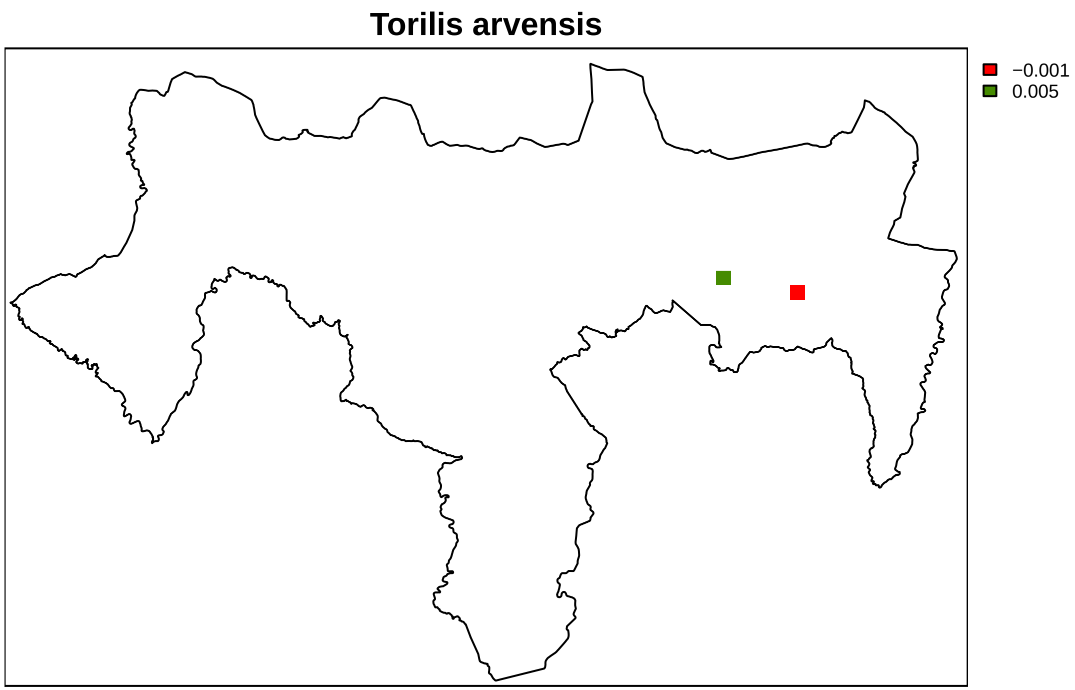
Torilis arvensis
−0.001
0.005

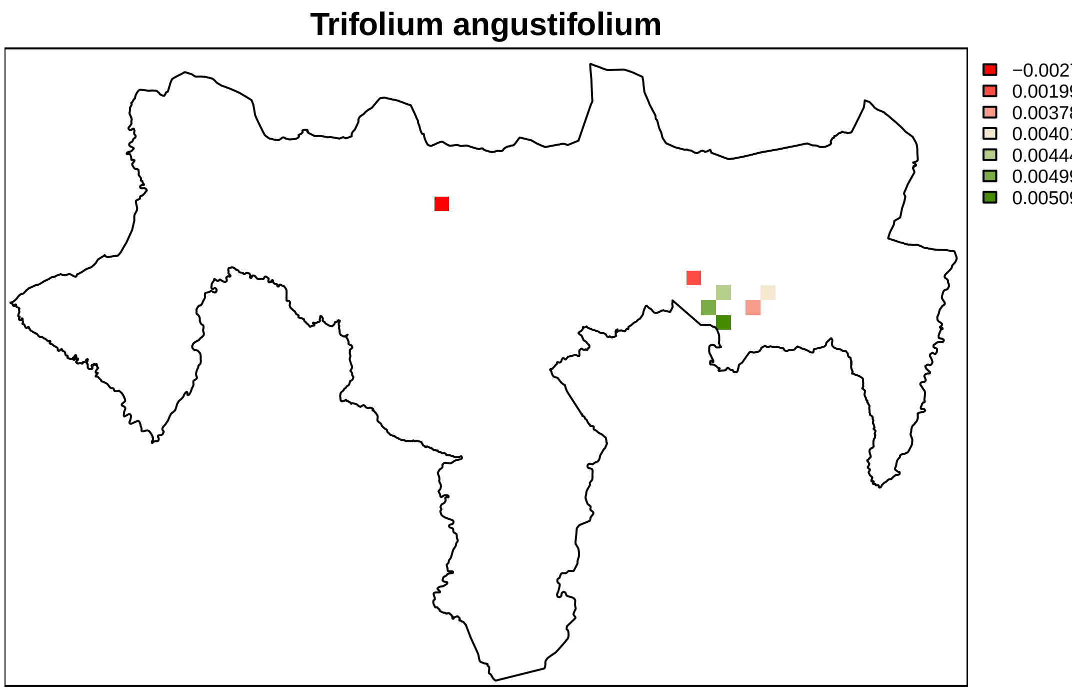
Trifolium angustifolium
−0.002
0.0019
0.0037
0.0040
0.0044
0.0049
0.0050

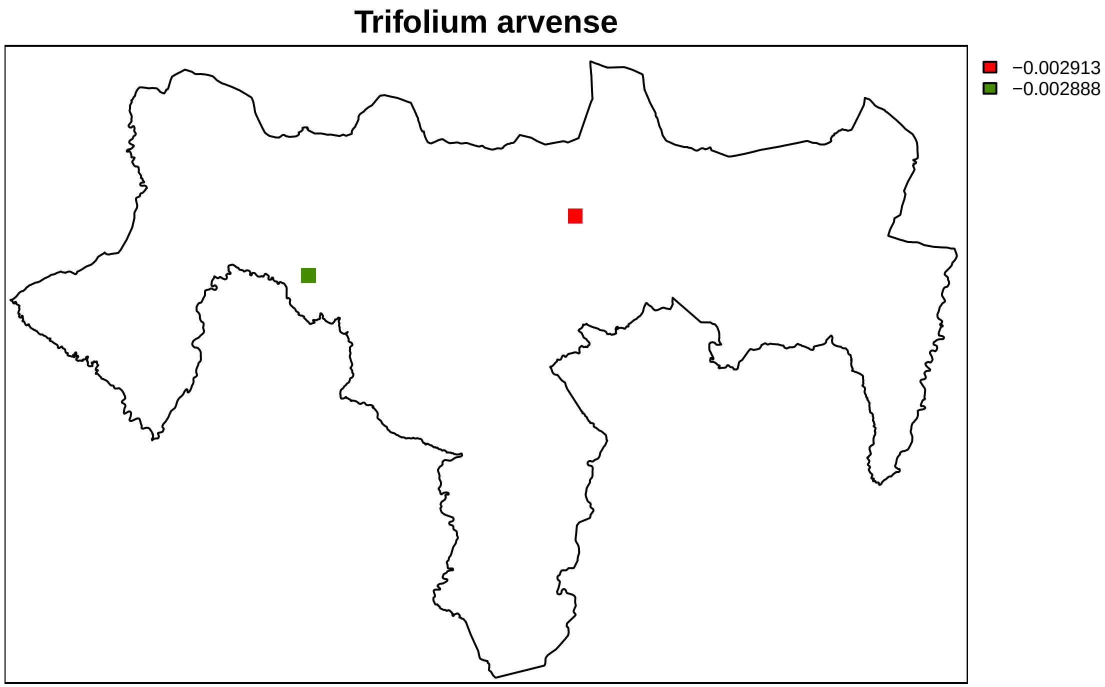
Trifolium arvense
−0.002913
−0.002888

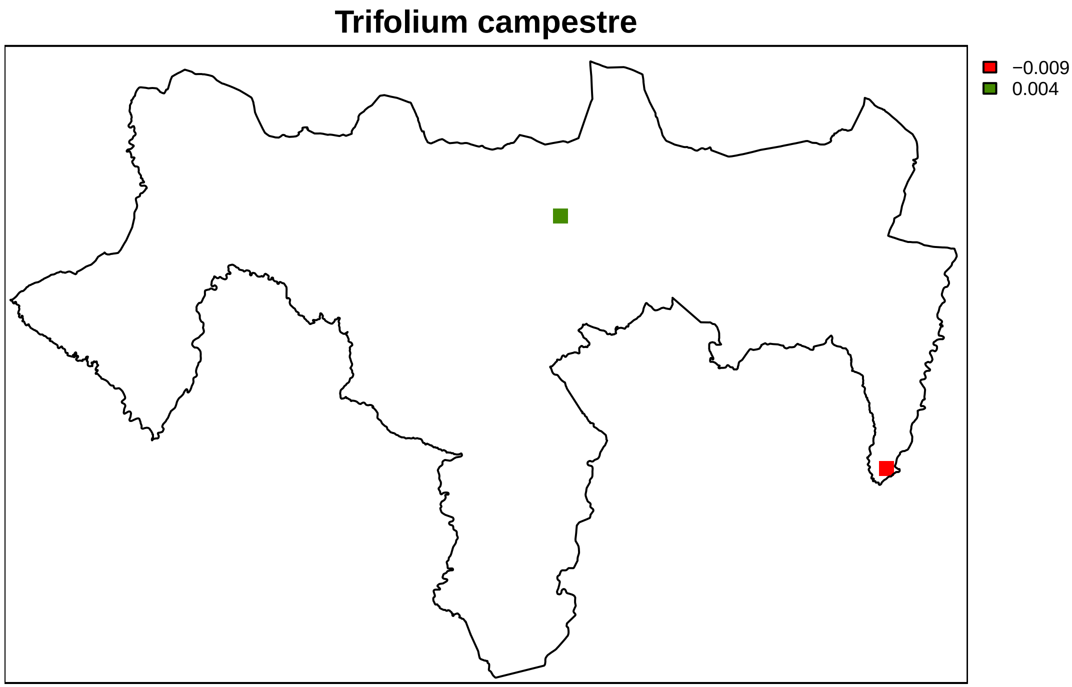
Trifolium campestre
−0.009
0.004

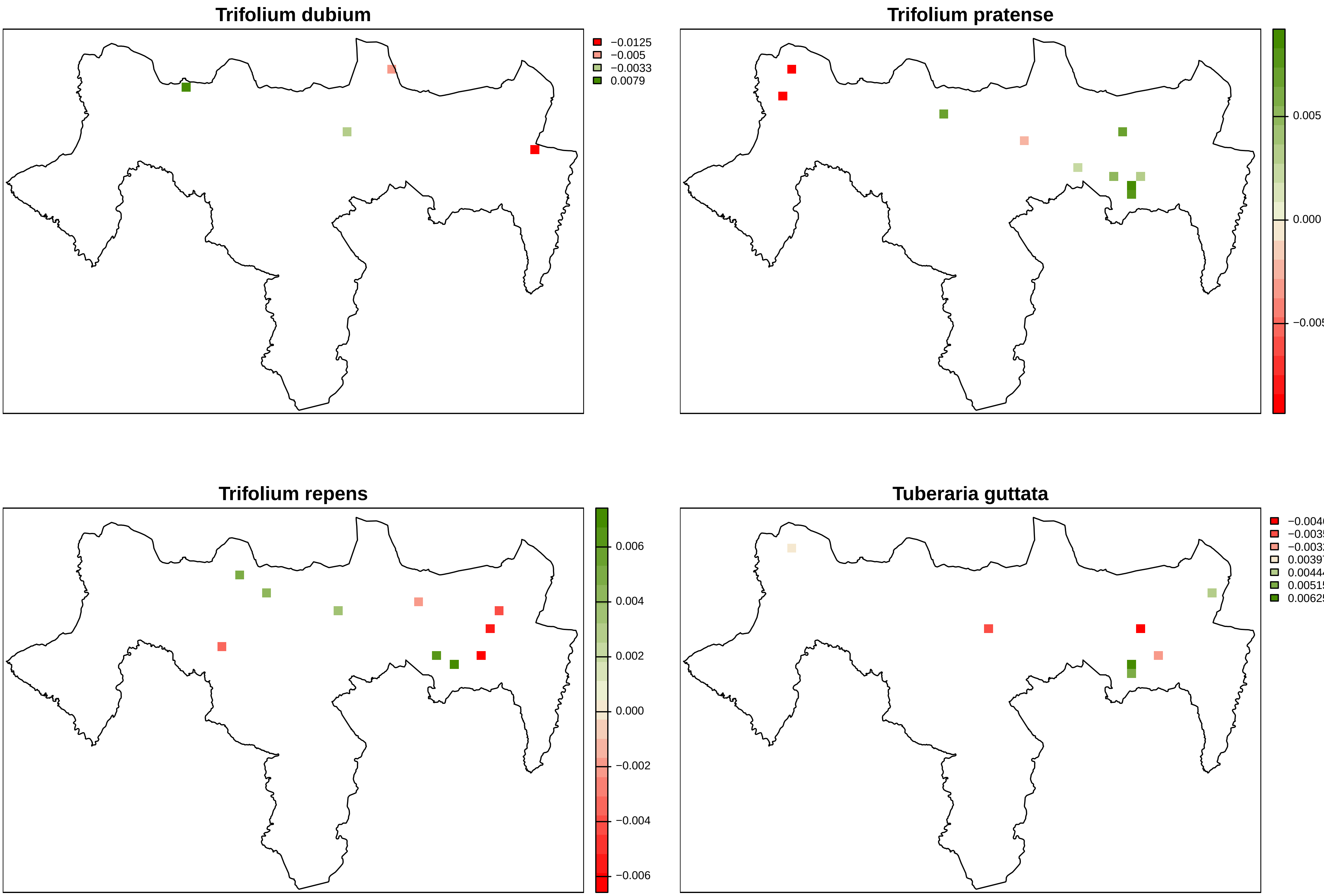
Trifolium dubium
−0.0125
−0.005
−0.0033
0.0079
Trifolium pratense
0.005
0.000
Trifolium repens
0.006
0.004
0.002
0.000
−0.002
−0.004
−0.006
Tuberaria guttata

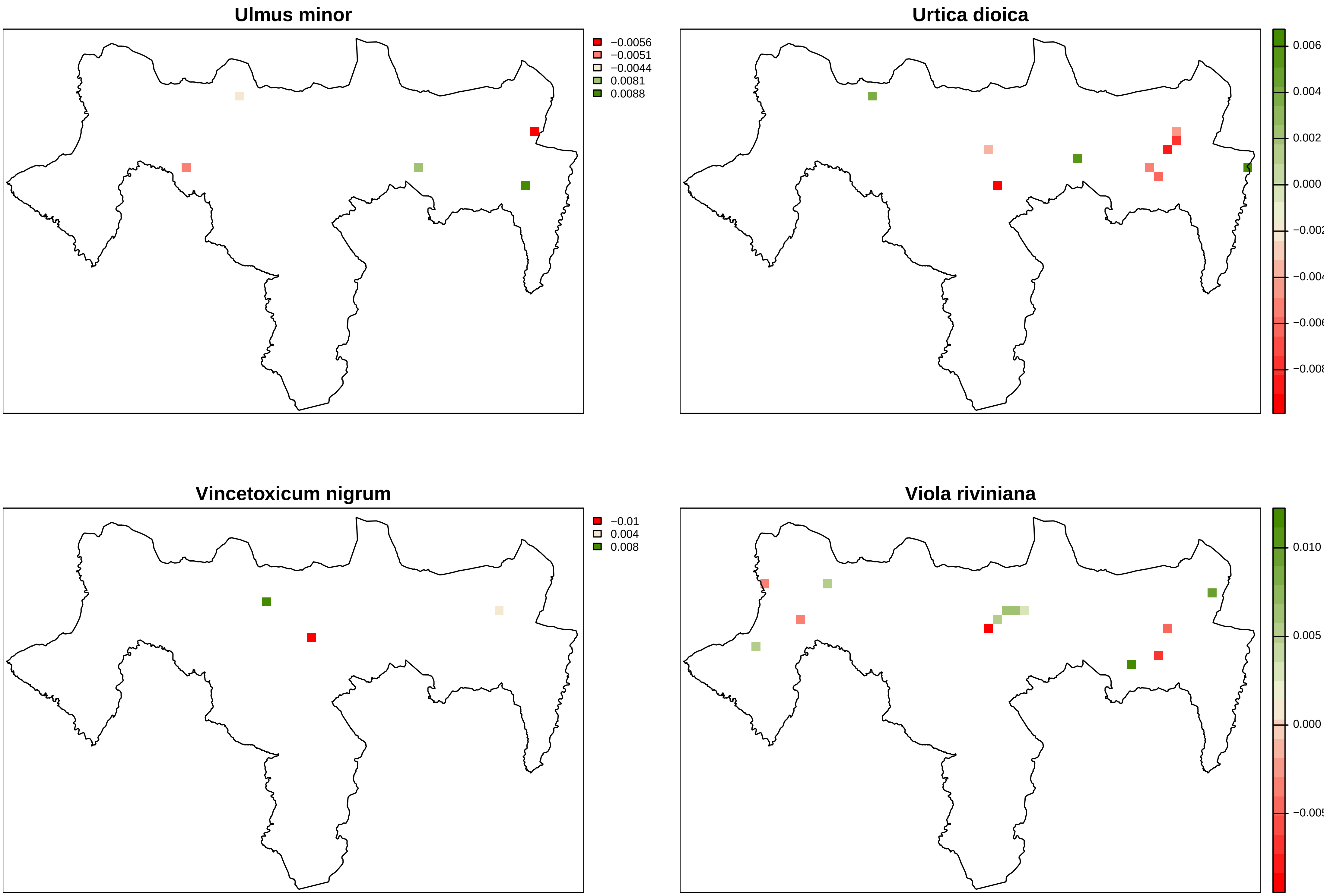
Ulmus minor
−0.0056
−0.0051
−0.0044
0.0081
0.0088
Urtica dioica
0.006
0.004
0.002
0.000
Vincetoxicum nigrum
−0.01
0.004
0.008
Viola riviniana
0.010
0.005
0.000

Vulpia muralis

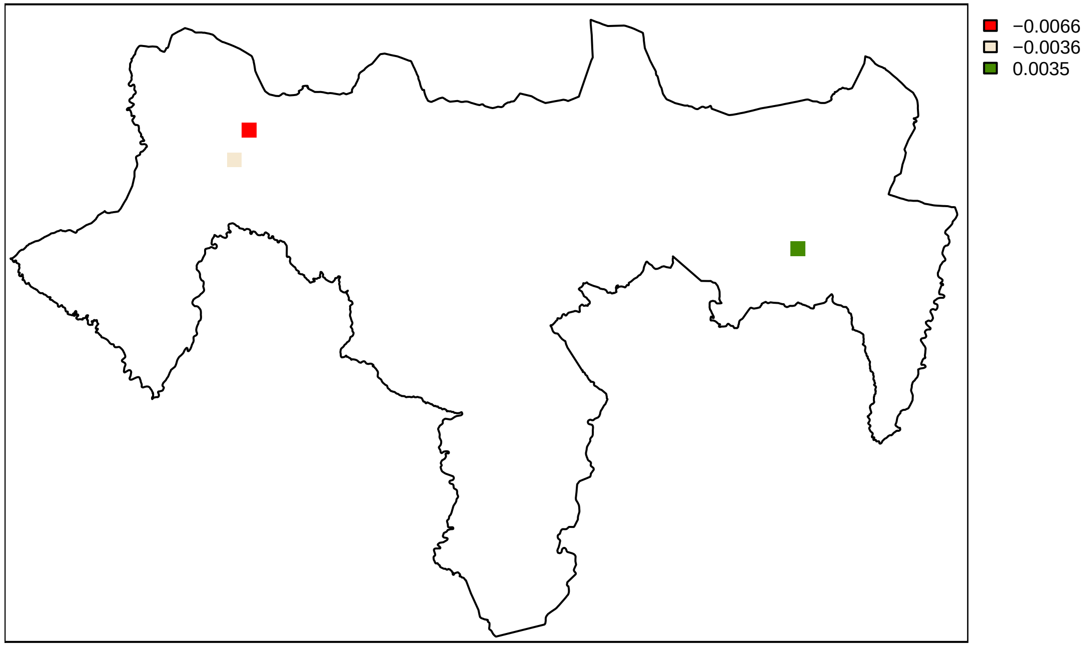

Supplement: Supplementary file 4 — ESM_4 [file 267_2026_2393_MOESM4_ESM.pdf]
