# Supplementary material for: Time-Series Niche Modelling Reveals Declining Tendencies of Habitat Suitability and Ecological Functions in a Mountainous Protected Area
Source: Environ Manage. 2026 Feb 18;76(3):101. doi: 10.1007/s00267-026-02393-5 (PMC12916538; doi:10.1007/s00267-026-02393-5)
Supplement: Supplementary file 5 — ESM_5.A [file 267_2026_2393_MOESM5_ESM.pdf]

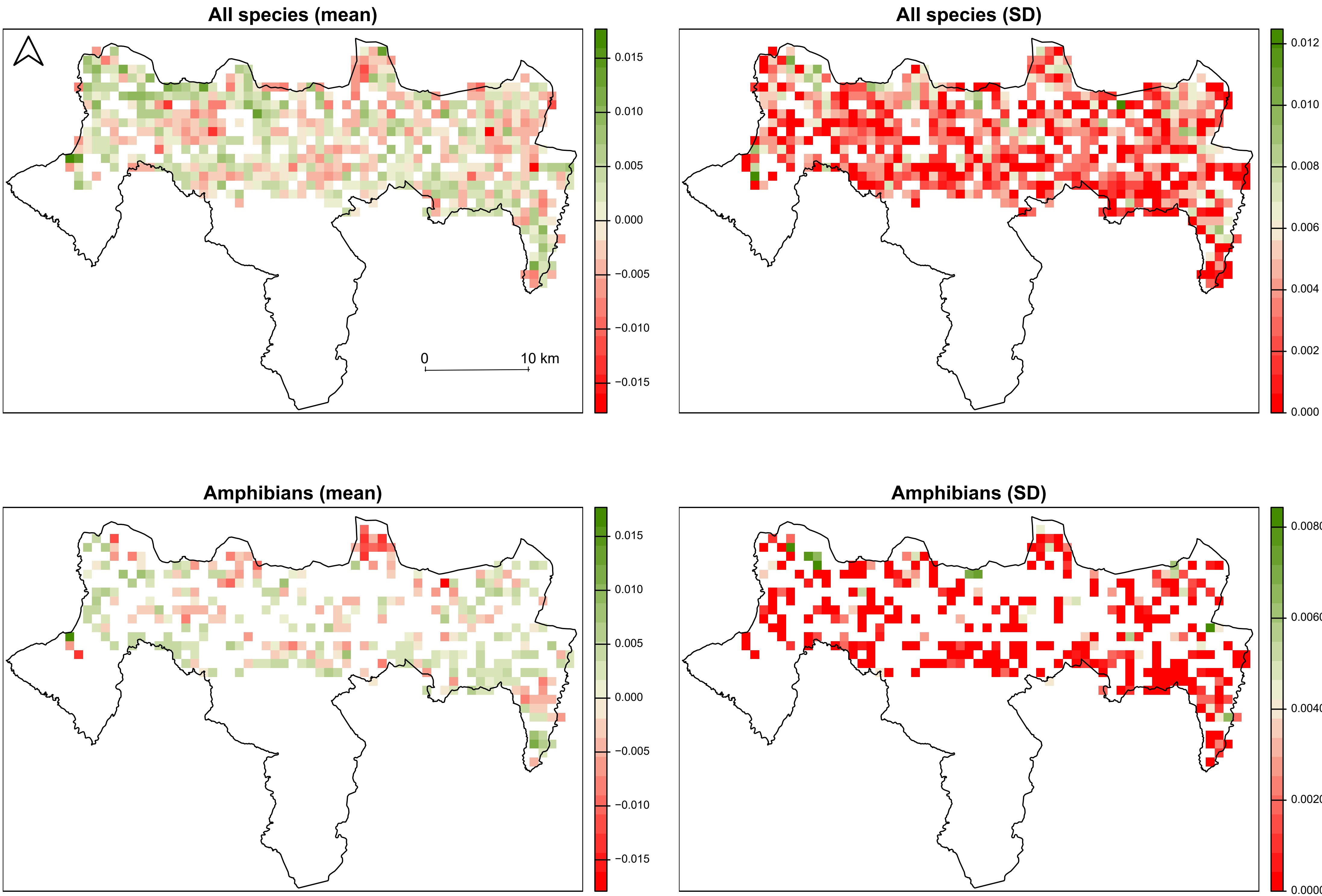
All species (mean)
0.015
0.010
0.005
0.000
−0.005
−0.010
−0.015
0
10 km
All species (SD)
0.012
0.010
0.008
0.006
0.004
0.002
0.000
Amphibians (mean)
0.015
0.010
0.005
0.000
−0.005
−0.010
−0.015
Amphibians (SD)
0.0080
0.0060
0.0040
0.0020
0.0000

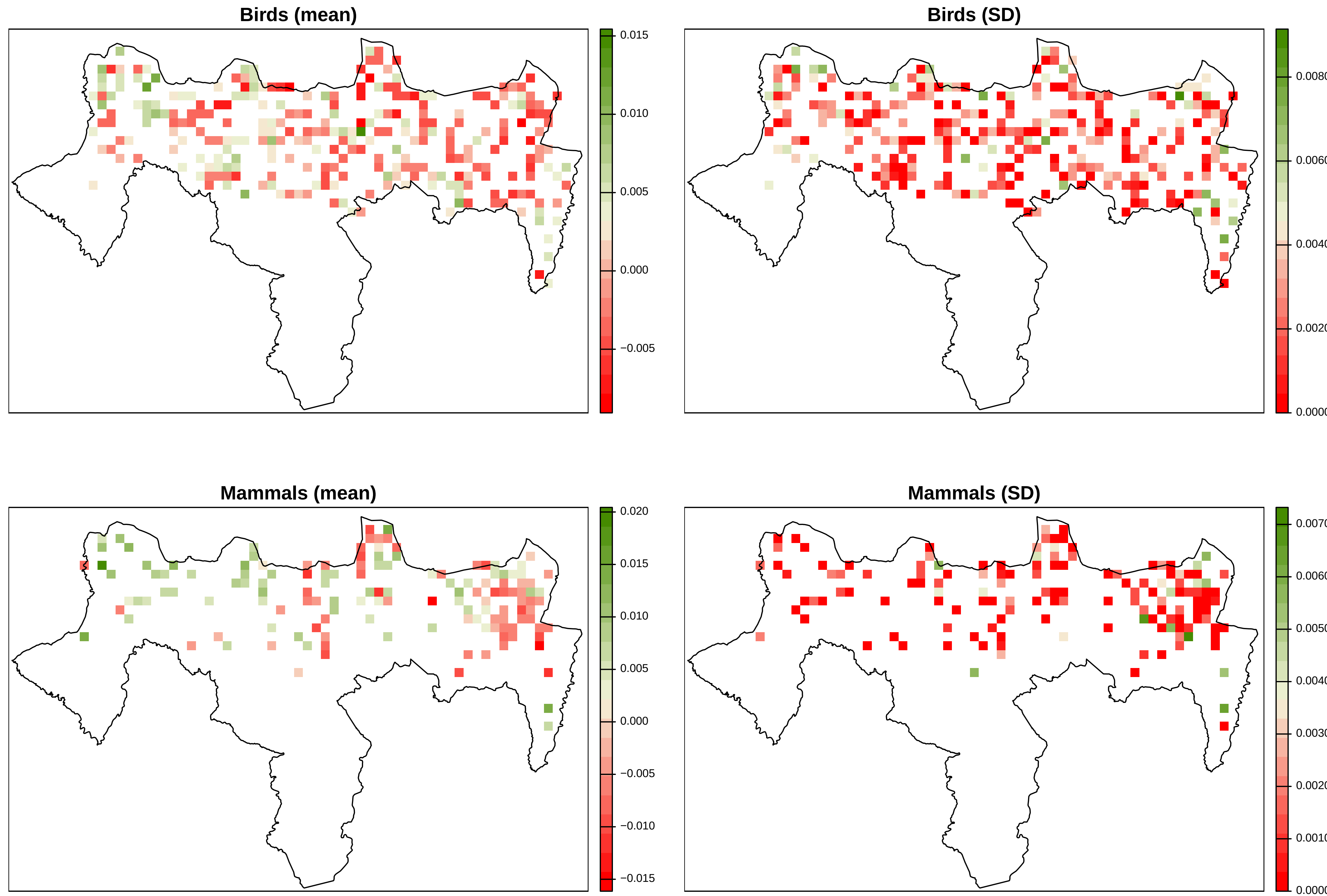

Birds (mean)
0.015
0.010
0.005
0.000
−0.005
Birds (SD)
0.008
0.006
0.004
0.002
0.000
Mammals (mean)
0.020
0.015
0.010
0.005
0.000
−0.005
−0.010
−0.015
Mammals (SD)
0.007
0.006
0.005
0.004
0.003
0.002
0.001
0.000

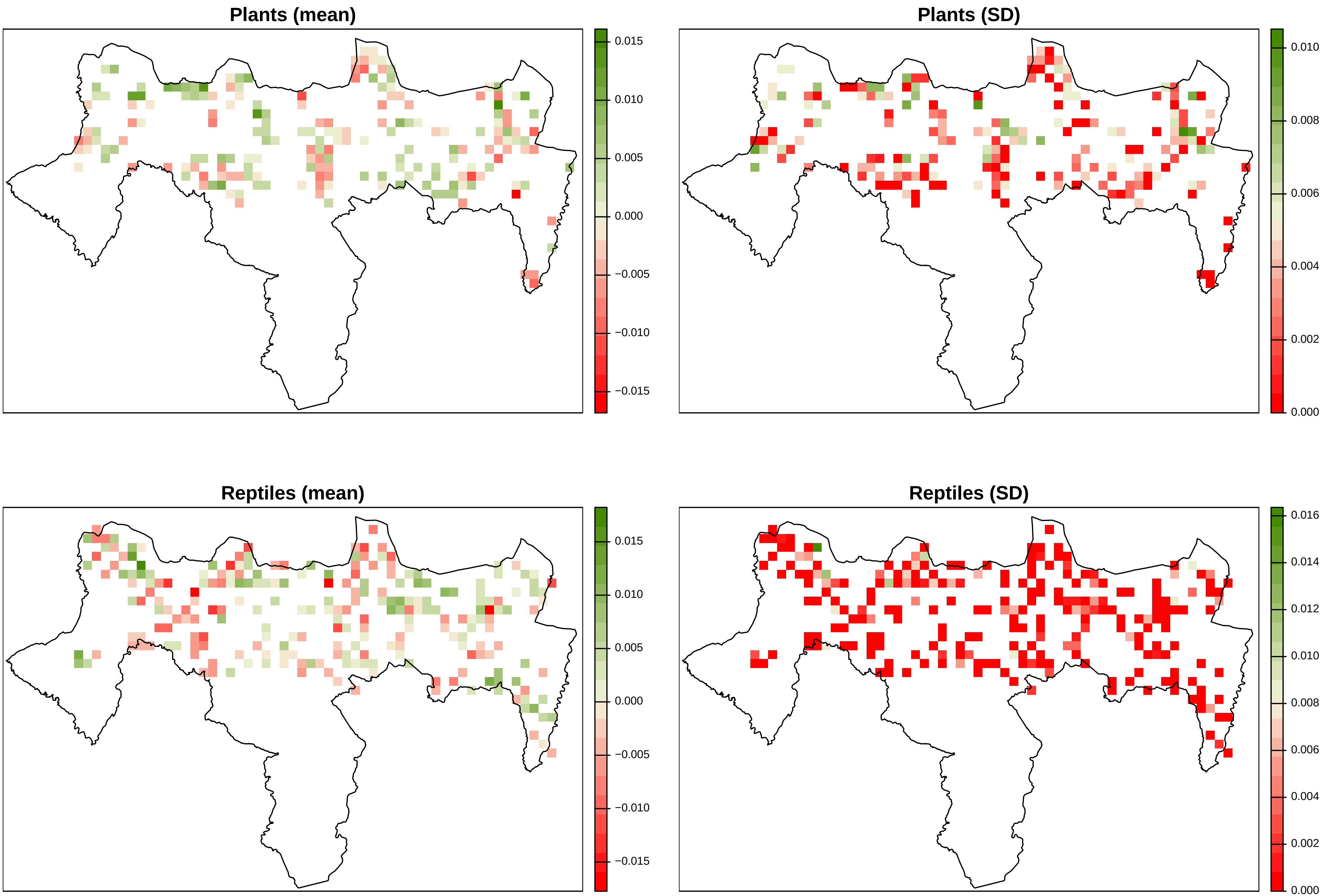
Plants (mean)
0.015
0.010
0.005
0.000
−0.005
−0.010
−0.015
Plants (SD)
0.010
0.008
0.006
0.004
0.002
0.000
Reptiles (mean)
0.015
0.010
0.005
0.000
−0.005
−0.010
−0.015
Reptiles (SD)
0.016
0.014
0.012
0.010
0.008
0.006
0.004
0.002
0.000
