# Supplementary material for: Time-Series Niche Modelling Reveals Declining Tendencies of Habitat Suitability and Ecological Functions in a Mountainous Protected Area
Source: Environ Manage. 2026 Feb 18;76(3):101. doi: 10.1007/s00267-026-02393-5 (PMC12916538; doi:10.1007/s00267-026-02393-5)
Supplement: Supplementary file 6 — ESM_5.B [file 267_2026_2393_MOESM6_ESM.pdf]

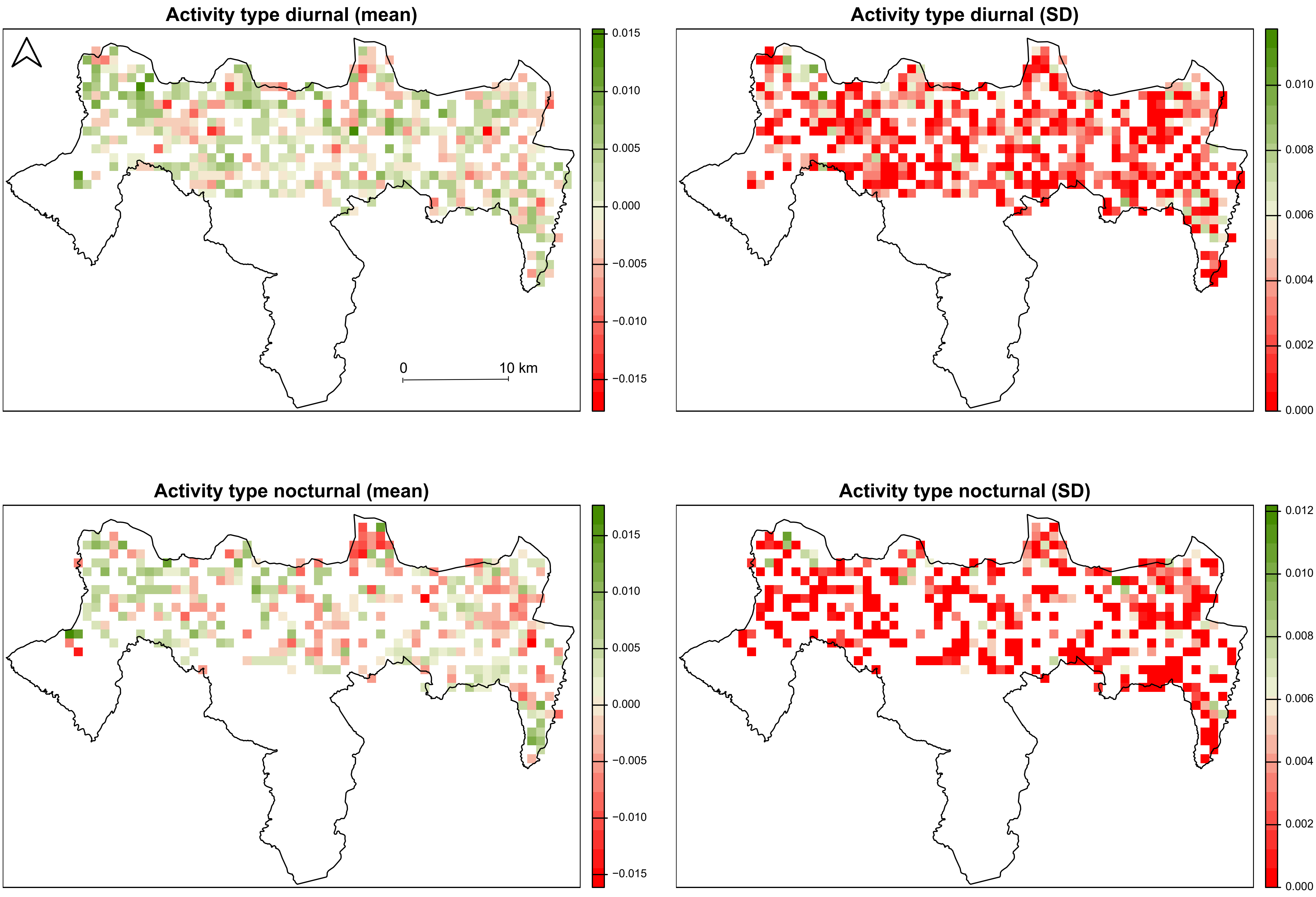

Activity type diurnal (mean)
0.015
0.010
0.005
0.000
−0.005
−0.010
−0.015
0
10 km
Activity type diurnal (SD)
0.010
0.008
0.006
0.004
0.002
0.000
Activity type nocturnal (mean)
0.015
0.010
0.005
0.000
−0.005
−0.010
−0.015
Activity type nocturnal (SD)
0.012
0.010
0.008
0.006
0.004
0.002
0.000

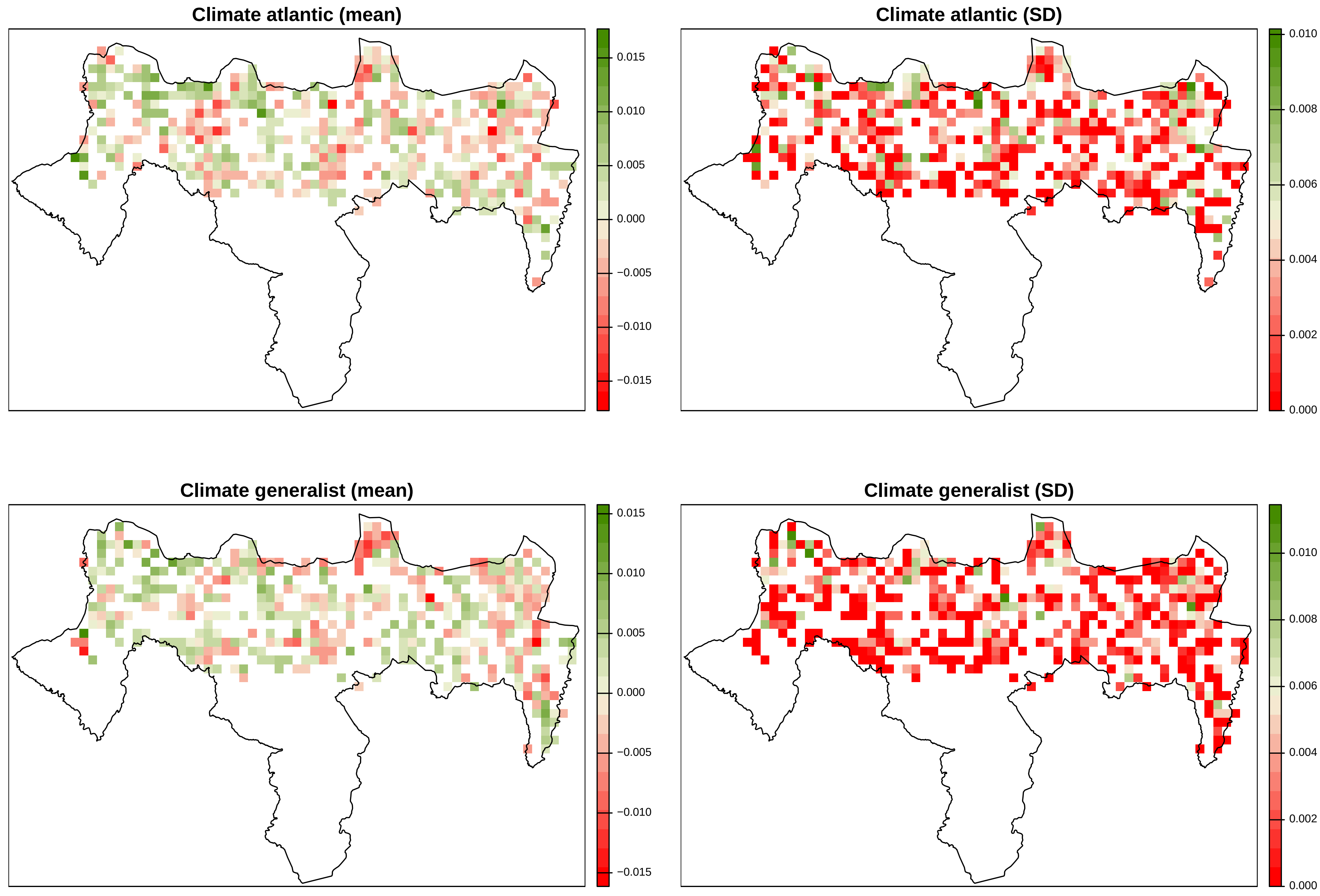
Climate atlantic (mean)
0.015
0.010
0.005
0.000
−0.005
−0.010
−0.015
Climate atlantic (SD)
0.010
0.008
0.006
0.004
0.002
0.000
Climate generalist (mean)
0.015
0.010
0.005
0.000
−0.005
−0.010
−0.015
Climate generalist (SD)
0.010
0.008
0.006
0.004
0.002
0.000

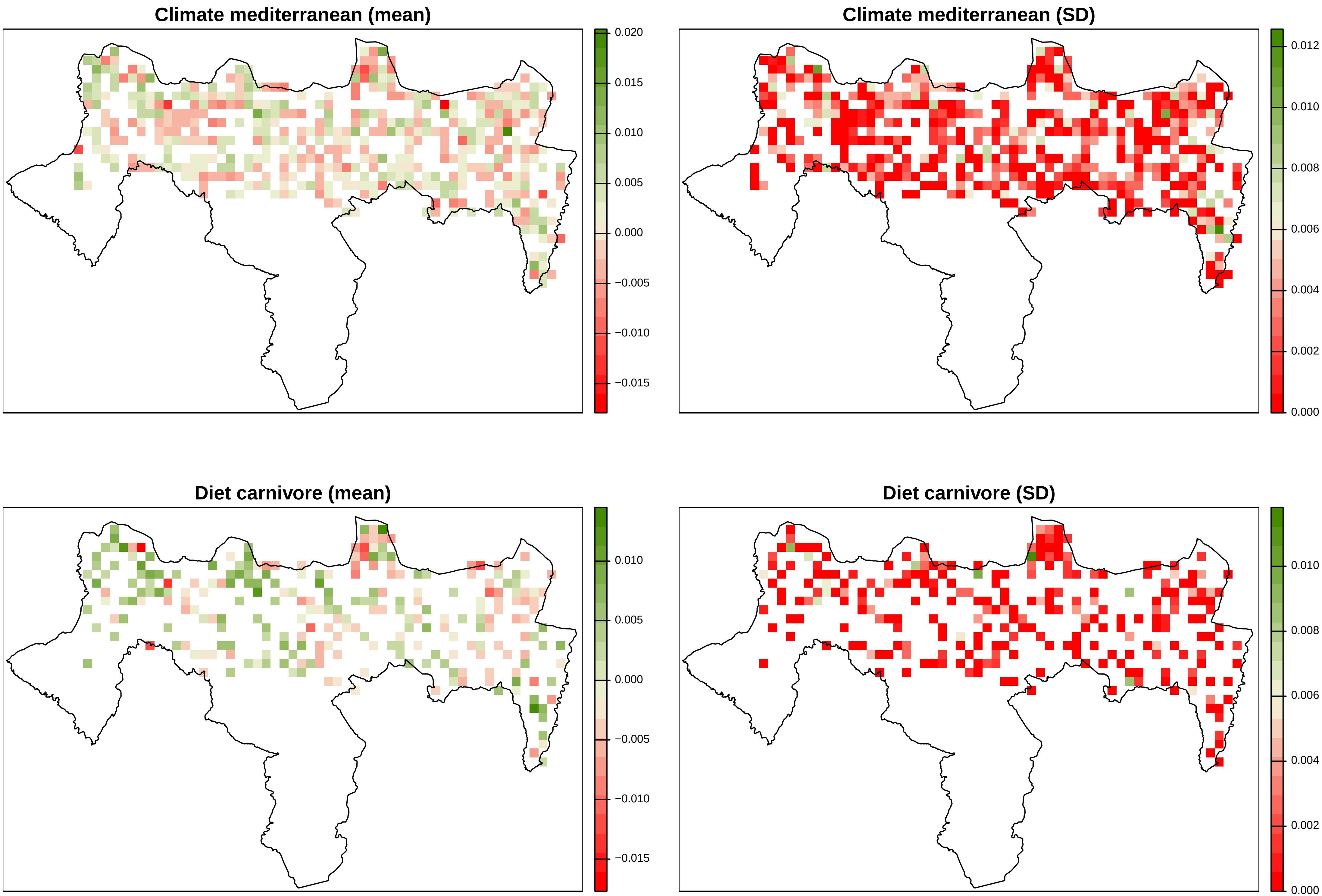
Climate mediterranean (mean)
0.020
0.015
0.010
0.005
0.000
−0.005
−0.010
−0.015
Climate mediterranean (SD)
0.012
0.010
0.008
0.006
0.004
0.002
0.000
Diet carnivore (mean)
0.010
0.005
0.000
−0.005
−0.010
−0.015
Diet carnivore (SD)
0.010
0.008
0.006
0.004
0.002
0.000

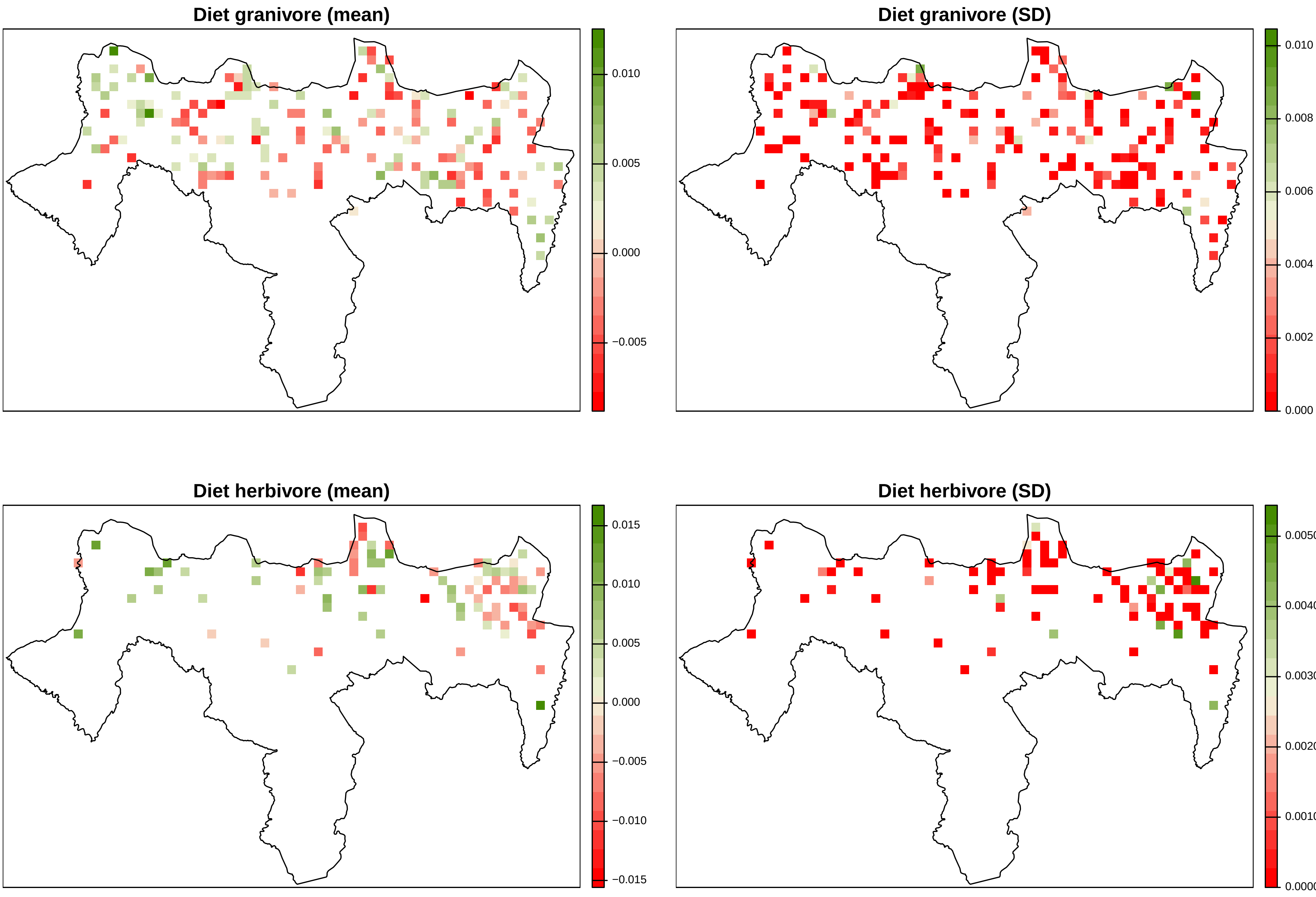
Diet granivore (mean)
0.010
0.005
0.000
−0.005
Diet granivore (SD)
0.010
0.008
0.006
0.004
0.002
0.000
Diet herbivore (mean)
0.015
0.010
0.005
0.000
−0.005
−0.010
−0.015
Diet herbivore (SD)
0.0050
0.0040
0.0030
0.0020
0.0010
0.0000

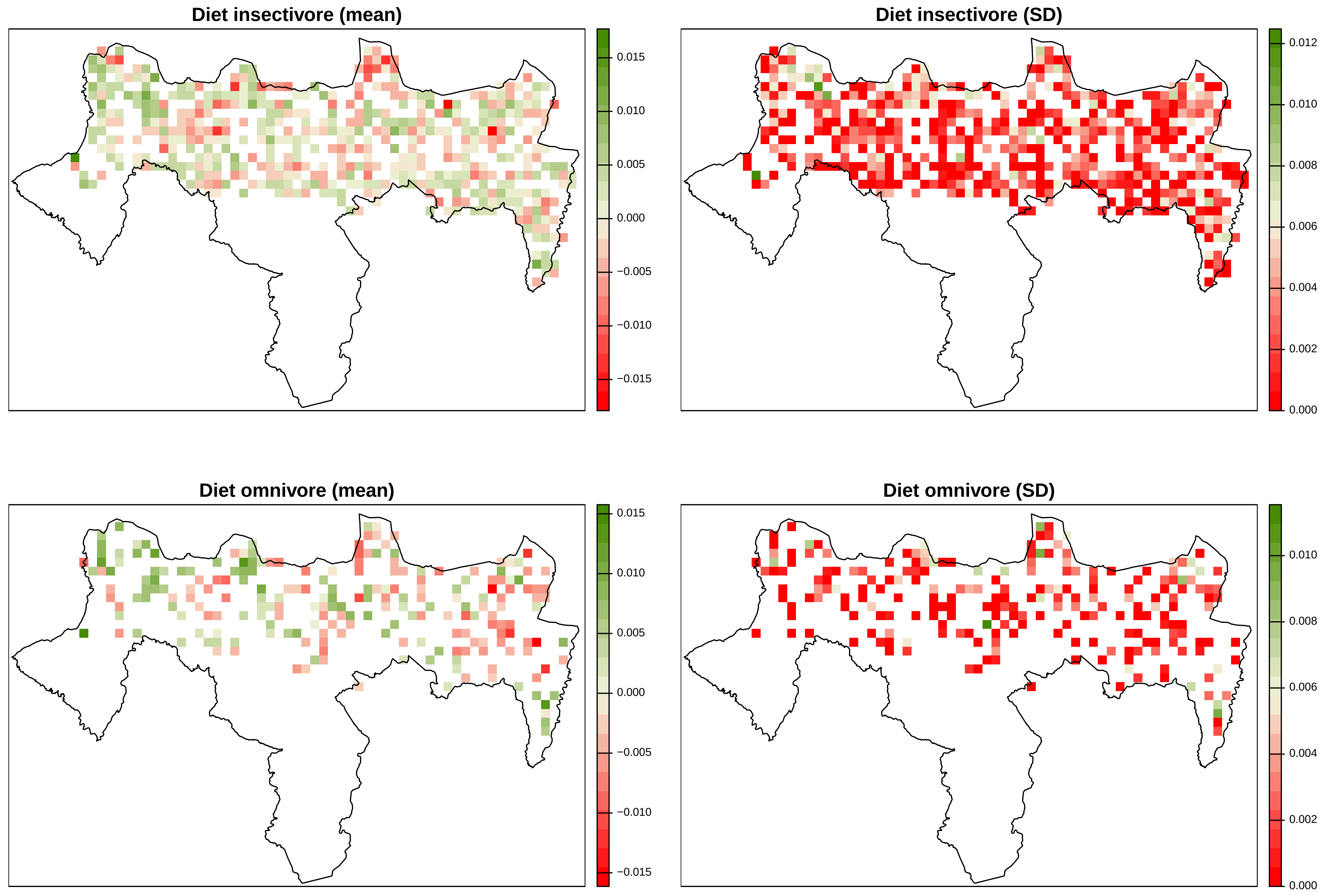
Diet insectivore (mean)
0.015
0.010
0.005
0.000
−0.005
−0.010
−0.015
Diet insectivore (SD)
0.012
0.010
0.008
0.006
0.004
0.002
0.000
Diet omnivore (mean)
0.015
0.010
0.005
0.000
−0.005
−0.010
−0.015
Diet omnivore (SD)
0.010
0.008
0.006
0.004
0.002
0.000

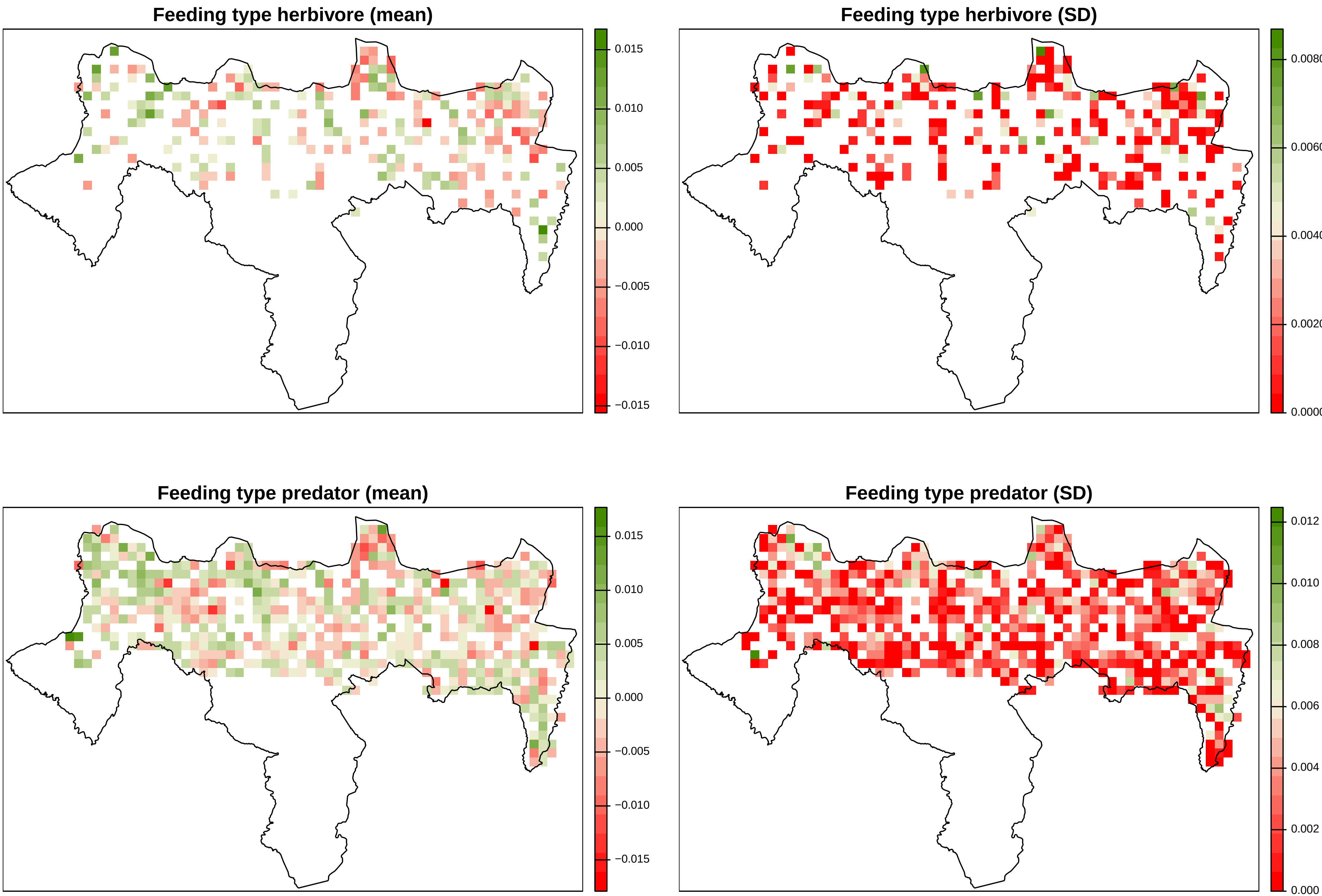
Feeding type herbivore (mean)
0.015
0.010
0.005
0.000
−0.005
−0.010
−0.015
Feeding type herbivore (SD)
Feeding type predator (mean)
0.015
0.010
0.005
0.000
−0.005
−0.010
−0.015
Feeding type predator (SD)
0.012
0.010
0.008
0.006
0.004
0.002
0.000

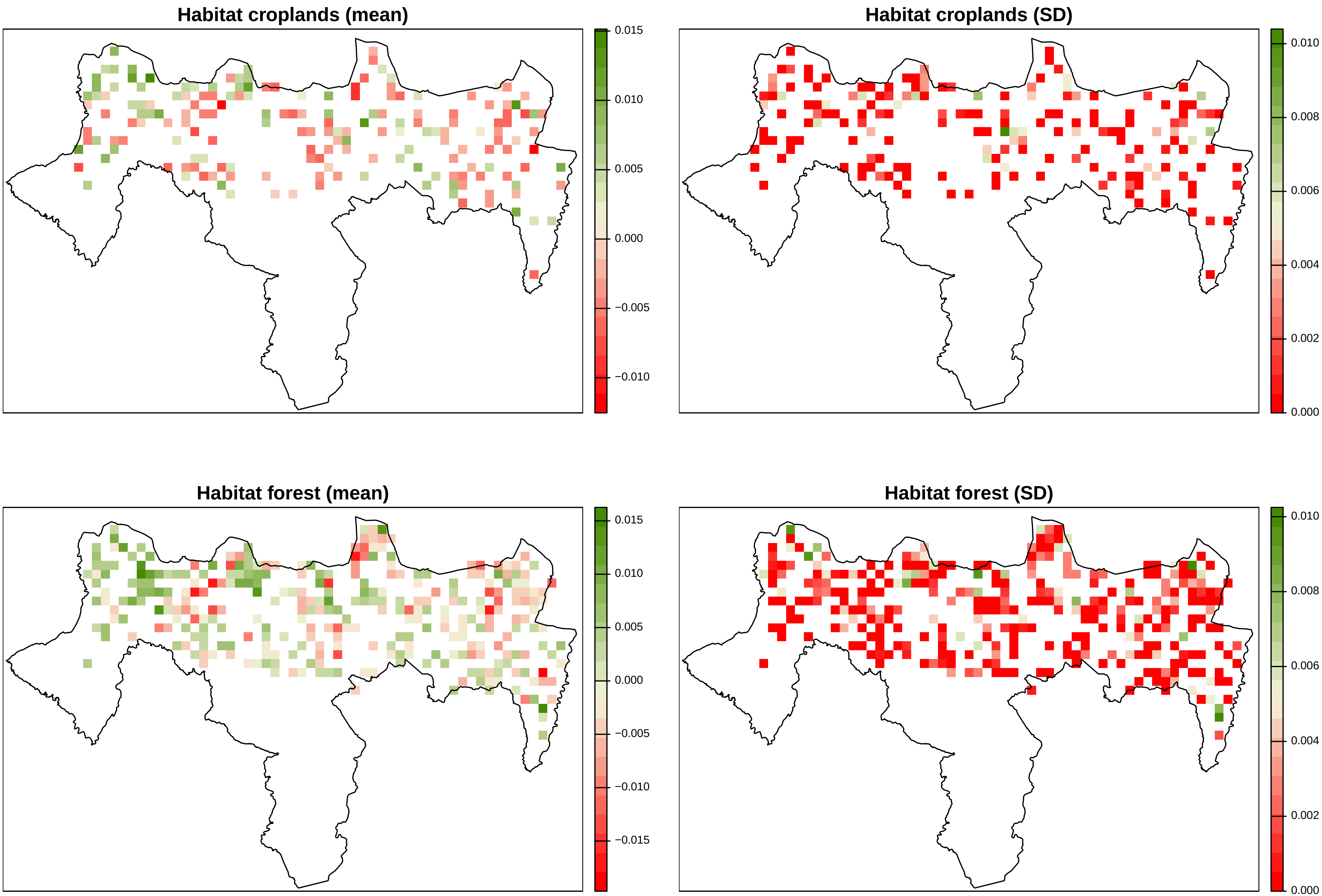
Habitat croplands (mean)
0.015
0.010
0.005
0.000
−0.005
−0.010
Habitat croplands (SD)
0.010
0.008
0.006
0.004
0.002
0.000
Habitat forest (mean)
0.015
0.010
0.005
0.000
−0.005
−0.010
−0.015
Habitat forest (SD)
0.010
0.008
0.006
0.004
0.002
0.000

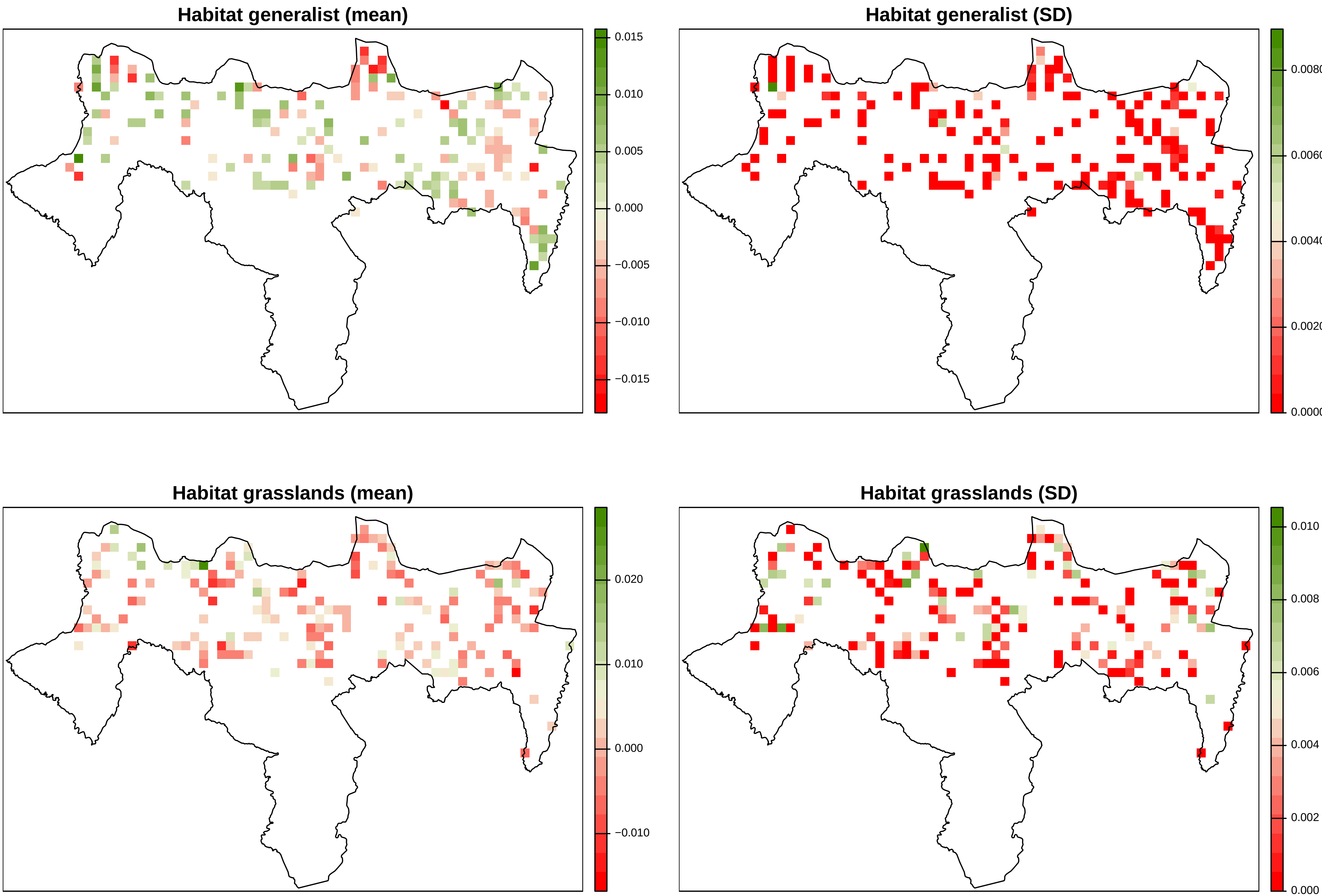
Habitat generalist (mean)
0.015
0.010
0.005
0.000
−0.005
−0.010
−0.015
Habitat generalist (SD)
0.0080
0.0060
0.0040
0.0020
0.0000
Habitat grasslands (mean)
0.020
0.010
0.000
−0.010
Habitat grasslands (SD)
0.010
0.008
0.006
0.004
0.002
0.000

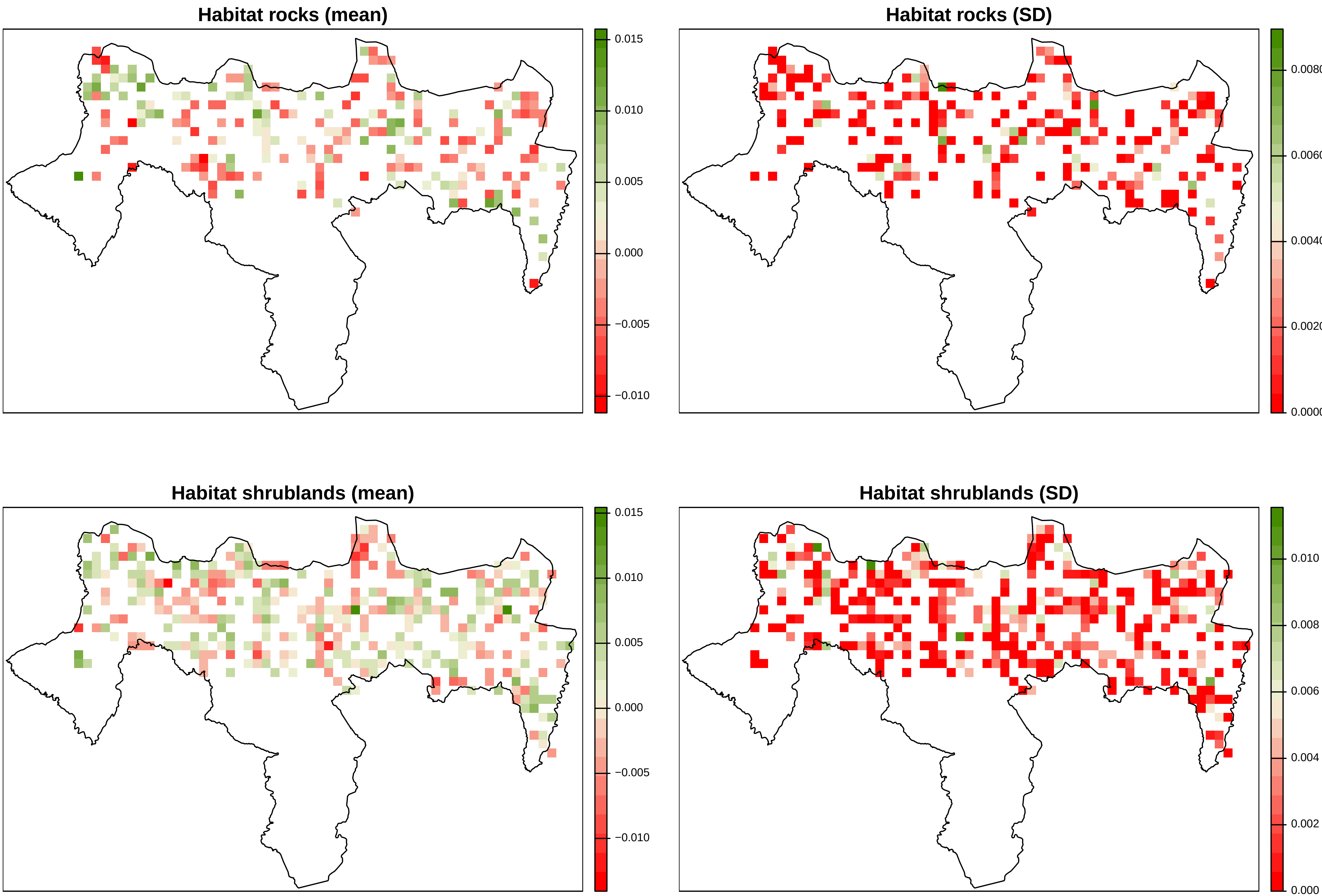
Habitat rocks (mean)
0.015
0.010
0.005
0.000
−0.005
−0.010
Habitat rocks (SD)
0.008
0.006
0.004
0.002
0.000
Habitat shrublands (mean)
0.015
0.010
0.005
0.000
−0.005
−0.010
Habitat shrublands (SD)
0.010
0.008
0.006
0.004
0.002
0.000

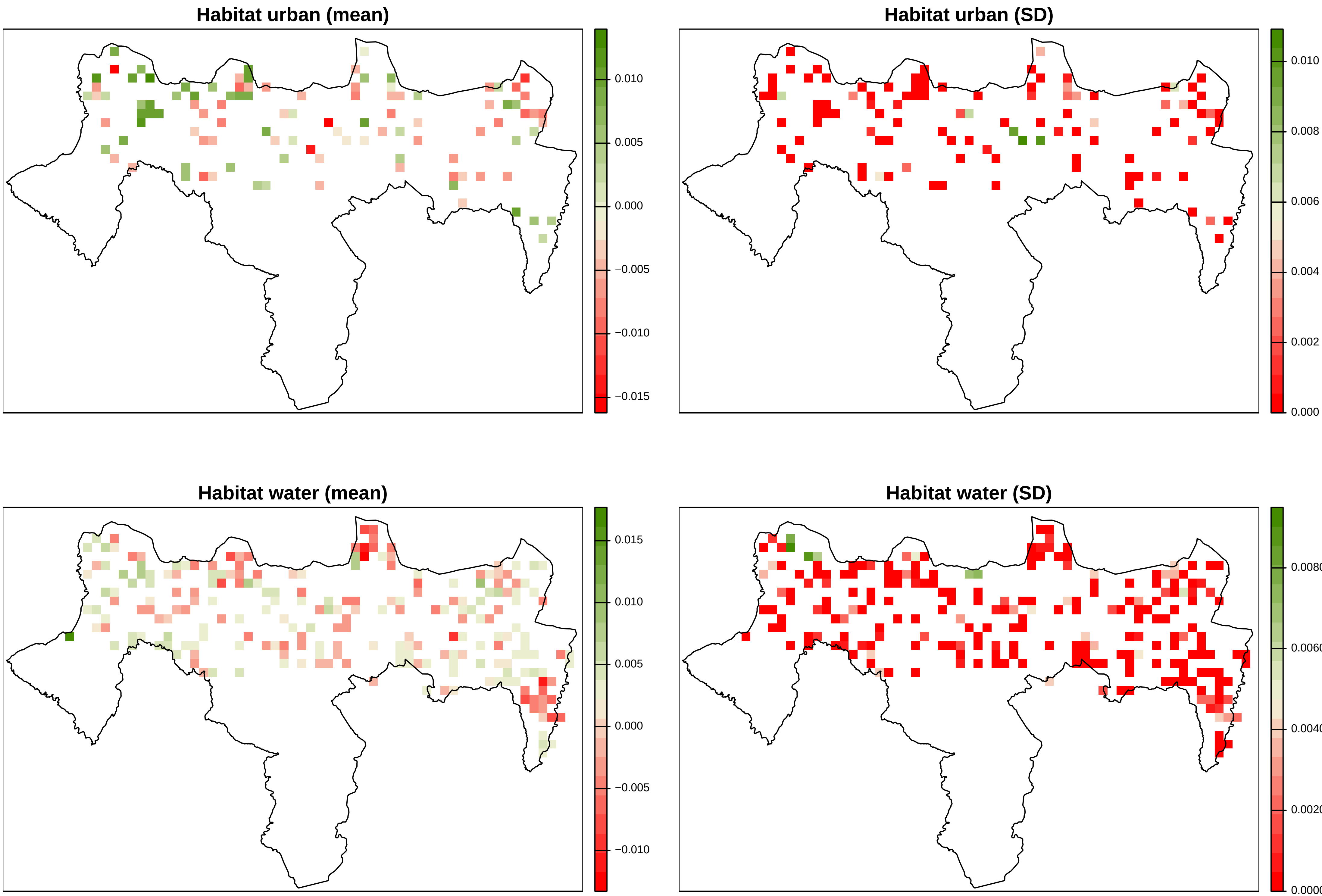
Habitat urban (mean)
0.010
0.005
0.000
−0.005
−0.010
−0.015
Habitat urban (SD)
0.010
0.008
0.006
0.004
0.002
0.000
Habitat water (mean)
0.015
0.010
0.005
0.000
−0.005
−0.010
Habitat water (SD)
0.008
0.006
0.004
0.002
0.000

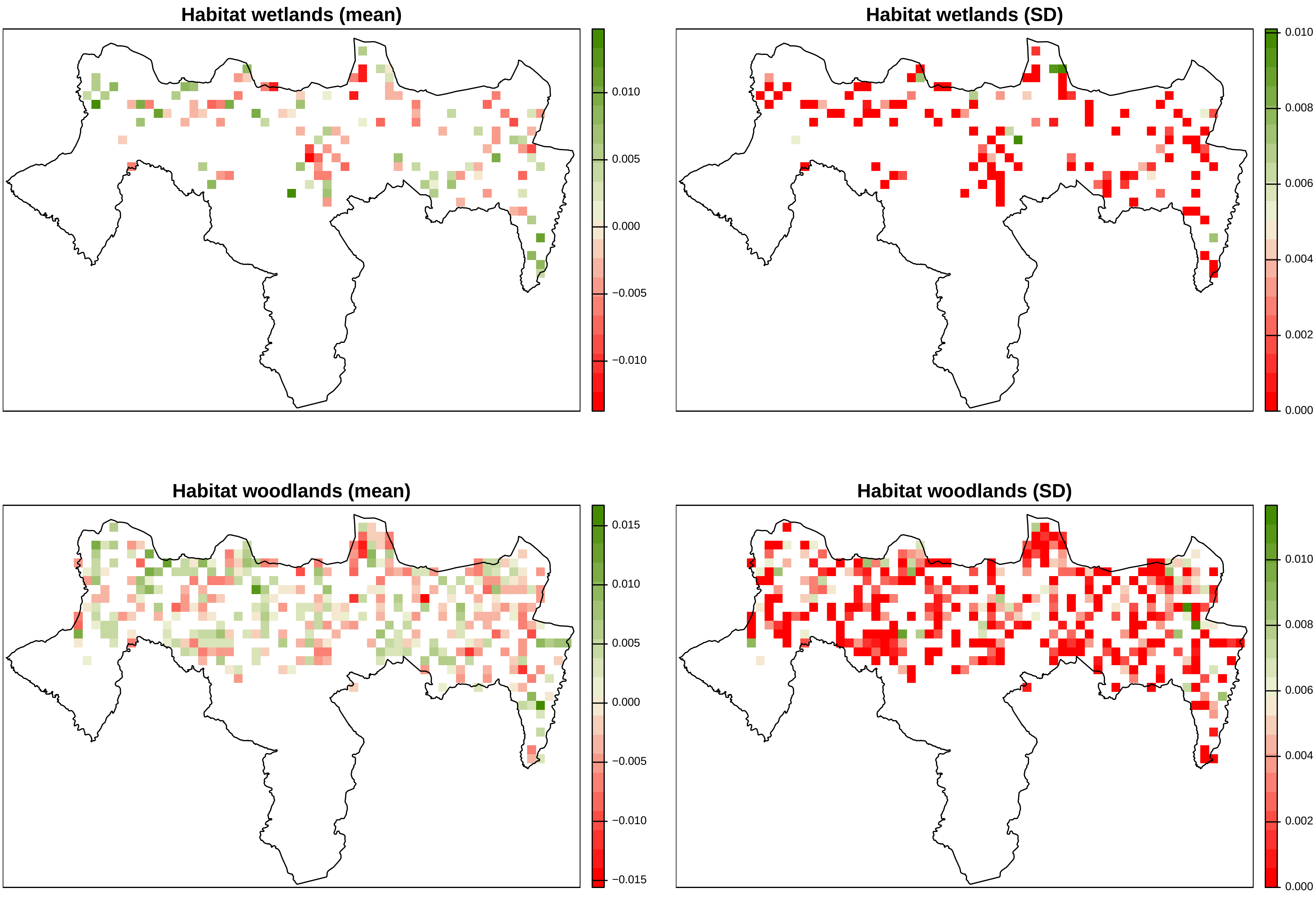
Habitat wetlands (mean)
0.010
0.005
0.000
−0.005
−0.010
Habitat wetlands (SD)
0.010
0.008
0.006
0.004
0.002
0.000
Habitat woodlands (mean)
0.015
0.010
0.005
0.000
−0.005
−0.010
−0.015
Habitat woodlands (SD)
0.010
0.008
0.006
0.004
0.002
0.000

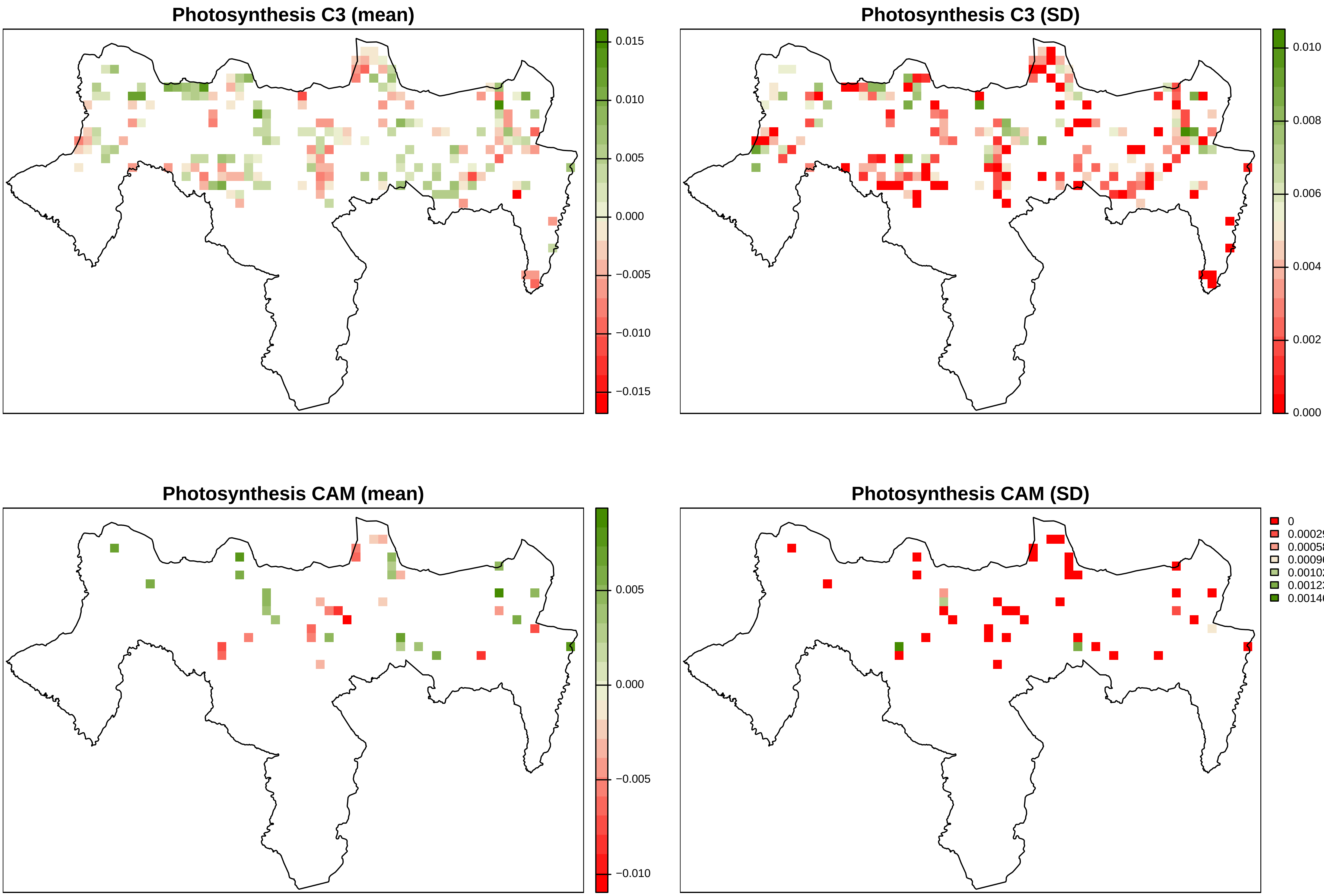
Photosynthesis C3 (mean)
0.015
0.010
0.005
0.000
−0.005
−0.010
−0.015
Photosynthesis C3 (SD)
0.010
0.008
0.006
0.004
0.002
0.000
Photosynthesis CAM (mean)
0.005
0.000
−0.005
−0.010
Photosynthesis CAM (SD)
0
0.0002
0.0005
0.0009
0.0010
0.0012
0.0014

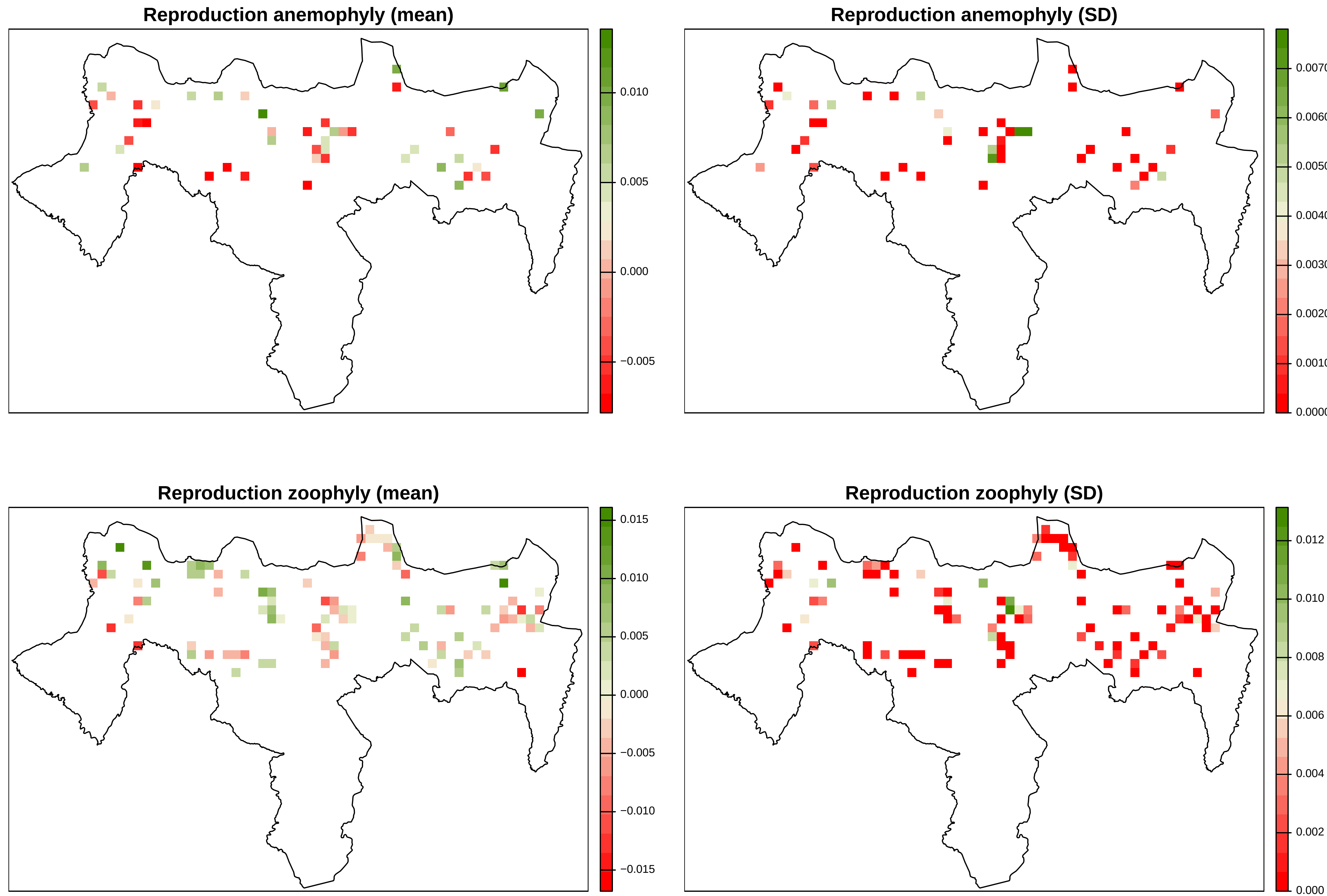
Reproduction anemophyly (mean)
0.010
0.005
0.000
−0.005
Reproduction anemophyly (SD)
0.007
0.006
0.005
0.004
0.003
0.002
0.001
0.000
Reproduction zoophyly (mean)
0.015
0.010
0.005
0.000
−0.005
−0.010
−0.015
Reproduction zoophyly (SD)
0.012
0.010
0.008
0.006
0.004
0.002
0.000
