# Supplementary material for: Time-Series Niche Modelling Reveals Declining Tendencies of Habitat Suitability and Ecological Functions in a Mountainous Protected Area
Source: Environ Manage. 2026 Feb 18;76(3):101. doi: 10.1007/s00267-026-02393-5 (PMC12916538; doi:10.1007/s00267-026-02393-5)
Supplement: Supplementary file 7 — ESM_5.C [file 267_2026_2393_MOESM7_ESM.pdf]

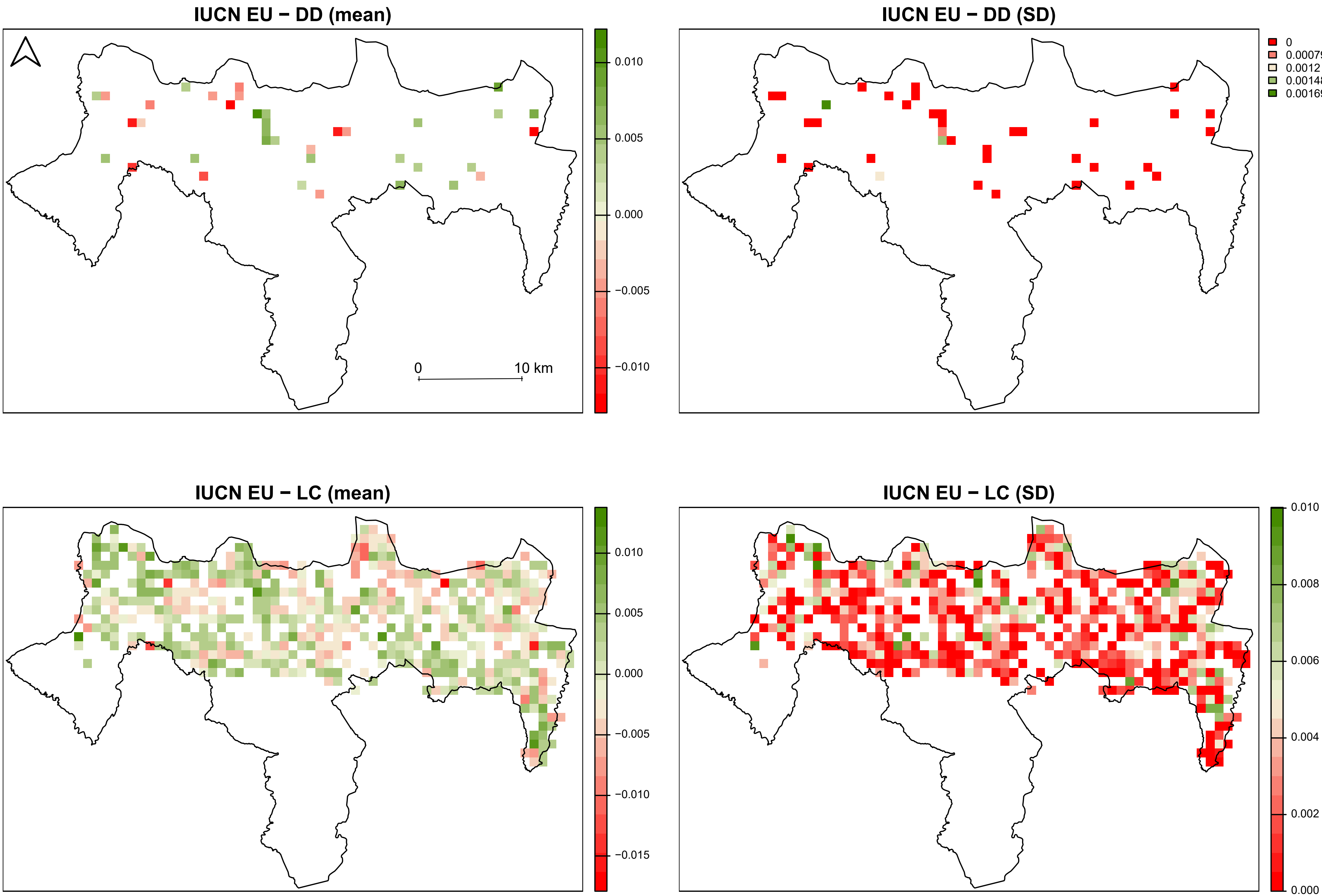
IUCN EU − DD (mean)
0.010
0.005
0.000
−0.005
−0.010
0
10 km
IUCN EU − DD (SD)
0
0.0007
0.0012
0.0014
0.0016
IUCN EU − LC (mean)
0.010
0.005
0.000
−0.005
−0.010
−0.015
IUCN EU − LC (SD)
0.010
0.008
0.006
0.004
0.002
0.000

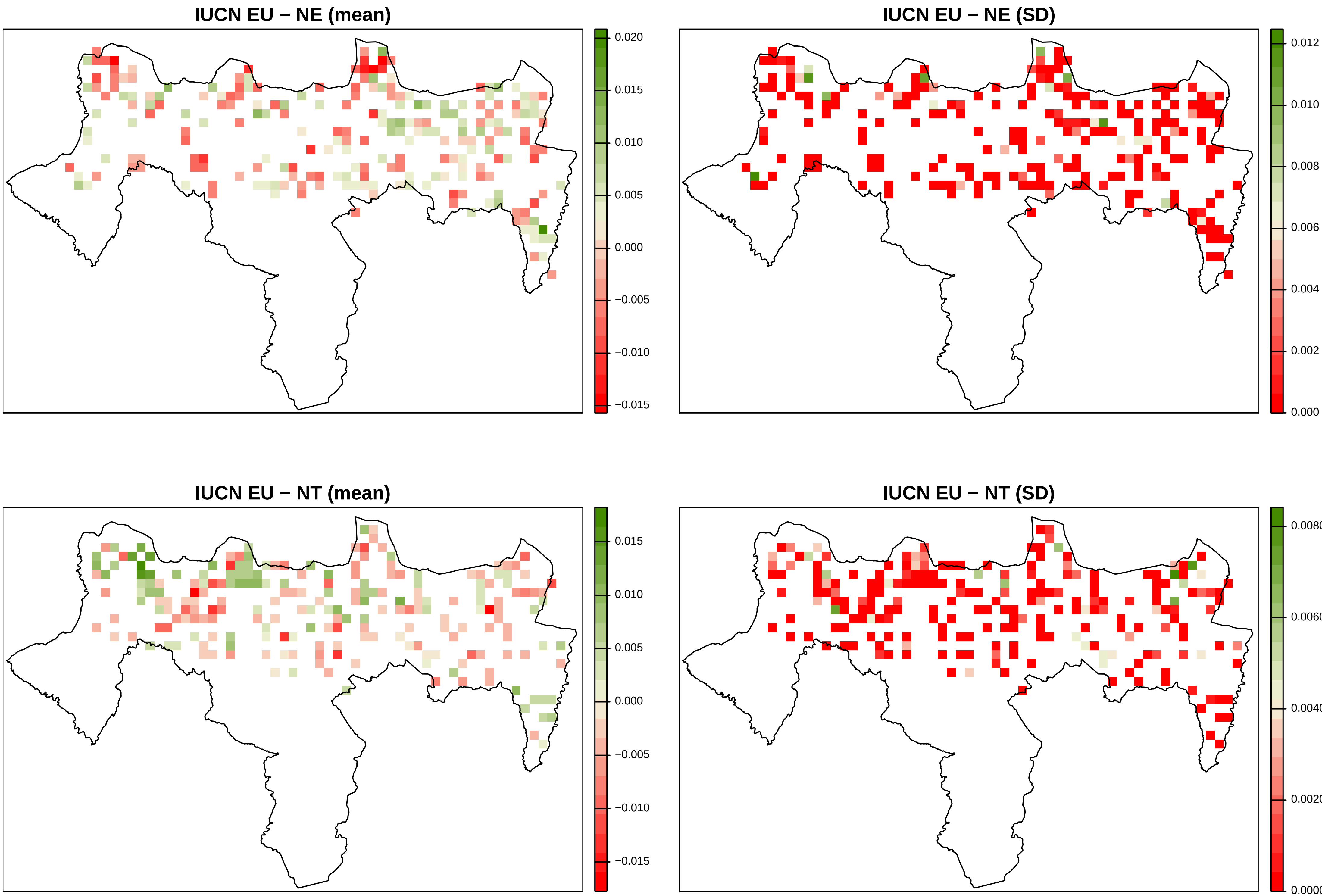
IUCN EU − NE (mean)
0.020
0.015
0.010
0.005
0.000
−0.005
−0.010
−0.015
IUCN EU − NE (SD)
0.012
0.010
0.008
0.006
0.004
0.002
0.000
IUCN EU − NT (mean)
0.015
0.010
0.005
0.000
−0.005
−0.010
−0.015
IUCN EU − NT (SD)
0.008
0.006
0.004
0.002
0.000

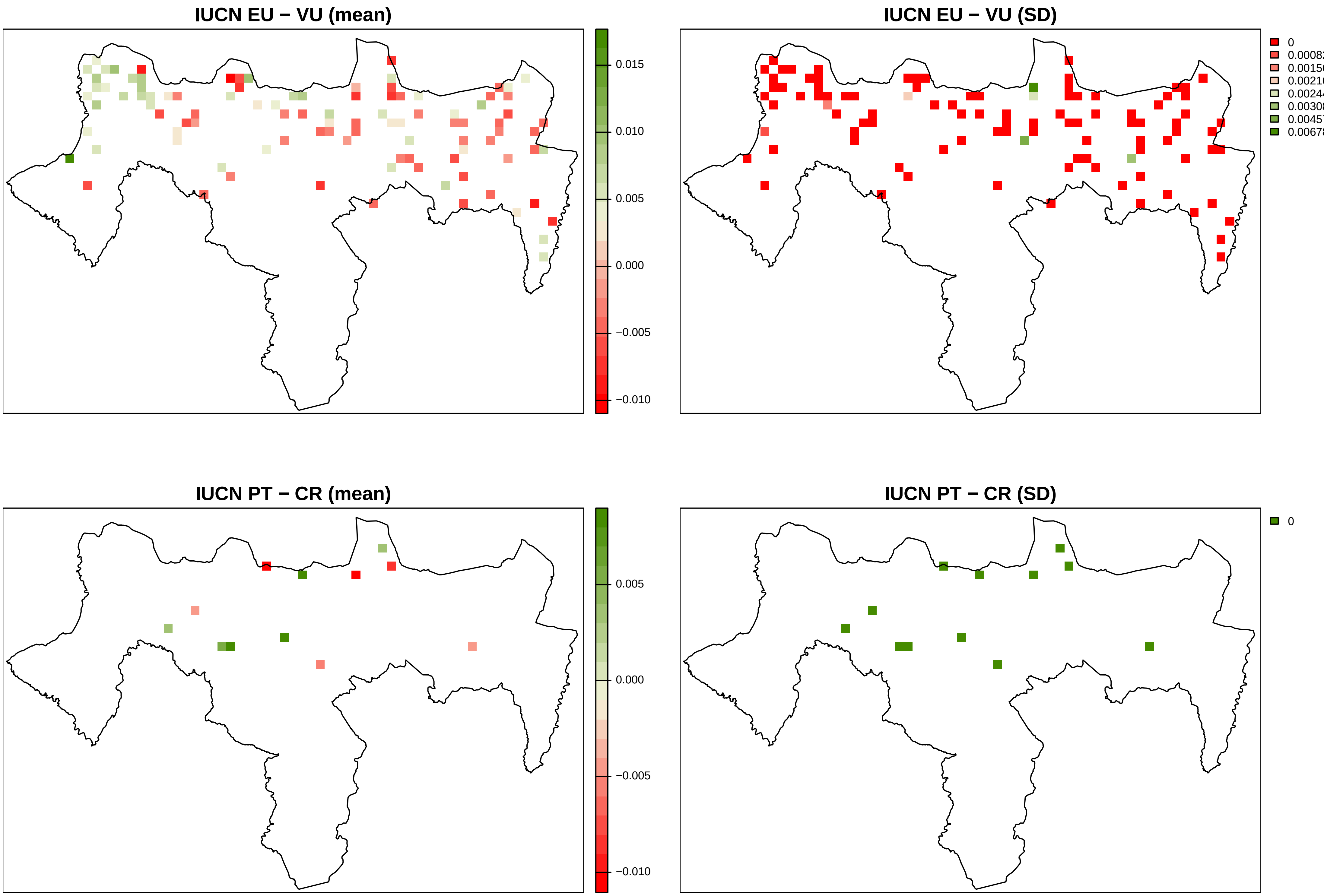

IUCN EU − VU (mean)
0.015
0.010
0.005
0.000
−0.005
−0.010
IUCN EU − VU (SD)
0
0.0008
0.0015
0.0021
0.0024
0.0030
0.0045
0.0067
IUCN PT − CR (mean)
0.005
0.000
−0.005
−0.010
IUCN PT − CR (SD)
0

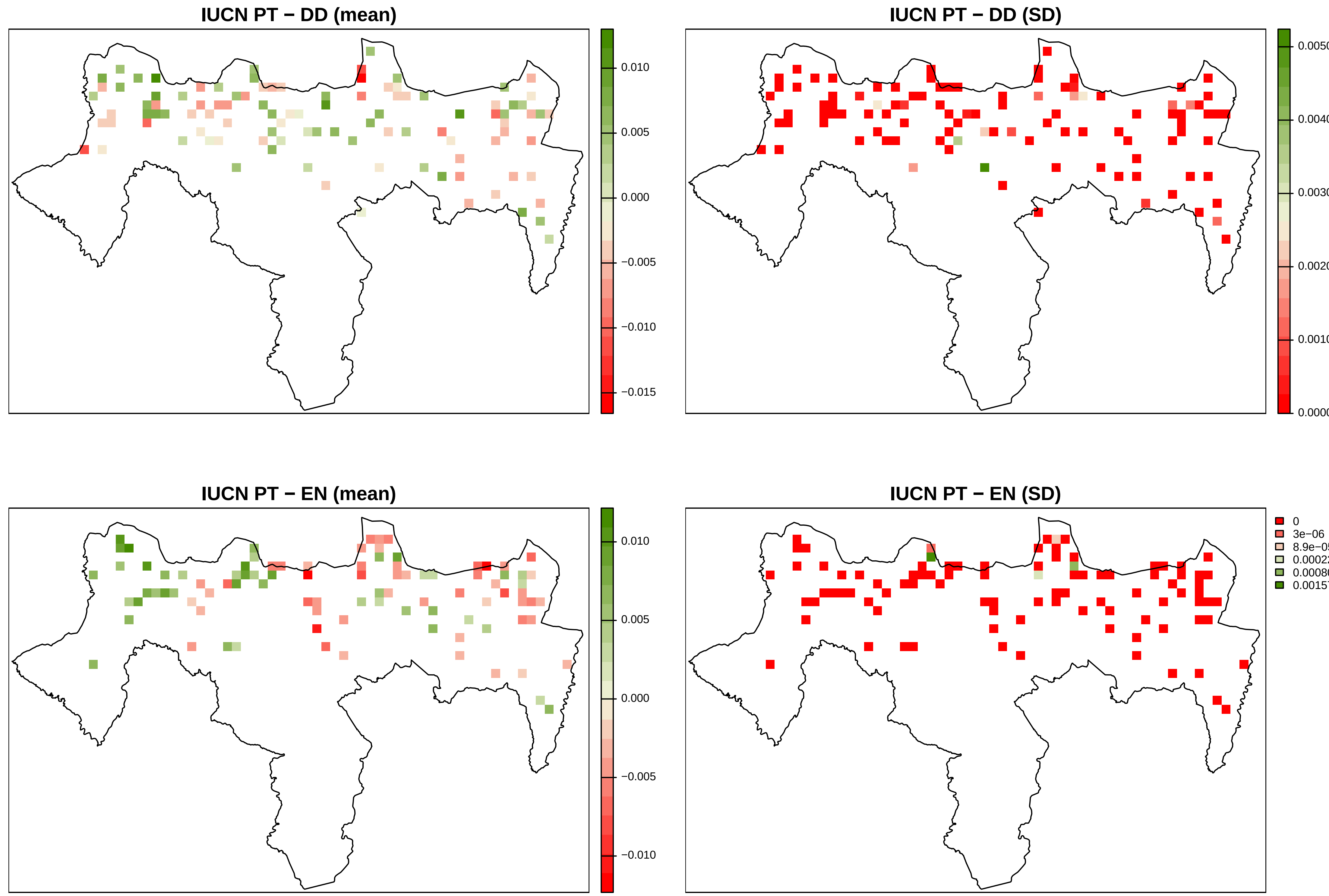
IUCN PT − DD (mean)
0.010
0.005
0.000
−0.005
−0.010
−0.015
IUCN PT − DD (SD)
IUCN PT − EN (mean)
0.010
0.005
0.000
−0.005
−0.010
IUCN PT − EN (SD)
0
3e−06

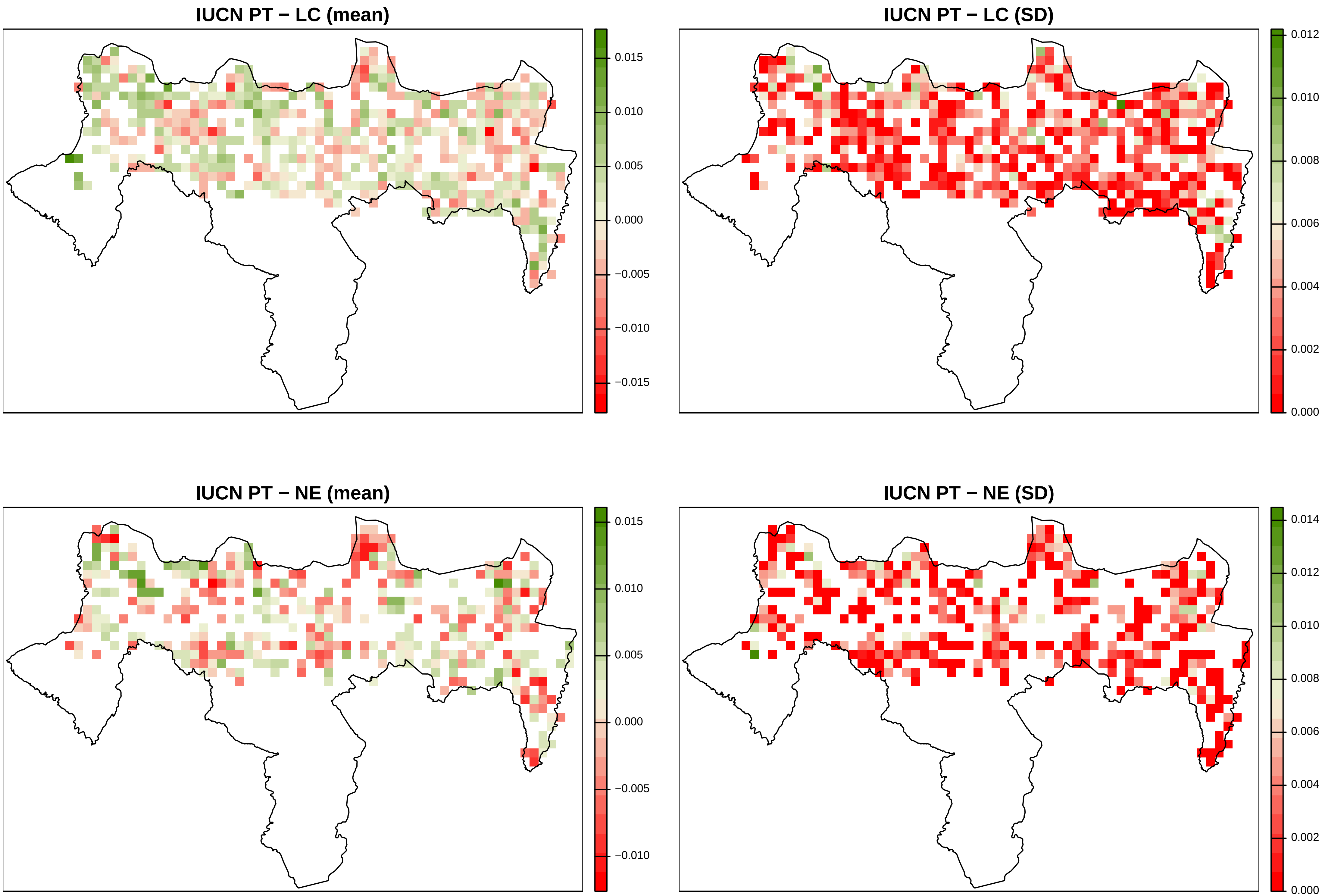
IUCN PT − LC (mean)
0.015
0.010
0.005
0.000
−0.005
−0.010
−0.015
IUCN PT − LC (SD)
0.012
0.010
0.008
0.006
0.004
0.002
0.000
IUCN PT − NE (mean)
0.015
0.010
0.005
0.000
−0.005
−0.010
IUCN PT − NE (SD)
0.014
0.012
0.010
0.008
0.006
0.004
0.002
0.000

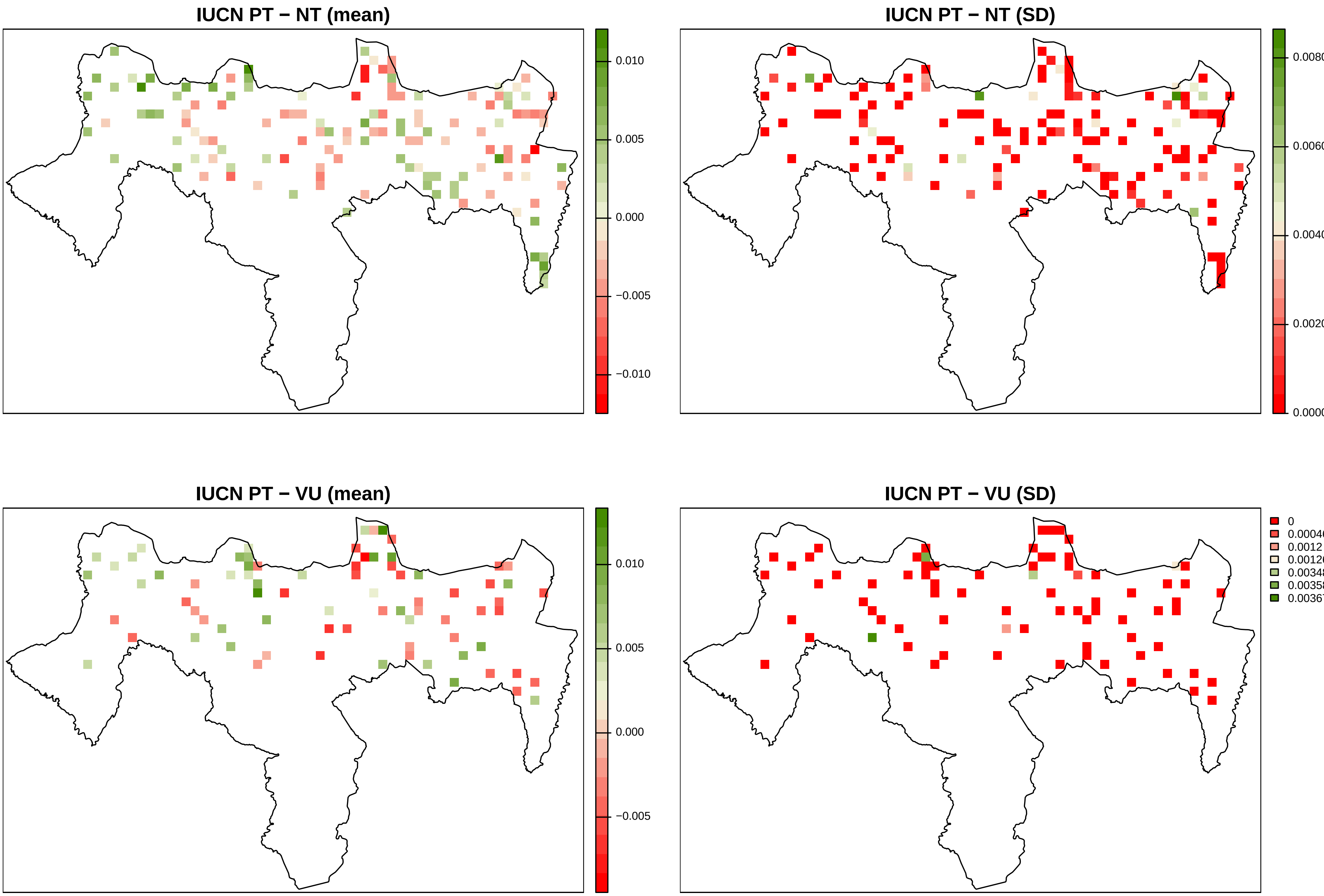

IUCN PT − NT (mean)
0.010
0.005
0.000
−0.005
−0.010
IUCN PT − NT (SD)
IUCN PT − VU (mean)
0.010
0.005
0.000
−0.005
IUCN PT − VU (SD)
0
