# Supplementary figures and images for: Time-Series Niche Modelling Reveals Declining Tendencies of Habitat Suitability and Ecological Functions in a Mountainous Protected Area
Source: Environ Manage. 2026 Feb 18;76(3):101. doi: 10.1007/s00267-026-02393-5 (PMC12916538; doi:10.1007/s00267-026-02393-5)

### Climate: Vascular Plants

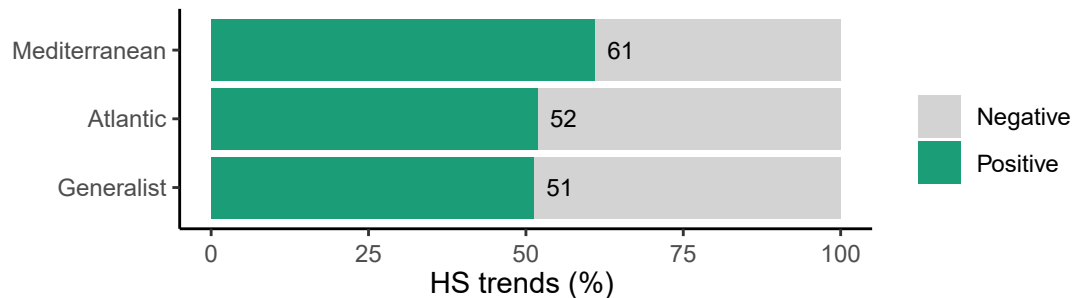

### Climate: Amphibians

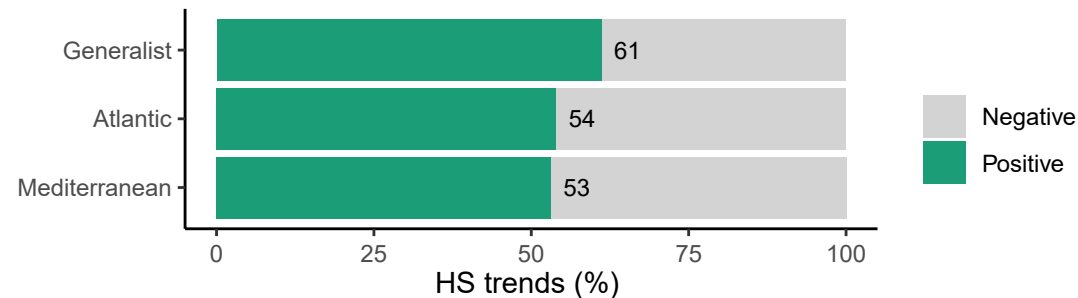

### Climate: Reptiles

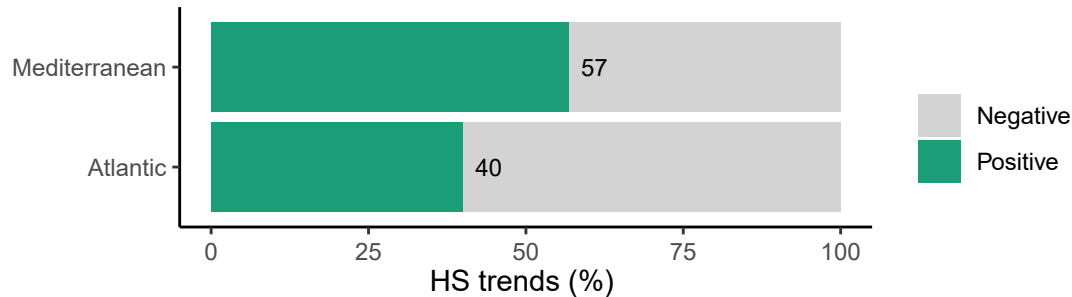

### Climate: Mammals

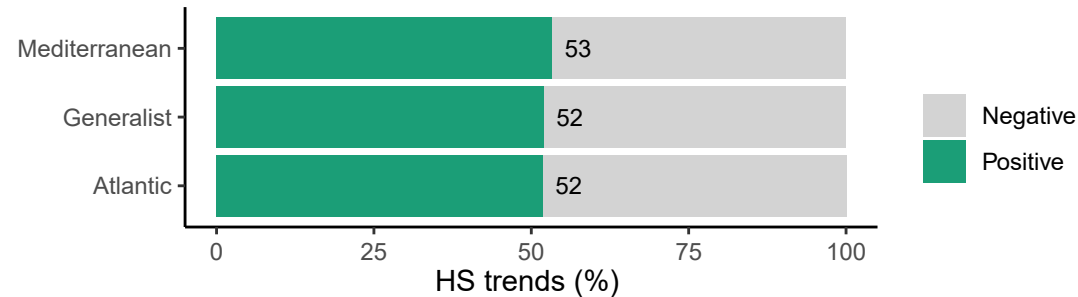

### Climate: Birds

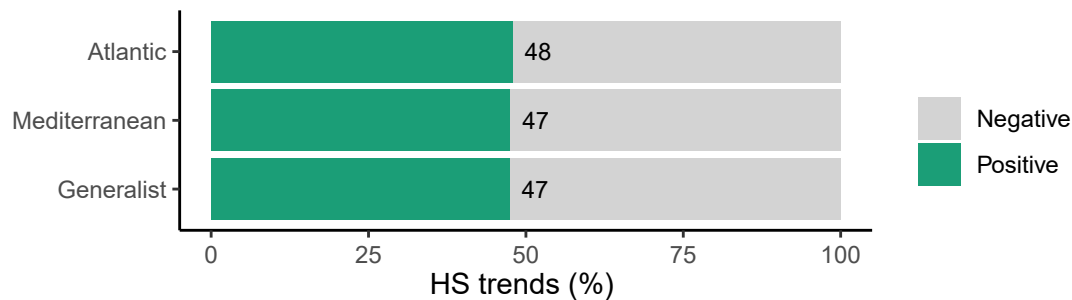

Supplement: Supplementary file 8 — ESM_6.A [file 267_2026_2393_MOESM8_ESM.pdf]

### Diet: Reptiles

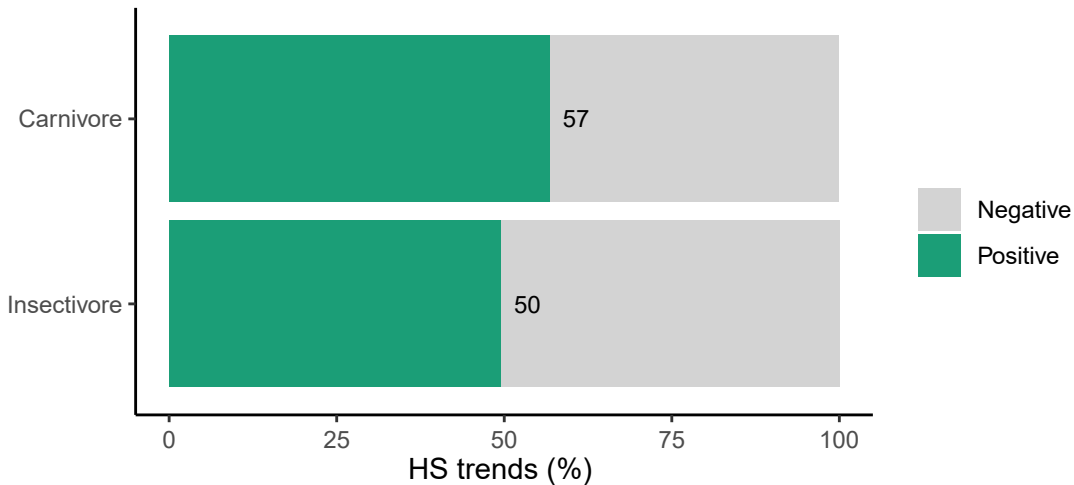

### Diet: Mammals

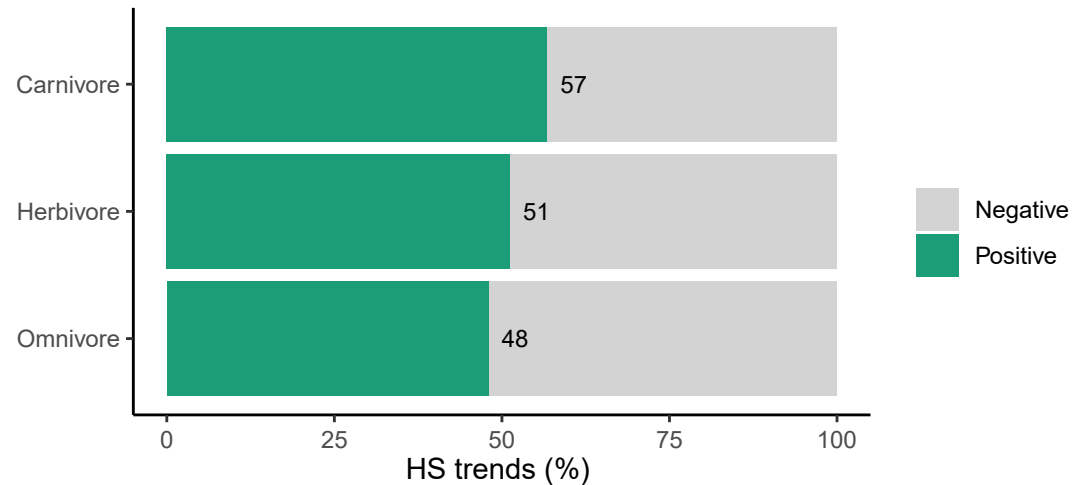

### Diet: Birds

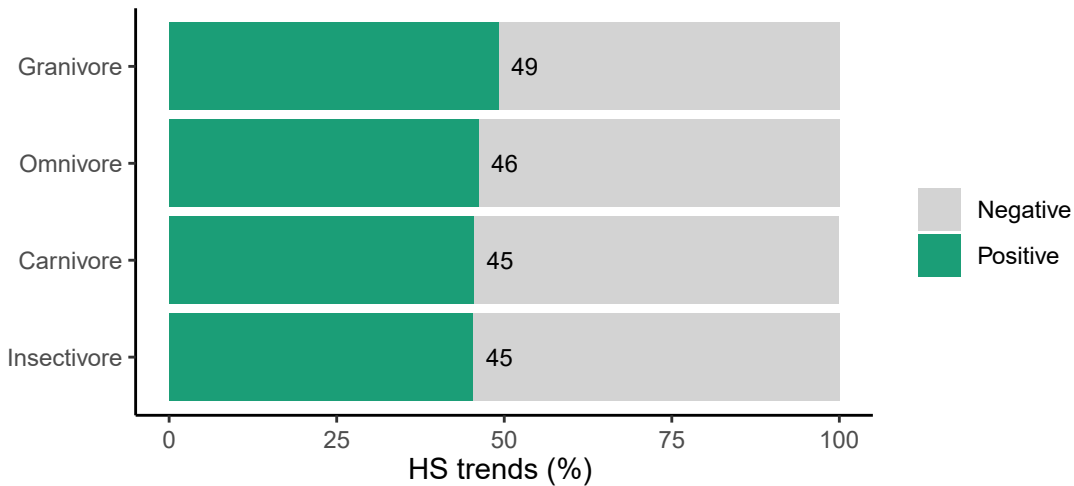

Supplement: Supplementary file 9 — ESM_6.B [file 267_2026_2393_MOESM9_ESM.pdf]

### Habitat: Vascular Plants

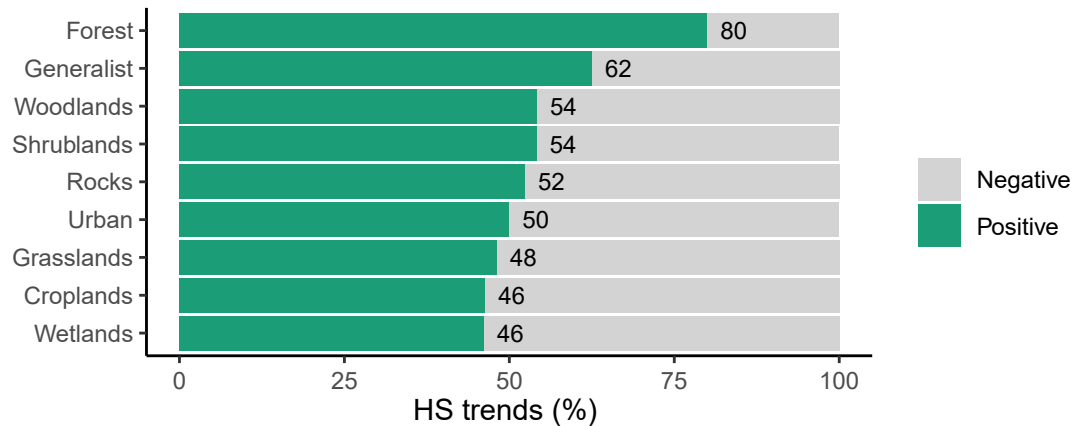

### Habitat: Amphibians

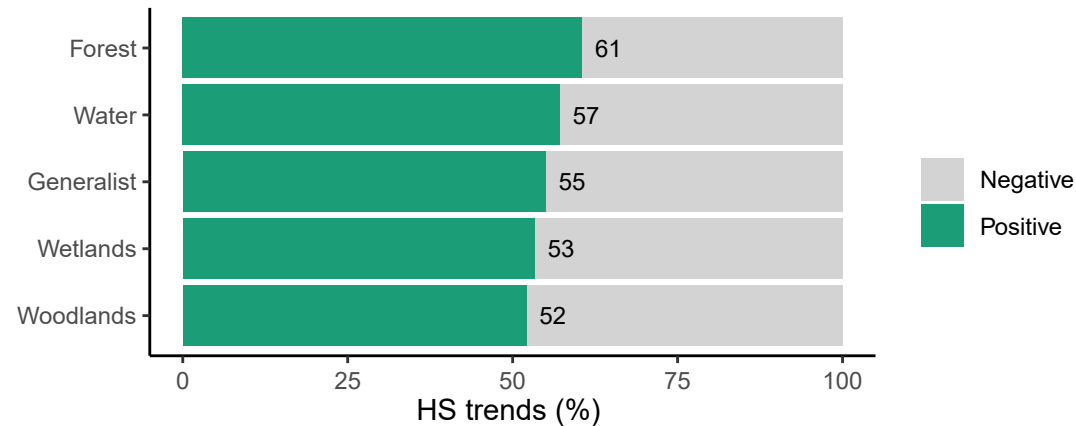

### Habitat: Reptiles

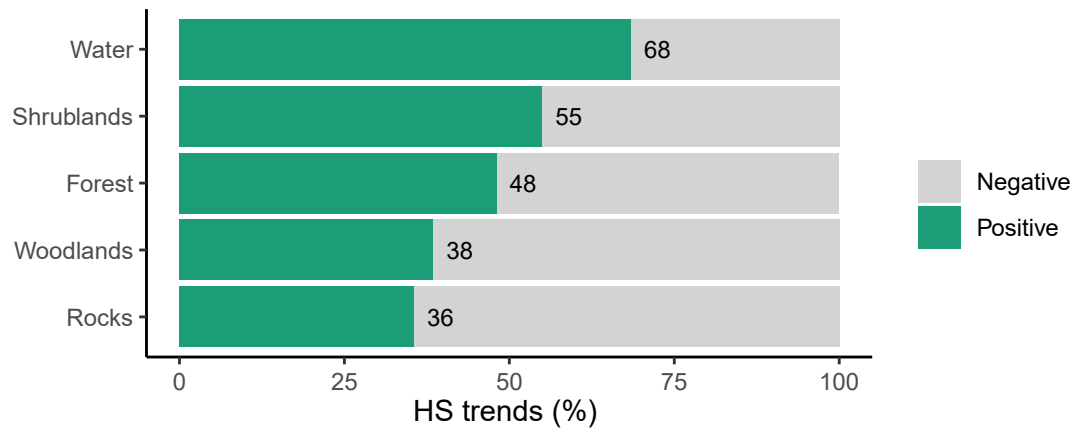

### Habitat: Mammals

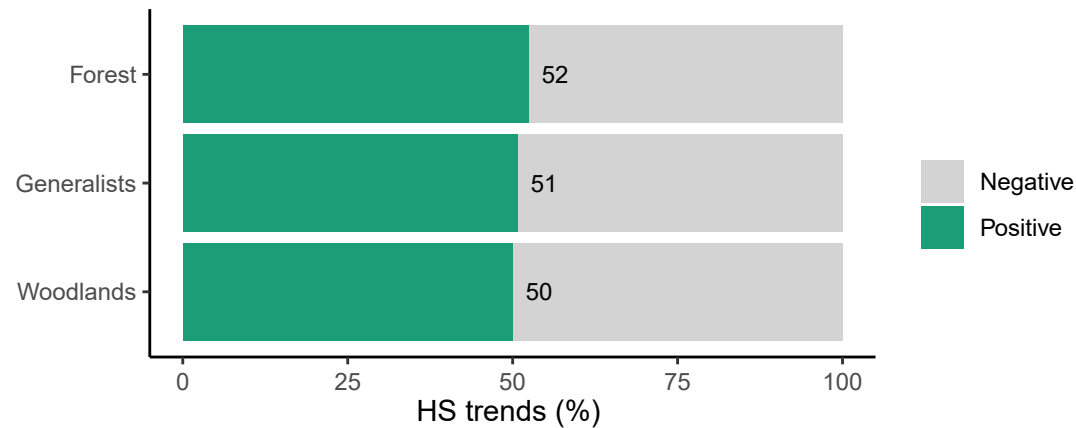

### Habitat: Birds

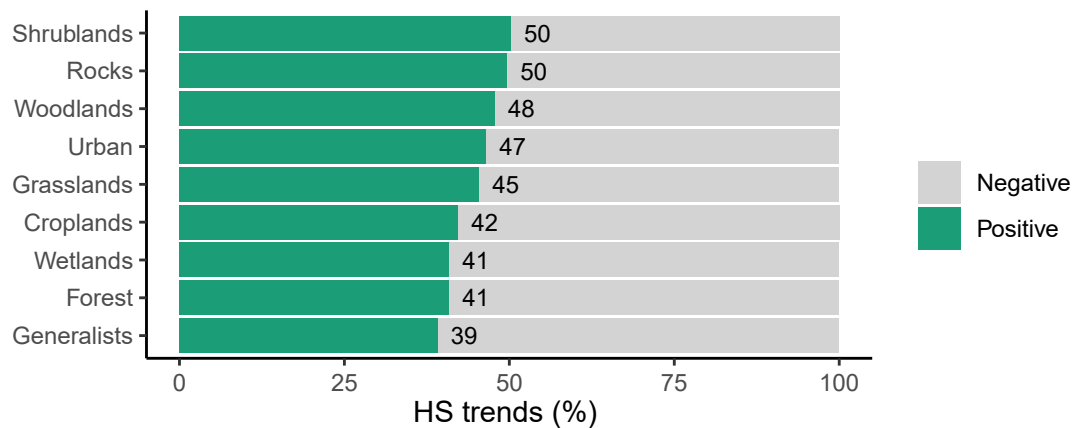

Supplement: Supplementary file 10 — ESM_6.C [file 267_2026_2393_MOESM10_ESM.pdf]

## Photosynthesis

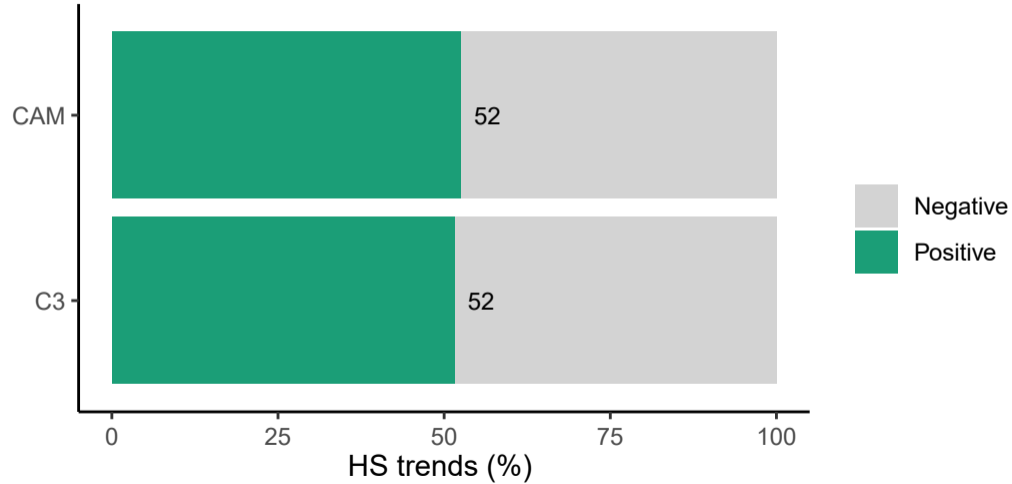

## Reproduction

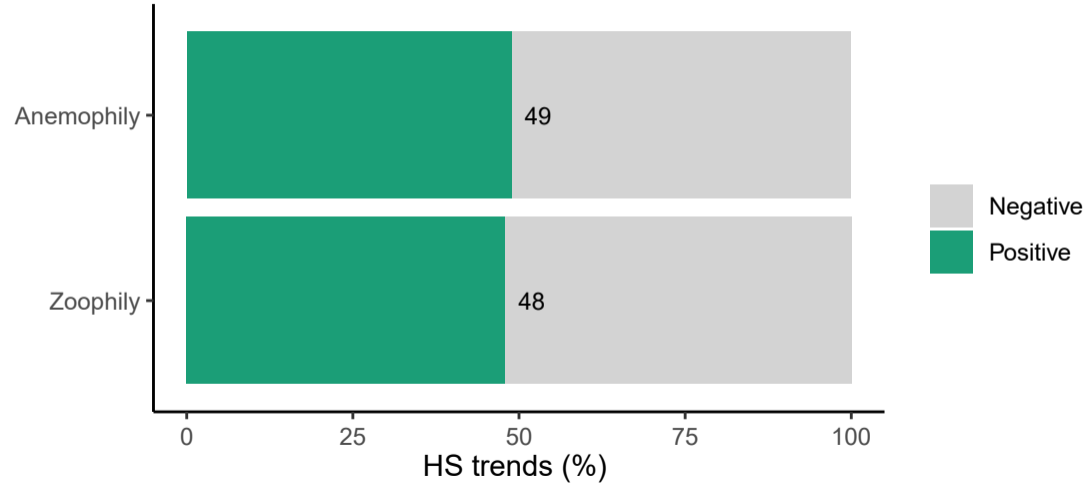

Supplement: Supplementary file 11 — ESM_6.D [file 267_2026_2393_MOESM11_ESM.pdf]
